# Double-Blind Controlled Randomized Trial of Cyclophosphamide versus Methylprednisolone in Secondary Progressive Multiple Sclerosis

**DOI:** 10.1371/journal.pone.0168834

**Published:** 2017-01-03

**Authors:** Bruno Brochet, Mathilde S. A. Deloire, Paul Perez, Timothé Loock, Louise Baschet, Marc Debouverie, Sophie Pittion, Jean-Christophe Ouallet, Pierre Clavelou, Jérôme de Sèze, Nicolas Collongues, Patrick Vermersch, Hélène Zéphir, Giovanni Castelnovo, Pierre Labauge, Christine Lebrun, Mikael Cohen, Aurélie Ruet, PROMESS study investigators

**Affiliations:** 1 Service de Neurologie et INSERM-CHU CIC-P 0005, CHU de Bordeaux, Bordeaux, France; 2 INSERM U 1215, Université de Bordeaux, Bordeaux, France; 3 Unité de Soutien Méthodologique à la Recherche Clinique et Epidémiologique, Pôle de Santé Publique, CHU de Bordeaux, Bordeaux France; 4 Service de Neurologie, CHU de Nancy, Nancy, France; 5 Service de Neurologie, CHU de Clermont-Ferrand, Clermont-Ferrand, France; 6 Service de Neurologie et CIC INSERM 1434, CHU de Strasbourg, Strasbourg, France; 7 Univ. Lille, CHU Lille, LIRIC-INSERM U995, FHU Imminent, Lille, France; 8 Service de Neurologie, CHU de Nîmes, Nîmes, France; 9 Service de Neurologie, CHU de Montpellier, Montpellier, France; 10 Service de Neurologie, CHU de Nice, Nice, France; University Medical Center Gottingen, GERMANY

## Abstract

**Background:**

Therapeutic options are limited in secondary progressive multiple sclerosis (SPMS). Open-label studies suggested efficacy of monthly IV cyclophosphamide (CPM) without induction for delaying progression but no randomized trial was conducted so far.

**Objective:**

To compare CPM to methylprednisolone (MP) in SPMS.

**Methods:**

Randomized, double-blind clinical trial on two parallel groups. Patient with SPMS, with a documented worsening of the Expanded Disability Status Scale (EDSS) score during the last year and an EDSS score between 4·0 and 6·5 were recruited and received one intravenous infusion of treatment (CPM: 750 mg /m^2^ body surface area—MP: 1g) every four weeks for one year, and every eight weeks for the second year. The primary endpoint was the time to EDSS deterioration, when confirmed sixteen weeks later, analyzed using a Cox model.

**Results:**

Due to recruitment difficulties, the study was terminated prematurely after 138 patients were included (CPM, n = 72; MP, n = 66). In the CPM group, 33 patients stopped treatment prematurely, mainly due to tolerability, compared with 22 in the MP group. Primary endpoint: the hazard ratio for EDSS deterioration in the CPM in comparison with the MP group was 0.61 [95% CI: 0·31–1·22](p = 0·16). According to the secondary multistate model analysis, patients in the CPM group were 2.2 times more likely ([1·14–4.29]; p = 0.02) to discontinue treatment than those in the MP group and 2.7 times less likely (HR = 0.37, 95% CI: 0.17–0.84; p = 0.02) to experience disability progression when they did not stop treatment prematurely. Safety profile was as expected.

**Conclusion:**

Although the primary end-point was negative, secondary analysis suggested that CPM decreases the risk of progression in SPMS, but its use may be limited by low tolerability.

**Trial Registration:**

Clinicaltrials.gov NCT00241254

## Introduction

Therapeutic options for multiple sclerosis (MS) have dramatically increased in the past 20 years. However, such treatments primarily benefit patients with relapsing-remitting MS. Until recently, only a few drugs have been tested in randomized controlled trials (RCTs) in progressive MS (PMS), including Interferon-β [1–8]. The inconsistent results of these trials led to the interpretation that secondary PMS (SPMS) may not be affected by immunological treatment because the main pathological substratum of disability progression is neurodegeneration [9]. However, recent pathological studies have suggested that inflammation still plays a role in SPMS and is associated with axonal injury [10–12]. Meningeal inflammation has been described in SPMS and it has been suggested that it may play a major role in the development of disability [13]. The inflammatory process observed in SPMS has been described as being confined to the brain or the meninges, with limited trafficking of blood-derived immune cells across the blood-brain barrier [10,13]. Therefore their inability to reach the central nervous system may explain the failure of most drugs in SPMS. Immunosuppressants have been proposed to treat PMS for many years [14,15]. Methotrexate [14] and mitoxantrone [15] have been studied in RCTs in patients with various clinical phenotypes including PMS, suggesting that immunosuppressive therapy could be useful in PMS, especially in patients showing evidence of active inflammation (relapses or lesion activity on magnetic resonance imaging) [13]. The Mitoxantrone trial included 50% of SPMS patients and showed a positive effect of mitoxantrone over placebo on the primary outcome which was a a multivariate analysis of five clinical measures [15]. Cyclophosphamide (CPM), a nitrogen mustard alkylating agent, is able to cross the blood-brain-barrier and has been considered as a treatment for many years [16]. Several RCTs of intravenous (IV) CPM have been conducted in PMS, using an induction regimen without maintenance therapy, with contradictory results [17–19]. The absence of maintenance therapy could explain the lack of sustained effect [20]. Repeated administration of CPM has also been proposed [20] supporting the use of maintenance therapy. Based on these results monthly administration of IV CPM has been used in several centres and encouraging results regarding efficacy and an acceptable safety profile have been reported in a large retrospective study [21]. To date, no RCT has studied the effect of IV pulses of CPM in SPMS without an induction regimen. The objectives of the present randomized controlled trial were to assess the efficacy of CPM in delaying disability progression, and the safety of IV pulses of CPM administered over two years, relative to IV pulses of methylprednisolone (MP). Considering that MP pulse therapy was which is frequently used in centres in France to treat SPMS after promising results emerged from a pilot study [22], Tthe steering committee considered that the use of this comparator will increase the feasibility of the trial.

## Patients and Methods

### Study design and patients

The PROgressive MultiplE Sclerosis Study (PROMESS) study was a two parallel group double-blind RCT. Eligible patients were men and women, 18–65 years of age, with SPMS [23,24], enrolled from 27 French Neurology departments. SPMS was defined by a gradual progression of disability for at least six months and less than four years. Additionally, a documented worsening on the Expanded Disability Status Scale (EDSS) [25] of at least 0·5 points in the last 12 months that was not due to a relapse and a reduction in walking distance in the past year were required for selecting patients with active progression. The baseline EDSS score had to be between 4·0 and 6·5, inclusive. Key exclusion criteria included any contraindications to the study drugs. An independent committee reviewed issues relating to safety. The study was coordinated by the coordinating center (CC) based at the University Hospital (CHU), Bordeaux. The study was conducted in accordance with the international guidelines for Good Clinical Practice and the principles of the Declaration of Helsinki. Patients provided written informed consent. The study was approved by the regional ethics committee (“CPP Sud-Ouest Outre-Mer”, N°2005/09) and registered on Clinicaltrials.gov: NCT00241254.

### Randomization and masking

Patients were allocated to receive either CPM or MP using a web-based secured system according to a randomization list generated and kept confidential by the statistician of the Clinical Trials Unit (CTU, CHU Bordeaux) using SAS 9·1^©^. Randomization, with a 1:1 ratio, was stratified by centre, with blocks of size four for small sites and size six for others. All study personnel were blinded to group allocation, including the neurologists and nurses administrating the treatments. Because the study drugs have potential adverse effects that may make patients and clinicians guess the treatment received, outcome assessments were performed by an evaluating neurologist (EN); the treating neurologist (TN) evaluated the clinical state, safety and tolerability. Study drugs were prepared in hospital pharmacies in similar infusion vials that precluded the identification of the group assignment by patients and study personnel.

### Treatments

Patients received either IV CPM (750 mg /m^2^ body surface area) or IV MP (1 g), administered every four weeks for one year and every eight weeks during the second year. The procedures for the administration of the study drugs were similar in both groups. All patients received a 5% glucose serum infusion spanning eight hours, with the study drug being infused during the first three hours, followed by serum alone for five hours. The purpose of this was to reduce the risk of toxic cystitis, which was expected with CPM treatment only. To reduce the incidence of gastrointestinal adverse events related to infusion, all patients received three IV injections of either an anti-emetic treatment (ondansetron, 8 mg) in the CPM group or a placebo in the MP group, in a double-blind manner. Three days before each admission all patients underwent laboratory testing to count peripheral blood cells, analyze sodium, potassium, and hepatic enzymes, and test renal function. The laboratory sent the results to a prescribing physician (PP) located at the CC who was not blinded to treatment allocation. Depending if the results were in the range of pre-defined normal values or not, the PP sent the prescription to the hospital pharmacist in the clinical centre, either with a standard, or with an adapted study drug dosage according to a pre-defined algorithm.

According to the TN’s decision, patients with relapses could receive IV MP one gram for five days. Relapses were defined as new or worsening neurological symptoms attributable to multiple sclerosis, lasting at least 48 h, without pyrexia, after at least 30 days of clinical stability with an EDSS increase of at least 0.5 points or an FSS increase of 1 point.

### Endpoints

The primary outcome was the time to sustained accumulation of disability over 16 weeks, defined as an increase from baseline of at least one EDSS point when the baseline EDSS was 4 or 4·5, or at least 0·5 points if the baseline EDSS was between 5·0 and 6·5, confirmed 16 weeks later. This 16-weeks interval was chosen because patients went at the clinic every two months during the second year for treatment. In order to limit inter-rater variability in EDSS grading, the ENs were asked to perform a standardized neurological examination, to grade Functional Status Scores (FSS) according to an adapted French version of the EDSS [25] and to objectively measure the patient’s walking distance up to 500 meters according to their functional ability. The results were sent to the CC, where the EDSS scores were established according to a pre-defined algorithm [25].

Key secondary outcomes were the proportion of patients with sustained EDSS progression at 2 years, MS Functional Composite (MSFC) z scores progression, the relapse rate, and the proportion of relapse-free patients. When patients refused to go on with their follow-up visits during the course of the trial, efforts were made for obtaining the outcome criteria at their two-year visit. Treatment failure was defined as either EDSS progression at 2 years or missing data on EDSS at 2 years due to early discontinuation of treatment because of adverse events. Adverse events were collected according to a standardized method using the EudraVigilance database.

### Sample size

The sample size calculation was based on a Log-Rank test, assuming an expected proportion of patients without EDSS progression at two years of 75% in the CPM group and 60% in the MP group. The type I error (two-sided) was fixed at 5% and the chosen power was 80%. Under these assumptions, 155 patients had to be included in each group (nQuery V 7.0). For taking into account 5% of drop outs and possible protocol deviations, 360 patients in total were planned to be recruited.

Study enrolment and retention of patients were slower than expected. As a result, in July 2009 enrolment was terminated at 138 patients.

### Statistical analysis

For the main analysis of the primary endpoint (time to sustained accumulation of disability over 16 weeks), an intention-to-treat strategy was employed. A survival frailty model that considered sites as random effects was first fitted. If the variance of the random effect was found to be negligible, a Cox model was fitted with and without adjustment [26]. Adjustment covariates were the presence of relapses in the year preceding the trial and the disease duration. The interaction between treatment effect and relapses was tested. The time to confirmed EDSS progression was censored whenever patients stopped follow-up before progression.

Due to the high number of treatment discontinuations the statistical analysis plan was revised and validated before the database freeze and treatment unblinding, including a pre-planned secondary analysis jointly modelling delays of early discontinuation of treatment and of confirmed EDSS progression, using a multistate model of the “illness-death” type [27,28] with and without adjustment, which was fit using the SmoothHazard R package [29].

Secondary endpoints were analyzed using logistic regression for dichotomous outcomes, linear regression for continuous outcomes. Comparisons were adjusted on covariates with sites as random effects when possible.

The R package msm, and more complex models with disability progression occurrence made possible after treatment discontinuation, were used as robustness analyses for the main multistate model [28–29]. In the analyses of secondary endpoints, missing data due to early discontinuation of treatment because of adverse events were considered treatment failures as were progressions. Based upon the sample size that there was little power to test the interaction effect. All analyses were performed by the biostatistician from the CTU. Except for multistate models, SAS 9·2^®^ was used. Type I error was fixed at 5%.

## Results

### Patient characteristics

One hundred and forty-eight patients were included between 11/16/2005 and 07/16/2009, when recruitment closed. Due to a low recruitment rate, it was decided to stop the study at this stage. Seventy-two patients were randomly allocated to receive CPM and 66 to receive MP treatment. Table 1 summarizes the baseline characteristics of the patients.

**Table 1:** Baseline characteristics, PROMESS trial, France.

	CPM (n = 72)	MP (n = 66)
Age*	48·6 (9·0)	46·8 (9·0)
Gender (% women)	62.0	68.2
Disease duration (y)*	13·9 (7·3)	11.9 (5·6)
Secondary progressive phase duration (y)*	1.9 (1·0)	1·9 (1·0)
% with relapse in the previous year	33·0	37·9
% with relapse in the SP phase	40·8	50·0
Median EDSS [Q1-Q3]	5 [4–6]	5 [4–6]
MSFC z scores*	-0·5 (0·7)	-0·3 (0·8)

* = mean (standard deviation);

CPM = Cyclophosphamide; MP = Methylprednisolone; EDSS = Expanded Disability Status Scale; MSFC = Multiple Sclerosis Functional Composite.

**Fig 1:**
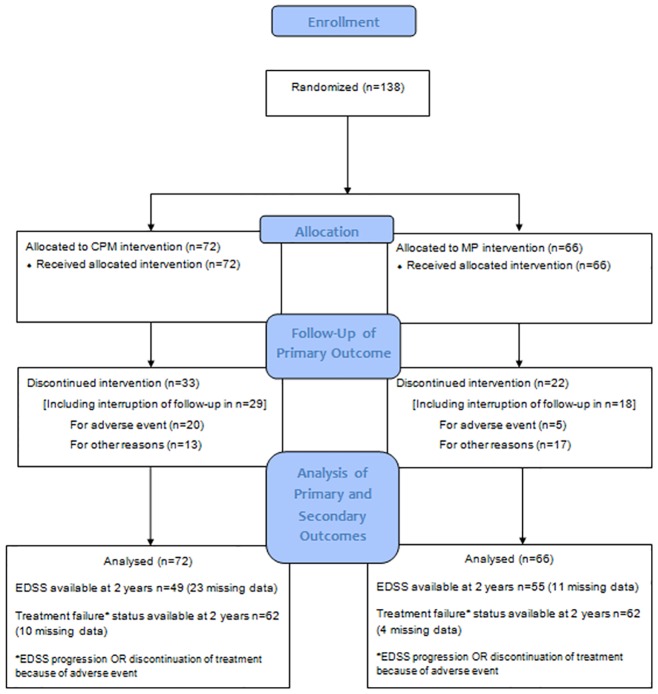
Flow chart of the PROMESS trial, France.

Fifty—five patients overall discontinued treatment, more frequently in the CPM than the MP arm (Fig 1).

Among the 33 patients who discontinued treatment in the CPM arm, 20 did so because of adverse events, compared to five out of 22 in the MP group. A high proportion of patients discontinuing treatment also stopped coming to follow-up visits (29 out of 33 in the CPM group and 18 out of 20 in the MP group), generating missing data on the primary outcome. The time to possible disability progression was therefore not possible to determine for those patients who were censored in the primary analysis. The mean follow-up duration was 87 weeks (Standard deviation (SD) = 37) in the CPM group and 95 weeks (SD = 26) in the MP group (p = 0.15).

### Outcomes

#### Primary endpoint

The proportion of patients with sustained EDSS progression at 2 years was 18·1% (n = 13) in the CPM group and 31·8% (n = 21) in the MP group (p = 0·06), but a high proportion of patients could not be assessed for this outcome, more frequently in the CPM group than in the MP group (Table 2).

**Table 2:** Secondary clinical endpoints, PROMESS trial, France.

	CPM (n = 72) n (%)	MP (n = 66) n (%)	p value*
EDSS during follow-up			0.21
sustained EDSS progression	13 (18·1)	21 (31·8)	
no sustained EDSS progression during 2 years	36 (50·0)	34 (51·5)	
loss to follow-up before progression	23 (31·9)	11 (16·7)	
Two-year treatment failure**			0.59
Yes	29 (46·8)	26 (41·9)	
No	33 (53·2)	36 (58·1)	
missing data	10	4	
Number of relapses during follow-up			0.17
0	53 (73·6)	39 (59·1)	
1	12 (16·7)	15 (22·7)	
>1	7 (9·7)	12 (18·2)	
Annualized relapse rate	0.3 (0.6)	0.4 (0.5)	0.12

* Chi-square test

** Failure was defined as EDSS progression or discontinuation of treatment because of an adverse event;

CPM = Cyclophosphamide; MP = Methylprednisolone; EDSS = Expanded Disability Status Scale.

According to the Cox model, where those patients were censored at the time of stopping their follow-up, the adjusted hazard ratio of progression was 0.61 (95% CI: 0.31–1.22) for the CPM group in comparison with the MP group (p = 0.16). The treatment effect was not different depending on the presence of relapses during the 12 months before inclusion: the interaction was not statistically significant (p = 0.74) and the inclusion of relapses in the Cox model did not change efficacy results.

#### Secondary analyses

**Fig 2:**
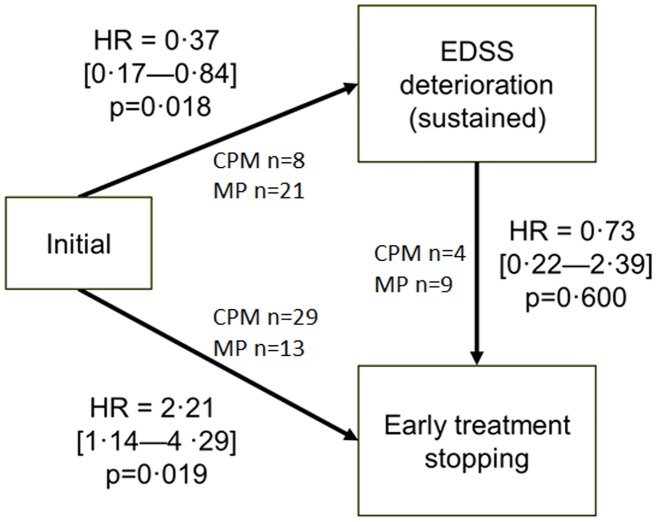
Multistate “illness-death” analysis scheme and results. This figure describes the risks of transition undergone by trial participants between three states. Of special interest are transitions from the initial state (at inclusion) to either EDSS deterioration (primary outcome) or early treatment discontinuation. Risks are expressed as hazard ratios (HR) (and their 95% confidence intervals), i.e., the risk of transition for the experimental (CPM) group relative to the control (MP) group of patients. When the HR is < 1, the risk of transition is decreased (treatment efficacy), and when it is > 1, the risk of transition is increased.

The multistate model assesses the risk for a patient to change from a state to another (from the state at inclusion to either EDSS deterioration or treatment discontinuation). According to this analysis, patients in the CPM group were 2.2 times more likely to discontinue their treatment as compared to the MP group (HR = 2.21; 95% CI: 1.14–4.29), but they were 2.7 times more likely not to experience sustained EDSS progression (HR = 0.37; 95% CI: 0.17–0.84) (Fig 2). The two transition HRs were statistically significant (p = 0.019 and p = 0.018) showing that patients had more chance to change from “on treatment” state to “discontinued treatment” state and from “stable EDSS” state to “deteriorated EDSS” state). In other words patients not discontinuing treatment were more likely to benefit from CPM than MP.

From the initial state (at inclusion), patients may evolve to EDSS deterioration or early treatment stopping. HR is the point estimate of the Hazard Ratio of transition from one state to the other for the CPM group in comparison to the MP group; the 95% confidence interval of HR is given between brackets. The number of patients is given for each transition in each group.

As a sensitivity analysis, we estimated the proportion of patients with treatment failure at two years in the two groups (failure was defined as either EDSS progression or early discontinuation of treatment due to an adverse event). Treatment failure occurred in 46·8% of the patients from the CPM group (N = 62, 10 missing data) and in 41·9% from the MP group (N = 62, four missing data) (p = 0.59).

The change in the MSFC z scores (CMP: -0.8 vs. MP: -0.5, p = 0.33) and its components at two years did not differ between groups. The proportion of relapse-free patients was 73·6% in the CPM group compared to 56·1% in the MP group (p = 0·07), but the follow-up duration differed between groups.

### Adverse events

A similar proportion of patients in the two treatment groups had adverse events (CPM: 97·2% versus MP: 92·4%), most of which were mild to moderate in severity (Tables 3 and 4). A similar proportion of patients in the two treatment groups had adverse events (CPM: 97·2% versus MP: 92·4%, p = 0.20), most of which were mild to moderate in severity (Tables 3 and 4). The proportion of serious adverse events was not different between the two groups (CPM: 22.2% vs. MP: 19.7%, p = 0.72)

**Table 3:** Adverse events, PROMESS trial, France.

System organ class	CPM (n = 72)		MP (n = 66)	
	Number of AE	Number (%) of patients with AE	Number of AE	Number (%) of patients with AE
All pooled system organ classes	617	70 (97.2)	409	61 (92.4)
Gastrointestinal disorders	280	59 (81.9)	71	28 (42.4)
Infections and infestations	129	42 (58.3)	109	38 (57.6)
Nervous system disorders	68	34 (47.2)	78	38 (57.6)
General disorders and administration site conditions	30	23 (31.9)	33	22 (33.3)
Musculoskeletal and connective tissue disorders	17	14 (19.4)	21	12 (18.2)
Skin and subcutaneous tissue disorders	18	15 (20.8)	17	11 (16.7)
Investigations	13	10 (13.9)	15	10 (15.2)
Injury, poisoning and procedural complications	16	12 (16.7)	10	10 (15.2)
Psychiatric disorders	9	8 (11.1)	8	7 (10.6)
Other	37	30 (41.7)	47	34 (51.5)

CPM = Cyclophosphamide; MP = Methylprednisolone; AE = Adverse Events

**Table 4:** Serious adverse events.

Group	CPM (n = 72)	MP (n = 66)
Serious Adverse Events (n)	Sudden death of probable cardio-vascular origin (1)Malignancies (2): one ovarian and one breast cancer, both occurred during the first year of treatment Pericarditis (1)Psychotic episode (1) Rash (1) Hospitalization for surgery (2) Thrombophlebitis (1)Osteonecrosis (1)Infections (2) Fractures (2)Hyperglycemia (1)Extravasation (1)	Myocardial infarction (1)Pulmonary embolism (1)Pneumonia (1)Seizure (1) Pancreatitis (1) Fever (1) Suicide attempt (1)Fractures (2) Wound (1) Surgery (1)Miscellaneous (2)

CPM = Cyclophosphamide; MP = Methylprednisolone;

## Discussion

After 25 years the conversion rate of patients with relapsing-onset MS towards SPMS approaches 70% [30]. Although this clinical phenotype is associated with severe disability, there are still few therapeutic options [13]. Disease-modifying therapies used in RRMS have usually no or little efficacy in PMS. There is, therefore, a need for new therapeutic options in SPMS. The mechanisms underlying disability in SPMS associates both inflammation and neurodegeneration. It is generally accepted that the inflammatory component is more important at the early stages of SPMS which are, therefore, probably, more accessible to immunosuppressive therapy [13]. The population selected for the PROMESS trial is characteristic of early SPMS with a recent onset of the SPMS phase and relapses present in more than 30% of patients in the previous year.

The statistical analysis was performed using an intention-to-treat strategy. The primary analysis using a Cox model did not show a statistically significant superiority of CPM in comparison with MP for preventing sustained EDSS progression in the 138 included patients. This recruitment was inferior to what was planned and a high proportion of patients stopped treatment and follow-up early, more frequently in the CPM group. To take into account the possibility that patient drop-outs precluding the assessment of the primary outcome may result in informative censoring threatening the validity of the Cox model, a multistate model analysis was conducted. This alternative analysis concluded that patients in the CPM group were significantly more likely to stop treatment, but also that those going on their treatment had significantly less EDSS progression. This may be interpreted as a possible indication of the efficacy of CPM in patients who were able to tolerate and continue their CPM treatment. There was also a trend in favour of CPM when examining the proportion of patients with sustained disability progression and the proportion of relapse-free patients at two years.

The primary analysis using a Cox model showed a not statistically significant reduction in the incidence of progression in the CPM group (HR = 0·61). The results of the Cox model are difficult to interpret because the trial was underpowered, and because a high proportion of patients stopped their follow-up before two years with this proportion being much higher in the CPM group. In survival analysis those patients are censored at the time of discontinuation of follow-up, but this analysis is valid only as long as censoring is non informative [26]. In the case when missing data are not completely at random, especially when they are related to the primary outcome with a proportion of censored patients different in the two groups, results of the Cox model may be biased. Here, missing data were often related to early discontinuation of treatment and the inability to convince patients to come to follow-up visits thereafter. To consider that discontinuation of follow-up was completely at random, i.e. independent from the risk of disability progression is a strong assumption which was impossible to check. The main reason for treatment discontinuation was low tolerability which explains that the drop-out rate was superior to those usually observed in some PMS trials but not all [31]. Among other causes of discontinuation no specific reason emerged. In particular, no other therapeutic trial was conducted at the same time.

Because of the high number of treatment discontinuations, a secondary analysis was planned (before database freeze and treatment unblinding) using a multistate model that could estimate the hazard of transition to disability progression in both randomization groups while taking into account at the same time the possible transition to early discontinuation of treatment. Hence, as indicated by ITT principles, all patients were analysed in their randomization group, although the transition toward disability progression was estimated only in a patient subgroup, those who did not discontinue treatment. All sensitivity analyses with different multistate models and statistical packages found a significant effect of CPM in delaying disability progression compared to MP. The non-significant result from the Cox model may be explained by the limited power of the trial due to the recruitment of less than half of the planned sample size and the number of censored observations.

Although the use of CPM has been proposed for MS since 1966 [16] and the first study was performed in 1967 [32], very few RCT examining the efficacy of CPM in PMS have been performed. In an uncontrolled, open-label trial in 86 patients with PMS, stabilization of the disease was reported for 1–5 years after a short course of CPM in 69% of cases [33]. In the first RCT, 58 patients with PMS were randomized to receive either a 2–3 week course of IV CPM to achieve a leucopoenia combined with adrenocorticotrophic hormone (ACTH), or ACTH alone, or plasma exchange with ACTH and oral CPM [17]. Positive results were reported, as 16 out of 20 patients in the CPM group improved or stabilized at 48 weeks, which was superior to other groups. The results were not confirmed by a small study in which CPM was compared to folic acid, used as a placebo [21], or by a double-blind RCT [19]. Limited sample sizes, inadequate inclusion criteria, the absence of blinding procedures and a lack of maintenance therapy may explain these inconsistent results.

An open-label study compared 261 patients with PMS who were randomized to receive either one induction treatment followed by a booster every two months over a two-year period or no pulse therapy [20]. A significant benefit, although modest, was found in the patients receiving booster therapy. The results were better in younger patients and patients with recent progression onset.

In the present trial, we selected patients with less than four years of progression, a recent documented worsening of disability, and a reduced walking distance in the past year. It is likely that the treatment of inflammation is more relevant in the early stages of SPMS than in later stages. However, taking into account relapses in the previous year did not influence the results.

It is difficult to compare the effect size of the treatment with CPM in SPMS with other drugs than MP. Rituximab and Ocrelizumab have not been tested in SPMS. The population of the mitoxantrone study^15^ was very different with only 50% of patients with SPMS and with a very slow progression even in the placebo group (22% deteriorating over 2 years). Simvastatin has shown a positive effect on brain atrophy in phase 2 trial in SPMS [34]. A phase 3 trial is needed to study its efficacy on clinical outcome. The IVIG trial did not show positive results in the SPMS subgroup [31].

The safety profile was as expected. Besides digestive side effects CPM was generally well tolerated. Some serious adverse events were reported in the two groups but were relatively balanced. We did not observe an increase in infections and severe infections in the CPM group, or haematological or bladder malignancies. A risk of bladder cancer has been reported with high accumulative doses [35], but not at the dosages used in MS [36]. However the duration of the study was probably too short to reliably assess this risk. Microscopic haematuria occurred in four patients with CPM and one with MP, but no symptomatic haemorrhagic cystitis occurred. The only difference between the two groups concerning side effects was seen in the gastrointestinal system, with 48 patients having nausea and 31 patients experiencing vomiting in the CPM group compared to 19 and three in the MP group. This low gastric tolerability, despite the use of ondansetron, explains the higher drop-out rate observed in the CPM group. These digestive adverse events are a clear limitation for CPM use but could be overcome by using more powerful new anti-emetic agents like aprepitant [37]. The safety profile of CPM seems better than the safety profile of mitoxantrone, which exposes to cardiac toxicity and leukaemia [38].

One limitation of the study is its limited power, due to a low rate of inclusions. This low inclusion rate could be explained, at least in part, by the fact that the two study drugs were available and used in many centres in France, without the need to bear the constraints related to the participation in a clinical trial. However, during the trial, participating centres proposed systematically to eligible patients to participate to the study. The baseline characteristics of the patients (age, EDSS, MSFC z score) which are very similar to a recent sample of SPMS patients enrolled in a therapeutic trial [33] do not suggest that this has biased significantly the recruitment of the study. Moreover blinding and randomisation contributed to a bias-free selection of patients included in the study and a good internal validity. The relatively high number of treatment discontinuation highlights the difficulty of running SPMS trials. In a controlled trial of IV immunoglobulin against placebo in PMS, the high number of treatment discontinuation in SPMS patients make difficult the interpretation of the results [31].

One possible limitation of the study is that digestive side effects may have precluded the correct blinding of the patients. However, all patients included were informed that both drugs may or may not induce this type of side effect, and all evaluations were performed by a blinded neurologist who was unaware of the side effects possibly reported by the patients to their treating neurologist. The two groups differed according to the use of ondansetron in the CPM group. Although ondansetron, which is a 5HT3 antagonist, has been proposed to treat cerebellar tremor [39], its efficacy has never been confirmed and an effect on neurological disability in MS is very unlikely.

Another possible limitation is the use of MP as a comparator. As mentioned above, although placebo-controlled studies are certainly better for demonstrating treatment efficacy, MP IV pulse therapy was proposed as an active comparator after the results of a phase II trial comparing high-dose IV MP (500mg) to low doses (10mg) every 8 weeks [22]. The primary end-point of this study was negative (a binomial comparison of proportions of sustained treatment failures on a composite outcome at 2 years) but a treatment effect was observed according to a comparison of time to onset of sustained progression of disability. A recent study of pulse oral MP failed to show an effect on intrathecal inflammation in PMS [40].

In spite of these limitations, although the primary endpoint was negative, the PROMESS study represents the best available evidence to date that IV pulses of CPM may offer a delay in disability progression for patients in the first years of SPMS, at least for those who are able to receive the entire two-year treatment. Using new, more potent antiemetic drugs may allow a longer treatment with CPM, possibly resulting in improved efficacy for patients. Aggressive immunosuppression is an option for a subgroup of patients in SPMS, which remains to be characterized, but has overall only limited efficacy in progressive MS.

## Supporting Information

S1 File SUPPLEMENT TO SUBMISSION. (DOC)

S2 File PROTOCOL SYNOPSYS. (DOCX)

S3 File PromessClinTrialsGovRecord. (PDF)

S4 File CONSORT 2010 Checklist promess. (DOC)
