# Supplementary material for: Double-Blind Controlled Randomized Trial of Cyclophosphamide versus Methylprednisolone in Secondary Progressive Multiple Sclerosis
Source: PLoS One. 2017 Jan 3;12(1):e0168834. doi: 10.1371/journal.pone.0168834 (PMC5207788; doi:10.1371/journal.pone.0168834)
Supplement: S1 File — (DOC) [file pone.0168834.s001.doc]

SUPPORTING INFORMATION

Double-Blind controlled randomized trial of cyclophosphamide versus methylprednisolone in secondary progressive multiple sclerosis

BROCHET B, et al.

"This supplement (Part 1) contains the following items:

1. a/ Original protocol (page 2) ,
 b/ final protocol (p 105),

c/ summary of changes. (p 189)

2. Statistical analysis plan,
 a) Introduction and summary of changes"(p 190)

b) Analysis Plan (p191)

1. Original protocol (03/02/2005)

**ESSAI THERAPEUTIQUE RANDOMISE MULTICENTRIQUE**

**EN DOUBLE INSU EN GROUPES PARALLELES DU**

**CYCLOPHOSPHAMIDE INTRAVEINEUX VERSUS METHYLPREDNISOLONE DANS LE TRAITEMENT DE FOND DES FORMES PROGRESSIVES SECONDAIRES RECENTES DE SCLEROSE EN PLAQUES :**

**ETUDE P.R.OM.E.S.S**

Titre abrégé : Essai randomisé multicentrique en double insu cyclophosphamide versus méthylprednisolone dans le traitement des formes progressives secondaires de sclérose en plaques : Etude P.R.OM.E.S.S.

**RECHERCHE AVEC BENEFICE INDIVIDUEL DIRECT**

Promoteur :

CHU de Bordeaux, Direction Générale du CHU de Bordeaux,

Unité de gestion de la recherche clinique

12 rue Dubernat, 33404 Talence

Tel : 05 56 79 47 98 Fax 05 56 79 49 26

Site internet : [www.chu-bordeaux.fr](http://www.chu-bordeaux.fr/)

Investigateur Coordonnateur :

Pr Bruno Brochet,

Service de Neurologie

Fédération des Neurosciences Cliniques, CHU de Bordeaux.

Hôpital Pellegrin CHU de Bordeaux.

Place Amélie Raba Leon, 33076 Bordeaux cedex

Tel 05 56 79 55 21 Fax 05 56 79 49 51

[bruno.brochet@chu-bordeaux.fr](mailto:bruno.brochet@chu-bordeaux.fr)

**II. LISTE DES PRINCIPAUX RESPONSABLES POUR LE PROTOCOLE :**

**Promoteur** : CHU de Bordeaux, rue Dubernat, 33400 Talence

**Investigateur Coordonnateur** : Pr Bruno Brochet, Service de Neurologie, Fédération des

Neurosciences Cliniques, CHU de Bordeaux, 33076 Bordeaux Cedex, France.

Tél. : 05 56 79 55 21

Fax : 05 56 79 49 51

Email : [bruno.brochet@chu-bordeaux.fr](mailto:bruno.brochet@chu-bordeaux.fr)

**Méthodologiste Coordonnateur** : Dr Paul Perez, USMR du CHU de Bordeaux, Bât

ISPED, Université Victor Segalen, 146 rue Léo Saignat, Case 11, 33076 Bordeaux Cedex,

France :

Tél.: 05 57 57 11 29

Fax : 05 57 57 15 78

Email : [usmr@isped.u-bordeaux2.fr](mailto:usmr@isped.u-bordeaux2.fr)

**Responsable Pharmacie, coordonnateur** : Dr Jean Grellet, Pharmacie centrale, CHU de Bordeaux, 33076 Bordeaux Cedex, France.

Tél.: 05 56 79 55 03

Fax : 05 56 79 56 74

Email : [jean.grellet@chu-bordeaux.fr](mailto:jean.grellet@chu-bordeaux.fr)

**Chef de projet:** Mathilde Deloire, EA 2966, Université Victor Segalen ,146 rue Léo

Saignat, case 78 , 33076 Bordeaux Cedex, France :

Tél. : 05 57 57 57 48 17

Fax : 05 57 57 48 18

Email : [mathilde.grassin@bb-luni.u-bordeaux2.fr](mailto:mathilde.grassin@bb-luni.u-bordeaux2.fr)

**Liste des centres et des investigateurs principaux :**

CHU Bordeaux:

Pr Bruno Brochet, Service de Neurologie B, CHU Pellegrin, 33076 Bordeaux cedex,

Tél. :05 56 79 55 21, Fax : 05 56 79 49 51, courriel : [bruno.brochet@chu-bordeaux.fr](mailto:bruno.brochet@chu-bordeaux.fr).

Dr Jean-Chirstophe Ouallet, Service de Neurologie B, CHU Pellegrin, 33076 Bordeaux cedex,

Tél. : 05 56 79 55 21, Fax : 05 56 79 60 25, courriel : [jean-christophe.ouallet@luni.u-bordeaux2.fr](mailto:jean-christophe.ouallet@luni.u-bordeaux2.fr).

CH d’Angoulême :

Dr Jean-Luc Devoize, Service de Neurologie, CH de Girac, 16470 Angoulême cedex,

Tél : 02 45 24 40 84, Fax : 02 45 24 60 90, courriel : [jeanlouis.devoize@ch-angouleme.rss.fr](mailto:jeanlouis.devoize@ch-angouleme.fr).

Dr Christophe Prat, Service de Neurologie, CH de Girac, 16470 Angoulême cedex,

Tél : 02 45 24 40 84, Fax : 02 45 24 60 90, courriel : [christophe.prat@ch-angouleme.rss.fr](mailto:christophe.prat@ch-angouleme.rss.fr).

CH de Bayonne :

Dr Emmanuel Ellie, Service de Neurologie, CH de la Côte Basque, 64109 Bayonne, Tél : 05 59 44 37 12, Fax : 05 59 44 37 19, courriel : [eellie001@CHCB.rss.fr](mailto:eellie001@CHCB.rss.fr).

CHU de Besançon :

Pr Lucien Rumbach, Service de Neurologie, CHU de Besançon, 25000 Besançon, Tél : 03 81 66 80 98, Fax : 03 81 66 84 70, courriel : [lrumbach@chu-besancon.fr](mailto:lrumbach@chu-besancon.fr).

CHU de Caen :

Pr Gilles Defer, Service de Neurologie, CHU de Caen, 14033 Caen cedex 5, Tél : 02 31 06 46 21, Fax : 02 31 06 46 27, courriel : [defer-g@chu-caen.fr](mailto:defer-g@chu-caen.fr).

CHU de Clermont-Ferrand :

Pr Pierre Clavelou, Service de Neurologie, CHU de Clermont-Ferrand, 63003 Clermont-Ferrand cedex1, Tél : 04 73 75 22 01, Fax : 04 73 75 22 02, courriel : [pclavelou@chu-clermontferrand.fr](mailto:pclavelou@chu-clermontferrand.fr).

CHU de Dijon :

Pr Thibault Moreau, Service de Neurologie, CHU de Dijon, 21000 Dijon, Tél : 03 80 29 37 53, courriel : [thibault.moreau@chu-dijon.fr](mailto:thibault.moreau@chu-dijon.fr).

Fondation Rothschild :

Dr Olivier Gout, Service de Neurologie, Fondation Ophtalmologique Rothschild, 75010 Paris cedex, Tél : 01 48 03 68 52, Fax : 01 48 03 27 10, courriel : [agout@fo.rothschild.fr](mailto:agout@fo.rothschild.fr).

CHS Philibert de Lille :

Pr Patrick Hautecoeur, Service de Neurologie, CH S’ Philibert, 59460 Lille cedex, Tél : 03 20 22 50 79, Fax : 03 20 22 33 55, courriel : Hautecoeur.Patrick@ghicl.fupl.asso.fr.

CHU de Lille :

Pr Patrick Vermersch, Service de Neurologie, CHU de Lille, 59037 Lille, Tél : 03 20 44 68 46, Fax : 03 20 44 44 84, courriel : [pvermersch@chru-lille.fr](mailto:pvermersch@chru-lille.fr).

CHU de Limoges :

Dr Laurent Magy, Service de Neurologie, CHU de Limoges, 87042 Limoges cedex, Tél : 05 55 05 65 61, Fax : 05 55 05 65 67, courriel : [laurent.magy@unilim.fr](mailto:laurent.magy@unilim.fr).

CHU de Lyon :

Pr Christian Confavreux, Service de Neurologie, Hôpital Neurologique Lyon, 69394 Lyon cedex 03, Tél : 04 72 35 75 22, Fax : 04 72 35 75 25, courriel : [christian.confavreux@chiu-lyon.fr](mailto:christian.confavreux@chiu-lyon.fr).

Dr Sandrine Blanc, Service de Neurologie, Hôpital Neurologique Lyon, 69394 Lyon cedex 03, Tél : 04 78 00 49 19, Fax : 04 78 75 30 19, courriel : [sandrine.blanc@chiu-lyon.fr](mailto:sandrine.blanc@chiu-lyon.fr).

Dr Pierre Aubertin, Service de Neurologie, Hôpital Neurologique Lyon, 69394 Lyon cedex 03,

Tél : 04 72 35 75 22, Fax : 04 72 35 75 25, courriel : [pierre.aubertin@chiu-lyon.fr](mailto:pierre.aubertin@chiu-lyon.fr).

Dr Georges Riche, Service de Neurologie, Hôpital Neurologique Lyon, 69394 Lyon cedex 03,

Tél : 04 72 35 75 22, Fax : 04 72 35 75 25, courriel : [georges.riche@chiu-lyon.fr](mailto:georges.riche@chiu-lyon.fr).

CHU de Nancy :

Dr Marc Debouverie , Service de Neurologie, CHU de Nancy, 54035 Nancy cedex, Tél : 03 83 85 12 75, Fax : 03 83 85 27 34, courriel : m. [debouverie@chu-nancy.fr](mailto:debouverie@chu-nancy.fr).

CHU de Nice :

Dr Christine Lebrun-Frenay, Service de Neurologie, CHU de Nice, 06002 Nice, Tél : 04 92 03 77 44, Fax : 04 92 03 79 07, courriel : [christine.lebrun-frenay@wanadoo.fr](mailto:christine.lebrun-frenay@wanadoo.fr).

Dr Véronique Bourg, Service de Neurologie, CHU de Nice, 06002 Nice, Tél : 04 92 03 77 44,

Fax : 04 92 03 79 07.

CHU de Nîmes :

Dr Giovanni Castelnovo, Service de Neurologie, CHU Caremeau, 30900 Nîmes, Tél : 04 66 68 32 61, Fax : 04 66 68 40 16, courriel : [giovanni.castelnovo@chu-nimes.fr](mailto:giovanni.castelnovo@chu-nimes.fr).

Pr Pierre Labauge, Service de Neurologie, CHU Caremeau, 30900 Nîmes, Tél : 04 66 68 32 61, Fax : 04 66 68 40 16, courriel : [pierre.labauge@chu-nimes.fr](mailto:pierre.labauge@chu-nimes.fr).

CHU de Marseille :

Pr Jean Pelletier, Service de Neurologie, CHU La Timone, 13385 Marseille cedex 5, Tél : 04 91 38 59 39, Fax : 04 91 38 62 56, courriel : [jean.pelletier@ap-hm.fr](mailto:jean.pelletier@ap-hm.fr).

CH de Pau :

Dr Bruno Barroso, Service de Neurologie, CH de Pau, 64046 Pau cedex, Tél : 05 59 92 43 65,

Fax : 05 59 92 67 49, courriel : [bruno.barroso@chu-pau.fr](mailto:bruno.barroso@chu-pau.fr).

Dr jean Marc Larrieu, Service de Neurologie, CH de Pau, 64046 Pau cedex, Tél : 05 59 92 43 65, Fax : 05 59 92 67 49, courriel : [jean-marc.larrieu@chu-pau.fr](mailto:jean-marc.larrieu@chu-pau.fr).

CHU de Reims**:**

Pr Serge Bakchine, Service de Neurologie, CHU de Reims, 51100 Reims cedex, Tél : 03 26 78 71 35, Fax : 03 26 78 43 19, courriel : [sbakchine@chu-reims.fr](mailto:sbakchine@chu-reims.fr).

Dr Marie pierre Chaunu, Service de Neurologie, CHU de Reims, 51100 Reims cedex, Tél : 03 26 78 71 35, Fax : 03 26 78 43 19, courriel : [mpchaunu@chu-reims.fr](mailto:mpchaunu@chu-reims.fr).

CHU de Rennes :

Pr Gilles Edan, Service de Neurologie, CHU de Rennes, 35033 Rennes cedex, Tél : 02 99 28 94 55,

Fax : 02 99 28 41 32, courriel : [gilles.edan@chu-rennes.fr](mailto:gilles.edan@chu-rennes.fr)

CHU de Toulouse**:**

Pr Michel Clanet, Service de Neurologie, CHU Purpan, 31059 Toulouse cedex, Tél : 05 61 77 20 67, Fax : 05 61 77 94 43, courriel : [clanet@cict.fr](mailto:clanet@cict.fr).

Hôpital Henri Mondor :

Pr Alain Créange, Service de Neurologie, Hôpital Henri Mondor, 94000 Créteil, Tél : 01 49 81 23 10, Fax : 01 49 81 23 26, Courriel : [alain.creange@hmn.ap-paris.fr](mailto:alain.creange@hmn.ap-paris.fr).

Hôpital Tenon :

Pr Etienne Roullet, Service de Neurologie, Hôpital Tenon, 75020 Paris, Tél : 01 56 01 66 21,

Fax : 01 56 01 72 02, courriel : [etienne.roullet@tnn.ap-hop-paris.fr](mailto:etienne.roullet@tnn.ap-hop-paris.fr)

Pr Olivier Heinzlef, Service de Neurologie, Hôpital Tenon, 75020 Paris, Tél : 01 56 01 66 21,

Fax : 01 56 01 72 02, courriel : [olivier.heinzlef@tnn.ap-hop-paris.fr](mailto:olivier.heinzlef@tnn.ap-hop-paris.fr)

Dr Caroline Bensa, Service de Neurologie, Hôpital Tenon, 75020 Paris, Tél : 01 56 01 66 21,

Fax : 01 56 01 72 02, courriel : [carobensa@netcourrier.com](mailto:carobensa@netcourrier.com)

Dr Dominique Pez, Service de Neurologie, Hôpital Tenon, 75020 Paris, Tél : 01 56 01 66 21,

Fax : 01 56 01 72 02, courriel : [dominique.pez@wanadoo.fr](mailto:dominique.pez@wanadoo.fr)

Dr Ludovic Benoist, Service de Neurologie, Hôpital Tenon, 75020 Paris, Tél : 01 56 01 66 21,

Fax : 01 56 01 72 02, courriel : [lbenoist@club-internet.fr](mailto:lbenoist@club-internet.fr)

Dr Djacoba Alain Tehindrazanarivelo, Service de Neurologie, Hôpital Tenon, 75020 Paris, Tél : 01 56 01 66 21, Fax : 01 56 01 72 02, courriel : [djacobaalain@aol.com](mailto:djacobaalain@aol.com)

**III- SOMMAIRE**
I. PAGE DE COUVERTURE ET PAGE DE GARDE
II. LISTE DES PRINCIPAUX RESPONSABLES POUR LE PROTOCOLE
III. SOMMAIRE
IV. RESUME DU PROTOCOLE
V. TEXTE DU PROTOCOLE
1. JUSTIFICATION DE L’ETUDE :
1.1 SITUATION DU PROBLEME
1.1.1 INTRODUCTION
1.1.2 ETAT DES CONNAISSANCES SUR LES TRAITAMENTS ACTUELS DES FORMES SECONDAIREMENT PROGRESSIVES DE SCLEROSE EN PLAQUES
1.1.2.1. Traitements par interféron:
1.1.2.2 Mitoxantrone :
1.1.2.3 Corticoïdes :
1.1.2.4 Traitement par cyclophosphamide :
1.1.2.4.1. Données d’efficacité 
1.1.2.4.2 Données de tolérance 
1.1.3 Retombées attendues de l’étude :
1.2 Description des traitements à l’essai :
1.2.1 Traitements à l’essai :
1.2.2 Intérêt par rapport aux traitements disponibles :
1.2.2.1 L’interféron bêta :
1.2.2.2 Mitoxantrone :
1.2.2.3 Corticoïdes :
1.3. Hypothèse testée :
1.4 Choix méthodologiques :
1.4.1 Traitement évalué :
1.4.1.1 Choix :
1.4.1.2 Choix de la dose :
1.4.2. Choix du Traitement de comparaison :
1.4.2.1.Existe-il un traitement de référence des formes SP :
1.4.2.2.Choix de la méthylprednisolone comme traitement de comparaison :
1.4.2.3 . Mode d’administration et choix des doses :
1.4.3 Méthodes de mise en œuvre du double-insu 
1.4.3.1 Problème des anti-émétiques :
1.4.3.2 Perfusion de rinçage :
1.4.3.3 Suivi biologique :
1.4.4 Choix de la population étudiée :
1.4.5 Choix du Critère de jugement principal :
1.4.6 Choix des critères secondaires :
2. OBJECTIFS
2.1 L’objectif principal
2.2 Les objectifs secondaires
3. METHODES GENERALES
3.1 Schéma d’étude :
3.2 Randomisation :
3.3 Sélection des centres investigateurs 
4. CRITERES D’ELIGIBILITE 
4.1 Critères d’inclusion:
4.2 Critères de non inclusion
4.3 Critères d’exclusion :
5 . Traitements de l’essai :
5.1 Traitements :
5.1.1 Groupe Cyclophosphamide (CPM) :
5.1.2 Groupe Méthylprednisolone (MP):
5.2 Déroulement des traitements :
6. Traitements associés :
6.1 Traitements antiémétiques

6.2 Traitements interdits :
6.3 Traitements déconseillés
6.4 Prise en charge des poussées :
7. Critères de jugement :
7.1 Critère de jugement principal 
7.2 Critères de jugement secondaire 
7.3 Critères d’évaluation exploratoires :
8. Déroulement de l’essai :
8.1 Investigateurs :
8.2 Calendrier de l’essai 
8.3 Consentement :
8.4 Visite de sélection
8.5 Période de sélection 
8.6 Visite d’inclusion  et d’initiation de traitement (visite 1°
8.7 Visites de suivi et hospitalisations pour traitement
8.8 Visites de fin de traitement et de fin d’étude
8.9 Survenue du critère principal de jugement

8.10 Levée d’insu
9. Evènements indésirables graves (EIG)
9.1 Définition et déclaration d’un évènement indésirable grave
9.1.1 Définition
9.1.2 Déclaration
9.1.3 Conduite à tenir en cas d’évènement indésirable grave (EIG)
9.2 Conduite à tenir en cas de survenue de grossesse
10. Monitorage
10.1 Organisation générale.
10.2 Cahiers d’observation
10.3 Stockage des documents
11. Surveillance de l’essai :
11.1 Conseil scientifique
11.2 Centre de Méthodologie et de Gestion de l’essai
11.3 Comité de suivi et de validation des évènements
11.4 Comité indépendant de surveillance
12. Aspects statistiques
12.1 Nombre de sujets nécessaires
12.2 Méthodes statistiques prévues pour l’analyse
12.2.1 Généralités
12.2.2 Description de l’inclusion et du suivi
12.2.3 Caractéristiques des patients avant la mise sous traitent
12.2.4 Analyse du critère de jugement principal
12.2.5Analyse des critères de jugement secondaires
13 Considérations éthiques
13.1 Avis du CCPPRB
13.2 Amendements du protocole
13.3 Confidentialité des données
13.4 Assurance
14.Déviations du protocole
14.1 Arrêt de traitement, abandon de l’essai
14.2 Fermeture des centres, Violations de protocole
15 Publications des résultats
16Aspects Budgétaires, Surcoûts
17. Références
18. Annexes**:**

1. Calendrier de l’essai.

2. Caractéristiques des traitements à l’essai (Zophren, Cyclophosphamide CPM, méthyprednisolone MP).

3-4. Note d’information et formulaire de consentement.

5 à 10. Echelles d’évaluation des critères de jugement : EDSS, FSS, MSFC, SEP-59, MSIS, MSWS12.
11. Coûts pharmacie

12. Attestation d’assurance responsabilité civile

13. Déclaration d’Helsinki

**IV- RESUME DU PROTOCOLE :**

Justificatif : Le traitement actuel des formes secondairement progressives (SP) de sclérose en plaques (SEP) ne fait pas l’objet d’un consensus. Les médicaments utilisés actuellement peuvent présenter des indications restreintes ou des effets indésirables importants, ou leur efficacité n’a pas été démontrée de manière indiscutable. Le cyclophosphamide (CPM) a montré des résultats encourageants dans des études ouvertes mais il n’a pas encore été évalué de manière rigoureuse. Il s’agit d’un essai clinique randomisé multicentrique national de phase IIIb en double insu sur deux groupes parallèles.

L’objectif principal est de comparer l’efficacité du traitement par CPM par rapport à un traitement par méthylprednisolone (MP) administrés par voie IV pour empêcher l’aggravation confirmée sur 4 mois de 1 point d’EDSS (ou de 0,5 point si l’EDSS de départ est égale à 5,5 ou 6) à deux ans. L’étude concernera deux groupes randomisés parallèles. Le premier groupe recevra chaque mois pendant un an puis tous les deux mois pendant un an une dose de 750mg/m² de surface corporelle (SC) de CPM par voie intraveineuse (IV). Le second groupe recevra chaque mois pendant un an puis tous les deux mois pendant un an une dose de 1g de MP par voie IV.

Les objectifs secondaires sont de comparer dans les deux groupes de randomisation  la tolérance et la sécurité de ces traitements, l’effet des traitements sur des critères d’évaluation secondaire : score composite (MSFC) et les 3 z scores le composant, et le pourcentage de patients ayant progressé de 0,5 point ou 1 point de l’échelle EDSS (1 si l’EDSS initial est 4,0 ou 4,5 ou 0,5 point si l’EDSS initial est 5,0, 5,5 ou 6,0) et nombre de poussées. La période d’inclusion est prévue sur un an et la durée de l’essai est de 2 ans par patient (durée totale 3 ans).
Critères d’inclusion principaux : Homme ou Femme de 18 à 65 ans inclus présentant une forme secondairement progressive récente de SEP  avec une phase d’aggravation progressive du handicap d’au moins 6 mois et de moins de 3 ans avec une réduction du périmètre de marche et une aggravation du score EDSS d’au moins 0,5 point dans les 12 derniers mois non attribuable à des séquelles de poussées. Le score EDDS doit être situé entre 4,0 et 6,0 inclus.
Organisation

Dans chaque centre, chaque patient sera suivi par deux médecins investigateurs, un neurologue traitant (NT) chargé de la prise en charge médicale et neurologique du patient et un neurologue évaluateur (NE) chargé de l’évaluation neurologique des patients. Le calcul de la taille de l’échantillon a été réalisé sur la base d’une proportion attendue de patients sans aggravation à 2 ans de 75% dans le groupe CPM et de 60% dans le groupe MP.
Le calcul pour pouvoir comparer les délais d’aggravation observés dans chacun des groupes par un test du logrank de formulation bilatérale, avec un risque α = 5% et une puissance (1-β) = 80%, le nombre de sujets à inclure par groupe doit être au minimum de 155 patients. 180 patients par groupe seront inclus.

L’analyse statistique sera réalisée en intention de traiter. Pour les tests statistiques, le risque de première espèce α est fixé à 5%. Le critère de jugement principal sera analysé dans un premier temps à l’aide de la méthode de Kaplan-Meier et les délais de survenue seront comparés entre groupes de traitement par un test du logrank.
Dans un second temps, un modèle de Cox sera construit pour étudier l’effet des facteurs pronostiques.

**V- PROTOCOLE**

**1. JUSTIFICATION DE L’ETUDE :**

- 1. **SITUATION DU PROBLEME**
     1. **INTRODUCTION**La sclérose en plaques (SEP) est une affection de l'adulte jeune, invalidante et fréquente (prévalence en France estimée à 60 pour 100 000 habitants). Les plaques sont formées d'un infiltrat inflammatoire se développant autour de veinules et de zones de destruction de la myéline, avec une atteinte axonale associée (Brochet, 2001a). Sur le plan clinique, la maladie débute le plus souvent (85% des cas) par des poussées partiellement ou totalement régressives. Ces poussées correspondent à la survenue de nouvelles lésions inflammatoires du SNC. Dans la majorité des cas cette phase de poussées dite phase rémittente est suivie par une phase d’évolution progressive émaillée ou non de poussées surajoutées. La caractéristique majeure de cette phase secondairement progressive (SP) est l’existence d’une aggravation continue du handicap et, si il y a des poussées, cette aggravation doit persister en dehors des poussées (Lublin et Reingold, 1996). Le handicap présenté par les patients est vraisemblablement lié à des lésions myéliniques et axonales irréversibles. Ce handicap s'accumule avec le nombre de poussées et surtout du fait de cette progression secondaire et fait toute la sévérité de cette affection dans ses dimensions médicales, humaines, et socio-économiques. Compte tenu de la chronicité de la maladie et de l’âge moyen de début vers 30 ans et de la durée moyenne d’évolution (30 à 40 ans), l’impact de la maladie en terme socio-économique est considérable (Amato et al., 2002). La SEP génère des handicaps physiques et sociaux dont la lourdeur dépend de la forme clinique, du mode évolutif et du degré de sévérité de l’atteinte. Le handicap physique est générateur d’un handicap social et d’une incapacité de travail plus ou moins longue, source de pertes financières pour le malade et sa famille mais aussi pour la collectivité par le biais des différentes prestations, indemnités et allocations versées. Peu d’études ont été consacrées, à ce jour, au versant économique. Les études disponibles mettent tout d’abord l’accent sur le poids significatif des coûts indirects dans le coût total de la maladie (Amato et al., 2002) avec des répercussions sur le monde professionnel considérables. Touchant avec prédilection le sujet jeune, en pleine période d’activité professionnelle, la SEP est responsable d’incapacités et de mises en inactivité importantes (arrêts de travail, invalidités précoces). La SEP provoque également un retrait de la vie sociale et associative en raison du handicap qui confine progressivement les patients à leur domicile. L’incidence sur l’entourage n’en est que plus importante puisque l’état du patient peut conduire à la nécessité d’une aide pluri-quotidienne dont l’ampleur s’accroît avec le handicap. Dans le même temps, les études montrent la part prépondérante des coûts hospitaliers dans les coûts directs, ce qui constitue un schéma commun à l’Allemagne et la France. Enfin pour les dépenses médicales comme pour les coûts indirects, deux variables cliniques apparaissent comme hautement prédictives du coût de cette maladie : la forme clinique et l’importance du handicap. Il est certain que toute prise en charge permettant de diminuer le handicap peut permettre de diminuer le poids de cette maladie sur la société. Il est donc essentiel de prendre le handicap comme critère d’efficacité des traitements.
     2. **ETAT DES CONNAISSANCES SUR LES TRAITAMENTS ACTUELS DES FORMES SECONDAIREMENT PROGRESSIVES DE SCLEROSE EN PLAQUES**
        1. **Traitements par interféron:**Plusieurs traitements ont fait leur preuve dans le traitement de fond des formes rémittentes de SEP comme l’interféron bêta (Bétaféron ®, Avonex ® et Rebif ®) et l’acétate de glatiramère (copaxone ®) (Freedman et al., 2002, Noseworthy, 1999). Les résultats obtenus à la phase progressive pour ces produits sont moins évidents. Quatre études multicentriques ont été menées avec l’interféron bêta les formes SP. Deux études ont concerné le Bétaféron®. L'étude européenne (European study group, 1998) a étudié 718 patients avec un EDSS < 6,5 dont 358 patients ayant reçu du placebo et 362 de l’interféron. Le critère principal d'efficacité était le temps pour atteindre une progression confirmée du handicap c'est-à-dire d'un point de l’échelle Expanded Disability Status Scale (EDSS) dans la population en intention de traiter. Cette échelle ordinale dépend principalement de la marche à ce niveau de handicap. A 33 mois la différence entre les deux groupes était significative. A l’issue du suivi de 30 mois 49,8% des patients sous placebo avaient une aggravation d’un point d’EDSS contre 38,9% sous interféron. A noter que les différences ont été acquises rapidement : 13-15 mois après le début de l'étude 35% avaient progressé d'un point EDSS dans le groupe placebo contre 23% dans le groupe IFN. Après cette date les deux courbes sont très parallèles. Tout se passe comme si l'effet principal (action sur le processus inflammatoire?) survenait très tôt et qu'ensuite le traitement entretienne cet effet. Il faut en effet noter qu'une proportion élevée de patients avait eu des poussées dans les deux ans précédant l'inclusion (71,8 et 68,1% respectivement pour les groupes IFN et placebo). L'effet était cependant aussi important dans le groupe des patients n'ayant pas de poussées surajoutées que celui en ayant et quelque soit l'EDSS de départ. Les résultats en imagerie par résonance magnétique (IRM), positifs sur les lésions en T2 et T1 mais négatifs en ce qui concerne l'atrophie confirment que l'IFN agit surtout sur la constitution des lésions focales. Cet essai a conduit à l’obtention d’une AMM européenne pour le bétaféron® dans les formes SP.
           L'étude nord-américaine, non encore publiée (abstract Neurology, 2000 (54)p 2352), réalisée avec le même interféron (bétaféron ®) avec des critères d'inclusion et une méthodologie identique n'a pas confirmé ces résultats. Cette étude a inclus 939 patient ayant une SEP SP. Alors que les résultats IRM sur les lésions en T2 et T1 sont très positifs, il n'y a aucune différence en terme d'évolution d'EDSS entre les 2 groupes. Il a été proposé comme explication de la différence de résultats entre ces deux études l'existence de caractéristiques différentes entre les deux populations. Les patients de l'étude américaine avaient eu beaucoup moins de poussées dans la période précédant l'étude (56% sans poussée dans les 2 ans précédents) et avaient une phase progressive plus ancienne. Ce point est essentiel : la plupart des études thérapeutiques effectuées dans les formes SP montre une efficacité plus importante quand le début de la phase progressive est récent.
           L’étude IMPACT (Cohen et al., 2002) a étudié l’efficacité de l’interféron béta 1 a intramusculaire une fois par semaine à la dose de 60µg/semaine dans les formes de SEP secondairement progressive chez 436 sujets durant 2 ans versus placebo. Une efficacité significative a été retrouvée concernant l’évolutivité du handicap évalué par un score composite (Multiple Sclerosis Functional Composite, MSFC) qui était l’élément principal de jugement. Ce score qui combine les z scores d’un test de marche chronométré de 25 pieds (TW25), d’un test chronométré d’habileté manuelle (nine hole peg test, 9HPT) et d’un test d’attention (PASAT version 3s) est plus sensible au changement que l’EDSS mais sa pertinence clinique est discutée (Cutter, 1999 ; Cutter et al., 1999). Une efficacité sur les poussées surajoutées a été retrouvée ainsi que sur l’activité en IRM. Il n’a pas été retrouvé par contre d’efficacité sur l’ambulation ou l’EDSS (critère secondaire).
           Les résultats de l'étude SPECTRIMS (SPECTRIMS study group, 2001), qui a étudié le Rebif ® 22µg et 44µg chacun 3 fois par semaines en sous-cutané contre placebo chez 618 patients ayant une SEP SP durant 3 ans vont dans le même sens. Dans cette étude les résultats IRM sont très significatifs, et meilleurs avec le 44 que le 22, mais les résultats cliniques concernant la progression du handicap sont négatifs. Une efficacité sur la fréquence des poussées surajoutées a été en revanche retrouvée. Le pourcentage de patients ayant eu des poussées dans les 2 ans précédents était de 47%, donc très proche de l'étude précédente. Une analyse post-hoc a montré que ces patients ayant eu des poussées dans les deux ans précédents avaient par contre une diminution significative de la progression du handicap. Une nouvelle indication d’AMM a donc été délivrée pour l’interféron béta 1 a sous cutané 3 fois par semaine (Rebif 44 ®), restreinte aux formes de SEP SP avec poussées surajoutées. Il semble donc que si l'interféron peut avoir un effet positif dans les formes SP, cet effet a beaucoup plus de chance d'être observé si on traite ces patients le plus précocement possible et s’il persiste des arguments en faveurs du caractère inflammatoire de la maladie (persistance de poussées, évolutivité inflammatoire en IRM).
           Ces essais ont cependant permis de montrer plusieurs points importants :
            - Il existe une certaine sensibilité des formes SP aux traitements à visée immunologique (effet concordant en IRM dans les 4 essais) mais l’effet des immunomodulateurs reste modeste dans ces formes
            -Les outils d’analyse utilisés (EDSS) sont peu sensibles au changement à ce stade de la maladie mais la signification clinique des autres critères (score composite) est incertaine. Ces essais ont concerné principalement des patients ayant une forme peu évolutive (patients s’étant aggravés d’un point EDSS sur 2 ans avant l’étude dans la majorité des cas) chez lesquels la mise en évidence d’un effet thérapeutique est plus difficile.
           - L’effet est plu s net si la forme SP est plus récente ou associée à des poussées.
        2. **Mitoxantrone :**
           D’autres traitements ont été essayés. La mitoxantrone, un immunosuppresseur puissant, a montré des résultats positifs au cours d’un essai randomisé contre placebo (Hartung et al., 2002) confirmant l’impact possible des traitements immunosuppresseurs dans ces formes mais ce traitement pose des problèmes de toxicité à long terme qui empêche son utilisation prolongée. L’AMM récente accordée à ce produit (ELSEP) concerne les formes SP mais à condition que le patient ait une forme très évolutive (deux points minimum d’aggravation d’EDSS dans l’année précédente et IRM active).
        3. **Corticoïdes :**
           Une étude a comparé un traitement par de fortes doses de méthylprednisolone (MP) IV tous les deux mois pendant 2 ans à de faibles doses de corticoïdes chez 109 patients ayant une SEP SP (Kinkel 1999) . L'objectif mesuré était la proportion de sujets avec un échec thérapeutique (aggravation d'une des composantes d’un score composite pendant au moins 5 mois ou 3 poussées en 12 mois). Parmi les patients traités à forte dose, 38,9% ont atteint cet objectif contre 53,7% dans le groupe contrôle. Ce résultat n'était pas significatif mais l'analyse par courbe de survie a montré un effet modeste mais significatif du traitement.
        4. **Traitement par cyclophosphamide :**Le cyclophosphamide (CPM) est un agent alkylant qui est couramment utilisé dans le traitement de maladies auto-immunes comme le Lupus Erythémateux Disséminé (LED). Il a fait l’objet de différentes études dans la SEP sans qu’un consensus ne soit atteint quant à son efficacité.
           1. **Données d’efficacité :**
              Les premières études concernaient un protocole d’induction à fortes doses sur quelques jours qui ne s’est pas révélé adapté à une maladie inflammatoire chronique nécessitant un traitement prolongé. C’est pourquoi les résultats initiaux encourageants (Hauser et al., 1983) n’ont pas été confirmés par deux essais contrôlés (Likovsky, 1991 ; Canadian Cooperative Multiple Sclerosis Study Group, 1991). L’essai principal réalisé au Canada (Canadian Cooperative Multiple Sclerosis Study Group, 1991) a été pénalisé par l’existence d’un groupe contrôle très stable puisque seuls 29% des patients sous placebo se sont aggravés en 30 mois de suivi moyen.
              Depuis ces essais, des études ouvertes ont concerné l’utilisation de bolus mensuels à plus faibles doses selon les protocoles utilisés dans le LED dans les formes progressives de SEP (750mg/m² de surface corporelle par mois) (Weiner et al., 1993 ; Hohol et al., 1999 ; Zephir et al., 2004).
              L’étude de Weiner et al.,(1993) a ouvert la voie en traitant par bolus mensuels des patients ayant reçu le traitement à forte dose. L’effet rapporté était plus important chez les sujets plus jeunes et ayant une forme progressive récente. L’étude de Hohol (1999) a confirmé ces résultats avec des résultats encourageants, en particulier chez les patient ayant une durée de progression antérieure courte : 2,1 ans en moyennes et inférieurs à 5 ans en général. L’étude rétrospective française (Zéphir et al., 2004) portant sur 490 patients a récemment montré que ce traitement permettait une stabilisation ou amélioration de 78% des patients ayant une sep SP et 73,5% des formes progressives primaires à un an(Zéphir et al., 2004). Les patients ayant une amélioration de l’EDSS avaient une ancienneté de la phase SP de 5,1 ans en moyenne.
              Plusieurs études pilotes de phase II, ont concerné des formes rémittentes comme celle de Killian et al. (1988) qui portait sur seulement 14 patients. Des études non encore publiées mais présentée à l’American Academy of Neurology en 2003, ont montré l’intérêt des bolus de CPM pendant 6 mois dans les formes rémittentes ou SP s’aggravant très rapidement (Smith et al., Patti et al. ; Gauthier et al.). Smith et al. ont étudié 58 patients en phase rémittente RRMS en échec d’interféron (soit 2 poussées en un an, soit deux cures IV de corticoïdes en 1 an soit aggravation de 1,5 EDSS en 1 an) traités par 3 jours de méthylprednisolone (MP) IV puis randomisés ensuite entre 6 cures mensuelles de cyclophosphamide (CY) (800 mg/m2) plus MP ou MP seule. •Les patients ont poursuivi l’ IFN b-1a (30 mcg IM par semaine) pendant la phase de traitement IV de 6 mois puis les 18 mois de suivi. •Le critère primaire d’efficacité était le changement du nombre de lésions rehaussées par le gadolinium en IRM. Un mois après les 3 cures initiales de stéroïdes le nombre moyen de lésion rehaussées étaient 0,87 et étaient équilibrées entre les 2 groupes (p=0,71). •A 3 mois une augmentation moyenne de + 0,57 lésions rehaussées a été mesurée sous stéroïdes contre une diminution de 0,7 dans le groupe CPM (p=0,01). •A 6 mois l’augmentation était de + 0.19 en moyenne sous corticoïdes contre une diminution de 0.77 sous CPM (p=0,04). Pendant la phase IV seules 8 IRM sur 60 étaient « actives » sous CPM contre 21 sur 54 sous MP. Le nombre moyen de lésions rehaussées était 1,18 sous MP contre 0,2 sous CPM (p=0.001). 6 mois après l'arrêt du traitement IV une augmentation de 0,58 lésions Gd + était observée dans le bras MP contre une diminution de 0,53 sous CPM (p=0.02).
              Patti et al ont étudié cliniquement et en IRM pendant 36 mois après un traitement de 18 mois combinant l’interféron beta et le CPM dans un groupe de 10 patients ayant une SEP rémittente très active et en échec d’IFNB . Après les 18mois de combinaison ils ont poursuivis l’IFN. Le taux de poussées très abaissé s’est maintenu pendant le suivi (0.13) et l’EDSS est resté stable (2.35 en moyenne), de même que le nombre de lésions en T2 alors qu’aucune nouvelle lésion rehaussée par le gadolinium n’était détectée sur des IRM annuelles.
              Gauthier ont •analysé 47 patients ayant une SEP rémittente traités par CPM IV et MP en secours après échec des autres traitements (interféron ou acétate de glatiramère) à cause d’une progression clinique (EDSS) [n=21] ou de changements sur l’IRM [n=24] ou une combinaison entre les deux. Les patients avaient un EDSS de 3.2 et une durée de maladie de 6.53 ans en moyenne. 80 % avaient été traités avec l'interféron-beta et 20 % avec copaxone ®. Le traitement (mensuel / 6 mois) a interrompu la progression de la maladie dans 78 % des cas (EDSS) et a stabilisé l'activité IRM dans 75 %.
           2. **Données de tolérance :**
              Ces différentes études ont montré une tolérance acceptable de ce traitement au cours de la SEP aux doses utilisées. Dans l’étude rétrospective française seuls 4,5% des patients ont présenté un événement indésirable sévère (nausées et vomissements, leucopénie, infections, alopécie réversible et hépatites). Seul un patient a du interrompre ce traitement du fait de ces événements indésirables (hépatite). Les doses utilisées (750 mg/m² de surface corporelle (SC)) permettent d’éviter d’atteindre les doses cumulées à risque onco-hématologique.
              Un risque de cancer de la vessie a été rapporté chez les patients ayant une SEP et ayant une sonde urinaire à demeure (ce qui constituera un critère de non inclusion) et ayant reçu des doses cumulées de plus de 50 grammes de cyclophosphamide ce qui est très supérieur aux doses proposées (De Ridder et al., 1998).
              Le mode d’action supposé du CPM utilisé à ces doses dans la SEP ne serait pas lié à une immunosuppression mais à un effet immunomodulateur : augmentation des lymphocytes de type Th2, sécréteurs d’interleukine 4 et porteurs du récepteur aux chémokines CCR4 (Comabella et al., 1998, Karni et al., 2004).
              Un essai contrôlé du CPM en bolus mensuels intraveineux (IV) est nécessaire pour établir son efficacité dans les formes SP récentes.
           3. **Retombées attendues de l’étude :**
              Le traitement actuel des formes SP de la SEP ne fait pas l’objet d’un consensus. Les médicaments utilisés actuellement peuvent présenter des indications restreintes ou des effets indésirables importants, ou leur efficacité n’a pas été démontrée de manière indiscutable. Le cyclophosphamide semble donner des résultats encourageants dans le traitement des formes SP de SEP, mais il n’a pas encore été évalué de manière rigoureuse. L’essai clinique randomisé en double insu qui fait l’objet de ce protocole permettra d’évaluer l’efficacité et la tolérance du cyclophosphamide dans les formes SP de la SEP. Si les résultats en sont positifs, une nouvelle option thérapeutique sera disponible pour ralentir l’aggravation du handicap neurologique chez les patients concernés.
  2. **Description des traitements à l’essai :**
     1. **Traitements à l’essai :**
        L’étude concernera deux groupes randomisés parallèles. Le premier groupe recevra chaque mois pendant un an puis tous les deux mois pendant un an une dose de 750mg/m² de surface corporelle (SC) de cyclophosphalmide (endoxan ®) par voie intraveineuse (IV). Le second groupe recevra chaque mois pendant un an puis tous les deux mois pendant un an une dose de 1g de méthilprednisolone (solumédrol ®) par voie IV.
     2. **Intérêt par rapport aux traitements disponibles :**Il n’existe pas de traitement de référence des formes SP de SEP. Comme nous l’avons vu trois médicaments disposent d’une AMM dans cette indication mais ne peuvent pas être considérés comme traitement de référence :
        1. **L’interféron bêta :**
           Le bétaféron ® (interféron bêta 1b) dispose d’une AMM pour les formes SP et le rebif 44 ® (interféron bêta 1a) dispose d’une AMM pour les formes SP avec poussées surajoutées. A l’heure actuelle la grande majorité des patients débutant une forme SP de SEP a été traitée par interféron bêta pour la phase rémittente. Le début de la phase SP signifie chez ces patients un échec du traitement de fond par interféron et ils ne relèvent donc pas, dans ce cas, de la poursuite d’un tel traitement. L’intérêt du traitement à l’essai est de stabiliser la maladie de ces patients ne répondant pas aux interférons. Les études pilotes décrites plus haut laissent augurer d’une efficacité chez les patients en échec d’interféron. Les patients débutant une phase SP, réunissant les critères d’inclusion du protocole et n’ayant pas eu d’interféron et ne présentant pas de contre-indications à ce traitement auront le choix de participer à l’étude ou de choisir de recourir au traitement par interféron. Les patients seront informés dans la notice d’information de la disponibilité du traitement par interféron et des résultats des études le concernant dans cette forme, en particulier l’absence d’effet sur le handicap mesuré par EDSS dans 3 des 4 études réalisées. S’ils font le choix de l’interféron et que ce traitement échoue ils pourront être inclus dans le protocole, s’ils le souhaitent, qu’ils réunissent toujours les critères d’inclusion et que la période d’inclusion ne soit pas terminée.
        2. **Mitoxantrone :**
           L’AMM concernant ce traitement ne concerne que les patients ayant eu une aggravation d’au moins deux points d’EDSS dans l’année précédente et la présence de lésions prenant le contraste sur l’IRM. Il s’agit donc de patients ayant une forme agressive de SEP qui ne relèvent pas de ce protocole.
        3. **Corticoïdes :**
           Voir plus loin choix du comparateur.
  3. **Hypothèse testée :**
     L’hypothèse testée dans l’étude est que le traitement par cyclophosphamide administré par voie IV en bolus mensuels pendant un an puis bimestriels pendant un an est supérieur à la méthylprednisolone (MP)administrée par voie IV au même rythme pour empêcher l’aggravation confirmée sur 4 mois de 1 point d’EDSS (ou de 0,5 point si l’EDSS de départ est égale à 5,5 ou 6) à deux ans.
     Il s’agit d’un essai de supériorité.
  4. **Choix méthodologiques :**L’objectif général de l’étude est d’évaluer l’efficacité (en termes de stabilisation) et la tolérance du cyclophosphamide (CPM) dans le traitement des formes progressives secondaires récentes de sclérose en plaques. Le schéma le plus adapté est celui d’un essai clinique randomisé en double insu.
     1. **Traitement évalué :**
        1. **Choix :**
           Les études préliminaires, non comparatives, du CPM dans les formes progressives de SEP ont montré des résultats encourageants, notamment lors de l’administration du produit en bolus mensuels (Cf. Chapitre 1.1.2.3.). Cependant, seul un essai clinique randomisé permettra d’évaluer de manière valide son efficacité.
        2. **Choix de la dose :**
           Le mode d’administration (bolus par voie IV) et la dose (750mg/m² de surface corporelle par bolus) sont ceux qui ont fait l’objet des études ouvertes (Hohol et al., 1999; Zephir et al., 2004).
     2. **Choix du Traitement de comparaison :**
        1. **Existe-il un traitement de référence des formes SP :**Bien que trois médicaments possèdent l’AMM en France dans cette indication (Interférons Bêta et mitoxantrone), il n’existe pas à l’heure actuelle de traitement de référence indiscutable pour les patients présentant des formes SP de SEP. En effet, le Betaferon® et le Rebif ® sont indiqués dans les formes rémittentes et la plupart des patients en bénéficient actuellement. Ainsi, le passage à une forme SP pour ces patients correspond à un échec thérapeutique qui ne justifie pas la poursuite de ce traitement. Le Rebif® possède l’AMM seulement pour les formes SP avec poussées surajoutées. Les résultats montrant une efficacité de ce médicament ont été obtenus, au cours d’un essai randomisé contre placebo, seulement dans le sous-groupe de patients ayant présenté des poussées et en regroupant les deux doses de Rebif étudiées, lors d’une analyse post hoc (SPECTRIMS study group, 2001). Les résultats étaient statistiquement significatifs sur le pourcentage de patients ayant une progression du handicap à 3 ans, mais seule une tendance était retrouvée pour le délai de progression du handicap. Ainsi, l’efficacité de ce produit n’est pas validée de manière indiscutable, même dans le sous-groupe de patients pour lesquels le traitement possède l’AMM.
           Enfin, l’Elsep® (mitoxantrone) possède une AMM seulement pour les formes SP très évolutives et présente une toxicité hématologique importante et une cardiotoxicité qui limitent son utilisation.
        2. **Choix de la méthylprednisolone comme traitement de comparaison :**
           L’absence de traitement de référence indiscutable des formes SP de SEP pourrait faire discuter la comparaison du traitement évalué à un placebo. Cependant, l’aggravation observée au cours de la phase SP de la SEP étant irréversible, il est difficilement envisageable de laisser des patients ayant une forme SP de SEP récente et évolutive sous placebo. Plusieurs études du CPM et de la mitoxantrone (Edan et al., 1997, Smith et al., 2003, AAN) ont utilisé la méthylprednisolone en bolus mensuels IV comme comparateur. L’étude décrite plus haut (Kinkel, 1999) n’a pas montré d’effet significatif par rapport au placebo mais l'analyse par courbe de survie a montré un effet modeste mais significatif du traitement. Le recours à ce comparateur peut risquer de diminuer la puissance de l’étude en diminuant la différence entre les groupes comparés mais ce risque apparaît modéré et il devrait faciliter l’acceptation du protocole et donc les inclusions
        3. **Mode d’administration et choix des doses :**
           La méthylprednisolone (Solumédrol) sera administrée à raison de 1 g par voie IV, une fois par mois pendant 1 an (selon le protocole de l’étude de Kinkel puis une fois tous les deux mois pendant un an pour suivre le même rythme que l’endoxan ®.
     3. **Méthodes de mise en œuvre du double-insu :**Les essais thérapeutiques dans la SEP font systématiquement appel à deux investigateurs, l’un chargé de l’évaluation des critères de jugement (neurologue évaluateur NE) et l’un chargé de l’évaluation des événements indésirables et de la prise en charge du patient (neurologue traitant NT).
        1. **- Problème des anti-émétiques :**Un problème spécifique posé par cette étude est la nécessité de prévenir les troubles digestifs (nausées, vomissements) dus au cyclophosphamide. Les protocoles habituels utilisent les médicaments anti-émétiques type anti-sérotonine (Zophren ®). Il est indispensable que les patients sous cyclophosphamide reçoivent un traitement de ce type pour limiter les événements indésirables, pour le confort des patients et pour maintenir au maximum le double-insu pour le neurologue traitant. En revanche il ne serait pas justifié d’administrer ce produit aux patients sous MP. De même à la fin du traitement intraveineux, du zophren® per os est habituellement prescrit pendant quelques jours. Pour régler ce problème la stratégie adoptée sera la fourniture par la pharmacie du zophren ® injectable ou de serum glucosé isotonique dans des seringues ne permettant pas d’identifier le produit par l’investigateur et les infirmières et la prescription d’une ordonnance pour tous les patients à la sortie de zophren ® per os « si besoin » qui y recourront en cas de nausées ou de vomissements.
        2. - **Perfusion de rinçage :**
           Tous les patients, quelque soit le groupe recevront la perfusion de rinçage de sérum glucosé dont le but est d’éviter les effets irritants vésicaux du cyclophosphamide. La durée de cette perfusion sera identique pour tous les patients, soit 8 heures afin de permettre la réalisation du traitement en hospitalisation de jour pour les deux groupes.
        3. **- Suivi biologique :**
           Le CPM induit fréquemment une lymphopénie alors que le MP induit plus fréquemment une polynucléose neutrophile. Cependant cette dernière est très transitoire et ne devrait pas persister sur le bilan réalisé avant l’hospitalisation suivante. Pour éviter que ces constatations biologiques ne perturbent l’insu les bilans biologiques seront transmis uniquement au centre coordonnateur qui ne les transmettra au neurologue traitant qu’en cas de nécessité médicale (suspicion d’infection par exemple). C’est le centre coordonnateur qui transmettra aux pharmaciens les consignes pour adaptation de dose.
     4. **Choix de la population étudiée :**
        La population étudiée est représentée par les patients atteints d’une formes SP de SEP pour lesquels il n’existe pas de traitement de référence efficace sur le handicap. Les données des études ouvertes du cyclophosphamide indiquant que ce traitement est principalement efficace si le début de la phase progressive est récent il a été choisi de sélectionner des patients dont la phase progressive a moins de 3 ans. Afin de mettre en évidence un effet clinique la population sélectionnée doit présenter des signes objectifs d’aggravation qui sont définis par la réduction du périmètre de marche critère majeur de mesure de la progression. Cette aggravation minimale est de 0,5 point d’EDSS sur les 12 derniers mois à cause d’une réduction du périmètre de marche. Afin de mesurer l’effet du traitement sur le périmètre de marche les patients doivent présenter une limitation de ce périmètre mais être capables de marcher, ce qui correspond aux bornes des critères d’éligibilité de 4 à 6 points d’EDSS.
     5. **Choix du Critère de jugement principal :**
        Le handicap conditionne la qualité de vie des patients atteints de SEP, le pronostic de leur maladie et les coûts médico-économiques attribuables à cette maladie. La mesure la plus utilisée du handicap dans la SEP est l’échelle « expanded disability status scale » (EDSS) (Kurtzke et al., 1983). C’est pourquoi le délai d’aggravation du score EDSS a été choisi. Une des critiques concernant cette échelle concerne sa variabilité inter-observateur (Brochet, 2001b). La variabilité est moindre dans le segment 4-6 de l’échelle dont la cotation dépend essentiellement des mesures de marche. Elle peut à ce stade de la maladie être due à une évaluation du périmètre de marche ne reposant que sur l’interrogatoire et sur des divergences d’interprétation de l’échelle en fonction du périmètre de marche. Afin d’éviter ces problèmes il sera demandé une mesure objective du périmètre de marche et la transposition en cotation EDSS sera effectuée par les experts du centre coordonnateur à partir de ces données brutes de mesure du périmètre de marche.
     6. **Choix des critères secondaires :**Le score composite de la sclérose en plaques (Multiple Sclerosis Functional Score, MSFC) a été développé récemment pour proposer un score plus sensible. Il représente la moyenne des z scores d’une mesure de marche chronométrée, d’un test d’habileté manuelle et d’un test d’attention auditive. Ce score a été utilisé dans les essais thérapeutiques récents sur la SEP et constitue le critère principal de l’étude IMPACT de l’avonex ® dans les formes SP de SEP. Afin de permettre la comparaison avec ces études ce score sera utilisé comme critère secondaire ainsi que les sous-scores le composant.
        Le nombre de poussées sera pris en compte pour comparaison avec les essais existant.
        Il sera également fait appel à titre de critères exploratoires à des autoquestionnaires de qualité de vie et de handicap. L’échelle SEP-59 (Vernay et al., 2000) est une échelle de qualité de vie validée dans la SEP en français. L’échelle MSIS est une échelle brève de handicap récemment traduite (Roullet, communication personnelle).

1. **OBJECTIFS**
   1. **L’objectif principal** de cette étude est de comparer l’efficacité d’un traitement par cyclophosphamide (CPM) en bolus IV à 750 mg/m² SC répétés mensuellement pendant un an puis tous les deux mois pendant un an par rapport à la méthylprednisolone (MP) en bolus IV à la dose de1 g délivrés au même rythme, sur le délai d’aggravation du handicap neurologique mesuré par l’échelle EDSS (plus d’1 point si l’EDSS de base est 4 ou 4,5 et plus de 0,5 point si l’EDSS de base est 5, 5,5 ou 6) à deux ans et confirmé après 4 mois (ou 2 mois si elle survient à la dernière administration de traitement).
   2. **Les objectifs secondaires** sont de comparer dans les deux groupes de randomisation :
      - la proportion de patients ayant progressé de 0,5 point ou 1 point de l’échelle EDSS (1 si l’EDSS initial est 4 ou 4,5 ou 0,5 point si l’EDSS initial est 5, 5,5 ou 6) à 2 ans (visite de fin de la période programmée de traitement), 
      - l’effet des traitements sur des critères d’évaluation secondaire : score composite (MSFC) et les 3 z scores le composant,

**-** le nombre de poussées,

- la tolérance aux traitements.

1. **METHODES GENERALES**
   1. **Schéma d’étude :**
      Il s’agit d’un essai clinique randomisé multicentrique national de phase IIIb en double insu sur deux groupes parallèles.
      La période d’inclusion est prévue sur un an et la durée de l’essai est de 2 ans par patient (durée totale 3 ans).
   2. **Randomisation :**

La liste de randomisation est établie par le statisticien de l’Unité de soutien méthodologique du CHU de Bordeaux (USMR) avant le début de l’essai. La randomisation est stratifiée sur le centre. Il n’est pas prévu de stratification sur les facteurs pronostiques éventuels, comme l’existence de poussées dans l’année précédant la randomisation ou la présence de lésions prenant le gadolinium. Les effectifs des deux groupes de traitement sont équilibrés. Un document complet décrivant la procédure de randomisation est conservé de manière confidentielle à l’USMR.

La randomisation des patients est effectuée de façon centralisée par le chef de projet du Centre de Méthodologie et de Gestion de l’étude via le site Internet de l’USMR. : http:/usmr.isped.u-bordeaux2.fr. Ce site permet de vérifier l’éligibilité des patients. Lorsqu’un investigateur souhaite effectuer la randomisation d’un patient et qu’il dispose de tous les résultats nécessaires à l’inclusion du patient dans l’essai, il remplit la demande de randomisation figurant dans le classeur d’observation et l’envoie par fax au chef de projet du Centre de Méthodologie et de Gestion. Celui-ci contrôle la conformité des données aux critères d’inclusion, puis remplit l’écran « demande de randomisation » et valide le contenu de l’écran. Si les données se révèlent conformes aux critères d’éligibilité, l’inclusion est effectuée. Le chef de projet prévient le pharmacien qu’une randomisation va lui être communiquée. Le pharmacien se connecte sur le site Internet pour connaître le groupe de traitement alloué au patient. Il conserve de manière confidentielle le groupe de traitement attribué à chaque patient

Le traitement débutera au mieux dans les 7 jours après la demande de randomisation et au maximum 15 jours après. Ce délai peut être prolongé en cas de survenue d’anomalie clinique ou biologique n’autorisant pas le début du traitement avec accord du centre de coordination.

1. **CRITERES D’ELIGIBILITE :**
   1. **Sélection des centres investigateurs :**
      Ce protocole d’essai clinique a été proposé et discuté aux centres spécialisés dans le prise en charge de la SEP qui participaient à la réunion annuelle du club francophone de la SEP (CFSEP). Un certain nombre de centres ont alors manifesté leur désir de participation. Lors de la rédaction du protocole un comité scientifique a été constitué comportant des représentants de la grande majorité des centres de référence dans la prise en charge de la SEP (et dépassant les centres précédents) et le protocole a été discuté par voie électronique entre ces experts. Les centres sélectionnés pour l’étude représentent d’une part un grand nombre de services de neurologie de CHU ayant développés une expertise dans le domaine de la SEP et ayant dans leur très grande majorité participé à des essais cliniques dans le domaine et pour quelques uns de service de neurologie d’hôpitaux généraux ayant développés une expertise dans le domaine de la SEP et participant au réseau de prise en charge de la SEP en Aquitaine (AQUISEP).
   2. **Critères d’inclusion**:
      Tous les critères suivants doivent être remplis par les patients pour être éligibles pour participer à l’étude :
      - Homme ou Femme de 18 à 65 ans inclus ;
      - Présentant une forme secondairement progressive récente de SEP :
       - SEP certaine selon les critères de Mc Donald et al. (2001) ;
       - Forme secondairement progressive de SEP selon la définition de Lublin et Reingold (1996).
       - Les patients doivent avoir présenté une phase rémittente comportant au moins deux poussées identifiées et une phase d’aggravation progressive du handicap d’au moins 6 mois et de moins de 3 ans;
       - L’aggravation du handicap récente doit être responsable d’une aggravation du score EDSS d’au moins 0,5 point dans les 12 derniers mois non attribuable à des séquelles de poussées ;
       - Les patients doivent avoir présenté une réduction du périmètre de marche dans l’année écoulée;
      - EDDS entre 4,0 et 6,0 inclus ;
      - Les patients doivent :
       - avoir signé un consentement éclairé après information et avant toute procédure nécessitée par le protocole;
       - accepter de recourir à une contraception efficace durant toute la durée de l’étude s’ils sont en âge de procréer ( préservatif ou contraception efficace du patient ou du partenaire).
   3. **Critères de non inclusion**Les patients ne peuvent être recrutés pour l’étude s’ils remplissent l’un des critères suivants :
      - Autres formes cliniques de SEP :
       - Phase SP ayant débuté depuis plus de 3 ans ;
       - Forme rémittente pure de SEP sans progression entre les poussées ;
       - Forme progressive primaire de SEP ;
       - Forme transitionnelle progressive de SEP c'est-à-dire ayant eu une seule poussée avant la phase progressive (Gayou et al., 1997) ;
      - Pathologies associées :
       -Présence de toute autre maladie autre que la SEP pouvant contribuer aux symptômes et signes neurologiques ou en affecter l’évaluation ;
       Maladie organique ou psychiatrique compromettant la capacité du patient a comprendre les informations données et à donner ou retirer son consentement en toute connaissance des informations nécessaires et sa capacité à suivre le protocole ;
       - Abus d’alcool ou de stupéfiants actuel ou dans les 2 ans précédents;
      - Contre-indication aux traitements à l’essai :
       - Cystite hémorragique préexistante.
       - Allergie connue au cyclophosphamide, aux corticoïdes et en particulier au Solumédrol ®;
       - Maladie infectieuse en cours ;
       - Patients porteurs d’une cathétérisation permanente de la vessie (sondage urinaire à demeure) ;
       - Grossesse et allaitement. ;
       - Insuffisance médullaire sévère (Hb <9g/dl  ou Leucocytes <3000/mm3  ou Lymphocytes < 900 /mm3  ou Plaquettes < 125 000/mm3). Ce critère devra être réévalué après le consentement, pendant la période de screening au vu du bilan biologique;
       - Etat psychotique ;
       - Ulcère gastrique ou duodénal en évolution ;
       - Diverticulose colique ;
       - Antécédents de troubles du rythme cardiaque après injection de MP IV ;
       - Atteinte hépatique sévère (ASAT > 3X limite supérieure de la normale) ou rénale sévère (Créatinine > 180 µmol/L) ;
       Diabète ;
      - Participation en cours ou dans les 3 mois précédents à un autre essai thérapeutique ;
      - Traitement antérieur par CPM, irradiation totale lymphoïde, anticorps monoclonal anti-CD4 ou anti-CD52 ou anti-VLA 4, mitoxantrone, cladribine ou cyclosporine A.
      - En cas de traitement antérieur par interféron bêta, ou méthotrexate un intervalle d’au moins un mois doit être respecté entre l’arrêt de ce traitement et le début du traitement à l’étude. Pour les statines voir chapitre 6.
      - En cas de traitement antérieur par corticoïdes intraveineux mensuels, ce traitement doit avoir été arrêté depuis au moins 1 an.
   4. **Critères d’exclusion :**Voir chapitre 14.
2. **Traitements de l’essai**:
   1. **Traitements**:
      Les solutés de perfusions des produits testés seront préparés à la pharmacie hospitalière. Le pharmacien sera informé par le centre de méthodologie et de gestion de l’essai du groupe dans lequel le patient a été randomisé.
      Les traitements préparés par la pharmacie seront étiquetés conformément à la réglementation selon la Procédure opératoire standard de l’USMR et de manière à maintenir l'insu.
      1. **Groupe Cyclophosphamide (CPM)**:
         Ce groupe recevra du Cyclophosphamide (DCI) anhydre (flacon de 1g de poudre pour préparation injectable).
         La posologie sera adaptée par le pharmacien selon les informations communiquées par le centre de méthodologie et de gestion de l’essai en fonction des résultats de la NFS.
         La posologie est de 750 mg/m² de surface corporelle si les lymphocytes sont > 1400 ; 500 mg/m² si les lymphocytes sont entre 1000 et 1400 et 400 mg/m² entre 900 et 1000.
         Afin de préparer une solution isotonique de CPM prête à l'emploi, la poudre doit être dissoute dans une solution de sérum glucosé à 5%. La solution reconstituée ne doit pas être conservée au-delà de 48 heures et ne doit pas dépasser une concentration de 2 %.
         La voie d'administration est la voie veineuse en perfusion courte (3 heures).Le médicament préalablement reconstitué dans une solution de sérum glucosé à 5 % est introduit dans le liquide de perfusion (soluté injectable isotonique de glucose) par le pharmacien.
         Une fois le traitement reçu de la pharmacie, l’infirmière doit mettre en place une voie veineuse (2,5 litres de G5 sur 8 heures), faire l’injection de traitement anti-émétique (voir plus loin) puis, après 30 minutes, mettre en Y la perfusion de traitement à l’étude pendant 3heures .
         Le schéma thérapeutique est le suivant : administration de CPM toutes les 4 semaines pendant 48 semaines (12 cycles) puis toutes les 8 semaines pendant 48 semaines
      2. **Groupe Méthylprednisolone (MP)**:
         Les patients du groupe MP recevront  un lyophylisat de méthylprednisolone (DCI) hemisuccinate (flacon de 500mg). La dose de MP sera de 1g dilué dans une solution injectable isotonique de glucose administré en 3 heures. Les solutés seront préparés à la pharmacie. Les procédures d’administration seront identiques à celles du groupe CPM.
         Une fois le traitement reçu de la pharmacie, l’infirmière doit mettre en place une voie veineuse (2,5 l G5 sur 8 heures), faire l’injection de traitement anti-émétique (voir plus loin) puis mettre en Y la perfusion de traitement à l’étude pendant 3heures. Le schéma thérapeutique est le suivant : administration de MP toutes les 4 semaines pendant 48 semaines (12 cycles) puis toutes les 8 semaines pendant 48 semaines.
   2. **Déroulement des traitements**:
      Les administrations se dérouleront au cours d’hospitalisations de jour.
      Trois jours ouvrables avant son admission le patient doit effectuer un bilan sanguin qui lui aura été prescrit par le neurologue traitant à la visite précédente  et comprenant une numération formule sanguine (NFS), plaquettes, vitesse de sédimentation, un ionogramme sanguin, le dosage de l’urée , de la créatinine et des transaminases.
      Le laboratoire devra faxer le résultat au centre de méthodologie et de gestion de l’essai.
      Si le taux d'Hb <9g/dl  ou le nombre de leucocytes est <3000/mm3  ou de lymphocytes < 900 /mm3  ou de plaquettes < 125 000/mm3 le centre informe le neurologue traitant qu’il doit: envoyer au patient une ordonnance pour une nouveau bilan (NFS) une semaine plus tard et reporter l’hospitalisation d’une semaine.
      Si il existe une augmentation des polynucléaires neutrophiles (> 2 fois la limite supérieure de la normale) et/ou une VS élevée (> 2X normale à la première heure) le centre communique les éléments pertinents du bilan au neurologue traitant qui doit contacter le patient afin de déterminer s’il présente des signes infectieux (fièvre, brûlures mictionnelles, douleurs lombaires ou autre). Une visite (consultation) non programmée peut être décidée et un ECBU réalisé afin de décider de reporter l’hospitalisation.
      A l’arrivée, il faut peser le patient, prendre la TA et la température, réaliser un ECG, et faire un examen des bandelettes urinaires : Si les bandelettes sont positives  il faut prélever un ECBU.
      En cas de bandelettes urinaires positives ou d’ECBU positif et en l’absence de signe clinique d’infection urinaire (fièvre, signes locaux) et biologiques d’infection le traitement pourra être effectué sous les conditions suivantes : débuter traitement par un antiseptique ou antibiotique, par exemple, noroxine ® (norfloxacine) 400 mg matin et soir pour 5 jours (en respectant contre-indications et règles d’associations médicamenteuses). La procédure pour le traitement anti-émétique est décrite au chapitre 6.
3. **Traitements associés**:
   1. **Traitements antiémétiques**:
      Les patients du groupe CPM doivent recevoir un traitement antiémétique :
      8mg de Zophren ® en IV lente dans la tubulure 30 minutes avant la mise en place du soluté de perfusion du traitement à l’étude suivi d’une administration de 8mg en IV lente dans la perfusion 4 heures et 8 heures après la première injection. Afin de préserver l’insu il sera organisé la fourniture par la pharmacie du zophren ® injectable ou de serum glucosé isotonique dans des seringues ne permettant pas d’identifier le produit par le neurologue traitant et les infirmières et étiquetés avec le numéro d’identification du patient.
      Les patients du groupe CPM devant également recevoir des antiémétiques per os pendant quelques jours après la perfusion une prescription sera remise à tous les patients de zophren ® per os « si besoin » afin que ceux-ci puissent y recourir en cas de nausées ou de vomissements à la dose de 8mg toutes les 12 heures jusqu’à cinq jours.
   2. .**Traitements interdits**:
      Sont contre-indiqués pendant toute la durée de l’essai le recours à une irradiation totale lymphoïde, aux anticorps monoclonaux anti-CD4, anti-CD52 ou anti-VLA 4, à la mitoxantrone, à la cladribine ou la cyclosporine A, et au vaccin contre la fièvre jaune, les autres vaccins vivants atténués (sauf fièvre jaune), et d’une façon générale tout agent immunosuppresseur ou immunomodulateur (dont azathioprine, methotrexate, interférons, acétate de glatiramère, pentoxyphylinne). Le recours à tout autre traitement en cours d’évaluation clinique est également interdit.
   3. **Traitements déconseillés**:
      Plusieurs études expérimentales et une étude clinique suggère un effet possible des statines dans la SEP. Les patients sous statine à dose fixe depuis au moins 12 mois et réunissant cependant les critères d’éligibilité dans l’étude pourront être inclus dans l’étude. Si au cours de l’étude l’état de santé d’un patient nécessite la mise sous statine le neurologue traitant devra préférer si possible la prescription d’une autre classe d’hypolipémiant. Si l’indication est formelle et que l’état morbide justifiant cette prescription ne constitue pas un critère d’exclusion de l’étude (par exemple accident vasculaire cérébral invalidant) le patient pourra être maintenu dans l’étude.
   4. **Prise en charge des poussées**:
      Les patients recevront des consignes afin de contacter le centre en cas de suspicion de poussée dès que possible. Le neurologue traitant devra alors demander au patient de venir au centre pour une visite non programmée comprenant une consultation avec lui et un examen neurologique avec le neurologue évaluateur au plus tard 9 jours après l’appel du patient.
      La définition des poussées retenue dans l’étude est l’apparition de nouveaux signes neurologiques ou l’aggravation de signes neurologiques présents de façon aiguë pendant au moins 48 heures et responsable d’une augmentation de 0,5 point d’EDSS au moins OU d’un point d’un score fonctionnel (FSS) de Kurtzke. Afin de distinguer les poussées d’une aggravation progressive plus rapide il sera requis pour retenir une poussée que l’aggravation doit s’être constituée en moins d’un mois.
      En cas de survenue d’une poussée le neurologue traitant peut décider la mise en route d’un traitement. Le protocole conseillé (Brochet, 2001c) est : Méthylprednisolone 1 gramme dans un soluté de glucosé à 5% sur 3 heures pendant 5 jours sans relais oral. Les hospitalisations pour poussée doivent être consignées comme événement indésirable mais ne constituent pas un événement indésirable grave.
4. **Critères de jugement**:
   1. **Critère de jugement principal**:
      Le critère de jugement principal est le délai d’aggravation du score EDSS (Kurtzke, 1983)( annexe 5,6). Cette aggravation est définie par une augmentation de 0,5 point de l’échelle EDSS si l’EDSS initial est 5, 5,5 ou 6ou 1 point si l’EDSS initial est 4,0 ou 4,5. et à condition que cette aggravation soit confirmée lors des évaluations effectuées quatre mois après la première visite montrant cette aggravation. Si l’aggravation est constatée lors de la dernière visite de la période de traitement la confirmation à deux mois, lors de la visite de fin d’étude, sera suffisante. Le délai d’aggravation sera calculé par la différence entre la date de la première visite avec cette aggravation et la date d’initiation du traitement.
      Afin de diminuer le risque de variations inter observateurs dans les cotations de l’EDSS il sera demandé aux neurologues évaluateurs de réaliser un examen neurologique type qui leur permettra de coter les échelles fonctionnelles FSS de Kurtzke qui servent à établir l’EDSS dans les premiers niveaux de l’échelle et de mesurer le périmètre de marche sur 500 mètres (annexe 5). Le score EDSS sera établi au niveau du centre coordonnateur à partir de ces données sources.
   2. **Critères de jugement secondaire**:
      - Proportion de patients ayant progressé de 0,5 point ou 1 point de l’échelle EDSS (1 si l’EDSS initial est 4 ou 4,5 ou 0,5 point si l’EDSS initial est 5, 5,5 ou 6) à 2 ans (visite de fin de la période programmée de traitement). 
      - Score composite de la SEP (Multiple Sclerosis composite score : MSFC) à deux ans (Cutter, 1999) (annexe 7). Le MSFC comprend la combinaison de 3 mesures, une mesure chronométrée d’habileté motrice du membre supérieur, le nine hole peg test (9HPT), une mesure de marche : temps de marche (T.M.) chronométré sur 8 m, et un test cognitif mesurant l’attention, le PASAT (version 3 secondes). Le score MSFC est établi à partir des 3 z scores de ces 3 mesures selon la formule : (Z 9HPT – Z TM + Z PASAT)/3.
      - Z scores du 9 HPT, z score du TM et z score du PASAT 3s à deux ans.
      - Nombre de poussées pendant l’étude.
      – Tolérance au traitement : proportion et délai de survenue des événements indésirables graves et non graves, évalués à la fin de l'étude.
   3. **Critères d’évaluation exploratoires :**Ces critères ne seront pas considérés comme des critères d’efficacité :
      - Autoquestionnaire de qualité de vie (échelle SEP 59, Vernay et al.,2000 ) à un an et à la visite de fin d’étude permettant d’établir 15 scores correspondant aux axes de l’échelle.
      - Autoquestionnaire de handicap de l’échelles MSIS et autoquestionnaire d’évaluation de la marche (MSWS12) à un an et à la visite de fin d’étude (voir annexes 8, 9,10) ;
      - Tous les critères de jugement secondaires précédents à 6 mois et 44 semaines.
5. **Déroulement de l’essai :**
   1. **Investigateurs :**Dans chaque centre, chaque patient sera suivi par deux neurologues :
       - Le neurologue traitant (NT) sera chargé de la prise en charge médicale et neurologique du patient et en particulier de l’évaluation des événements indésirables cliniques et biologiques. Il sera en insu du groupe assigné. Il sera responsable de tous les aspects de la prise en charge neurologique (examen clinique, prise en charge des poussées).
       -Le neurologue évaluateur (NE) sera chargé de l’évaluation neurologique des patients : examen neurologique, mesure du temps de marche et du périmètre de marche, autres échelles cliniques. Il pourra être aidé pour la mesure du MSFC et du périmètre de marche par un technicien de recherche clinique ou une infirmière. Il est en insu du groupe de traitement (ainsi que les personnels cités ci-dessus) et ne doit recevoir aucune information du patient ou d’autre membre de l’équipe sur les événements indésirables et les problèmes médicaux du patient autres que le recueil des symptômes neurologiques nécessaire à l’évaluation. Il n’a pas accès aux classeurs de recueil (CRF) du NT. Il transmet les feuilles d’évaluation au NT qui les classe dans le CRF.
   2. **Calendrier de l’essai :**Tableau ( annexe 1)
   3. **Consentement :**
      Lors de la visite de sélection, avant toute autre procédure prévue dans l’essai, le neurologue traitant (NT) informera le patient de l’objectif, de la nature des contraintes et des risques prévisibles de l’essai. Il remettra au patient la lettre d’information détaillée et le formulaire de consentement (figurant en annexe 3, 4). Le patient pourra obtenir toutes les réponses à ses questions de la part du médecin avant toute procédure d’évaluation. Si le patient donne son accord de participation, il inscrira son nom ainsi que le neurologue traitant sur le formulaire de consentement puis ils dateront et signeront ce formulaire en 3 exemplaires. Un exemplaire du consentement sera conservé par l’investigateur en lieu sûr pour une durée de 30 ans après la fin de l’essai. Une autre copie sera remise au patient et le troisième exemplaire sera remis au promoteur à la fin de l’essai.
   4. **Visite de sélection :**
      Elle est assurée par le NT. Au cours de cette visite le NT :
       - vérifie les critères d’éligibilité,
       - Informe le patient des modalités, des contraintes et des risques prévisibles de l’essai et recueille le consentement,
       - procède à un examen clinique général, à une mesure du périmètre de marche permettant de vérifier que le patient remplit les critères d’inclusion en terme d’EDSS (évalué par le NE),
       - recueille les antécédents, les pathologies associées et les traitements en cours et passés,.
       - remet une prescription pour le bilan biologique (NFS, plaquettes, VS, ionogramme sanguin, glycémie, créatininémie, transaminases et pour les femmes en âge de procréer test de grossesse),
      - une mesure du résidu post-mictionnel sera réalisée par bladder scan ou échographie,
      - en absence de bilan radiologique (thorax, sinus, dents) et d’une IDR à la tuberculine datant de moins d’un mois, prescrit ces examens,
      - donne les consignes au patient concernant les poussées.
   5. **Période de sélection (visite 1) :**
      Pendant la période de sélection, située entre la visite de sélection et la visite d’inclusion, qui ne doit pas excéder 5 semaines, est réalisée un examen par imagerie par résonance magnétique avec injection de gadolinium. Cet examen ne fait pas partie des évaluations du protocole puisqu’il est considéré comme rentrant dans le suivi habituel des patients et permettra d’analyser d’éventuels facteurs de prédiction de l’efficacité des traitements. Un protocole recommandé sera proposé aux centres.
      Pendant cette période le NE (ou le technicien de recherche si le centre en dispose) doit faire passer à deux reprises le MSFC (temps de marche sur 10m, 9HPT, PASAT 3s).
      Pendant cette période le NT vérifie sur les résultats du bilan que le patient ne présente pas de critères de non éligibilité puis contacte le centre coordonnateur par fax pour que la randomisation soit effectuée. Une fois que le centre de méthodologie et de gestion de l’essai a informé la pharmacie à quel groupe le patient a été assigné, le NT est informé par le Centre de méthodologie et de gestion qu’il peut convoquer le patient pour sa visite d’inclusion.
   6. **Visite d’inclusion  et d’initiation de traitement (visite 2):**
      Cette visite a lieu en hospitalisation.
      Le NT informe le patient des résultats biologiques et après avoir vérifié que le patient remplit toujours les critères d’éligibilité procède à l’inclusion définitive.
      Il effectue un examen clinique général, note les traitements associés et les événements indésirables.
      Le NE (avec l’aide éventuelle d’un technicien de recherche) procède à :
       - un examen neurologique,
       - une mesure du périmètre de marche sur 500 mètres chronométré avec l’aide minimum,
       - une mesure du temps de marche sur 8 m,
       - une mesure du NHPT, deux essais par main,
       - un test de la PASAT 3s,
      Il doit remettre au patient les auto questionnaires que le patient doit remplir avant le début du traitement et récupérer les questionnaires remplis.
      Le NE remet au NT les résultats des évaluations et les questionnaires qui sont classés dans le CRF et dont une copie est transmise au centre de méthodologie et de gestion de l’essai.
      Avant le traitement il faut peser le patient, prendre la TA et la température, réaliser un ECG, et faire un examen des bandelettes urinaires : si les bandelettes sont positives  il faut prélever un ECBU. Les procédures du traitement sont décrites au chapitre 5.2.
      La procédure pour le traitement anti-émétique est décrite au chapitre 6.
      - Après le traitement le NT recueille les événements indésirables éventuels et procède à un examen clinique.
      - Les ordonnances (traitement anti-émétique éventuel et bilan biologique) sont remises au patient ainsi que son rendez-vous pour l’hospitalisation suivante.
   7. **Visites de suivi et hospitalisations pour traitement :**
      Les visites et traitements ont lieu toutes les 4 semaines durant 44 semaines puis toutes les 8 semaines, les 44 semaines suivantes en hospitalisation de jour.
      Si une des conditions indiquées au paragraphe 5.2 est présente le centre de méthodologie et de gestion de l’essai en informe le NT qui propose au patient une visite non programmée et décale la visite et le traitement programmés.
      Si le centre de méthodologie et de gestion de l’essai a donné son feu vert pour le traitement le patient est hospitalisé.
      Le NT effectue un examen clinique général, note les traitements associés et les évènements indésirables.
      Le NE procède à :
       - un examen neurologique,
       - une mesure du périmètre de marche sur 500 mètres chronométré avec l’aide minimum,
       - une mesure du temps de marche sur 8 m (aux visites 13, 19, 20 et en cas de visite de fin de traitement prématuré),
       - une mesure du NHPT, deux essais par main (aux visites 13, 19,20 et à la visite de fin d’étude et en cas de visite de fin de traitement prématuré),
       - un test de la PASAT 3s (aux visites 13, 19, 20 et en cas de visite de fin de traitement prématuré).
      Il doit remettre au patient les auto questionnaires que le patient doit remplir avant le début du traitement à la visite 2,13 et 19.
      Le NE remet au NT les résultats des évaluations pour classement dans le CRF et une copie est transmise au centre de méthodologie et de gestion de l’essai.
      Les procédures de traitement sont identiques à celle de la visite d’initiation de traitement.
      - Après le traitement le NT recueille les événements indésirables éventuels et procède à un examen clinique.
      - Les ordonnances (traitement anti-émétique éventuel et bilans biologiques) sont remises au patient ainsi que son rendez-vous pour l’hospitalisation suivante.
   8. **Visites de fin de traitement et de fin d’étude :**
      Huit semaines après la dernière administration programmée de traitement a lieu la visite de fin d’étude. Dans la période de 8 semaines qui précède cette visite une IRM encéphalique, rentrant dans le suivi normal de ces patients sera réalisée.
      Au cours de cette visite :
      Le NT procède au recueil des événements indésirables, traitements associés et procède à un examen clinique.
      Le NE procède à :
       - un examen neurologique,
       - une mesure du périmètre de marche sur 500 mètres chronométré avec l’aide minimum,
       - une mesure du temps de marche sur 8 m,
       - une mesure du NHPT, deux essais par main,
       - un test de la PASAT 3s,
      Il doit remettre au patient les auto questionnaires que le patient doit remplir sur place.
      Le NE remet au NT les résultats des évaluations pour classement dans le CRF et une copie est transmise au centre de méthodologie et de gestion de l’essai.
      Un questionnaire d’évaluation de l’insu est rempli par le patient, le NE et le NT et le technicien de recherche éventuel.
      En cas d’arrêt prématuré de traitement (Cf. paragraphe Déviations au protocole) une visite de fin de traitement est organisée huit semaines après la dernière administration au cours de laquelle le NT évalue les événements indésirables, les traitements associés et procède à un examen clinique. Si le patient ne souhaite pas poursuivre le suivi il est procédé à la visite de fin d’étude comme indiqué au début de ce paragraphe. Sinon les évaluations sont poursuivies comme prévues par le protocole jusqu’à la date programmée de visite de fin d’étude, date à laquelle a lieu la visite de fin d’étude. Les traitements reçus par le patient durant cette période seront recueillis.
   9. **Survenue du critère principal de jugement :**
      Le centre coordonnateur établit l’EDSS pour chaque patient dès réception des données de base des évaluations (périmètre de marche mesuré et examen neurologique). Dès qu’un patient présente le critère de jugement principal (augmentation de 1 point d’EDSS si l’EDSS initial est 4 ou 4,5 ou de 0,5 point si l’EDSS initial est 5, 5,5 ou 6, confirmée à la visite réalisée quatre mois après) il en informe le NT. Lors de la visite suivante le NT doit en informer le patient et réévalue avec celui-ci les options thérapeutiques. Si le patient décide d’arrêter le traitement à l’essai et s’il est d’accord, il sera suivi jusqu’à la fin de l’essai selon le même calendrier que prévu initialement. Le maintien du patient dans l’essai permettra une meilleure analyse des critères de jugement secondaires.
   10. **Levée d’insu :**

En cas d’événement indésirable grave pour lequel une levée d’insu pourrait être nécessaire (légitime uniquement si la connaissance du traitement administré peut modifier la prise à charge à effectuer), le neurologue traitant contacte l’investigateur coordonnateur pour prendre cette décision après discussion.

Pour chaque levée d’insu, le neurologue traitant doit noter précisément les raisons, la date et le nom de la personne qui a effectué la levée d’insu. Toute levée d’insu doit être notifiée au Centre de Méthodologie et de Gestion de l’essai.

En cas d’urgence mettant en jeu la vie ou la santé du patient, le neurologue traitant pourra demander au pharmacien responsable du centre de lever l’insu s’il juge que cette information est indispensable à la prise en charge du patient sans attendre l’accord de l’investigateur coordonnateur.

1. **Evènements indésirables**
   1. **Définition et déclaration d’un évènement indésirable**
      1. **Définitions:**Un événement indésirable est une manifestation nocive et non recherchée, subie par une personne participant à une recherche biomédicale, quelle que soit la cause de cette manifestation, et qui n'est pas nécessairement liée au traitement évalué ou à la recherche.
         Tous les événements indésirables survenus pendant l'étude seront recherchés et reportés dans le cahier d'observation.
         Les événements suivants sont définis comme événements indésirables graves (EIG) : - Décès ou événement menaçant le pronostic vital (en particulier événement indésirable de grade 4).
          - Evénement entraînant une incapacité organique ou fonctionnelle ;
          - Evénement nécessitant ou prolongeant une hospitalisation (à l’exception des hospitalisations de jour prévues pour l’administration du traitement à l’essai et des hospitalisations pour poussée de SEP en l’absence de complications) ;
         - Anomalie congénitale touchant la descendance d’un patient ayant reçu le traitement de l’essai ;
         -.**Evénements potentiellement graves**
         Il s'agit d'événements indésirables ou de résultats anormaux d'analyse biologique, en tenant compte du contexte.
         **Evénements inattendus**
         Evénements dont la nature, la gravité, l'évolution ou la fréquence ne concordent pas avec les informations relatives au produit (Brochure investigateur).
      2. **Déclaration et suivi des EIG :**.
         Déclaration
         Tout EIG doit être déclaré, s'il survient à partir de la date de signature du consentement et pendant toute la durée de suivi de la personne participant à l'étude. Tout événement indésirable grave (EIG), potentiellement grave ou inattendu, doit être déclaré par le neurologue traitant dès qu'il en a connaissance quelle que soit son imputabilité à la recherche ou au traitement de l'étude selon les procédures définies au CHU de Bordeaux
         Ces déclarations se feront par transmission par fax de la fiche de déclaration des événements indésirables graves figurant dans le cahier d'observation :
         - au centre régional de Pharmacovigilance de Bordeaux Aquitaine (CRPV) qui évalue l'imputabilité (UGRC du CHU de Bordeaux),
         - au Centre de Méthodologie et de Gestion de l’étude,
         - et au CCPPRB en cas de décès
         Si l’imputabilité de l’Evènement Indésirable Grave, inattendu ou potentiellement grave, n’est pas exclue, le CRPV transmettra le dossier dans un délai de 48 heures après la connaissance de l'événement au promoteur (UGRC du CHU de Bordeaux) pour déclaration aux autorités de santé (AFSSAPS). avec copie au centre de méthodologie et de gestion et au comité indépendant de surveillance.
         **Suivi de l'événement après sa notification**
         Après sa notification initiale, l'EIG doit obligatoirement faire l'objet d'un suivi jusqu'à sa résolution.
         Le NT doit recueillir toutes informations complémentaires relatives à l'EIG sur le formulaire de déclaration complémentaire d'un événement indésirable grave et le faxer dès qu'il en a connaissance au CRPV de Bordeaux et au Centre de Méthodologie et de gestion de l'étude.
      3. **Conduite à tenir en cas d’évènement indésirable grave (EIG)**
         La survenue d’un événement indésirable grave peut nécessiter un arrêt immédiat du traitement administré dans le cadre de l’essai, sur décision du NT. Le traitement d’urgence sera adapté à la situation rencontrée et, si nécessaire, au bras de randomisation du patient (l’investigateur s’adressera alors au pharmacien du site d’hospitalisation pour une levée d’insu). (Cf. Chapitre Levée d’insu).
   2. **Conduite à tenir en cas de survenue de grossesse
      Notification d'une grossesse**La survenue d'une grossesse dans la période ou au décours immédiat d'une étude chez une patiente participant à l'étude ou, chez la partenaire d'un patient participant à l'étude doit être déclare par l'investigateur selon les mêmes modalités qu'un EIG
      De même, toute interruption volontaire de grossesse (IVG), interruption thérapeutique de grossesse (ITG) ou fausse couche (FC) nécessitant une hospitalisation est un EIG à déclarer
      **Suivi de la grossesse après notification**La grossesse fera l'objet d'un suivi particulier jusqu'à l'accouchement ou l'IVG **Conséquences pour la prise en charge :** La survenue d’une grossesse chez une patiente participant à l'étude nécessite, après confirmation, une levée d’insu sur le bras de traitement ainsi qu’un arrêt immédiat de celui-ci. Cette décision devra également être confirmée par le Comité de gestion. Toutes les mesures devront être prises pour adapter le suivi de cette grossesse au traitement attribué à la patiente pendant l’essai.
2. **Monitorage**
   1. **Organisation générale.**Afin d’assurer la mise en place, le suivi et le monitorage des centres un attaché de recherche clinique senior (chef de projet, CP) et trois attaché(e)s de recherche clinique (ARC) basé(e)s sur le centre coordonnateur seront recruté(e)s.
      Le CP aidera l'investigateur coordonnateur (Pr Bruno Brochet ) à élaborer les différents documents de l'étude en collaboration avec le Centre de Méthodologie et de Gestion.
      Le CP et les ARC assureront la mise en place de l’étude dans les différents centres. Au niveau du centre de méthodologie et de gestion de l’essai le CP, aidé des ARC, effectuera le suivi de l’essai, des inclusions, des EIG et des paramètres d’évaluation cliniques et biologiques. Il transmettra toutes les données nécessaires au suivi de l’essai et en particulier toutes les données biologiques à l’équipe médicale coordinatrice du centre de méthodologie et de gestion afin que celle-ci statue sur les adaptations de dose. Il transmettra aux pharmaciens les instructions relatives aux doses. Il transmettra les informations utiles également, au conseil scientifique et au comité de surveillance.
      Visite de Monitorage
      Les ARC assurent le monitorage de tous les centres par une visite sur site une fois tous les 4 mois environ à partir de la première inclusion, par des transmissions de courrier et de fax, et des appels téléphoniques réguliers.
      La validation des données portera sur 100% des données clé (consentement éclairé, critères d'éligibilité, dates de suivi, événements indésirables graves, doses prescrites et reçues du traitement, évaluation neurologique).
      Le CP et les ARC transmettent les données au centre de saisie selon un rythme et des modalités qui seront déterminées au début de l’essai.
   2. **Cahiers d’observation :**
      Dans chaque centre, les données seront recueillies sur des cahiers d' observation (CRF) imprimés en dupliqué qui seront fournis au début de l’étude. Ils sont gérés par le neurologue traitant (NT). Le NE n’a pas accès au CRF. Des formulaires d’évaluation seront remis au NE qui les transmettra au fur et à mesure au NT, avec les auto questionnaires remplis, qui les classera après transmission d’une copie au centre de méthodologie et de gestion.
      Les CRF seront conservés dans le centre pendant toute la durée de l’étude jusqu’à la fin de l’analyse afin de ne pas compromettre l’insu le NE disposera d’un dossier source séparé.
   3. **Audit**
      Le promoteur ou les autorités de santé peuvent pratiquer un audit des données pour vérifier le déroulement de l'étude et sa gestion selon les Bonnes Pratiques Cliniques. Il pourra également s'agir d'un audit indépendant mandaté par le promoteur.
   4. **Archivage des documents à la fin de l'étude**Les médecins investigateurs archiveront et conserveront - pendant au moins 15 ans après la fin de l’étude les documents suivants relatifs aux études : version actualisée du protocole et des annexes et amendements éventuels ; cahiers d’observation; tous les autre documents et correspondance relatifs à l’essai.
      -pendant une durée de 30 ans suivant la fin de l'étude : un exemplaire des consentements éclairés signés des participants stockés dans une enveloppe scellée.
      Les dossiers sources des patients devront également rester disponibles pendant tout ce temps.
      Le promoteur archivera
      - pendant une durée de 15 ans : Le protocole et les amendements éventuels au protocole, les cahiers d'observation, tous les autres documents et courriers relatifs à l'étude
      - pendant une durée de 30 ans suivant la fin de l'étude : un exemplaire des consentements éclairés signés des participants stockées dans une enveloppe scellée.
3. **Surveillance de l’essai :**
   1. **Conseil scientifique**Le protocole a été élaboré après discussions au sein du conseil scientifique qui est composé des investigateurs principaux de chaque centre :
      Pendant la durée de l’essai il se réunira au moins tous les 3 mois (réunion téléphonique ou physique). Il s’assure de la bonne marche de l’essai et du respect du protocole, et vérifie ses aspects. éthiques. Il s’informe auprès du Centre de Gestion de l’état d’avancement de l’essai, des problèmes éventuels et des résultats. Il décide toute modification pertinente du protocole nécessaire à la poursuite de l’essai. En présence d’un rythme d’inclusion trop lent, d’un trop grand nombre de perdus de vue, de violations du protocole, ou pour des raisons médicales et/ou administratives, le Conseil Scientifique se réserve le droit de prendre la décision d’interrompre ou de poursuivre l’essai. Il précisera, avant la fin de l’essai, les modalités éventuelles du suivi prolongé des patients inclus dans l’essai.
   2. **Centre de Méthodologie et de Gestion de l’essai**Le Centre de Méthodologie et de Gestion de l’essai est situé à Bordeaux. Il comprend l’équipe médicale coordinatrice dirigée par le Pr B Brochet, Fédération des Neurosciences Cliniques du CHU de Bordeaux, Mme Mathilde Deloire, Chef de Projet, l’équipe de l’Unité de Soutien Méthodologique à la Recherche Clinique et Epidémiologique du CHU de Bordeaux (USMR, Dr Paul Perez, méthodologiste, C Germain Statisticienne et leur collaborateurs) et Mr Jean Grellet, Pharmacien Coordonnateur. Le centre de méthodologie et de gestion sera aidé par le recrutement de 3 ARC.
      Une réunion mensuelle de l’équipe au complet, puis trimestrielle, après 3 mois, permet de suivre l’avancement de l’essai. L’équipe réalise la mise en place de l’essai, la randomisation des patients, le recueil et le monitorage des données de l’essai, la gestion de la base de données. Le centre coordonne le fonctionnement des centres investigateurs, des pharmacies hospitalières. Il informe le Conseil Scientifique du déroulement de l’essai, prépare les réunions des différents comités et les assemblées générales des investigateurs.
   3. **Comité de suivi et de validation des évènements**Il est localisé à Bordeaux et est composé de membres de l’équipe médicale coordinatrice dirigée par le Pr B Brochet et est complété lors de réunions élargies d’experts extérieurs.
      Il se réunit chaque semaine tout au long de l’essai et organise des réunions élargies au moins une fois par trimestre.
      Le Comité de Validation des Evénements est chargé d’analyser et de classer les événements indésirables et leur imputabilité aux traitements de l’essai, d’assurer le suivi biologique prévu lors de l’essai et de communiquer aux pharmaciens les ajustements de posologie ou de traitement qui en découlent. Il est également chargé d’analyser et de valider les diagnostics de progression clinique selon le critère de jugement principal et d’en informer les investigateurs.
   4. **Comité indépendant de surveillance**Il est composé d’experts en neurologie, en pharmacovigilance et en méthodologie ne participant pas à l’essai.
      Le Comité Indépendant de Surveillance se réunit au moins une fois au début de l’essai, et une fois pour examiner les données de tolérance des 30 premiers sujets inclus puis tous les 3 mois (par conférence téléphonique). Il examine le rythme des inclusions afin de vérifier si l’essai permettra d’atteindre ses objectifs et il s’assure que l’essai se déroule conformément au protocole approuvé par le CCPPRB et conformément à la législation. Il revoit toutes les données disponibles sur les événements indésirables survenus au cours de l’essai.
4. **Aspects statistiques :**
   1. **Nombre de sujets nécessaire :**Le critère de jugement principal est le délai d’aggravation du score EDSS (Cf. Chapitre 7).
      L’aggravation est définie :
       - par une augmentation du score de plus d’1 point (EDSS initial égal à 4 ou 4,5 points) ou de plus de 0,5 points (EDSS initial entre 5 et 6 points inclus),
       - confirmée lors d’une visite de contrôle réalisée 4 mois après la 1^ère^ visite montrant cette aggravation ou 2 mois après si l’aggravation est constatée lors de la dernière visite de la période de traitement.
      Le délai d’aggravation sera calculé par la différence entre la date de la 1^ère^ visite avec aggravation et la date d’initiation du traitement.
      Le calcul de la taille de l’échantillon a été réalisé grâce au logiciel N-Query® (v 4.0). La proportion attendue de patients sans aggravation à 2 ans est de 75% dans le groupe CPM (76% à deux ans dans l’étude de Hohol et al, 1999) et de 60% dans le groupe MP ( Kinkel, 1999).
      Si l’on fait le calcul pour pouvoir comparer les courbes de survenue d’une aggravation dans chacun des groupes par un test du logrank de formulation bilatérale, avec un risque α = 5% et une puissance (1-β) = 80%, le nombre de sujets à inclure par groupe doit être au minimum de 155 patients (2 groupes de même effectif). Pour une proportion attendue de perdus de vue égale à 5%, il est nécessaire d’inclure au moins 155 patients par groupe. Par précaution, 180 patients seront inclus dans chacun des 2 groupes.
   2. **Méthodes statistiques prévues pour l’analyse:**
      1. **Généralités :**L’analyse sera réalisée en intention de traiter, c’est à dire que tous les patients qui ont fait l’objet de la randomisation seront analysés dans leur groupe de randomisation, même s‘ils n’ont jamais pris le traitement, s’ils l’ont arrêté en cours d’étude, ou s’ils ont changé de traitement. Il n’est pas prévu d’analyse intermédiaire. Pour les tests statistiques, le risque de première espèce α est fixé à 5%. Les analyses statistiques seront réalisées avec le logiciel SAS.
      2. **Description de l’inclusion et du suivi :**Le nombre de patients inclus, la courbe des inclusions (évolution du nombre de patients inclus entre la première et la dernière inclusion), le nombre de visites théoriques correspondant au nombre de patients inclus et le nombre de visites réellement effectuées seront présentés et comparés entre groupes.
      3. **Caractéristiques des patients avant la mise sous traitement :** Les patients seront décrits selon le groupe de traitement et en fonction des violations au protocole :
         - Patients exclus de l’analyse / Patients inclus dans l’analyse.
         - Patients inclus dans l’analyse avec violations mineures du protocole / Patients inclus dans l’analyse sans violation mineure du protocole.
         Les caractéristiques des patients avant la mise sous traitement seront décrites par groupes : fréquences pour les variables qualitatives ; moyennes, écarts-types, médianes, minimum et maximum pour les variables quantitatives
      4. **Analyse du critère de jugement principal :**Le critère de jugement principal est le délai d’aggravation du score EDSS (Cf. Chapitre 7), estimé 2 ans après le début du traitement. L’analyse sera réalisée lorsque tous les patients auront terminé l’ensemble du suivi prévu dans l’étude.
         Dans un premier temps, les courbes de survenue d’une aggravation seront tracées à l’aide de la méthode de Kaplan-Meier et les délais de survenue seront comparés entre groupes de traitement par un test du logrank.
         Dans un second temps, un modèle de Cox sera construit pour étudier l’effet des facteurs pronostiques connus sur l’efficacité du traitement.
         Les facteurs pronostiques analysés sont :
          - l’existence de poussées dans les 12 derniers mois précédant le début du traitement,
          - la présence de lésions rehaussées par le produit de contraste à l’IRM réalisée avant l’initiation du traitement,
          - la durée de la maladie à la date d’initiation du traitement.
         Le modèle de Cox prendra en compte chacun de ces 3 facteurs pronostiques ainsi que l’interaction entre le traitement et l’existence de poussée ou la présence de lésions rehaussées par le produit de contraste afin d’étudier une éventuelle différence d’effet des traitements en fonction de l’existence de ces facteurs pronostiques. En cas d’interaction statistiquement significative, les résultats des deux traitements seront décrits et comparés dans chaque sous-groupe de facteur pronostique.
      5. **Analyse des critères de jugement secondaires :**- Proportion de patients aggravés à 2 ans :

La proportion de patients aggravés à 2 ans sera décrite dans les 2 groupes de traitement (proportion et intervalle de confiance à 95 % de cette estimation). La comparaison des proportions de patients aggravés à 2 ans sera réalisée par un test du Chi² si les effectifs théoriques sont suffisants (n ≥ 5) ou par un test de Fisher sinon.

- Z-scores et MSFC :
Le calcul des z scores utilisera comme population de référence les données de la population à l’inclusion selon les recommandations internationales (Cutter et al ., 1999).
Les moyennes des z-scores et du MSFC seront comparées par un test de Student si les conditions d’application sont respectées (variances homogènes, distributions normales). Si les variances ne sont pas homogènes, on utilisera un test de Student pour variances inégales. Si la distribution n’est pas normale, on utilisera un test de Mann-Withney

- Nombre de poussées :

Le nombre de poussées sera décrit dans les deux groupes de patients (moyenne et intervalle de confiance à 95 %, médiane, minimum-maximum). Les moyennes du nombre de poussées observées dans les deux groupes seront comparées par un test de Student si les conditions de validité du test sont respectées (distribution normale, variances homogènes). Si les variances sont inégales entre les deux groupes, on utilisera un test de Student pour variances inégales, si la distribution du nombre de jours n’a pas une distribution normale, on utilisera un test non paramétrique de Mann-Whitney

- Comparaison des groupes pour la tolérance
La proportion d’événements indésirables graves et non graves survenus au cours du suivi dans chaque groupe de traitement sera décrite, et les groupes seront comparés pour la fréquence de survenue de ces événements, leur gravité et leur imputabilité aux traitements de l’essai. Les comparaisons de proportions seront réalisées à l’aide du test du Chi-2 si les effectifs théoriques sont suffisants (n ≥ 5), ou par un test exact de Fisher sinon.
L’analyse du délai de survenue d’un événement sera effectuée en prenant en compte uniquement la première survenue de l’événement. Les courbes de survenue d’événement seront tracées à l’aide de la méthode de Kaplan-Meier. Les tests de comparaison du délai de survenue de l’événement entre groupes de traitement seront réalisés par un test du log-rank.

- - 1. **Analyse des critères de jugement exploratoires :**- Autoquestionnaire de qualité de vie à 2 ans, autoquestionnaire de handicap de l’échelles MSIS et autoquestionnaire d’évaluation de la marche à un an et à la visite de fin d’étude :

Pour décrire l’évolution de chaque score au cours du suivi, pour ces questionnaires, les moyennes des scores seront comparées entre les deux groupes de traitement par une analyse de variance avec mesures répétées dans le temps.

- Pour chacun des critères secondaires (proportion de patients aggravés à 2 ans, Z-scores et MSFC, nombre de poussées), les mêmes analyses (cf 12.2.5) seront faites à 6 mois, ainsi qu’à 44 semaines. Une analyse de variance avec mesures répétées dans le temps sera réalisée pour décrire l’évolution au cours de la période de suivi de chacun de ces critères.

1. **Considérations éthiques** et réglementaires
   1. **Considérations éthiques générales et avis du CCPPRB**Le promoteur et les investigateur s'engagent à ce que cette étude soit réalisée conformément :
       - au texte de la déclaration d’Helsinki adopté par l’Assemblée Mondiale en juin 1964, modifié à Tokyo (Octobre 75), à Venise (Octobre 83), et à Hongkong (Septembre 89), Washington 2002, Tokyo 2004
       - aux recommandations des Bonnes Pratiques Cliniques (ICH 4 du 1^er^ Mai 1996)
       - au texte de la loi française n°88-11.38 sur la protection des personnes se prêtant à des recherches biomédicales (loi Huriet du 20/12/88, modifiée le 23/01/90)
      - L'étude est conduite conformément au protocole, hormis dans les situations d'urgence nécessitant la mise en place d'actes thérapeutiques précis. Les investigateurs s'engagent à respecter le protocole en tous points en particulier en ce qui concerne le recueil du consentement, la notification et le suivi des événements indésirables graves.
      Le protocole de cette étude, la note d'information aux patients et le formulaire de consentement (cf Annexe 3, 4) sera soumis au CCPPRB de Bordeaux et ne pourra débuter qu’après avoir reçu un avis favorable. La version définitive sera approuvée par le promoteur et tous les investigateurs participant à l’essai.
      Le promoteur adresse avant le début du protocole une lettre d'intention à l'Agence Française de Sécurité des Produits de Santé (AFSSAPS) et informe les directeurs de hôpitaux concernés.
      Les données enregistrées à l'occasion de cette étude feront l'objet d'un traitement informatisée à l'USMR du CHU de Bordeaux dans le respect de la loi "Informatique et Libertés" du 6 août 2004 complétée par la loi du 1^er^ juillet 1994 et son décret d'application du 9 mai 1995 et par la loi du 4 mars 2002.
      La soumission au CCTIRS et la déclaration à la Commission Nationale de l'Informatique et des Libertés (CNIL) de ce traitement informatisé des données conformément à l'article 40 de la loi "Informatique et Libertés" seront effectuées par l'USMR..
      1. **Amendements du protocole :**Après l’approbation du protocole par le CCPPRB, toute modification substantielle fera l'objet d'un amendement écrit qui sera soumis au Conseil Scientifique, au promoteur de l’essai, au CCPPRB et signé par tous les médecins investigateurs.
         Les amendements mineurs, ne modifiant pas le sens du protocole, sont communiqués au CCPPRB à titre d'information.
      2. **Confidentialité des données :**Chaque patient est identifié par un numéro d’identification, composé d’un numéro de centre investigateur sur 2 chiffres (01 à 99), choisi par le Centre de Méthodologie et de Gestion lors de l’accord de participation du centre, de la première initiale de son nom et de son prénom (code lettre), et d’un numéro de patient dans le centre, sur 2 chiffres (01 à 99), établi de façon séquentielle dans l’ordre d’inclusion des patients. Chaque centre investigateur tiendra à jour une liste de correspondance entre les nom et prénom de chaque patient inclus et son numéro d’identification dans l’essai. Cette liste sera conservée dans un lieu sûr fermant à clé.
         Les données concernant un patient inclus et nécessaires à l’essai sont reportées dans le cahier d’observation du patient par le neurologue traitant, après chaque visite prévue dans le protocole. Il y insérera les fiches d’évaluation que le neurologue évaluateur lui transmet. Seuls sont notés sur le cahier d’observation le numéro d’identification du patient et le code lettre. Les cahiers d’observation sont conservés à l’écart du dossier clinique du patient, dans un lieu sûr. Seuls les doubles des fiches du cahier d’observation sont adressés au Centre de Méthodologie et de Gestion. Les doubles des fiches sont saisis en double saisie, puis classés dans un lieu sûr fermant à clé. Les données sont régulièrement chargées dans la base de données, gérées à travers un système de gestion de base de données et une application développée spécifiquement pour l’essai. Dans la base de données, chaque patient n’est identifié que par son numéro d’identification. L’accès à cette base de données est protégé par un mot de passe. Les actions possibles sur les données sont fonction des autorisations attribuées à chaque utilisateur de la base.
         Les patients seront informés que toutes les données de l’essai seront informatisées et conservées de façon confidentielle.
      3. **Assurance**Le CHU de Bordeaux, promoteur de cet étude a souscrit une assurance responsabilité civile auprès de la société Gerling France conformément aux dispositions de l’article L209.7 du code de santé publique du 20/12/1988 et art. 5 du 25/07/1991. Une copie de l'attestation d'assurance est jointe en Annexe 12.
2. **Déviations au protocole :**
   1. **Arrêt de traitement, abandon de l’essai :**Le traitement peut être arrêté :
       - en cas de retrait de son consentement par le patient. Tout patient peut décider de se retirer de l’étude à n’importe quel moment sans en fournir de raisons. Cependant l’investigateur peut demander au patient quelle est cette raison en lui précisant qu’il peut ne pas répondre à cette question.
       - survenue d’une grossesse ou d’une autre condition de non inclusion,
       - survenue d’un EIG nécessitant selon l’avis du NT un arrêt du traitement ou survenue d’un nouvel état morbide justifiant, selon le NT l’arrêt du traitement.
      En cas d’arrêt prématuré du traitement tout effort doit être fait pour poursuivre le suivi jusqu’à la fin de l’essai selon le même calendrier que prévu initialement.
      Quand le neurologue traitant envisage un arrêt prématuré de traitement, le centre de méthodologie et de gestion doit être avisé immédiatement (dans un délai de 3 jours ouvrables) par télécopie. Une visite de fin de traitement et éventuellement de fin d’étude doit être organisée un mois après la dernière administration comme indiqué paragraphe 8.8.
      Lorsqu’un patient souhaite abandonner l’essai, comme il a le droit de le faire à tout moment, le NT proposera une visite de fin d’étude comme indiquée paragraphe 8.8 et devra en aviser le centre coordonnateur par fax et courrier.
      Après l’abandon de l’essai par la patient celui-ci bénéficiera d’une prise en charge de son état de santé la meilleure possible compte tenu des connaissances du moment. Les patients ayant quitté l’étude,ne seront pas remplacés.
   2. **Fermeture des centres, Violations de protocole :**En cas d’absence d’inclusion après 6 mois le centre de gestion se réserve le droit de fermer un centre et de le remplacer.
      En cas de violation majeure au protocole manifeste (absence de consentement, erreur d’attribution de traitement) le centre de gestion se réserve le droit de retirer un patient du protocole et éventuellement de fermer un centre si les mesures prises pour corriger ces violations ne sont pas suivies d’effet. Les patients retirés du protocole ne seront pas remplacés.
      Les violations mineures au protocole seront notées afin d’être prise en compte dans l’analyse.
      En cas de perdus de vus les investigateurs devront tenter d’obtenir du patient des informations sur son devenir, et en particulier sur la survenue éventuelle du critère du jugement principal ou d’EIG.
   3. **Modifications du schéma thérapeutique**
      Les modifications éventuelles de dose ou de rythme d’administration du produit évalué (report d’une administration du traitement en cas de signes de toxicité) devront être notées dans le cahier d’observation.
3. **Publications des résultats :**A l’issue de cette étude un comité de rédaction rédigera les publications scientifiques exposant les résultats et les soumettra aux journaux scientifiques internationaux les plus appropriés selon un choix pris par ce conseil scientifique à la majorité.
   Sur ces publications il sera mentionné que le CHU de Bordeaux est promoteur de cet essai. Il sera fait également mention des sources de financement.
   La liste des auteurs sera proposée par le Comité de Méthodologie et de Gestion au comité scientifique en fonction de la participation à l’élaboration du protocole, à son déroulement, à l’analyse des données et à la rédaction du manuscrit. Les autres personnes ayant contribué à l’étude seront réunies sous l’appellation «groupe d’étude français du cyclophosphamide dans la sclérose en plaques ».
   Les noms de tous les investigateurs de tous les centres seront cités et remerciés en fin d’article.
4. **Aspects Budgétaires, Surcoûts :**
   Afin d’assurer la réalisation de l’étude un financement a été obtenu auprès du programme hospitalier de recherche clinique national 2004 Ce budget doit couvrir les frais médicamenteux inhérents à l’essai (cyclophosphamide), les surcoûts générés au niveau des pharmacies hospitalières par le travail de préparation et d’étiquetage des traitements à l’essai, le coût d’analyse statistique, les fournitures (CRF)’et le recrutement d’un chef de projet et des ARC.
   Les traitements préventifs des effets secondaires émétisants seront considérés comme des surcoûts et leur étiquetage par la pharmacie sera pris en compte.
5. **Références**- Amato MP, Battaglia MA, Caputo D, Fattore G, Gerzeli S, Pitaro M, Reggio A, Trojano M; Mu. S. I. C. Study Group. The costs of multiple sclerosis: a cross-sectional, multicenter cost-of-illness study in Italy. J Neurol. 2002 Feb;249 (2):152-63
   - Brochet B. Aspects physiopathologiques, cliniques, thérapeutiques de la sclérose en plaques. Encyclop.Méd.Chir. (Paris, France), Neurologie, 17-074-B-10, 2001a, 26p.
   - Brochet B. Principales échelles neurologiques utilisées en pratique courante: sclérose en plaques. . Encyclop.Méd.Chir. (Paris, France), Neurologie, 17-035-A-81-2001b, 9p.
   - Brochet B. Indications thérapeutiques lors des poussées de sclérose en plaques. Rev Neurol (Paris) 2001c, 157: 988-995
   - Canadian Cooperative Multiple Sclerosis Study Group. The Canadian cooperative trial od cyclophosphamide and plasma exchange in progressive multiple sclerosis. Lancet, 1991, 337: 442-446
   - Cohen JA, Cutter GR, Fischer JS et al., Benefit of interferon beta 1 a on MSFC progression in secondary progressive MS. Neurology, 2002, 59: 679-687.
   - Comabella M, Balashov K, Issazadeh S et al. Elevated interleukin 12 in progressive multiple sclerosis correlates with disease activity and is normalized by pulse cyclophosphamide therapy. J Clin Invest, 1998, 102: 671-678
   - Cutter GR. Measures of impairment and disability. In : Rudick RA, Goodkin DE eds. Multiple sclerosis therapeutics. London : Martin Dunitz Ltd, 1999 : 19-30
   - Cutter GR, Baier ML, Rudick RA, Cookfair DL, Fisher JS, Petkau J et al. Development of a multiple sclerosis functional composite as a clinical trial outcome measure. Brain 1999 ; 122 : 871-882
   - de Ridder D, van Poppel H, Demonty L, DHooghe B, Gonsette R, Carton H et al. Bladder cancer in patients with multiple sclerosis treated with cyclophosphamide. J Urol 1998 ; 159 : 1881-1884
   - Edan G, Miller D, Clanet M, Confavreux C, Lyon-Caen O, Lubetzki C et al. Therapeutic effect of mitoxantrone combined with methylprednisolone in multiple sclerosis: a randomised multicentre study of active disease using MRI and clinical criteria. J Neurol Neurosurg Psychiatry 1997; 62 : 112-118
   - European Study Group on Interferon β−1b in secondary progressive MS. Placebo-Controlled multicentre randomised trial of interferon β−1b in treatment of secondary progressive multiple sclerosis. Lancet, 1998,352:1491-97
   - Freedman MS, Blumhardt LD, Brochet B, Comi G, Noseworthy JH, Sandberg-Wollheim M, Soelberg-Sørensen and the Paris Workshop group. International consensus on the use of disease-modifying agents in multiple sclerosis. Multiple sclerosis, 2002, 8: 19-23
   - Gauthier SA, Bharanidharan P et al., Treatment of Relapsing Remitting Interferon / Glatiramer Acetate Unresponsive Patients with Pulse Cyclophosphamide. AAN, 2003 (abstract P02-130).
   - Gayou A, Brochet B, Dousset V. Transitional progressive multiple sclerosis: a clinical and imaging study. J Neurol Neurosurg Psychiatry 1997 ; 63 : 396-398
   - Hauser SL, Dawson DL, Lehrich JR et al. Intensive immunosuppression in progressive multiple sclerosis. A randomized three arm study of high dose intravenous cyclophosphamide, plasma exchange and ACTH. NEJM, 1983, 308: 173-180
   - Hartung HP, Gonsette R, Konig N, Kwiecinski H, Guseo A, Morrissey SP, Krapf H, Zwingers T; Mitoxantrone in Multiple Sclerosis Study Group (MIMS). Mitoxantrone in progressive multiple sclerosis: a placebo-controlled, double-blind, randomised, multicentre trial. Lancet. 2002 Dec 21-28;360(9350):2018-25.
   - Hohol MJ, Olek MJ, Orav EJ, Stazzone L, Hafler DA, Khoury SJ et al. Treatment of progressive multiple sclerosis with pulse cyclophosphamide /methylprednisolone: response to therapy is linked to the duration of progressive disease. Mult Scler 1999 ; 5 : 403-409
   - Karni A, Balashov K, Hancock WW et al. Cyclophosphamide modulates CD4+ T cells into a T helper type 2 phenotype and reverses increased interferon gamma production of CD8+ T cells in secondary progressive multiple multiple sclerosis. J Neuroimmunolgy, 2004, 146: 189-198
   - Killian J.M., Bressler R.B., Armstrong R.M., Huston D.P. (1988). Controlled pilot trial of monthly intravenous cyclophosphamide in multiple sclerosis. Arch Neurol, 1988, 145 : 27-30
   - Kinkel RP. Methylprednisolone. In : Rudick RA, Goodkin DE eds. Multiple sclerosis therapeutics. London : Martin Dunitz Ltd, 1999 : 349-370.
   - Kurtzke JF. Rating neurological impairment in multiple sclerosis : an expanded disability status scale (EDSS). Neurology 1983; 33 : 1444-1452
   - Likosky W.H., Fireman B., Elmore R.et al.. Intense immunosuppression in chronic progressive multiple sclerosis: the Kaiser study. J Neurol Neurosurg Psychiatry, 1991, 54: 1055-1060
   - Lublin FD, Reingold SC. Defining the clinical course of multiple sclerosis: results of an international survey. National Multiple Sclerosis Society (USA) Advisory Committee on Clinical Trials of New Agents in Multiple Sclerosis. Neurology 1996 ; 46 : 907-911
   - McDonald WI, Compston A, Edan G, Goodkin D, Hartung HP, Lublin FD, McFarland HF,Paty DW, Polman CH, Reingold SC, Sandberg-Wollheim M, Sibley W, Thompson A, van den Noort S, Weinshenker BY, Wolinsky JS. Recommended diagnostic criteria for multiple sclerosis: guidelines from the International Panel on the diagnosis of multiple sclerosis. Ann Neurol. 2001 Jul;50(1):121-7.
   - Noseworthy JH, Gold R, Hartung HP. Treatment of multiple sclerosis: recent trials and future perspectives. Curr Opin Neurol 1999 ; 12 : 279-293
   - Patti F, Reggio E et al., Rapidly Transitional Multiple Sclerosis Patients Treated with Combination of Cyclophosphamide and Interferon Beta: Follow-Up 36 Months after Discontinuation of Therapy. AAN, 2003 (abstract P02-132).
   - Smith DR; Weinstock-Guttman, B, Cohen JA et al. Blinded, Randomized Trial of Pulse Cyclophosphamide in IFN B Resistant Active MS, AAN, 2003 (abstract S11-005)
   - SPECTRIMS study group. Randomized controlled-trial of interferon beta 1a in secondary progressive MS. Neurology, 2001, 56: 1496-504.
   - Vernay D; Gerbaud L; Biolay S, Coste J, Debourse J, Aufauvre D et al. Qualité de vie et sclérose en plaques: validation de la version française d'un auto questionnaire (SEP-59). Rev Neurol 2000; 156 : 247-63
   - Weiner HL, Mackin GA, Orav EJ, Intermittent cyclophosphamide pulse therapy in progressive multiple sclerosis. Neurology, 1993, 43: 910-918
   -Zephir H, de Sèze J, Duhamel A et al., Treatment of progressive forms of multiple sclerosis with cyclophosphamide: a cohort study of 490 patients. J of Neurol Sci, 2004, 218: 73-77.
6. **Annexes :**1. Calendrier de l’essai.

2. Caractéristiques des traitements à l’essai (Zophren, Cyclophosphamide CPM, méthyprednisolone MP).

3-4. Note d’information et formulaire de consentement.

5 à 10. Echelles d’évaluation des critères de jugement : EDSS, FSS, MSFC, SEP-59, MSIS, MSWS12.
11. Coûts pharmacie

12. Attestation d’assurance responsabilité civile
13. Déclaration d’helsinki

**ANNEXE 1 CALENDRIER**

Les mesures en italiques sont à effectuer par le NE.

**ANNEXE 2 : Caractéristiques des traitements à l’essai**

**1-ENDOXAN® injectable 500 mg** : **cyclophosphamide : BAXTER - Division Oncology**.

**FORMES ET PRESENTATIONS**

*Poudre pour solution injectable à 500 mg :*
Flacon + ampoule de solvant de 25 ml, boîte unitaire.
Modèle hospitalier : Flacon sans solvant, boîte unitaire.

**COMPOSITION**

|  | *p flacon* |
| --- | --- |
| Cyclophosphamide (DCI) anhydre | 500 mg |
| (soit en cyclophosphamide monohydraté : 534,5 mg/fl) | |

| *Solvant :* | *p ampoule* |
| --- | --- |
| Eau pour préparations injectables | 25 ml |

##### DC/INDICATIONS

- Traitement adjuvant et en situation métastatique des adénocarcinomes mammaires.
- Traitement des cancers ovariens, des cancers bronchiques notamment à petites cellules, des séminomes et carcinomes embryonnaires testiculaires, des cancers de la vessie, des sarcomes, des neuroblastomes, des lymphomes malins hodgkiniens et non hodgkiniens, des myélomes multiples, des leucémies aiguës, notamment lymphoïdes.
- A forte dose, conditionnement des allo et autogreffes médullaires.

A dose plus faible, traitement des polyarthrites rhumatoïdes, de certaines formes sévères de lupus érythémateux aigus disséminés, de néphropathies auto-immunes corticorésistantes.

**DC/POSOLOGIE ET MODE D’ADMINISTRATION**

**Posologie :** La posologie du cyclophosphamide est fonction de l'indication thérapeutique (traitement antitumoral ou immunodépresseur, type et localisation de la tumeur, traitement initial ou d'entretien) et de la place du médicament dans le traitement entrepris (utilisé seul ou en association avec d'autres médicaments cytostatiques). Elle est individuelle et doit tenir compte de l'état clinique et hématologique du patient (cf Mises en garde/Précautions d'emploi).

Le cyclophosphamide injectable est habituellement utilisé à des doses moyennes de 150 à 1200 mg/m ^2^ chez l'enfant et de 500 à 4000 mg/m ^2^ chez l'adulte, toutes les 3 à 4 semaines, administrées :

- sur 1 à 3 jours à chaque cycle,
- en deux injections à 7 jours d'intervalle.

**Mode d'administration :** Afin de préparer une solution isotonique prête à l'emploi, la poudre doit être dissoute dans une solution de chlorure de sodium à 0,9 % à raison de 50 ml pour 1 g. En cas de besoin, elle peut également être dissoute dans une solution de Ringer ou du sérum glucosé. La solution reconstituée ne doit pas être conservée au-delà de 48 heures et ne doit pas dépasser une concentration de 2 %.
La voie d'administration habituelle est la voie veineuse en perfusion courte (30 minutes à 2 heures). La perfusion sur 24 heures est également possible. Le médicament préalablement reconstitué dans une solution de chlorure de sodium à 0,9 % est introduit dans le liquide de perfusion (soluté injectable isotonique de glucose ou de chlorure de sodium).
Il est recommandé d'associer l'administration d'Uromitexan à partir de 600 mg/m 2/j et/ou d'assurer une hydratation suffisante.
Dans certains cas (capital veineux altéré), la voie IM peut être utilisée sans dépasser la dose de 500 mg par injection pour des raisons de volume. La dissolution dans du chlorure de sodium à 0,9 % permet d'obtenir une solution isotonique.
D'autres voies peuvent être utilisées comme la voie intra-artérielle.

**DC/CONTRE-INDICATIONS**

**Absolues :**

- Insuffisance médullaire sévère.
- Infection urinaire aiguë, cystite hémorragique préexistante.
- Allergie connue au cyclophosphamide.
- Grossesse et allaitement.
- Vaccin contre la fièvre jaune, phénytoïne à visée prophylactique (cf Interactions).

**Relatives :**

- Vaccins vivants atténués (cf Interactions).

**DC/MISE EN GARDE ET PRECAUTIONS D’EMPLOI**

**Mises en garde :**

- Les patients des deux sexes en période d'activité génitale doivent suivre une contraception efficace.
- La prudence est recommandée en cas d'insuffisance hépatique ou rénale préexistante qui devra, si besoin, être corrigée avant le début du traitement, ou pourra nécessiter une réduction de dose.
- Avant de débuter le traitement, il est nécessaire de contrôler les infections éventuelles et de corriger les troubles électrolytiques importants.
- Pendant le transport et le stockage d'Endoxan, il peut éventuellement se produire une liquéfaction du produit en raison d'une température trop élevée.
  Il est facile de distinguer visuellement les flacons qui ont subi une telle altération : le cyclophosphamide liquéfié apparaît comme un liquide visqueux, incolore ou jaunâtre (habituellement sous forme de gouttelettes ou d'une phase continue). Ne pas utiliser de flacons présentant une telle altération.

**Précautions d'emploi :**

- Une surveillance régulière de l'hémogramme est nécessaire pendant toute la durée du traitement (avant chaque cycle).
- L'utilisation du cyclophosphamide peut nécessiter une adaptation de la posologie ou une variation de l'espacement des cycles chez les patients présentant un diabète insipide, une leucopénie, une thrombopénie ou une infiltration cellulaire tumorale de la moelle osseuse.
- Lors de l'utilisation prolongée ou de l'utilisation de fortes doses du médicament ou chez les patients à risque (radiothérapie antérieure du petit bassin, toxicité thérapeutique vésicale antérieure...), il est recommandé d'assurer une hydratation abondante et d'associer la prise d'Uromitexan pour prévenir les risques de cystite hémorragique (cf Posologie/Mode d'administration). Il convient également de s'assurer que la diurèse du patient est bonne et de pratiquer si nécessaire des recherches d'hématuries microscopiques.
- L'alopécie peut parfois être prévenue par la mise en place d'un garrot pneumatique à la racine des cheveux lors du traitement ou par celle d'un casque réfrigérant.

**DC/INTERACTIONS**

**Interactions médicamenteuses :** En raison de l'augmentation du risque thrombotique lors des affections tumorales, le recours à un traitement anticoagulant est fréquent. La grande variabilité intra-individuelle de la coagulabilité au cours de ces affections, à laquelle s'ajoute l'éventualité d'une interaction entre les anticoagulants oraux et la chimiothérapie anticancéreuse, imposent, s'il est décidé de traiter le patient par anticoagulants oraux, d'augmenter la fréquence des contrôles de l'INR.

*Associations contre-indiquées :*

- Phénytoïne (introduite en prophylaxie de l'effet convulsivant de certains anticancéreux) ; décrit pour busulfan, ifosfamide, étoposide, téniposide : risque de majoration de la neurotoxicité (busulfan, ifosfamide) ou de perte d'efficacité du cytotoxique (étoposide, téniposide) par augmentation du métabolisme hépatique du cytotoxique par la phénytoïne.
- Vaccin contre la fièvre jaune : risque de maladie vaccinale généralisée mortelle.

*Associations déconseillées :*

- Vaccins vivants atténués (sauf fièvre jaune) : risque de maladie vaccinale généralisée éventuellement mortelle. Ce risque est majoré chez les sujets déjà immunodéprimés par la maladie sous-jacente.
  Utiliser un vaccin inactivé lorsqu'il existe (poliomyélite).

*Associations nécessitant des précautions d'emploi :*

- Phénytoïne (en cas de traitement antérieur à la chimiothérapie) ; décrit pour busulfan, ifosfamide, étoposide, téniposide : risque de majoration de la neurotoxicité (busulfan, ifosfamide) ou de perte d'efficacité du cytotoxique (étoposide, téniposide) par augmentation du métabolisme hépatique du cytotoxique par la phénytoïne. Surveillance clinique et adaptation de la posologie de l'anticancéreux.

*Associations à prendre en compte :*

- Ciclosporine (décrit pour doxorubicine, étoposide) : immunodépression excessive avec risque de lymphoprolifération.
- Tacrolimus (par extrapolation à partir de la ciclosporine) : immunodépression excessive avec risque de lymphoprolifération.

**DC/EFFETS INDESIRABLES**

La tolérance générale et locale du cyclophosphamide est bonne.
Une neutropénie et rarement une thrombopénie modérée peuvent être observées : elles sont toujours spontanément réversibles après diminution de la posologie ou à l'arrêt du traitement.
Certains patients peuvent présenter des nausées associées ou non à des vomissements qui sont facilement prévenus ou supprimés par les antiémétiques.
A forte dose, on peut également observer une toxicité gastro-intestinale à type de mucites et/ou de diarrhées.
L'alopécie est inconstante, transitoire et réversible.
Lors de l'emploi de doses élevées ou lors de traitements prolongés, il existe un risque de cystite hémorragique (cf Mises en garde/Précautions d'emploi) ainsi qu'une possibilité d'altération rénale, particulièrement en cas de lésions préexistantes.
Dans de rares cas, une hépatotoxicité avec modifications du bilan biologique hépatique a été observée.
Une aménorrhée ou une azoospermie sont possibles, voire définitives.
A très fortes doses, il existe un risque de cardiotoxicité (cardiomyopathie aiguë), potentialisé par irradiation antérieure de l'aire cardiaque ou l'utilisation d'anthracyclines et/ou de pentostatine.
En cas de leucopénies sévères, les mesures suivantes sont préconisées : administration d'antibiotiques et/ou d'antifongiques. Elles peuvent être prévenues par l'utilisation des facteurs de croissance granulocytaire.
Comme pour tout traitement cytostatique à doses cumulées élevées, le traitement par le cyclophosphamide peut être responsable de tumeurs secondaires. Le risque de développer une tumeur du tractus urinaire, comme un syndrome myélodysplasique pouvant évoluer en leucémie aiguë, est augmenté.

Autres effets indésirables :

- des cas de pneumopathies interstitielles, voire de fibroses pulmonaires, de pseudosécrétions inappropriées d'hormone antidiurétique (SIADH) ont pu être observées avec de fortes doses ;
- des réactions d'hypersensibilité au cyclophosphamide sont possibles, pouvant évoluer dans des cas isolés en état de choc ;
- des sensations vertigineuses associées à des troubles de la vision transitoires ont été rapportées.

**DC/GROSSESSE ET ALLAITEMENT** Contre-indiqué.

**DC/SURDOSAGE**

Il n'existe pas d'antidote spécifique du cyclophosphamide.
En cas de surdosage, il sera nécessaire d'adapter les soins en fonction de la toxicité constatée.
Au niveau rénal, l'uromitexan bloque le pouvoir irritant de l'acroléine, métabolite toxique pour la muqueuse vésicale formé au cours de la biotransformation du cyclophosphamide.
Le cyclophosphamide est dialysable.

**PP/PHARMACODYNAMIQUE**

Agent alkylant, moutarde à l'azote (L : antinéoplasique et immunomodulateur).
Agent alkylant bifonctionnel de type oxazaphosphorine appartenant à la famille des moutardes azotées agissant après transformation dans l'organisme.
Le cyclophosphamide agit par interaction directe sur l'ADN en formant des liaisons covalentes avec les substrats nucléophiles par l'intermédiaire de ses radicaux alcoyles. Ceci entraîne des modifications profondes chimiques ou enzymatiques de l'ADN ainsi que la formation de ponts alcoyles intrabrins ou interbrins, avec pour conséquence une inhibition de la transcription et de la réplication de l'ADN aboutissant à la destruction cellulaire. Cette action est cycle dépendante, elle respecte les cellules en Go.
Immunodépresseur.

**PP/PHARMACOCINETIQUE**

La molécule initiale est une prodrogue inactive. Elle est hydroxylée dans le foie par les microsomes hépatiques aboutissant essentiellement à la formation du 4-hydroxycyclophosphamide et à son tautomère l'aldo-cyclophosphamide puis à la moutarde phosphoramide (métabolite actif) et à l'acroléine (métabolite urotoxique).
Une voie métabolique accessoire conduit à une quantité négligeable des métabolites déchloréthylés.
Le temps moyen de demi-vie plasmatique du cyclophosphamide varie de 4 à 7 heures : il est plus court chez l'enfant (4 heures) que chez l'adulte (7 heures en moyenne).
Sous forme inchangée, il n'est pas lié de façon significative aux protéines plasmatiques (12 à 14 %) alors que ses métabolites le sont davantage (52 à 60 %).
La barrière hématoencéphalique est facilement traversée par le cyclophosphamide et un peu moins par ses métabolites (20 %) ce qui explique son intérêt dans le traitement de certaines tumeurs cérébrales. Son élimination à l'état inchangé ainsi que celle de ses métabolites est essentiellement urinaire.

**DP/CONDITIONS PARTICULIERES DE CONVERSATION**

A conserver à une température inférieure à 25 °C.
**- Après reconstitution :** 24 heures à une température comprise entre 2 °C et 8 °C et à l'abri de la lumière.
**- Après dilution dans le milieu de perfusion :** à utiliser immédiatement.

**DP/MODALITES DE MANIPULATION**

Utiliser de préférence en perfusion intraveineuse après dilution extemporanée dans 25 ml d'une solution de chlorure de sodium à 0,9 %.

**LISTE 1**

| AMM | 315 820.4 (1972/97 rév 13.06.2003) 1 fl 500 mg + solv. |
| --- | --- |
|  | 321 195.0 (1977/97 rév 13.06.2003) 1 fl 500 mg. |

| **PRIX :** | 5,12 € (1 fl 500 mg + solv). |
| --- | --- |

Remb Séc soc à 100 %. Collect.

Modèle hospitalier : Collect.

2-ZOPHREN® solution injectable ondansétron**: Laboratoire GlaxoSmithKline**

**FORMES et PRÉSENTATIONS**

*Solution injectable IV à 2 mg/ml :*
Ampoule de 2 ml, boîte unitaire.
Ampoule de 4 ml, boîte unitaire.
Modèle hospitalier : Boîtes de 5.

COMPOSITION

|  | *p ampoule* | |
| --- | --- | --- |
|  | *de 2 ml* | *de 4 ml* |
| Ondansétron (DCI) chlorhydrate dihydraté exprimé en ondansétron | 4 mg | 8 mg |

*Excipients :* acide citrique monohydraté, citrate de sodium, chlorure de sodium (9 mg/ml), eau ppi.

**DC/INDICATIONS**

- Prévention et traitement des nausées et vomissements aigus induits par la chimiothérapie cytotoxique moyennement à hautement émétisante et la radiothérapie hautement émétisante chez l'adulte.
- Prévention des nausées et vomissements aigus induits par la chimiothérapie cytotoxique moyennement à hautement émétisante chez l'enfant.
- Traitement des nausées et vomissements postopératoires chez l'adulte et l'enfant.

**DC/POSOLOGIE et MODE D'ADMINISTRATION**

**Posologie :**

**- Adulte à partir de 15 ans :**
**- Nausées et vomissements induits par les traitements cytotoxiques :**
La dose initiale habituelle est de 8 mg administrée soit en IV lente 30 minutes avant la chimiothérapie ou la radiothérapie, soit en comprimé ou sirop 2 heures avant la chimiothérapie moyennement émétisante ou la radiothérapie.
Dans certaines circonstances (utilisation de drogues cytotoxiques très émétisantes et/ou prescrites à très fortes doses, facteurs liés au patient tels que sujet jeune, de sexe féminin, ayant l'expérience de phénomènes émétiques lors de précédents traitements cytotoxiques...), une dose plus élevée (32 mg en IV lente sur plus de 15 minutes avant le début du traitement cytotoxique, ou 8 mg en IV lente suivis d'une perfusion de 1 mg/heure sur 24 heures ou 8 mg en IV lente suivis de 2 injections de 8 mg en IV lente à 4 heures d'intervalle) et/ou une association à une corticothérapie pourront être utilisées d'emblée.
**- Nausées et vomissements postopératoires :**
4 mg en IV lente.
**- Enfant de plus de 2 ans :**
**- Nausées et vomissements induits par les traitements cytotoxiques :**
La dose initiale est de 5 mg/m ^2^ administrée en IV lente juste avant la chimiothérapie.
**- Nausées et vomissements postopératoires :**
0,1 mg/kg en IV lente unique jusqu'à un maximum de 4 mg.
Coût d'une injection :

11,86 € (4 mg) ; 22,24 € (8 mg).
**- Sujet âgé :**
Chez le sujet âgé de plus de 65 ans, l'efficacité et la tolérance ont été semblables à ce qui est observé chez l'adulte plus jeune.
**- Insuffisant hépatique :**
Il est recommandé de ne pas dépasser une dose totale journalière de 8 mg chez ces patients.
**- Patient métaboliseur lent :**
Le métabolisme de la spartéine et de la débrisoquine au niveau du cytochrome P450 n'est pas modifié. Aucune adaptation posologique n'est donc nécessaire chez ce type de patients.

**Mode d'administration :**

**- Compatibilité avec les liquides de perfusion :**
En accord avec les bonnes pratiques pharmaceutiques, les solutions de perfusion doivent être préparées extemporanément. Toutefois, il a été prouvé que l'ondansétron injectable est stable 7 jours à température ambiante (au-dessous de 25 °C) sous éclairage fluorescent ou dans un réfrigérateur lorsqu'il est dilué dans les liquides de perfusion suivants :

- chlorure de sodium à 0,9 %,
- solution glucosée à 5 %,
- solution de mannitol à 10 %,
- solution de Ringer,
- solution de chlorure de potassium à 0,3 % + chlorure de sodium à 0,9 %,
- solution de chlorure de potassium à 0,3 % + solution glucosée à 5 %.

Des études de compatibilité ont été effectuées, les solutions d'ondansétron sont stables :

- dans les poches à perfusion en chlorure de polyvinyle,
- dans les nécessaires de perfusion en chlorure de polyvinyle,
- dans les poches de perfusion en polyéthylène,
- dans les flacons en verre de type 1.

Les solutions d'ondansétron dans une solution de chlorure de sodium à 0,9 % ou de glucose à 5 % administrées dans des seringues en polypropylène sont stables. On peut donc considérer que l'ondansétron injectable, dilué avec les autres liquides de perfusion compatibles, est stable dans des seringues en polypropylène.
Remarque : la préparation doit être faite dans des conditions aseptiques appropriées.

**- Compatibilité avec d'autres produits :**
L'ondansétron peut être administré en perfusion intraveineuse à partir d'une poche de perfusion ou d'une seringue électrique.

Les produits suivants peuvent être administrés simultanément au niveau d'une perfusion en Y pour peu que les concentrations d'ondansétron soient comprises entre 16 µg/ml et 160 µg/ml (c'est-à-dire 8 mg pour 500 ml et 8 mg pour 50 ml respectivement) :

- Cisplatine : à des concentrations n'excédant pas 0,48 mg/ml (c'est-à-dire 240 mg dans 500 ml) administrées sur 1 à 8 heures.
- 5-fluoro-uracile : à des concentrations n'excédant pas 0,8 mg/ml (c'est-à-dire 2,4 g dans 3 litres ou 400 mg dans 500 ml) administrées à un débit d'au moins 20 ml par heure (500 ml par 24 heures). Des concentrations plus élevées de 5-fluoro-uracile peuvent provoquer une précipitation d'ondansétron. Les perfusions de 5-fluoro-uracile peuvent contenir jusqu'à 0,045 % de chlorure de magnésium en addition des autres excipients compatibles.
- Carboplatine : à des concentrations allant de 0,18 mg/ml à 9,9 mg/ml (c'est-à-dire 90 mg dans 500 ml à 990 mg dans 100 ml), administrées sur une période de 10 minutes à une heure.
- Étoposide : à des concentrations allant de 0,14 mg/ml à 0,25 mg/ml (c'est-à-dire 72 mg dans 500 ml à 250 mg dans 1 litre), administrées sur une période de 30 minutes à une heure.
- Cyclophosphamide : à des doses de 100 mg à 1 g diluées avec de l'eau pour préparations injectables, 5 ml pour 100 mg de cyclophosphamide selon les recommandations du fabricant et administrées en bolus IV sur environ 5 minutes.
- Doxorubicine : à des doses de 10 à 100 mg reconstituées avec de l'eau pour préparations injectables, 5 ml pour 10 mg de doxorubicine selon les recommandations du fabricant et administrées en bolus IV sur environ 5 minutes.
- Ceftazidime : à des doses de 250 mg à 2000 mg diluées dans de l'eau pour préparations injectables selon les recommandations du fabricant (c'est-à-dire 2,5 ml pour 250 mg et 10 ml pour 2 g de ceftazidime) et administrées en bolus IV sur environ 5 minutes.

**DC/CONTRE-INDICATIONS**

Allergie à l'un des composants.

**DC/MISES EN GARDE et PRÉCAUTIONS D'EMPLOI**

**Mises en garde :**

Un bilan cardiovasculaire doit être effectué en cas de survenue de douleurs thoraciques et de syncope, ou de troubles du rythme cardiaque.
Prendre en compte le risque éventuel d'hypersensibilité croisée avec les autres antagonistes des récepteurs 5 HT_3_.

**Précautions d'emploi :**

En cas d'insuffisance hépatique sévère, les paramètres pharmacocinétiques de l'ondansétron sont significativement modifiés : réduction de la clairance plasmatique totale, augmentation de la demi-vie plasmatique.
L'ondansétron pouvant favoriser un syndrome occlusif, il convient de surveiller attentivement le transit des patients en cours de traitement (cf Effets indésirables).
En cas de régime désodé ou hyposodé, tenir compte de la teneur en sodium (cf Composition).

**DC/GROSSESSE et ALLAITEMENT**

**Grossesse :**

Les études effectuées chez l'animal n'ont pas mis en évidence d'effet tératogène de l'ondansétron.
En l'absence d'effet tératogène chez l'animal, un effet malformatif dans l'espèce humaine n'est pas attendu. En effet, à ce jour, les substances responsables de malformations dans l'espèce humaine se sont révélées tératogènes chez l'animal au cours d'études bien conduites sur deux espèces.
En clinique, il n'existe pas actuellement de données suffisamment pertinentes pour évaluer un éventuel effet malformatif ou fœtotoxique de l'ondansétron lorsqu'il est administré pendant la grossesse.
En conséquence, par mesure de précaution, il est préférable de ne pas utiliser l'ondansétron pendant la grossesse.

**Allaitement :**

En cas d'allaitement ou de désir d'allaitement et compte tenu du passage de l'ondansétron dans le lait maternel, l'utilisation de ce produit est déconseillée.

**DC/EFFETS INDÉSIRABLES**

Les effets indésirables suivants peuvent survenir :

- céphalées, bouffées de chaleur ou flush, hoquets,
- rarement, anomalies biologiques hépatiques,
- constipation pouvant, dans de rares cas, se compliquer d'iléus ou d'occlusion intestinale, en particulier chez des patients présentant des facteurs de risque associés : ralentisseurs du transit, antécédent de chirurgie digestive...,
- hypotension, douleurs thoraciques avec ou sans décalage du segment ST, troubles du rythme et bradycardie,
- réactions extrapyramidales telles que crises oculogyres, dystonies sans séquelles cliniques ou convulsions,
- réactions allergiques immédiates, quelquefois sévères, incluant des réactions anaphylactiques,
- troubles visuels transitoires et vertiges au cours des injections IV rapides,
- possibilité de réactions au point d'injection ou le long de la veine perfusée (érythème, urticaire, prurit, douleur) et rares cas de veinites.

**DC/SURDOSAGE**

Un surdosage en ondansétron peut entraîner les effets indésirables déjà mentionnés dans la rubrique Effets indésirables. Il n'existe pas d'antidote spécifique de l'ondansétron. Par conséquent, en cas de surdosage, seule une thérapeutique symptomatique appropriée sera instaurée.

**PP/PHARMACODYNAMIQUE**

Antagoniste de la sérotonine (A04AA01 : appareil digestif et métabolisme).
L'ondansétron est un antagoniste des récepteurs 5HT_3_ à la sérotonine, impliqués dans les phénomènes de réflexe émétique.
L'administration d'ondansétron ne modifie pas les taux sériques de prolactine.

**PP/PHARMACOCINETIQUE**

L'ondansétron est métabolisé et les métabolites sont excrétés dans les fèces et l'urine.
Après administration IV, le pic sérique est atteint en environ 15 minutes. La concentration au pic est de 95,6 ng/ml.
La demi-vie d'élimination est d'environ 3 heures ; cependant, elle peut être prolongée jusqu'à 5 heures chez le sujet âgé.
Liaison aux protéines : 70 à 76 %.

**DP/COMPATIBILITES**

L'ondansétron injectable est incompatible avec les solutions bicarbonatées.
Il ne doit pas être administré dans la même seringue ou la même perfusion que d'autres médicaments.
Il ne doit être administré qu'avec les liquides de perfusion recommandés.
Il ne doit pas être autoclavé.

**DP/CONDITIONS PARTICULIERES DE CONSERVATION**

Ce médicament doit être conservé à une température inférieure à 25 °C et à l'abri de la lumière dans son étui d'origine.

**LISTE1**

| AMM | 335 392.8 (1992 rév 09.05.2001) 1 amp 4 mg. |
| --- | --- |
|  | 556 831.4 (1990 rév 09.05.2001) 5 amp 4 mg. |
|  | 335 393.4 (1992 rév 09.05.2001) 1 amp 8 mg. |
|  | 556 832.0 (1990 rév 09.05.2001) 5 amp 8 mg. Mis sur le marché en 1990. |

| **PRIX :** | 11,86 € (1 amp à 4 mg). |
| --- | --- |
|  | 22,24 € (1 amp à 8 mg). |

Remb Séc Soc à 65 % selon la procédure des médicaments d'exception (prescription en conformité avec la fiche d'information thérapeutique). Collect.

3- SOLU-MÉDROL® 20 mg, 40 mg, 120 mg, méthylprednisolone,  **PHARMACIA SAS**

## **FORMES et PRÉSENTATIONS**

*Poudre et solvant pour solution injectable à 20 mg, 40 mg et 120 mg :*
Flacon de lyophilisat + ampoule autocassable de solvant (2 ml), boîte unitaire.
Modèle hospitalier : Boîtes de 10 flacons.

COMPOSITION

| Lyophilisat : | p flacon |
| --- | --- |
| Méthylprednisolone (DCI) hémisuccinate exprimé en méthylprednisolone | 20 mg |
| ou | 40 mg |
| ou | 120 mg |

| Solvant : | p ampoule |
| --- | --- |
| Eau pour préparations injectables | 2 ml |

*Excipients :* Solu-Médrol 20 mg et 40 mg : phosphate monosodique anhydre, phosphate disodique anhydre, lactose. Solu-Médrol 120 mg : phosphate monosodique anhydre, phosphate disodique anhydre, solution d'hydroxyde de sodium à 10 % qsp pH 7,5 à 7,7.

**DC/INDICATIONS**

Celles de la corticothérapie générale per os, lorsque la voie parentérale est nécessaire en cas d'impossibilité de la voie orale (vomissements, aspiration gastrique, troubles de la conscience).

Les affections nécessitant un effet thérapeutique rapide :

- Allergiques : œdème de Quincke sévère en complément des antihistaminiques ; choc anaphylactique en complément de l'adrénaline.
- Infectieuses : fièvre thyphoïde sévère, en particulier avec confusion mentale, choc, coma ; laryngite striduleuse (laryngite sous-glottique) chez l'enfant.
- Neurologiques : œdème cérébral des tumeurs, de l'hématome sous-dural et œdème cérébral lié à un abcès à toxoplasme.
- ORL : dyspnée laryngée.

**DC/POSOLOGIE et MODE D'ADMINISTRATION**

Cette spécialité n'est pas adaptée à l'administration par voie inhalée par nébulisateur.
Équivalence anti-inflammatoire (équipotence) : 5 mg de prednisone = 4 mg de méthylprednisolone.
Injections intraveineuse ou intramusculaire.
La posologie est variable en fonction du diagnostic, de la sévérité de l'affection, du pronostic, de la réponse du patient et de la tolérance au traitement.
**- Solu-Médrol 20 mg et 40 mg :**
Après mélange, la solution obtenue peut être administrée directement par voie IM profonde, par voie IV lente ou par perfusion IV après dilution dans une solution de chlorure de sodium ou de glucose.

- Adulte : 20 à 60 mg par jour. Cette dose peut être renouvelée 2 à 3 fois par 24 heures, si nécessaire. Coût du traitement journalier : 1,84 à 5,52 € (Solu-Médrol 20 mg).
- Enfant : 1 à 3 mg/kg/jour. Coût du traitement journalier : 0,092 à 0,28 €/kg (Solu-Médrol 20 mg).

**- Solu-Médrol 120 mg :**
Réservé à l'adulte.

Après mélange, la solution obtenue est administrée par voie IV :

- soit directement en injection lente,
- soit par perfusion IV après dilution dans une solution isotonique de chlorure de sodium ou de glucose.

Lorsque la voie IV ne peut être utilisée, l'administration pourra être effectuée par voie IM profonde sous asepsie rigoureuse.
La posologie est de 120 mg par jour. Dans des situations très exceptionnelles, cette posologie pourra être répétée.
Coût du traitement journalier : 5,17 €.

**DC/CONTRE-INDICATIONS**

**Absolues :**

- Tout état infectieux à l'exclusion des indications spécifiées (cf Indications).
- Certaines viroses en évolution (notamment hépatites, herpès, varicelle, zona).
- États psychotiques encore non contrôlés par un traitement.
- Vaccins vivants.
- Hypersensibilité à l'un des constituants.
- Troubles de l'hémostase ou traitement anticoagulant en cours, en cas d'injection intramusculaire.

Il n'existe toutefois aucune contre-indication absolue pour une corticothérapie d'indication vitale.

**Relatives :**

- Médicaments non antiarythmiques donnant des torsades de pointes (cf Interactions).

**DC/MISES EN GARDE et PRÉCAUTIONS D'EMPLOI**

**Mises en garde :**

De rares cas de réactions pseudo-anaphylactiques étant survenus chez des patients traités par une corticothérapie parentérale, une attention particulière sera portée avant toute administration chez des sujets présentant un terrain atopique.
En cas d'ulcère gastroduodénal, la corticothérapie n'est pas contre-indiquée si un traitement anti-ulcéreux est associé.
En cas d'antécédents ulcéreux, la corticothérapie peut être prescrite, avec une surveillance clinique et au besoin après fibroscopie.
La corticothérapie peut favoriser la survenue de diverses complications infectieuses dues notamment à des bactéries, des levures et des parasites. La survenue d'une anguillulose maligne est un risque important. Tous les sujets venant d'une zone d'endémie (régions tropicale, subtropicale, sud de l'Europe) doivent avoir un examen parasitologique des selles et un traitement éradicateur systématique avant la corticothérapie.
Les signes évolutifs d'une infection peuvent être masqués par la corticothérapie.
Il importe, avant la mise en route du traitement, d'écarter toute possibilité de foyer viscéral, notamment tuberculeux, et de surveiller, en cours de traitement, l'apparition de pathologies infectieuses.
En cas de tuberculose ancienne, un traitement prophylactique antituberculeux est nécessaire s'il existe des séquelles radiologiques importantes et si l'on ne peut s'assurer qu'un traitement bien conduit de 6 mois par la rifampicine a été donné.
L'emploi des corticoïdes nécessite une surveillance particulièrement adaptée, notamment chez les sujets âgés et en cas de colites ulcéreuses (risque de perforation), diverticulites, anastomoses intestinales récentes, insuffisance rénale, insuffisance hépatique, ostéoporose, myasthénie grave.
L'attention est attirée chez les sportifs, cette spécialité contenant un principe actif pouvant induire une réaction positive des tests pratiqués lors des contrôles antidopage.

**Précautions d'emploi :**

Un traitement par voie orale sera institué en relais dès que possible.
Une rétention hydrosodée est habituelle, responsable en partie d'une élévation éventuelle de la pression artérielle. L'apport sodé sera réduit.
La supplémentation potassique n'est justifiée que pour des traitements à fortes doses, prescrits pendant une longue durée ou en cas de risque de troubles du rythme ou d'associations à un traitement hypokaliémiant.
Lorsque la corticothérapie est indispensable, le diabète et l'hypertension artérielle ne sont pas des contre-indications mais le traitement peut entraîner leur déséquilibre. Il convient de réévaluer leur prise en charge.
Les patients doivent éviter le contact avec des sujets atteints de varicelle ou de rougeole.

**DC/INTERACTIONS**

**Interactions médicamenteuses :**

*Associations déconseillées :*

- Médicaments donnant des torsades de pointes : astémizole, bépridil, érythromycine IV, halofantrine, pentamidine, sparfloxacine, sultopride, terfénadine, vincamine. Torsades de pointes (l'hypokaliémie est un facteur favorisant, de même que la bradycardie et un espace QT long préexistant). Utiliser des substances ne présentant pas l'inconvénient d'entraîner des torsades de pointes en cas d'hypokaliémie.

*Associations nécessitant des précautions d'emploi :*

- Acide acétylsalicylique par voie générale (et par extrapolation, autres salicylés) : diminution de la salicylémie pendant le traitement par les corticoïdes et risque de surdosage salicylé après son arrêt (augmentation de l'élimination des salicylés par les corticoïdes).
  Adapter les doses de salicylés pendant l'association et après l'arrêt du traitement par les corticoïdes.
- Antiarythmiques donnant des torsades de pointes (amiodarone, disopyramide, quinidiniques, sotalol) : torsades de pointes (l'hypokaliémie est un facteur favorisant, de même que la bradycardie et un espace QT long préexistant). Prévention de l'hypokaliémie et, si besoin, correction ; surveillance de l'espace QT. En cas de torsades, ne pas administrer d'antiarythmique (entraînement électrosystolique).
- Anticoagulants oraux : impact éventuel de la corticothérapie sur le métabolisme de l'anticoagulant oral et sur celui des facteurs de la coagulation. Risque hémorragique propre à la corticothérapie (muqueuse digestive, fragilité vasculaire) à fortes doses ou en traitement prolongé supérieur à 10 jours. Lorsque l'association est justifiée, renforcer la surveillance : contrôle biologique au 8 ^e^ jour, puis tous les 15 jours pendant la corticothérapie et après son arrêt.
- Autres hypokaliémiants : diurétiques hypokaliémiants (seuls ou associés), laxatifs stimulants, amphotéricine B (voie IV) : risque accru d'hypokaliémie (effet additif). Surveillance de la kaliémie, avec, si besoin, correction à prendre particulièrement en compte en cas de thérapeutique digitalique.
- Digitaliques : hypokaliémie favorisant les effets toxiques des digitaliques. Surveillance de la kaliémie et, s'il y a lieu, ECG.
- Héparines (voie parentérale) : aggravation par l'héparine du risque hémorragique propre à la corticothérapie (muqueuse digestive, fragilité vasculaire) à fortes doses ou en traitement prolongé supérieur à 10 jours. L'association doit être justifiée, renforcer la surveillance.
- Inducteurs enzymatiques : anticonvulsivants (carbamazépine, phénobarbital, phénytoïne, primidone), rifampicine. Diminution des concentrations plasmatiques et de l'efficacité des corticoïdes par augmentation de leur métabolisme hépatique. Les conséquences sont particulièrement importantes chez les addisoniens et en cas de transplantation. Surveillance clinique et biologique, adaptation de la posologie des corticoïdes pendant l'association et après arrêt de l'inducteur enzymatique.
- Insuline, metformine, sulfamides hypoglycémiants : élévation de la glycémie avec parfois cétose (diminution de la tolérance aux glucides par les corticoïdes). Prévenir le patient et renforcer l'autosurveillance sanguine et urinaire, surtout en début de traitement. Adapter éventuellement la posologie de l'antidiabétique pendant le traitement par les corticoïdes et après son arrêt.
- Isoniazide (décrit pour la prednisolone) : diminution des concentrations plasmatiques de l'isoniazide. Mécanisme invoqué : augmentation du métabolisme hépatique de l'isoniazide et diminution de celui des glucocorticoïdes. Surveillance clinique et biologique.

*Associations à prendre en compte :*

- Antihypertenseurs : diminution de l'effet antihypertenseur (rétention hydrosodée des corticoïdes).
- Ciclosporine : augmentation possible des concentrations plasmatiques de ciclosporine et de la créatinémie. Mécanisme invoqué : diminution de l'élimination hépatique de la ciclosporine.
- Interféron alpha : risque d'inhibition de l'action de l'interféron.
- Vaccins vivants atténués : risque de maladie généralisée éventuellement mortelle. Ce risque est majoré chez les sujets déjà immunodéprimés par la maladie sous-jacente.
  Utiliser un vaccin inactivé lorsqu'il existe (poliomyélite).

**DC/GROSSESSE et ALLAITEMENT**

**Grossesse :**

Chez l'animal, l'expérimentation met en évidence un effet tératogène variable selon les espèces.
Dans l'espèce humaine, il existe un passage transplacentaire. Cependant, les études épidémiologiques n'ont décelé aucun risque malformatif lié à la prise de corticoïdes lors du premier trimestre.
Lors de maladies chroniques, nécessitant un traitement tout au long de la grossesse, un léger retard de croissance intra-utérin est possible. Une insuffisance surrénale néonatale a été exceptionnellement observée après corticothérapie à doses élevées. Il est justifié d'observer une période de surveillance clinique (poids, diurèse) et biologique du nouveau-né.
En conséquence, les corticoïdes peuvent être prescrits pendant la grossesse si besoin.

**Allaitement :**

En cas de traitement à doses importantes et de façon chronique, l'allaitement est déconseillé.

**DC/EFFETS INDÉSIRABLES**

- De rares cas de réactions anaphylactiques ont pu être rapportés chez des patients traités par des corticostéroïdes par voie parentérale (cf Mises en garde/Précautions d'emploi). Des troubles du rythme cardiaque ont également été décrits, liés à l'administration intraveineuse.
- Désordres hydroélectrolytiques : hypokaliémie, alcalose métabolique, rétention hydrosodée, hypertension artérielle, insuffisance cardiaque congestive.
- Troubles endocriniens et métaboliques : syndrome de Cushing iatrogène, inertie de la sécrétion d'ACTH, atrophie corticosurrénalienne parfois définitive, diminution de la tolérance au glucose, révélation d'un diabète latent, arrêt de la croissance chez l'enfant, irrégularités menstruelles.
- Troubles musculosquelettiques : atrophie musculaire précédée par une faiblesse musculaire (augmentation du catabolisme protidique), ostéoporose, fractures pathologiques, en particulier tassements vertébraux, ostéonécrose aseptique des têtes fémorales.
- Troubles digestifs : ulcères gastroduodénaux, ulcérations du grêle, perforations et hémorragies digestives ; des pancréatites aiguës ont été signalées, surtout chez l'enfant.
- Troubles cutanés : acné, purpura, ecchymoses, hypertrichose, retard de cicatrisation.
- Troubles neuropsychiques :
  - fréquemment : euphorie, insomnie, excitation ;
  - rarement : accès d'allure maniaque, états confusionnels ou confuso-oniriques, convulsions ;
  - état dépressif à l'arrêt du traitement.
- Troubles oculaires : certaines formes de glaucome et de cataracte.

**PP/PHARMACODYNAMIE**

Glucocorticoïde, usage systémique (H : hormones non sexuelles ; D : dermatologie ; M : système locomoteur ; S : organes sensoriels).
Les glucocorticoïdes physiologiques (cortisone et hydrocortisone) sont des hormones métaboliques essentielles. Les corticoïdes synthétiques incluant la méthylprednisolone sont utilisés principalement pour leur effet anti-inflammatoire. A forte dose, ils diminuent la réponse immunitaire. Leur effet métabolique et de rétention sodée est moindre que celui de l'hydrocortisone.

**PP/PHARMACOCINÉTIQUE**

Diffusion rapide ; demi-vie : 3 h 30.
L'élimination est à la fois urinaire et biliaire.
Passage dans le lait et à travers le placenta.

**DP/CONDITIONS PARTICULIÈRES DE CONSERVATION**

*Après reconstitution :* la solution doit être utilisée extemporanément.

LISTE I

| AMM | 309 717.0 (1991 rév 1998) 20 mg, fl + solv. |
| --- | --- |
|  | 309 718.7 (1991 rév 1998) 40 mg, fl + solv. |
|  | 315 528.1 (1991 rév 1998) 120 mg, fl + solv. |
|  | 558 648.2 (1995 rév 1998) 20 mg, 10 fl. |
|  | 558 650.7 (1995 rév 1998) 40 mg, 10 fl. |
|  | 558 653.6 (1995 rév 1998) 120 mg, 10 fl. Mis sur le marché en 1962 et 1969 (120 mg). |

| **PRIX :** | 1,84 € (flacon de 20 mg + solvant). |
| --- | --- |
|  | 2,50 € (flacon de 40 mg + solvant). |
|  | 5,17 € (flacon de 120 mg + solvant). |

Remb Séc soc à 65 %. Collect.
Collect (boîtes de 10 flacons).

**ANNEXE 3 :NOTICE D’INFORMATION**

Le Dr …………………exerçant dans le service …………………….. ……………………………
de l’Hôpital ………………………. ………………………..vous propose de participer au protocole :

**ESSAI THERAPEUTIQUE RANDOMISE MULTICENTRIQUE**

**EN DOUBLE INSU EN GROUPES PARALLELES**

**DU CYCLOPHOSPHAMIDE INTRAVEINEUX VERSUS METHYLPREDNISOLONE DANS LE TRAITEMENT DE FOND DES FORMES PROGRESSIVES SECONDAIRES RECENTES DE SCLEROSE EN PLAQUES : ETUDE P.R.OM.E.S.S**

**Promoteur : Centre Hospitalier Universitaire de Bordeaux**

### Investigateur Coordonnateur : Pr. Bruno Brochet

NOTE D'INFORMATION AU PATIENT

Version protocole du 04/04/2005

*Recherche avec bénéfice individuel direct*

***Merci de lire attentivement ce document d’information, il nous servira de base pour les discussions ultérieures. Vous trouverez un glossaire des termes médicaux spécifiques à la fin de ce document pour une meilleure compréhension de cette étude****.*

**Partie 1 - Pourquoi cette étude ?**

Vous présentez une sclérose en plaques qui est une maladie inflammatoire du système nerveux. Cette maladie se présente sous différentes formes évolutives et les traitements de ces différentes formes ne sont pas identiques.
La forme actuelle de votre maladie est ce qu’on appelle la forme secondairement progressive ce qui veut dire que l’aggravation que vous avez présenté l’année dernière n’était pas uniquement (ou pas du tout) due à des séquelles de nouvelles poussées mais à une aggravation progressive en particulier des troubles de la marche.
Actuellement il existe plusieurs traitements validés pour les formes rémittentes (c'est-à-dire uniquement par poussées) de sclérose en plaques. Les traitements disponibles à la phase progressive sont moins nombreux et d’efficacité inconstante. Deux interférons disposent d’une autorisation de mise sur le marché, le bétaféron ® pour les patients ayant une forme secondairement progressive et le rebif ® pour les patients ayant une forme secondairement progressive avec poussées surajoutées. Ces produits peuvent stabiliser la maladie d’un certain pourcentage de patients dans cette situation mais ne sont pas utiles chez ceux qui ont débuté leur aggravation progressive alors qu’ils étaient déjà sous interféron auparavant. Ils ne sont pas efficaces chez tous les patients ayant une forme secondairement progressive.
Un autre médicament l’Elsep ® (mitoxantrone) a reçu une autorisation de mise sur le marché pour les patients ayant une forme agressive de SEP définie par certains critères cliniques et d’IRM. La majorité des patients ayant une forme secondairement progressive ne réunit pas ces critères et ne relève donc pas de ce traitement mais dans le cas où vous réuniriez les critères d’indication d’Elsep ® votre neurologue vous en informera avant votre décision de participer ou non au présent protocole.

Depuis plusieurs années plusieurs centres spécialisés dans la prise en charge de la SEP utilisent le cyclophosphamide (endoxan®) par cures de perfusions mensuelles dans cette indication.

Ce médicament est un médicament immunosuppresseur qui agit sur les globules blancs responsables de l’inflammation présente dans cette maladie. Aux doses utilisées son action est en fait essentiellement immunomodulatrice c'est-à-dire qu’il diminue l’inflammation sans diminuer de façon importante le nombre de globules blancs. Deux études dites « ouvertes », c'est-à-dire sans groupe contrôle, ont été publiées ces dernières années sur un total de 585 patients ayant une sclérose en plaques montrant des résultats très encourageants avec ce traitement puisque dans ces études 73 à 78% des patients ne se sont pas aggravés. Aucune étude contrôlée n’a à ce jour été réalisée à ces doses dans cette indication.
Des études contrôlées avaient été réalisées en utilisant des doses et des schémas d’administration différents il y a quelques années avec des résultats contradictoires mais ces études utilisaient un traitement sur quelques jours sans traitement d’entretien. Des études pilotes ont été réalisées ces dernières années dans d’autres formes de la SEP avec ce médicament avec des résultats positifs. Il est donc d’une grande importance de réaliser une étude contrôlée pour déterminer l’efficacité de ce traitement.

**Quel est l'objectif de cette étude ?**

**L'objectif principal de cette étude est donc de comparer l’efficacité du traitement par du cyclophosphamide (endoxan®) par rapport à un traitement par méthylprednisolone (Solumédrol®) administrés par voie intraveineuse.** Ce protocole a été élaboré, relu et approuvé par les experts du comité scientifique de l’étude. Il a été soumis et approuvé par les experts du programme hospitalier de recherche clinique national.

**Partie 2 - Comment va se dérouler cette étude ?**

La durée prévue de l’étude est de 3 ans soit un an de recrutement des patients y participant et deux ans de traitement au sein de l’étude. 6 mois supplémentaires seront ensuite nécessaires pour l’analyse des résultats. La participation de chaque patient est de 96 semaines (environ 2 ans). Il est prévu de faire participer 360 patients à cette étude dans plusieurs services hospitaliers de neurologie en France.
Tous les patients acceptant de participer à cette étude devront avoir entre 18 et 65 ans inclus et  une forme secondairement progressive de SEP dont la phase d’aggravation progressive ne doit pas avoir dépassé 3 ans avec certains critères, en particulier l’aggravation récente doit correspondre à une réduction du périmètre de marche (distance de marche sans arrêt) dans les 12 derniers mois non attribuable à des séquelles de poussées.
Ne peuvent participer à l’étude les patients ayant des antécédents de cystite hémorragique, d’allergie connue au cyclophosphamide (endoxan®), aux corticoïdes et en particulier au méthylprednisolone (Solumédrol ®)  ou ayant une maladie infectieuse en cours, ainsi que les patients porteurs d’une cathétérisation permanente de la vessie (sondage urinaire à demeure). Les femmes enceintes ou allaitantes ne peuvent participer à l’étude et une contraception efficace doit être envisagée pour les femmes en âge de procréer (voir plus loin). Les patients ayant déjà été traités antérieurement par du cyclophosphamide (endoxan®), une irradiation totale lymphoïde, un anticorps monoclonal anti-CD4 ou anti-CD52 ou anti-VLA4, de la mitoxantrone, de la cladribine ou la cyclosporine A ne peuvent pas participer à l’étude.
En cas de traitement antérieur par interféron bêta, ou méthotrexate, un intervalle d’au moins un mois doit être respecté entre l’arrêt de ce traitement et le début du traitement à l’étude.
Si vous recevez un traitement contre l’hypercholestérolémie, avertissez en votre neurologue, certaines restrictions pouvant intervenir.
Les patients participant à l’étude seront répartis en deux groupes par tirage au sort sans qu’eux-mêmes ou les médecins les suivant ne sache dans quel groupe ils auront été affecté (double-insu). Les patients du premier groupe recevront le traitement par cyclophosphamide (endoxan ®) et les patients du second groupe recevront de la méthylprednisolone (solumédrol ®) au même rythme. Aucun patient ne reçoit de placebo. Le traitement de comparaison est un traitement corticoïde qui a été choisi pour ne pas exposer le groupe de comparaison à un placebo. Le solumédrol ® est le traitement habituel des poussées de SEP sous formes de perfusions pendant plusieurs jours consécutifs. Sous la forme de perfusions tous les deux mois il a fait l’objet d’une étude dans le traitement des formes secondairement progressive de SEP en le comparant pendant 2 ans à de faibles doses de corticoïdes chez 109 patients. Une tendance favorable mais non significative a été observée mais l’analyse de l’évolution du handicap a montré un effet significatif du traitement. Le traitement administré dans ce groupe est donc probablement supérieur à l’abstention thérapeutique.
Le traitement par cyclophosphosphamide (endoxan®) ou par méthylprednisolone (solumédrol ®) est injecté dans une perfusion intraveineuse sur 3 heures toutes les 4 semaines pendant 44 semaines puis toutes les 8 semaines jusqu’à la semaine 92.
Le bénéfice attendu est une supériorité du traitement par cyclophosphosphamide (endoxan®) par rapport au traitement par méthylprednisolone (solumédrol ®) pour empêcher l’aggravation du handicap après deux ans de traitement.

Cet objectif sera analysé en utilisant une échelle de mesure clinique reposant sur l’examen neurologique et la mesure du périmètre de marche appelée EDSS et fréquemment utilisé dans le suivi de la SEP et dans les essais thérapeutiques concernant cette maladie. La constatation d’une aggravation de ce score confirmée à deux examens successifs à 4 mois d’écart servira à définir cette aggravation du handicap.
En cas d’aggravation confirmée du handicap le patient en sera informé et il pourra rediscuter les choix thérapeutiques éventuels avec son neurologue. Il lui sera proposé également de poursuivre l’étude jusqu’à son terme s’il le souhaite.
En cas de suspicion de poussée vous devez contacter dès que possible le neurologue qui vous suit dans l’étude. Le neurologue vous demandera de venir au centre pour une visite non programmée au plus tard 7 jours après votre appel. Si nécessaire il vous proposera le traitement nécessaire pour la poussée (solumédrol ®).

**Partie 3 - Quels sont les inconvénients possibles ?**

La participation à cette étude impose certaines contraintes. Les traitements sont administrés dans des perfusions intraveineuses en milieu hospitalier. La durée totale de perfusion est de 8 heures.
Une hospitalisation de jour est donc nécessaire pour administrer ces perfusions toutes les 4 semaines pendant 44 semaines puis toutes les 8 semaines pendant 44 semaines.
Une à deux visites interviendront avant la première perfusion et une visite 4 semaines après la dernière perfusion. Au cours des visites ou hospitalisations deux neurologues seront chargés de vous suivre. L’un (neurologue évaluateur), le Dr …………………... sera chargé de l’examen neurologique, de la mesure du périmètre de marche, et de tests neurologiques. Vous ne devrez lui communiquer aucune information sur les éventuels effets indésirables du traitement. Il pourra se faire aider d’une infirmière ou d’un assistant de recherche. L’autre (appelé neurologue traitant), le Dr ……………………….. sera chargé de votre suivi médical et recueillera tous les symptômes et signes éventuellement imputables au traitement reçu. C’est lui que vous devez contacter en cas de poussée.

Des prises de sang seront réalisées régulièrement au cours de l’étude afin de surveiller en particulier la numération des globules sanguins, le ionogramme sanguin, la glycémie, créatininémie, les transaminases et pour les femmes en âge de procréer des test de grossesse). Une prise de sang sera faite avant le début du traitement et une entre chaque hospitalisation. Un examen cytobactériologique des urines sera effectué en même temps. Avant chaque perfusion un électrocardiogramme sera réalisé et une analyse d’urines sur bandelettes.

Avant le début de l’étude, une intradermoréaction à la tuberculine (IDR), des radiographies simples des poumons, des sinus et des dents et une échographie de la vessie seront réalisés.
Cette étude vous est proposée parce que les médecins qui vous soignent considèrent que les avantages liés au traitement à l’essai sont supérieurs aux inconvénients. Néanmoins, un certain nombre d’effets indésirables ont été décrits avec le cyclophosphamide (endoxan®) et nécessite une surveillance attentive. Cependant ces effets sont rares aux doses utilisées. Dans l’étude rétrospective française portant sur 490 patients ayant une SEP progressive et traités à la même dose que dans cette étude, seuls 4,5% des patients ont présenté un effet secondaire sévère, nausées et vomissements, leucopénie (baisse des globules blancs importante), infections, quelques pertes de cheveux réversibles et augmentation des enzymes hépatiques. Seul un patient a du interrompre ce traitement du fait de ces effets secondaires (augmentation prolongée des enzymes hépatiques).

- Effets hématologiques : une baisse des globules blancs et plus rarement des plaquettes est fréquemment observée après la perfusion. La baisse des lymphocytes est souhaitée et reflète l’efficacité du traitement. Ces baisses sont réversibles rapidement et spontanément mais dans certains cas peuvent nécessiter la diminution de la dose voire l'arrêt du traitement. Cette baisse des globules blancs est le plus souvent modérée et sans conséquences. Elle n’impose pas de contraintes particulières en dehors de la réalisation des numérations sanguines. Dans de rares cas, cette baisse des globules blancs est plus importante et pourrait favoriser une infection. En cas de fièvre ou de fatigue inexpliquée brutale il faut prévenir votre médecin pour qu’un contrôle de la numération sanguine soit effectué. Un traitement antibiotique pourrait être indiqué.
De façon exceptionnelle, des leucémies et des tumeurs ont été décrites chez des patients traités par cyclophosphamide (endoxan®) après des traitements prolongés et à des doses très supérieures à celles utilisées dans la sclérose en plaques. Le risque, s’il existe, paraît très faible. Un risque de cancer de la vessie a été rapporté chez les patients ayant une SEP et ayant une sonde urinaire à demeure (ce qui constitue un critère de non inclusion) et ayant reçu des doses cumulées de plus de 50 grammes de cyclophosphamide (endoxan®) ce qui est très supérieur aux doses proposées. Les doses cumulées maximales seront dans cette étude inférieures à 25 grammes.

- Effets allergiques : comme pour tout médicament des épisodes d’allergie ont été rarement signalés notamment des érythèmes (rougeurs), exceptionnellement, des réactions anaphylactiques.

- Effets gastro-intestinaux : des nausées et des vomissements sont possibles lors des perfusions surtout à des doses plus élevées. Cependant pour les éviter un traitement par un *antiémétique* (zophren ®) sera administré en même temps que l’endoxan®. Des inflammations de la cavité buccale ont été observées à des doses supérieures.

- Grossesse et vie hormonale : le traitement est contre-indiqué pendant la grossesse en raison du risque de fausse couche spontanée et du risque de malformation possible pour le bébé. Il est également contre-indiqué durant l’allaitement. Des modifications du rythme menstruel et des aménorrhées ont également été décrites chez la femme. Les femmes en âge de procréer doivent accepter de recourir à une contraception efficace durant toute la durée de l’étude et pendant les 3 mois après l’arrêt de celle-ci et doivent avoir un test de grossesse négatif à l’inclusion. De rares azoospermies ont été observées chez l’homme. Une conservation de votre sperme peut être envisagée avant le début du traitement pour un éventuel projet parental par le CECOS (centre d’étude pour la conservation des œufs et de sperme humain). Les hommes susceptibles de procréer doivent accepter de recourir à une contraception efficace (préservatif ou contraception efficace du partenaire) durant toute la durée de l’étude.
- Vessie : afin d’éviter les rares inflammations de la vessie et cystites hémorragiques observées surtout à fortes doses, une perfusion de sérum physiologique est administrée pour assurer une diurèse suffisante.

- Peaux et cheveux : Une perte de quelques cheveux est possible au cours du traitement mais les rares alopécies sont observées à plus fortes doses. Exceptionnellement, ont été rapportés : une cardiomyopathie aiguë, une fibrose pulmonaire.

Les perfusions de méthylprednisolone (solumédrol ®) seront administrées aux doses habituellement utilisées dans la SEP qui sont le plus souvent très bien tolérées mais peuvent occasionnellement exposer à des effets indésirables :

De rares cas de réactions anaphylactiques (allergique) ont pu être rapportés chez des patients traités par des corticostéroïdes par voie injectable. Des troubles du rythme cardiaque ont également été décrits, liés à l'administration intraveineuse, en particulier si la vitesse de la perfusion est trop rapide.

Les effets secondaires habituels des corticoïdes au long cours sont rarement observés : Désordres hydroélectrolytiques, rétention hydrosodée, hypertension artérielle, insuffisance cardiaque, troubles endocriniens et métaboliques , troubles musculosquelettiques, ostéoporose, troubles digestifs, ulcères gastroduodénaux, ulcérations du grêle, perforations et hémorragies, troubles cutanés,  acné.

Des troubles neuropsychiques à type d’euphorie, insomnie, excitation peuvent survenir et plus rarement des accès d'allure maniaque,
En cas d’arrêt ou de sortie d’étude prématurée votre neurologue vous proposera le traitement de votre maladie le plus adapté à votre cas en fonction des connaissances médicales du moment.
Le neurologue traitant prendra le temps nécessaire pour vous expliquer les effets secondaires du traitement et la façon de les prévenir. Il restera à votre disposition au cours de l’essai. N’hésitez pas à lui signaler tout effet attendu ou inattendu.

**Partie 4 - Que vous demandera-t-on ?**

Si, après avoir lu cette note d’information et avoir discuté de l’étude avec votre médecin, vous décidez d’y participer, vous devrez signer un formulaire confirmant votre accord de participation. Vous serez alors « pré-inclus » et on effectuera une évaluation neurologique et des tests biologiques pour voir si vous pouvez être définitivement inclus dans l’étude. Si vos tests ne correspondent pas aux règles d’inclusion dans l’étude, votre participation à l’étude s’arrêtera et vous déciderez avec votre médecin du meilleur traitement à recevoir dans votre cas.
Si vous réunissez les critères des tests complémentaires seront réalisés (radiographies, échographie, intradermo-réaction IDR) et chez les femmes, un test de grossesse sera effectué. Vous serez ensuite convoqués pour la première perfusion. Le tableau vous décrit le calendrier des visites prévues.

A la fin de l’étude, votre neurologue vous proposera le traitement de votre maladie le plus adapté à votre cas en fonction des connaissances médicales du moment.

**Partie 5 - Que se passera-t-il en cas de survenue d’effets indésirables ?**

En cas d’effet secondaire peu important en particulier biologique la dose cyclophosphamide (endoxan®) sera ajustée par le pharmacien de l’hôpital. En cas d’effet secondaire grave (par exemple, baisse importante des globules blancs), le traitement sera provisoirement arrêté jusqu’à ce que la prise de sang revienne à des valeurs autorisant le traitement. Si la baisse est très importante votre médecin en sera informé et vous proposera, si nécessaire, un bilan sanguin toutes les deux semaines jusqu’à ce que l’effet disparaisse. Si l’effet secondaire persiste mais ne progresse pas après réduction des doses, le traitement sera poursuivi à dose réduite.

En cas de présence de germes dans les urines sans signes d’infection le traitement pourra être administré mais sous couverture d’un antiseptique urinaire. En cas d’infection urinaire symptomatique un traitement antibiotique vous sera prescrit en fonction des germes présents.

**Partie 6 - Quels sont vos droits ?**

Votre médecin doit vous fournir tous les détails concernant cette étude.

Votre participation à cette étude est entièrement volontaire. Si vous ne désirez pas prendre part à cette étude ou si vous souhaitez vous en retirer à quelque moment que ce soit, et quel que soit le motif, vous continuerez à bénéficier du suivi médical et cela n'affectera en rien votre surveillance future. Si vous décidez de participer à cette étude, votre médecin peut décider de vous faire arrêter les médicaments de l’essai à tout moment, en continuant une surveillance, si par exemple votre état de santé se dégrade et que d'autres traitements sont préférables ou si des effets secondaires le justifient.

Les données recueillies au cours de cette étude resteront strictement confidentielles, vos nom et adresse ne seront connus que de votre médecin. Compte tenu des nécessités de la recherche et de son analyse ultérieure, les données recueillies qui vous concernent feront l'objet d'un traitement informatisé et anonyme à l’unité de soutien méthodologique du CHU de Bordeaux. Les informations qui feront l’objet de ce traitement informatisé seront les suivantes : âge, sexe, antécédents médicaux, histoire de votre maladie, les traitements pris, les différentes évaluations réalisées au cours de l’étude, les événements cliniques et les résultats biologiques. Les articles 40 et 43 de la loi « Informatique et Libertés » prévoit votre droit d'accès, d'opposition et de rectification des données enregistrées sur informatique, à tout moment, par l’intermédiaire de votre médecin.

Cette étude a reçu un avis favorable du comité de protection des personnes pour les recherches biomédicales de Bordeaux (A) après examen lors de sa séance du 30/03/2005
Le promoteur de cette étude est le CHU de Bordeaux, 2 rue Dubernat, 33400 Talence qui a souscrit une assurance de responsabilité civile conformément à la loi.

Lorsque cette étude sera terminée, vous serez tenu informé personnellement des résultats par votre médecin dès que ceux-ci seront disponibles. Après avoir lu cette note d’information, n'hésitez pas à poser à votre médecin toutes les questions que vous désirez.

***Si vous acceptez de participer à cette étude, il vous suffit de signer le formulaire de consentement de participation*. Un exemplaire du document complet vous sera remis.**

**Nous vous remercions de votre collaboration à cette étude.**

**Nom du médecin ayant remis la note d'information**

**Hôpital : Date :**

**Paraphe du patient Paraphe du médecin**

**GLOSSAIRE**

- **Interféron** : Substance fabriquée par les globules blancs ayant des propriétés antivirales et immunomodulatrices.
- **Médicament immunosuppresseur** : Qui supprime ou réduit les réactions immunologiques spécifiques de l’organisme, en bloquant le système immunitaire de cet organisme. Médicaments qui diminuent la réponse immunitaire de l'organisme.
- **Médicament immunomodulateur** : Qui module les réactions immunitaires en les inhibant ou en les stimulant. . Médicament qui agit en modifiant les signaux émis par les cellules de défense de l'organisme
- **Réactions Immunitaires** : Ensemble de moyens de défense de l’organisme contre les agressions extérieures.
- **Etude contrôlée** : Etude correspondant aux critères scientifiques de bonne expérimentation clinique, avec un groupe témoin, et l’attribution des traitements par tirage au sort.
- **Ionogramme sanguin** : Formule représentant les concentrations des différents ions ou minéraux (sodium, potassium, chlore...) contenus dans le sang.
- **Créatininémie** : Mesure de la créatinine dans le sang.
- **Transaminase** : enzyme du foie.
- **Examen cytobactériologique des urines** : consiste à observer au microscope une goutte d'urine afin de rechercher la présence de globules blancs (ou leucocytes), de globules rouges (ou hématies) et d'éventuels microbes.
- **Réactions anaphylactiques** : Réaction aiguë allergique due à l'exposition de l’organisme à une substance étrangère précédemment présentée
- **Médicament antiémétique** : Médicament employé pour prévenir ou soulager les nausées et les vomissements.
- **Aménorrhées** : C'est l'absence de règles
- **Azoospermie**: c’est l'absence de spermatozoïdes dans le sperme.
- **Diurèse** : C’est la quantité d'urine par jour.
- **Alopécie** : correspond à une chute de cheveux sur tout ou une partie du cuir chevelu.
- **Cardiomyopathie** : Maladie du muscle cardiaque.
- **Fibrose pulmonaire** : transformation fibreuse des poumons.
- **Désordres hydro électrolytiques** : Déséquilibre de l’eau et des électrolytes dans l’organisme.
- **Rétention hydrosodée** : Présence en excès, dans l’organisme, d’eau et de sodium.
- **Troubles endocriniens** : Troubles caractéristiques de l’atteinte des glandes endocrines (se dit d’une glande dont la sécrétion est directement déversée dans le sang).
- **Ostéoporose** : Décalcification des os responsable d’une fragilité osseuse et, par suite, une augmentation du risque de fracture.
- **Intradermo-réaction (IDR) à la tuberculine** : Injection intradermique (entre l'épiderme et le derme) de tuberculine, contre laquelle l'organisme réagit en formant un bouton. La formation d'un bouton sur la peau dans les jours qui suivent l'intradermoréaction indique que l'organisme a été en contact avec (tuberculose ancienne ou en cours), ou que la vaccination par le BCG a été efficace et que l'organisme est donc immunisé contre la tuberculose.

**ANNEXE 4 : FORM ULAIRE DE CONSENTEMENT**

**ESSAI THERAPEUTIQUE RANDOMISE MULTICENTRIQUE**

**EN DOUBLE INSU EN GROUPES PARALLELES**

**DU CYCLOPHOSPHAMIDE INTRAVEINEUX VERSUS METHYLPREDNISOLONE DANS LE TRAITEMENT DE FOND DES FORMES PROGRESSIVES SECONDAIRES RECENTES DE SCLEROSE EN PLAQUES : ETUDE P.R.OM.E.S.S**

**Promoteur : Centre Hospitalier Universitaire de Bordeaux**

### Investigateur Coordonnateur : Pr. Bruno Brochet

**Formulaire de consentement**

Version protocole du 04/04/2005

*Recherche avec bénéfice individuel direct*

Je soussigné(e)..................................................................................................................(nom, prénom), certifie avoir lu et compris le document d’informations qui m’a été remis et avoir eu la possibilité de poser toutes les questions que je souhaitais au Dr ……………………………….

Je comprends les contraintes (en particulier les visites régulières à l’hôpital *et les différents examens)* liées à ma participation.

Je connais la possibilité qui m’est donnée d’interrompre le traitement à tout moment sans avoir à justifier ma décision mais je ferai mon possible pour informer le Dr . . . . . . . . . . . . . . . . . . . . . .

Cela ne remettra pas en cause la qualité des soins ultérieurs.

J’ai eu l’assurance que les décisions qui s’imposent pour ma santé seront prises à tout moment, conformément à l’état de connaissances sur la sclérose en plaques et à l’évolution de ma propre maladie.

J’accepte que les données enregistrées à l’occasion de ce protocole puissent faire l’objet d’un traitement informatisé. J’ai bien noté que le droit d’accès prévu par la loi “Informatique et Libertés” (article 40) s’exerce à tout moment auprès du Dr . . . . . . . . . . . . . . . . . . . . . . . . . et que je pourrai exercer mon droit de rectification et d’opposition auprès de celui -ci.

J’accepte que seuls les médecins ou organisateurs impliqués dans le déroulement de cet essai, ainsi que les représentants des Autorités de Santé aient accès à l’information me concernant dans le respect le plus strict de la confidentialité.

Mon consentement ne décharge en rien les organisateurs de l’étude de leurs responsabilités. Je conserve tous les droits garantis par la loi.

A l'issue de cette étude, vous serez tenu personnellement informé des résultats globaux par votre médecin dès qu'ils seront disponibles.

*Le promoteur de l’étude (CHU de Bordeaux, 12 rue Dubernat, 33400 Talence) a souscrit une assurance de responsabilité civile en cas de préjudice auprès de la société Gerling France*

*(n° 01/8088558) conformément aux dispositions de l’article L1127-07 du Code de la Santé Publique du 20 décembre 1988 et à l’article 5 de la loi du 25 juillet 1991*.

J'accepte de participer librement à cette recherche dans les conditions précisées dans le document d’information.

Fait à ..........................................le ...........................................

Nom  et prénom du Patient : .....................................................................................................................................

Signature du Patient

Je soussigné(e), Docteur . . . . . . . . . . . . . . . . . . . . . . . . ., certifie avoir communiqué toutes informations utiles concernant cette étude. Je m’engage à faire respecter les termes de cette note de consentement, conciliant le respect des droits et des libertés individuelles et les exigences d’un travail scientifique.

Nom du Médecin : ..................................................................................................................................................

*Adresse du Médecin...........................................................................................................................................*

*Numéro de téléphone du Médecin :……………………………………………..*

Fait à ............................................... le .............................................................

Signature du Médecin :

**ANNEXE 5 : EDSS ADAPTEE d’après Kurtzke (1983)
Seule la partie de 4 à 10 de l’échelle est présentée :**

Les scores fonctionnels (FSS) sont explicités en annexe 6.
Ce score EDSS est calculé au niveau du centre coordinateur à partir du test de marche et de l’examen neurologique.

1) Si le patient marche sans aide ni repos plus de 500 m (mais il doit présenter une limitation du périmètre de marche pour participer à l’étude) il est côté 4 en fonction de la combinaison des FSS (un score = 4 (ou FSS visuel 6); les autres = 0 ou 1 ; ou un score = 3 (ou FSS visuel 5 ou 4) et plus de deux scores = 2 ; ou deux scores = 3 (ou FSS visuel 5 ou 4) et un score ou plus = 2 ; ou plus de deux scores = 3 (ou FSS visuel 5 ou 4); mais tous < 4 (ou FSS visuel 6).
2) Si la marche est limitée mais le périmètre supérieur à 300m et inférieur à 500 m (avec habituellement les mêmes combinaisons de FSS que précédemment) le score est 4,5.
3) Si la marche est limitée à moins de 300 m le score suit les grades suivants :
- 5.0: Capable de marcher sans aide ni repos entre 200m et 299m

- 5.5: Capable de marcher sans aide ni repos entre 100m et 199m

- 6.0: Ne peut marcher sans aide ni repos au moins 100m mais peut parcourir 100m avec une aide unilatérale constante ou intermittente, avec possibilité d’un repos bref en cours de route,ou marche avec 2 cannes plus de 200 m.

- 6.5: Ne peut parcourir 100 m avec une aide mais peut marcher avec une aide bilatérale 20 m sans arrêt;

- 7.0: Ne peut marcher avec une aide bilatérale 20 m sans arrêt mais peut marcher 5m avec aide. Habituellement au fauteuil roulant au moins 12h/jour; mais effectue ses transferts, et fait avancer lui-même le fauteuil ;
- 7.5:confiné au fauteuil roulant; ne fait que quelques pas (moins de 5 m); a parfois besoin d'aide pour le transfert; peut faire avancer lui-même le fauteuil; ne peut y rester toute la journée; peut nécessiter un fauteuil électrique ;

-8.0:confiné au lit ou au fauteuil; mais promené au fauteuil par un tiers; peut rester hors du lit la majeure partie de la journée ;

-8.5 confiné au lit la majeure partie de la journée

- 9.0 grabataire

- 9.5 totalement impotent

- 10. Décès lié à la SEP.

**ANNEXE 6 : SCORES FONCTIONNELS (Kurtzke) : FSS**
PYRAMIDAL:
(déficit minime = testing musculaire à 4; déficit modéré= testing à 3 (mouvement possible contre gravité); déficit sévère: testing <3 )

0-Pas de signes pyramidaux

1-signes pyramidaux; pas de déficit ni gène fonctionnelle

2-déficit minime de plus d'un membre (minimum à 4) ou monoparésie modérée (minimum 3)

3-monoparésie sévère (testing <3); ou paraparésie ou hémiparésie modérée (minimum 3)

4-paraparésie ou hémiparésie sévère (<3); ou tri-tétraparésie modérée (minimum 3), ou monoplégie;

5-paraplégie ou hémiplégie ou -tri- ou tétraparésie sévère (testing <3)

6-tétraplégie

CERVELET

0-aucun signe cérébelleux

1-signes cérébelleux sans gène fonctionnelle

2-ataxie statique et/ou incoordination des membres supérieurs minime(s) (signes évidents à l'examen mais interfèrent peu avec la fonction)

3-ataxie statique et/ou incoordination des membres supérieurs modérée (s) (signes évidents à l'examen qui interfèrent avec la fonction)

4-ataxie sévère statique et/ou cinétique des 4 membres (toutes les fonctions sont très difficiles)

5-mouvements coordonnés impossibles.

TRONC CEREBRAL

0-aucune atteinte

1-anomalies cliniques des fonctions du tronc cérébral sans gène fonnctionelle.

2à5: anomalies avec gène fonctionnelle: coter la plus sévère:
Nystagmus:
 2-modéré (non épuisable dans le regard latéral; pas dans la position de repos);
 3-sévère (permanent, interfère avec vision, OIA, oscillopsie).

Paralysie oculomotrice:
2-modérée (diplopie, POM incomplète)
3-sévère (paralysie complète d'un mouvement d'un oeil)
Atteinte du V:
2-minime hypoesthésie

3-hypoesthésie de 2 branches du V

4-anesthésie d'un ou deux nerfs trijumeaux

Atteinte du VII:
2-parésie faciale

3-impossibilté de fermer l'oeil

4-plégie faciale uni ou bilatérale

Dysarthrie:
2-minime

3-modérée (ne rends pas incompréhensible)

4-incompréhensible

5-ne peut parler

Troubles de la déglutition
2-minimes

3-modérées

4-difficultés constantes pour avaler

5-ne peut avaler

VISION: Acuité visuelle corrigée:

0-AV normale; pas de symptôme;

1-scotôme; AV >67%;

2-oeil le plus atteint entre 34% et 67%;

3-oeil le plus atteint entre 20 et 33% % (autre oeil>30%);

4-oeil le plus atteint entre 10 et 20%% (autre oeil>30%) ou entre 20 et 33% et autre oeil<31%

5-oeil le plus atteint <10% (autre oeil>30%) ou entre 10 et 20% et autre oeil <31%

6-oeil le plus atteint <10% et autre oeil <31%

SENSIBILITE

0-Normal

1-Le patient ne se rend pas compte du déficit;
Tact et douleur normaux; Hypoesthésie (minime) vibratoire et ou graphestésique d'un ou deux membres; sens de position normal

2-une des possibilités suivantes:

*Tact/douleur: hypoesthésie d'un ou deux membres, discrimine normalement

*Sens de position: diminution minime d'1 ou 2 mb

* Hypoesthésie vibratoire et ou graphestésique minime de 3 à 4 membres ou marquée de1 ou membres.

3- une des possibilités suivantes:

*Tact/douleur: discrimination anormale d'un ou deux membres; ou hypoesthésie minime (discrimination normale) de 3 à 4 membres;

*Sens de position: diminution modérée d'un ou deux membres ou minime de 3 à 4 membres;

*Anesthésie vibratoire d'un ou deux membres ou hypoesthésie marquée de 3 à 4 membres.

4- une des possibilités suivantes:

*Tact-douleur: discrimination anormale dans 3-4membres ou impossible dans 1-2 membres;

*Sens de position: diminution modérée de 3-4 membres ou déficit total d'1-2 membres;

*Anesthésie vibratoire totale;

5- une des possibilités suivantes:

*Anesthésie à tous les modes d'un ou deux membres;

*Tact-Douleur: discrimination impossible des 4 membres;

*Sens de position: perte totale de la proprioception

6-Anesthésie totale (sauf extrémité céphalique).

SPHINCTERS:

0-Aucun trouble

1-dysurie ou mictions impérieuses minimes (pas d'impact fonctionnel sur le mode de vie);

2-dysurie ou mictions impérieuses modérées ou troubles modérés de la défécation ou incontinence urinaire rare; autosondages intermittents.

3-fuites fréquentes.

4-sondage à demeure ou quasi permanent; mesures constantes d'aide à l'exonération fécale;

5-perte totale des fonctions sphinctériennes vésicales;

6-perte totale des fonctions sphinctériennes;

MENTAL:

0-normal

1-dépression isolée (ne pas en tenir compte pour le calcul EDSS)

2-atteinte minime des fonctions cognitives (observables par le patient et son entourage mais non apparents dans l'entretien simple)

3-troubles cognitifs modérés liés à la SEP (MMS perturbé; mais orientation spatio-temporelle normale)

4-troubles cognitifs marqués (avec retentissement sur vie quotidienne, DTS débutante)

5-démence (DTS complète).

**ANNEXE 7 : LE SCORE COMPOSITE**
(MSFC ou multiple sclerosis functional composite).
Les limites de l’EDSS comme critère d’efficacité clinique lors des essais thérapeutiques dans la SEP a amené la National Multiple Sclerosis Society (NMSS) des USA à former un groupe de travail pour développer un nouvel outil
LE SCORE

Le MSFC comprend la combinaison de 3 mesures :
 - une mesure chronométrée d’habileté motrice du membre supérieur, le nine hole peg test (9HPT),
 - une mesure de marche : temps de marche chronométré sur 8 m
 - un test cognitif mesurant l’attention, le PASAT (version 3 secondes).
LE NINE HOLE PEG TEST :
Il consiste en une tâche manuelle chronométrée. Le patient doit placer 9 chevilles en bois dans des puits aménagés dans une boîte carré en bois. Ce test est très reproductible et très sensible au changement. Comme tous les tests chronométrés il n’est pas exempt d’effet plafond. Il fait partie du MCSC. Une aggravation de 20% du temps de réalisation du test est significative.

LE TEMPS DE MARCHE :
On chronomètre le temps de marche avec ou sans aide sur 8 m sur terrain plat.

LE PASAT :
Le Paced Auditory Serial Attention Task (PASAT), évalue l’attention soutenue et la vitesse de traitement de l’information (dimension attentionnelle de la mémoire de travail). Une série de chiffre est présentée au rythme d’un chiffre toutes les 3 secondes. La consigne est la suivante :
Ecoutez les deux premiers chiffres, additionnez-les, et donnez moi le total. Lorsque vous entendez le chiffre suivant, additionnez-le au chiffre précédent. Il ne faut pas donner un total cumulé de tous les chiffres, mais simplement la somme des 2 derniers chiffres entendus.

Par exemple, si vous avez entendu 5, 7, 3 et 2, vous devriez donner les réponses 12, 10, 5.

MESURE DU SCORE COMPOSITE :
Il est réalisé 4 tests avec le 9HPT (deux avec chaque main), deux tests de marche et un test PASAT à 3 secondes. La moyenne des moyennes des scores de main droite et de la main gauche est calculée. La moyenne des scores de deux tests de marche sur 8 m est calculée et le nombre de réponses correctes au PASAT est utilisé.
CALCUL :

Pour combiner ces mesures exprimées en unités différentes (secondes et nombres de réponses correctes) la méthode des Z scores est utilisée. Un Z score est un nombre standardisé qui exprime à quelle distance de la moyenne des résultats d’une population de référence se situe le résultat du patient. Le Z score est exprimé en unité de déviation standard habituellement de –3 à +3 même s’il n’y a en fait aucune limitation à sa valeur. Le Z score est donc obtenu en soustrayant la moyenne de la population de référence du résultat du test et en divisant le résultat par la déviation standard de cette population.
Le score MFSC est obtenu par la formule : (Z MS – Z MI + Z cognitif)/3
Tout dépend donc de la population de référence choisie qui ne doit pas être une population témoin saine.

En pratique plusieurs méthodes différentes sont proposées selon que l’on veut comparer deux groupes dans un essai thérapeutique, suivre une cohorte de patients ou comparer des résultats entre essais. Dans une étude de cohorte il est ainsi recommandé d’utiliser les scores à l’inclusion (t0) comme scores de référence. Le score MSFC pour un patient à un temps X (tX) est alors calculé en fonction de l’inverse de ses scores des 2 mains au 9HPT (1/9HPT) à tX et des scores au test de marche sur 8 m (8 m tX) et au PASAT 3 secondes (PASAT3) :
Score = (moyenne 1/9HPT tX – moyenne 1/9HPT t0)/(déviation standard t0 1/9HPT)
- (moyenne 8 m tX – moyenne 8 m t0)/(déviation standard t0 8 m)
+ (moyenne PASAT3 tX – moyenne PASAT3 t0)/(déviation standard t0 PASAT3)
Si on veut comparer les résultats à d’autres populations on peut utiliser comme population de référence les données de la NMSS task force. Le score sera alors calculé selon la formule :
(moyenne 1/9HPT tX – 0,0439)/(0,0101) - (moyenne 8 m tX –9,5353)/(11,4058) + (moyenne PASAT3 tX – 45,0311)/(12,00771)
Les chiffres figurant dans cette formule étant les moyennes et déviations standards de cette population de référence.
L’utilisation de cette population de référence externe change bien entendu les Z scores mais ne modifie pas les différences entre les sous-groupes.
Dans un essai thérapeutique la méthode recommandée est d’utiliser la moyenne des valeurs à l’inclusion de l’ensemble de la population étudiée pour calculer les scores ce qui sera fait ici.

**ANNEXE 8 : ECHELLE SEP 59**

COMMENT REPONDRE A L’AUTOQUESTIONNAIRE: Les questions qui suivent portent sur votre santé, telle que vous la ressentez. Ces informations nous permettront de mieux savoir comment vous vous sentez dans votre vie de tous les jours.Veuillez répondre à toutes les questions en entourant le chiffre correspondant à la réponse choisie, comme il est indiqué. Si vous ne savez pas très bien comment répondre, choisissez la réponse la plus proche de votre situation.

1 - Dans l'ensemble, pensez-vous que votre santé est (entourez la réponse de votre choix, une par ligne) : Excellente............................... 1

Très bonne.............................. 2

Bonne...................................... 3

Médiocre................................ 4

Mauvaise................................ 5

2 - Par rapport à l'année dernière à la même époque, comment trouvez-vous votre état de santé en ce moment ? (Entourez la réponse de votre choix, une par ligne)

Bien meilleur que l'an dernier... 1

Plutôt meilleur............................ 2

A peu près pareil........................ 3

Plutôt moins bon......................... 4

Beaucoup moins bon.................. 5

3 - Voici une liste d'activités que vous pouvez avoir à faire dans votre vie de tous les jours. Pour chacune d'entre elles indiquez si vous êtes limité(e) en raison de votre état de santé actuel.(entourez la réponse de votre choix, une par ligne)

| LISTE D'ACTIVITES | OUI BEAUCOUP LIMITE(E) | OUI UN PEU LIMITE(E) | NON PAS DU TOUT LIMITE(E) |
| --- | --- | --- | --- |
| a - Efforts physiques importants tels que courir, soulever un objet lourd, faire du sport. | 1 | 2 | 3 |
| b - Effort physiques modérés tels que déplacer une table, passer l'aspirateur, jouer aux boules. | 1 | 2 | 3 |
| c - Soulever et porter les courses. | 1 | 2 | 3 |
| d - Monter plusieurs étages par l'escalier | 1 | 2 | 3 |
| e - Monter un étage par l'escalier | 1 | 2 | 3 |
| f - Se pencher en avant, se mettre à genoux, s'accroupir. | 1 | 2 | 3 |
| g - Marcher plus d'un km à pied | 1 | 2 | 3 |
| h - Marcher plusieurs centaines de mètres. | 1 | 2 | 3 |
| i - Marcher une centaine de mètres | 1 | 2 | 3 |
| j - Prendre un bain, une douche ou s'habiller | 1 | 2 | 3 |

4 - Au cours de ces 4 dernières semaines, et en raison de votre état physique :(entourez la réponse de votre choix, une par ligne)

|  | OUI | NON |
| --- | --- | --- |
| a - Avez-vous réduit le temps passé à votre travail ou à vos activités habituelles. | 1 | 2 |
| b - Avez-vous accompli moins de choses que ce que vous auriez souhaité | 1 | 2 |
| c - Avez-vous dû arrêter de faire certaines choses | 1 | 2 |
| d - Avez-vous eu des difficultés à faire votre travail ou toute autre activité | 1 | 2 |

5 - Au cours de ces 4 dernières semaines, et en raison de votre état émotionnel, (vous sentir triste, nerveux(se) ou déprimé(e)) (entourez la réponse de votre choix, une par ligne) :

|  | OUI | NON |
| --- | --- | --- |
| a - Avez-vous réduit le temps passé à votre travail ou à vos activités habituelles | 1 | 2 |
| b - Avez-vous accompli moins de choses que ce que vous auriez souhaité | 1 | 2 |
| c - Avez-vous eu des difficultés à faire ce que vous aviez à faire avec autant de soin et d'attention | 1 | 2 |

6 - Au cours de ces 4 dernières semaines dans quelle mesure est-ce que votre état de santé physique ou émotionnel, vous a gêné(e) dans votre vie et vos relations avec les autres : votre famille, vos amis, vos connaissances ? (entourez la réponse de votre choix, une par ligne)

Pas du tout........................................ 1

Un petit peu...................................... 2

Moyennement.................................. 3

Beaucoup.......................................... 4

Enormément..................................... 5

7 - Au cours de ces 4 dernières semaines, quelle a été l'importance de vos douleurs physiques (entourez la réponse de votre choix, une par ligne) ?

- Nulle................................................. 1

- Très faible........................................ 2

- Faible................................................ 3

- Moyenne.......................................... 4

- Grande.............................................. 5

- Très grande...................................... 6

8 - Au cours de ces 4 dernières semaines, dans quelle mesure vos douleurs physiques vous ont limite(e) dans votre travail ou vos activités domestiques (entourez la réponse de votre choix, une par ligne) ?

- Pas du tout....................................... 1

- Un petit peu..................................... 2

- Moyennement................................. 3

- Beaucoup......................................... 4

- Enormément................................... . 5

9 - Les questions qui suivent portent sur comment vous vous êtes senti (e) au cours de ces 4 dernières semaines. Pour chaque question merci d'indiquer la réponse qui vous semble la plus appropriée. Au cours de ces 4 dernières semaines, y-a-t-il eu des moments où (entourez la réponse de votre choix, une par ligne) :

|  | EN PERMANENCE | TRES  SOUVENT | SOUVENT | QUELQUEFOIS | RAREMENT | JAMAIS |
| --- | --- | --- | --- | --- | --- | --- |
| a - Vous vous êtes senti(e) dynamique | 1 | 2 | 3 | 4 | 5 | 6 |
| b - Vous vous êtes senti(e) très nerveux(se) | 1 | 2 | 3 | 4 | 5 | 6 |
| c - Vous vous êtes senti(e) si découragé(e) que rien ne pouvait vous remonter le moral | 1 | 2 | 3 | 4 | 5 | 6 |
| d - Vous vous êtes senti(e) calme et détendu(e) | 1 | 2 | 3 | 4 | 5 | 6 |
| e - Vous vous êtes senti(e) débordant(e) d'énergie | 1 | 2 | 3 | 4 | 5 | 6 |
| f - Vous vous êtes senti(e) triste et abattu(e) | 1 | 2 | 3 | 4 | 5 | 6 |
| g - Vous vous êtes senti(e) épuisé(e) | 1 | 2 | 3 | 4 | 5 | 6 |
| h - Vous vous êtes senti(e) heureux(se) | 1 | 2 | 3 | 4 | 5 | 6 |
| i - Vous vous êtes senti(e) fatigué(e) | 1 | 2 | 3 | 4 | 5 | 6 |

10 - Au cours de ces 4 dernières semaines y-a-t-il eu des moments où votre état de santé, physique ou émotionnel, vous a gêné(e) dans votre vie et vos relations avec les autres: votre famille, vos amis, vos connaissances (entourez la réponse de votre choix, une par ligne) ?

- Tout le temps.................................... 1

- Une bonne partie du temps.............. 2

- De temps en temps........ .................. 3

- Rarement............................. ............ 4

- Jamais.............................................. 5

11 - Indiquez pour chacune des phrases suivantes dans quelle mesure elles sont vraies ou fausses dans votre cas (entourez la réponse de votre choix, une par ligne) :

|  | TOTALEMENT VRAIE | PLUTOT VRAIE | JE NE SAIS PAS | PLUTOT FAUSSE | TOTALEMENT FAUSSE |
| --- | --- | --- | --- | --- | --- |
| a - Je tombe malade plus facilement que les autres | 1 | 2 | 3 | 4 | 5 |
| b - Je me porte aussi bien que n'importe qui | 1 | 2 | 3 | 4 | 5 |
| c - Je m'attends à ce que ma santé se dégrade | 1 | 2 | 3 | 4 | 5 |
| d - Je suis en parfaite santé | 1 | 2 | 3 | 4 | 5 |

12 - Concernant votre santé, au cours de ces 4 dernières semaines, y a-t-il eu des moments où (entourez la réponse de votre choix, une par ligne) :

|  | EN PERMANENCE | TRES  SOUVENT | SOUVENT | QUELQUEFOIS | RAREMENT | JAMAIS |
| --- | --- | --- | --- | --- | --- | --- |
| a - Vous vous êtes senti découragé par vos problèmes de santé ? | 1 | 2 | 3 | 4 | 5 | 6 |
| b - Vous vous êtes senti frustré par votre état de santé ? | 1 | 2 | 3 | 4 | 5 | 6 |
| c - Votre santé a-t-elle été un souci dans votre vie ? | 1 | 2 | 3 | 4 | 5 | 6 |
| d - Vous vous êtes senti accablé par vos problèmes de santé ? | 1 | 2 | 3 | 4 | 5 | 6 |

13 - Concernant votre sommeil, au cours de ces 4 dernières semaines, y a-t-il eu des moments où (entourez la réponse de votre choix, une par ligne) :

|  | EN PERMANENCE² | TRES SOUVENT | SOUVENT | QUELQUEFOIS | RAREMENT | JAMAIS |
| --- | --- | --- | --- | --- | --- | --- |
| a - Vous vous êtes senti perturbé dans votre sommeil (crampes, contractures, un sommeil agité, une tension nerveuse) ? | 1 | 2 | 3 | 4 | 5 | 6 |
| b - Vous vous êtes senti reposé au réveil, le matin ? | 1 | 2 | 3 | 4 | 5 | 6 |

14 - Concernant votre attention, au cours de ces 4 dernières semaines y a-t-il eu des moments où (entourez la réponse de votre choix, une par ligne) :

|  | EN PERMANENCE | TRES  SOUVENT | SOUVENT | QUELQUEFOIS | RAREMENT | JAMAIS |
| --- | --- | --- | --- | --- | --- | --- |
| a - Vous avez eu des difficultés à vous concentrer ou à réfléchir? | 1 | 2 | 3 | 4 | 5 | 6 |
| b - Vous avez eu des difficultés pour garder longtemps votre attention sur une activité ? | 1 | 2 | 3 | 4 | 5 | 6 |
| c - Vous avez eu des troubles de mémoire ? | 1 | 2 | 3 | 4 | 5 | 6 |
| d - D’autres (tels que des membres de la famille ou des amis), ont noté que vous avez eu des problèmes de mémoire ou de concentration ? | 1 | 2 | 3 | 4 | 5 | 6 |

15 - Les questions qui suivent portent sur votre vie sexuelle et votre satisfaction personnelle. Pour chaque question merci d’indiquer la réponse qui vous semble la plus appropriée. Au cours de ces 4 dernières semaines dans quelle mesure avez-vous eu un problème de (entourez la réponse de votre choix, une par ligne) :

|  | EN PERMANENCE | SOUVENT | QUELQUEFOIS | JAMAIS |
| --- | --- | --- | --- | --- |
| a - Manque d’intérêt sexuel | 1 | 2 | 3 | 4 |
| b - Difficulté à obtenir une intimité, un climat confortable | 1 | 2 | 3 | 4 |
| c - Difficulté à ressentir du plaisir | 1 | 2 | 3 | 4 |
| d - Capacité à satisfaire votre partenaire | 1 | 2 | 3 | 4 |

16 - Globalement, au cours de ces 4 dernières semaines, dans quelle mesure avez vous été satisfait de votre vie sexuelle ?

- Très satisfaisant 1

- Assez satisfaisant 2

- Ni satisfaisant, ni insatisfait 3

- Assez insatisfait 4

- Très insatisfait 5

17 - Parfois les gens recherchent d’autres personnes pour trouver une compagnie, de l’aide ou un soutien. Lorsque vous en ressentez le besoin, dans quelle mesure de telles personnes sont-elles disponibles (entourez la réponse de votre choix, une par ligne) ?

|  | EN PERMANENCE | TRES  SOUVENT | SOUVENT | QUELQUEFOIS | RAREMENT | JAMAIS |
| --- | --- | --- | --- | --- | --- | --- |
| a - Quelqu’un pour vous aider dans les tâches quotidiennes en cas de maladie | 1 | 2 | 3 | 4 | 5 | 6 |
| b - Quelqu’un à aimer et pour qui vous comptez | 1 | 2 | 3 | 4 | 5 | 6 |
| c - Quelqu’un avec qui vous pouvez vous détendre | 1 | 2 | 3 | 4 | 5 | 6 |
| d - Quelqu’un qui pourrait vous aider à résoudre un problème personnel | 1 | 2 | 3 | 4 | 5 | 6 |

18 - Au cours de ces 4 dernières semaines y-a-t-il eu des moments où les problèmes urinaires ou intestinaux vous ont gênés dans vos relations avec votre famille, amis ou connaissances :

Pas du tout........................................ 1

Un petit peu...................................... 2

Moyennement....................... 3

Beaucoup.............................. 4

Enormément..................................... 5

19 - Au cours de ces 4 dernières semaines, dans quelle mesure votre plaisir de vivre a-t-il ete gene par la douleur :

Pas du tout........................................ 1

Un petit peu...................................... 2

Moyennement.................................. 3

Beaucoup.......................................... 4

Enormément..................................... 5

20 - Globalement, comment évaluez-vous votre qualité de vie ?

☺ 😐 ☹

|______|______|______|______|______|______|______|______|______|______|

10 9 8 7 6 5 4 3 2 1 0

| La meilleure qualité de vie possible | La plus mauvaise qualité de vie voire pire que la mort |
| --- | --- |

21 - Globalement, quel terme decrit le mieux votre vie ?

Horrible........................................................................ 1

Malheureuse ........ ... 2

Plutôt insatisfaisante .......... 3

Partagé entre la satisfaction et l’insatisfaction........ . 4

Plutôt satisfaisante..................................... ......... .. 5

Heureuse ........... 6

Très heureuse........ ........................... .......... 7

# ANNEXE 9 : Echelle MSIS : *ECHELLE DE MESURE DU RETENTISSEMENT DE LA SEP*

**MULTIPLE SCLEROSIS IMPACT SCALE (MSIS –29)**

Dans le questionnaire ci-dessous, nous vous demandons votre avis sur le retentissement de la Sclérose en Plaques dans votre vie quotidienne **pendant les 2 dernières semaines.**

Pour chaque item, **entourez** **le** **chiffre** qui correspond **le** **mieux** à votre situation (**un seul** chiffre ;

même si vous hésitez, choisissez **un seul** chiffre).

Veuillez répondre à **toutes** **les questions.**

**Vérifier que vous avez bien répondu à TOUTES les questions avant de passer à la page suivante**

# Vérifiez que vous avez bien entouré UN SEUL chiffre pour CHACUNE des questions. Merci

**© 2000 Neurological Outcome Measures Unit, 4^th^ Floor Queen Mary Wing, NHNN, Queen Square, London WC1N 3BG, UK**

Traduction française 2002 : CITESEP (Hôpital Tenon et Université Paris VI), 4 rue de La Chine, 75020. Paris, France.

**ANNEXE 10 : ECHELLE MSWS12**

# ECHELLE DE MARCHE DANS LA SEP

*MULTIPLE SCLEROSIS WALKING SCALE (MSWS –12)*

Ces questions concernent la **limitation de votre marche** due à la Sclérose en Plaques (SEP) au cours **des 2 dernières semaines.**

Pour chaque item, **entourez** **le** **chiffre** qui décrit **le** **mieux** votre situation (**un seul chiffre** ; même si vous hésitez, choisissez **un seul chiffre**).

Veuillez répondre à **toutes** **les questions,** même si certaines vous semblent proches l’une de l’autre ou vous paraissent ne pas s’appliquer à votre cas.

Si vous **ne pouvez pas marcher du tout**, cochez cette case

ANNEXE 11 : PHARMACIE

##### PROTOCOLE CPM (ENDOXAN DANS LA S.E P) : SCHEMA D’ADMINISTRATION PROPOSE

| **Horaire** | **BRAS CPM (Endoxan)** | **BRAS CORTICOIDE (Methylprednisolone)** |
| --- | --- | --- |
| T = 0T = 0 **T = 30 min**  **T = 3heures** T=3h30 min **T = 8heures** | **Début hydratation :**  . **Acte infirmier** : mise en place poche G5 1 l  . **Produit fourni par la Pharmacie :**  poche gluc 5% 1 litre + 3 mg odansetron  (Zophren) +/- ( ½ dose)/  **IV lente sétron :**  **Acte infirmier :** IV lente dans tubulure  . **Produit fourni par la Pharmacie :**  seringue 10 ml : 8 mg odansetron (Zophren) dans G5%. Début perfusion CPM **. Acte infirmier :** branchement poche en Y  **. Produit fourni par la Pharmacie :**  poche de glucosé 5 % 500 ml + CPM  (Endoxan) dose totale Changement perfusion hydratation **Acte infirmier** : mise en place poche G5 1 l  . **Produit fourni par la Pharmacie :**  poche gluc 5% 1 litre + 3 mg odansetron  (Zophren) Arrêt perfusion CPM **. Acte infirmier :** débranchement poche en Y  **Arrêt Protocole administration** | **Début hydratation :**  . **Acte infirmier** : mise en place poche G5 1 l  . **Produit fourni par la Pharmacie :**  poche gluc 5% 1 litre  **IV lente placébo:**  **Acte infirmier :** IV lente dans tubulure  . **Produit fourni par la Pharmacie :**  seringue 10 ml de G 5 % Début perfusion Methylprednisolmone **. Acte infirmier :** branchement poche en Y  **. Produit fourni par la Pharmacie :**  poche de glucosé 5 % 500 ml + 1 g  méthylprednisolone Changement perfusion hydratation **Acte infirmier** : mise en place poche G5 1 l  . **Produit fourni par la Pharmacie :**  poche gluc 5% 1 litre Arrêt perfusion CPM **. Acte infirmier :** débranchement poche en Y  **Arrêt Protocole administration** |

**REMARQUE : Quelque soit le bras d’inclusion du patient :**

- **les produits fournis par la pharmacie seront présentés de manière rigoureusement identique**
- **ils seront identifiés par un étiquetage identique comportant : Nom du patient, prénom, service, N° d’inclusion / randomisation, N° de la cure , date , date limite d’utilisation, conditions de conservation.**

#### EVALUATION DU COUT PHARMACEUTIQUE PAR CURE : PROTOCOLE CPM (ENDOXAN DANS LA S.E P) :

**A/ BRAS CPM (ENDOXAN)**

Coût évalué sur la base des prix marchés CHU de Bordeaux en utilisant le schéma d’administration proposé précédent.

| **Préparation** | Présentation | **Coût unitaire** | **Nombre unités** | **Coût total** |
| --- | --- | --- | --- | --- |
| **1/ Odansétron (Zophren) 8 mg pour IV lente**  **2/ Perfusion glucosé 5 % hydrat**  **+ odansetron (Zophren) 3 mg**    - Poche souple Gluc 5 %  - Zophren 8 mg  **3/ CPM (Endoxan) pour perf sur 3 heures(Pos moy 1400 mg)**  **- Poche souple Gluc 5 %**  **- Endoxan 1000 mg**  **- Endoxan 500 mg**  **4/ Opération pharmaceutique** | Seringue 10 ml :  4 ml zophren + 6 ml G 5%  Poche souple G 5% 1 litre  Poche souple G 5 500 ml  Temps évalué : 20 minutes  (facturé selon TAREX) | 2.35 €  0.53 €  2.35 €  5 €  0.42 €  10.57 €  5.06 €  5.09€ | 1  2  1  1  1  1  1  1 | 2.35 €  1.06 €  2.35 €  5 €  0.42 €  10.57 €  5.08 €  5.09 € |
| **Total** |  |  |  | **27 €** |

**REMARQUE**: Calcul basé sur une posologie moyenne de 1400 mg de CPM

**B/ BRAS CORTICOIDE ( Methylprednisolone)**

Coût évalué sur la base des prix marchés CHU de Bordeaux en utilisant le schéma d’administration proposé précédent.

| **Préparation** | Présentation | **Coût unitaire** | **Nombre unités** | **Coût total** |
| --- | --- | --- | --- | --- |
| **1/ Placébo en IV lente (G 5%)**  **2/ Perfusion glucosé hydratation**  **3/ Méthylprednisolone 1 gr en perfusion sur 3 heures**  **-** Poche souple G 5% 500 ml  - Methylprednisolone flacon 500  mg  **4/ Opération pharmaceutique** | Seringue 10 ml (10 ml G 5 %)  Poche souple Gluc 5 % 1 litre  Poche souple Gluc 5 % 500 ml.  Temps évalué : 10 minutes  (facturé selon TAREX) | - 1. €   0.53 €  0.42 €  6.22 €  2.5 € | 1  2  1  2  1 | - 1. €   1.06 €  0.42 €  12.44 €  2.5 € |
| Total |  |  |  | **16.1 €** |

**ANNEXE** 13

# DECLARATION D'HELSINKI DE L'ASSOCIATION MEDICALE MONDIALE

# Principes éthiques applicables aux recherches médicales sur des sujets humains

Adoptée par la 18e Assemblée générale, Helsinki, Juin 1964 et amendée par les

29e Assemblée générale, Tokyo, Octobre 1975

35e Assemblée générale, Venise, Octobre 1983

41e Assemblée générale, Hong Kong, Septembre 1989

48e Assemblée générale, Somerset West (Afrique du Sud), Octobre 1996

52e Assemblée générale, Edimbourg, Octobre 2000

l'Assemblée générale de l'AMM, Washington 2002 (addition d'une note explicative concernant le paragraphe 29) et

l'Assemblée générale de l'AMM, **Tokyo 2004 (**addition d'une note explicative concernant le paragraphe 30).

### INTRODUCTION

1. La Déclaration d'Helsinki, élaborée par l'Association médicale mondiale, constitue une déclaration de principes éthiques dont l'objectif est de fournir des recommandations aux médecins et autres participants à la recherche médicale sur des êtres humains. Celle-ci comprend également les études réalisées sur des données à caractère personnel ou des échantillons biologiques non anonymes.

2. La mission du médecin est de promouvoir et de préserver la santé de l'être humain. Il exerce ce devoir dans la plénitude de son savoir et de sa conscience.

3. Le Serment de Genève de l'Association médicale mondiale lie le médecin dans les termes suivants : "La santé de mon patient sera mon premier souci" et le Code international d'éthique médicale énonce que "le médecin devra agir uniquement dans l'intérêt de son patient lorsqu'il lui procure des soins qui peuvent avoir pour conséquence un affaiblissement de sa condition physique ou mentale".

4. Les progrès de la médecine sont fondés sur des recherches qui, in fine, peuvent imposer de recourir à l'expérimentation humaine.

5. Dans la recherche médicale sur les sujets humains, les intérêts de la science et de la société ne doivent jamais prévaloir sur le bien-être du sujet.

6. L'objectif essentiel de la recherche médicale sur des sujets humains doit être l'amélioration des méthodes diagnostiques, thérapeutiques et de prévention, ainsi que la compréhension des causes et des mécanismes des maladies. Les méthodes diagnostiques, thérapeutiques et de prévention, même les plus éprouvées, doivent constamment être remises en question par des recherches portant sur leur efficacité, leur efficience et leur accessibilité.

7. Dans la recherche médicale comme dans la pratique médicale courante, la mise en œuvre de la plupart des méthodes diagnostiques, thérapeutiques et de prévention expose à des risques et à des contraintes.

8. La recherche médicale est soumise à des normes éthiques qui visent à garantir le respect de tous les êtres humains et la protection de leur santé et de leurs droits. Certaines catégories de sujets sont plus vulnérables que d'autres et appellent une protection adaptée. Les besoins spécifiques des sujets défavorisés au plan économique comme au plan médical doivent être identifiés. Une attention particulière doit être portée aux personnes qui ne sont pas en mesure de donner ou de refuser elles-mêmes leur consentement, à celles qui sont susceptibles de donner leur consentement sous la contrainte, à celles qui ne bénéficieront pas personnellement de la recherche et à celles pour lesquelles la recherche est conduite au cours d'un traitement.

9. L'investigateur doit être attentif aux dispositions éthiques, légales et réglementaires applicables à la recherche sur les sujets humains dans son propre pays ainsi qu'aux règles internationales applicables. Aucune disposition nationale d'ordre éthique, légal et réglementaire ne doit conduire à affaiblir ou supprimer les mesures protectrices énoncées dans la présente déclaration.

**B. PRINCIPES FONDAMENTAUX APPLICABLES A TOUTE FORME DE**

**RECHERCHE MEDICALE**

10. Dans la recherche médicale, le devoir du médecin est de protéger la vie, la santé, la dignité et l'intimité de la personne.

11. La recherche médicale sur des êtres humains doit se conformer aux principes scientifiques généralement reconnus. Elle doit se fonder sur une connaissance approfondie de la littérature scientifique et des autres sources pertinentes d'information ainsi que sur une expérimentation appropriée réalisée en laboratoire et, le cas échéant, sur l'animal.

12. Des précautions particulières doivent entourer les recherches pouvant porter atteinte à l'environnement et le bien-être des animaux utilisés au cours des recherches doit être préservé.

13. La conception et l'exécution de chaque phase de l'expérimentation sur des sujets humains doivent être clairement définies dans un protocole expérimental. Ce protocole doit être soumis pour examen, commentaires, avis et, le cas échéant, pour approbation, à un comité d'éthique mis en place à cet effet. Ce comité doit être indépendant du promoteur, de l'investigateur ou de toute autre forme d'influence indue. Il doit respecter les lois et règlements en vigueur dans le pays où s'effectuent les recherches. Il a le droit de suivre le déroulement des études en cours. L'investigateur a l'obligation de fournir au comité des informations sur le déroulement de l'étude portant en particulier sur la survenue d'événements indésirables d'une certaine gravité. L'investigateur doit également communiquer au comité, pour examen, les informations relatives au financement, aux promoteurs, à toute appartenance à une ou des institutions, aux éventuels conflits d'intérêt ainsi qu'aux moyens d'inciter des personnes à participer à une recherche.

14. Le protocole de la recherche doit contenir une déclaration sur les implications éthiques de cette recherche. Il doit préciser que les principes énoncés dans la présente déclaration sont respectés.

15. Les études sur l'être humain doivent être conduites par des personnes scientifiquement qualifiées et sous le contrôle d'un médecin compétent. La responsabilité à l'égard d'un sujet inclus dans une recherche doit toujours incomber à une personne médicalement qualifiée et non au sujet, même consentant.

16. Toute étude doit être précédée d'une évaluation soigneuse du rapport entre d'une part, les risques et les contraintes et d'autre part, les avantages prévisibles pour le sujet ou d'autres personnes. Cela n'empêche pas la participation à des recherches médicales de volontaires sains. Le plan de toutes les études doit être accessible.

17. Un médecin ne doit entreprendre une étude que s'il estime que les risques sont correctement évalués et qu'ils peuvent être contrôlés de manière satisfaisante. Il doit être mis un terme à la recherche si les risques se révèlent l'emporter sur les bénéfices escomptés ou si des preuves consistantes de résultats positifs et bénéfiques sont apportées.

18. Une étude ne peut être réalisée que si l'importance de l'objectif recherché prévaut sur les contraintes et les risques encourus par le sujet. C'est particulièrement le cas lorsqu'il s'agit d'un volontaire sain.

19. Une recherche médicale sur des êtres humains n'est légitime que si les populations au sein desquelles elle est menée ont des chances réelles de bénéficier des résultats obtenus.

20. Les sujets se prêtant à des recherches médicales doivent être des volontaires informés des modalités de leur participation au projet de recherche.

21. Le droit du sujet à la protection de son intégrité doit toujours être respecté. Toutes précautions doivent être prises pour respecter la vie privée du sujet, la confidentialité des données le concernant et limiter les répercussions de l'étude sur son équilibre physique et psychologique.

22. Lors de toute étude, la personne se prêtant à la recherche doit être informée de manière appropriée des objectifs, méthodes, financement, conflits d'intérêts éventuels, appartenance de l'investigateur à une ou des institutions, bénéfices attendus ainsi que des risques potentiels de l'étude et des contraintes qui pourraient en résulter pour elle. Le sujet doit être informé qu'il a la faculté de ne pas participer à l'étude et qu'il est libre de revenir à tout moment sur son consentement sans crainte de préjudice. Après s'être assuré de la bonne compréhension par le sujet de l'information donnée, le médecin doit obtenir son consentement libre et éclairé, de préférence par écrit. Lorsque le consentement ne peut être obtenu sous forme écrite, la procédure de recueil doit être formellement explicitée et reposer sur l'intervention de témoins.

23. Lorsqu'il sollicite le consentement éclairé d'une personne à un projet de recherche, l'investigateur doit être particulièrement prudent si le sujet se trouve vis-à-vis de lui dans une situation de dépendance ou est exposé à donner son consentement sous une forme de contrainte. Il est alors souhaitable que le consentement soit sollicité par un médecin bien informé de l'étude mais n'y prenant pas part et non concerné par la relation sujet-investigateur.

24. Lorsque le sujet pressenti est juridiquement incapable, physiquement ou mentalement hors d'état de donner son consentement ou lorsqu'il s'agit d'un sujet mineur, l'investigateur doit obtenir le consentement éclairé du représentant légal en conformité avec le droit en vigueur. Ces personnes ne peuvent être inclues dans une étude que si celle-ci est indispensable à l'amélioration de la santé de la population à laquelle elles appartiennent et ne peut être réalisée sur des personnes aptes à donner un consentement.

25. Lorsque le sujet, bien que juridiquement incapable (un mineur par exemple), est cependant en mesure d'exprimer son accord à la participation à l'étude, l'investigateur doit obtenir que cet accord accompagne celui du représentant légal.

26. La recherche sur des personnes dont il est impossible d'obtenir le consentement éclairé, même sous forme de procuration ou d'expression préalable d'un accord, ne doit être conduite que si l'état physique ou mental qui fait obstacle à l'obtention de ce consentement est une des caractéristiques requises des sujets à inclure dans l'étude. Les raisons spécifiques d'inclure des sujets dans une étude en dépit de leur incapacité à donner un consentement éclairé doivent être exposées dans le protocole qui sera soumis au comité pour examen et approbation. Le protocole doit également préciser que le consentement du sujet ou de son représentant légal à maintenir sa participation à l'étude doit être obtenu le plus rapidement possible.

27. Les auteurs et les éditeurs de publications scientifiques ont des obligations d'ordre éthique. Lors de la publication des résultats d'une étude, les investigateurs doivent veiller à l'exactitude des résultats. Les résultats négatifs aussi bien que les résultats positifs doivent être publiés ou rendus accessibles. Le financement, l'appartenance à une ou des institutions et les éventuels conflits d'intérêt doivent être exposés dans les publications. Le compte-rendu d'une étude non conforme aux principes énoncés dans cette déclaration ne doit pas être accepté pour publication.

**C. PRINCIPES APPLICABLES A LA RECHERCHE MEDICALE CONDUITE AU**

**COURS D'UN TRAITEMENT**

28. Le médecin ne peut mener une recherche médicale au cours d'un traitement que dans la mesure où cette recherche est justifiée par un possible intérêt diagnostique, thérapeutique ou de prévention. Quand la recherche est associée à des soins médicaux, les patients se prêtant à la recherche doivent bénéficier de règles supplémentaires de protection.

29. Les avantages, les risques, les contraintes et l'efficacité d'une nouvelle méthode doivent être évalués par comparaison avec les meilleures méthodes diagnostiques, thérapeutiques ou de prévention en usage. Cela n'exclut ni le recours au placebo ni l'absence d'intervention dans les études pour lesquelles il n'existe pas de méthode diagnostique, thérapeutique ou de prévention éprouvée.1

30. Tous les patients ayant participé à une étude doivent être assurés de bénéficier à son terme des moyens diagnostiques, thérapeutiques et de prévention dont l'étude aura montré la supériorité.2

31. Le médecin doit donner au patient une information complète sur les aspects des soins qui sont liés à des dispositions particulières du protocole de recherche. Le refus d'un patient de participer à une étude ne devra en aucun cas porter atteinte aux relations que le médecin entretient avec ce patient.

32. Lorsqu'au cours d'un traitement, les méthodes établies de prévention, de diagnostic ou de thérapeutique s'avèrent inexistantes ou insuffisamment efficaces, le médecin, avec le consentement éclairé du patient, doit pouvoir recourir à des méthodes non éprouvées ou nouvelles s'il juge que celles-ci offrent un espoir de sauver la vie, de rétablir la santé ou de soulager les souffrances du malade. Ces mesures doivent, dans toute la mesure du possible, faire l'objet d'une recherche destinée à évaluer leur sécurité et leur efficacité. Toute nouvelle information sera consignée et, le cas échéant, publiée. Les autres recommandations appropriées énoncées dans la présente déclaration s'appliquent.

1 **Note explicative concernant le paragraphe 29**

## L'AMM note avec préoccupation que le paragraphe 29 de la Déclaration d'Helsinki (Octobre 2000) est l'objet d'interprétations diverses et de possibles malentendus. Elle réaffirme par ailleurs que les essais avec témoins sous placebo ne doivent être utilisés qu'avec de grandes précautions et, d'une façon générale, lorsqu'il n'existe pas de traitement éprouvé. Toutefois, même s'il existe un traitement éprouvé, les essais avec témoins sous placebo peuvent être éthiquement acceptables dans les conditions suivantes:

• lorsque, pour des raisons méthodologiques impérieuses et scientifiquement solides, il n'existe pas d'autres moyens qui permettent de déterminer l'efficacité ou l'innocuité d'une méthode prophylactique, diagnostique ou thérapeutique ; ou

• lorsqu'une méthode prophylactique, diagnostique ou thérapeutique est mise à l'essai pour une affection bénigne et que la participation à l'essai n'expose pas à des risques supplémentaires de dommages significatifs ou durables.

Toutes les dispositions énoncées dans la DoH doivent être respectées, en particulier, la nécessité d'un examen éthique et scientifique approfondi.

2 **Note explicative concernant le paragraphe 30**

Par la présente, l'AMM réaffirme la nécessité d'identifier, lors de la planification d'une étude, l'accès post-étude pour les participants aux procédures prophylactiques, diagnostiques et thérapeutiques considérées comme bénéfiques par cette même étude ou un accès à d'autres soins

appropriés. Les dispositions prises pour un tel accès ou pour d'autres soins doivent être décrites dans le protocole d'étude afin que le comité de révision éthique puisse étudier ces dispositions.

*La déclaration d'Helsinki (Document 17.C) est un document officiel de l'Association médicale mondiale,représentante des médecins dans le monde. Adoptée en 1964 à Helsinki (Finlande), elle fut révisée en 1975 à Tokyo (Japon), en 1983 à Venise (Italie), en 1989 à Hong Kong, en 1996 à Somerset West (Afrique du Sud), en 2000 à Edimbourg (Ecosse), par l'Assemblée générale de l'AMM, Washington 2002 (addition d'une note explicative concernant le paragraphe 29), et par l'Assemblée générale de l'AMM, Tokyo 2004 (addition d'une note explicative concernant le paragraphe 30).*

b) FINAL PROTOCOL

(Incorporating all 12 amendments)

**ESSAI THERAPEUTIQUE RANDOMISE MULTICENTRIQUE**

**EN DOUBLE INSU EN GROUPES PARALLELES DU**

**CYCLOPHOSPHAMIDE INTRAVEINEUX VERSUS METHYLPREDNISOLONE DANS LE TRAITEMENT DE FOND DES FORMES PROGRESSIVES SECONDAIRES RECENTES DE SCLEROSE EN PLAQUES : ETUDE P.R.O.M.E.S.S**

Titre abrégé : Essai randomisé multicentrique en double insu cyclophosphamide versus méthylprednisolone dans le traitement des formes progressives secondaires de sclérose en plaques : Etude P.R.O.M.E.S.S.

Version 8.0 du 12/11/2009

Promoteur :

CHU de Bordeaux, Direction Générale du CHU de Bordeaux,

Unité de gestion de la recherche clinique

12 rue Dubernat, 33404 Talence

Tel : 05 57 82 03 13 Fax 05 56 79 49 26

Site internet : [www.chu-bordeaux.fr](http://www.chu-bordeaux.fr/)

Investigateur Coordonnateur :

Pr Bruno Brochet,

Département de Neurologie

Fédération des Neurosciences Cliniques, CHU de Bordeaux.

Hôpital Pellegrin CHU de Bordeaux.

Place Amélie Raba Leon, 33076 Bordeaux cedex

Tel 05 56 79 55 21 Fax 05 56 79 60 25

[bruno.brochet@chu-bordeaux.fr](mailto:bruno.brochet@chu-bordeaux.fr)

**II. LISTE DES PRINCIPAUX RESPONSABLES POUR LE PROTOCOLE :**

**Promoteur** : CHU de Bordeaux, 12 rue Dubernat, 33400 Talence.

**Investigateur Coordonnateur** : Pr Bruno Brochet, Département de Neurologie du CHU de Bordeaux, Fédération des Neurosciences Cliniques, Hôpital Pellegrin, Place Amélie Raba Leon, 33076 Bordeaux cedex.

Tél. : 05 56 79 55 21

Fax : 05 56 79 49 51

Email : [bruno.brochet@chu-bordeaux.fr](mailto:bruno.brochet@chu-bordeaux.fr)

**Méthodologiste Coordonnateur** : Dr Paul Perez, USMR du CHU de Bordeaux, Bât

ISPED, Université Victor Segalen, 146 rue Léo Saignat, Case 11, 33076 Bordeaux Cedex.

Tél.: 05 57 57 11 29

Fax : 05 57 57 15 78

Email : [usmr@isped.u-bordeaux2.fr](mailto:usmr@isped.u-bordeaux2.fr)

**Responsable Pharmacie, coordonnateur** : Dr Jean Grellet, Pharmacie centrale, CHU de Bordeaux, 33076 Bordeaux Cedex.

Tél.: 05 56 79 55 03

Fax : 05 56 79 56 74

Email : [jean.grellet@chu-bordeaux.fr](mailto:jean.grellet@chu-bordeaux.fr)

**Centre Coordonnateur :**

**Médecin prescripteur :** Dr Pierre Louiset, Neurologue, Clinique Saint Augustin, Unité de Neurologie, 114 Avenue d’Arès, 33074 Bordeaux Cedex.

Tél.: 05 56 24 20 24

Fax : 05 56 99 55 00

Email : pierre.louiset@modulonet.fr

**Chef de projet:** Mathilde Deloire, [mathilde.grassin@bb-luni.u-bordeaux2.fr](mailto:mathilde.grassin@bb-luni.u-bordeaux2.fr)

**Attaché de recherche clinique** : Timothé Loock, timothe.loock@hotmail.fr

Département de Neurologie Pr Brochet,

146 rue Léo Saignat, case 78, 33076 Bordeaux Cedex.

Tél. : 05 57 57 48 17 ; Fax : 05 57 57 48 18

Liste des centres, des investigateurs principaux et co-investigateurs :

| **ANGOULEME** | | Centre N^o^  **01** | CH d’Angoulême Girac, Rte de Bordeaux16470 Saint Michel. | | | |
| --- | --- | --- | --- | --- | --- | --- |
| **Investigateur**  **Principal** | **Dr DEVOIZE Jean Louis** | | | [jeanlouis.devoize@ch-angouleme.rss.fr](mailto:jeanlouis.devoize@ch-angouleme.fr).  Tel : 05 45 24 40 84 Fax : 05 45 24 60 90 | | 16/1035 |
| **Investigateurs** | (NT) Dr PIN Jean Christophe | | | [jeanchristophe.pin@changouleme.fr](mailto:jeanchristophe.pin@changouleme.fr) | | 16/1453 |
|  | (NT) Dr PRAT Christophe | | | [christophe.prat@ch-angouleme.fr](mailto:christophe.prat@ch-angouleme.fr). | | 16/1644 |
|  | (NE) Dr SHAROV Igor | | | igorcharov@hotmail.com | | En cours |
| **Pharmacien(s)** | Dr CANCEL | | | | [dominique.cancel@ch-angouleme.fr](mailto:dominique.cancel@ch-angouleme.fr) | |
|  | Dr Girard Anne et Franck | | | | franck.girard@ch-anouleme.fr | |
|  | Tel : 05 45 24 42 22 Fax : 05 45 24 61 28 | | | | | |

| **BAYONNE** | | Centre N^o^  **02** | CH de la Cote Basque, 13 avenue de l’Interne Jacques Löeb, BP 8, 64109 Bayonne Cedex. | | | |
| --- | --- | --- | --- | --- | --- | --- |
| **Investigateur**  **Principal** | **Dr ELLIE Emmanuel** | | | [eellie001@chicb.com](mailto:eellie001@chicb.com).  Tel : 05 59 44 37 12 Fax : 05 59 44 37 19 | | 64/4003 |
| **Investigateurs** | (NT) Dr BALLAN Guillaume | | | [gballan001@chicb.com](mailto:gballan001@chicb.com) | | 64/5445 |
|  | (NE) Dr BERNADY Patricia | | | [pbernady001@chicb.com](../../../../../Mes%20documents/Mes%20fichiers%20reçus/pbernady001@chicb.com) | | 64/4353 |
|  | (NE) Dr RUMMENS Catherine | | | crummens001@chicb.com | | 64/2808 |
| **Pharmacien(s)** | Dr BURTIN Christophe | | | | cburtin001@chicb.com | |
|  | Dr CHAMBON Anne | | | | achambon@chicb.com | |
|  | Tel : 05 59 44 35 60 Fax : 05 59 44 35 59 | | | | | |

| **BESANÇON** | | Centre N^o^  **03** | (CHU Besançon) Hôpital Jean Minjoz, 3 Boulevard Fleming  25030 Besançon Cedex. | | | |
| --- | --- | --- | --- | --- | --- | --- |
| **Investigateur**  **Principal** | **Pr RUMBACH Lucien** | | | [lrumbach@chu-besancon.fr](mailto:lrumbach@chu-besancon.fr).  Tel : 03 81 66 80 98 Fax : 03 81 66 84 70 | | 06/1980 |
| **Investigateurs** | (NT) Dr MEDEIROS DE  BUSTOS Elisabeth | | |  | | 25/4012 |
|  | (NE) Dr BERGER Eric | | | [eric.berger@ufc-chu.univ-fcomte.fr](mailto:eric.berger@ufc-chu.univ-fcomte.fr). | | 25/03197 |
| **Pharmacien(s)** | Dr JACQUET | | | | [mjacquet@chu-besancon.fr](mailto:mjacquet@chu-besancon.fr) | |
|  | Dr ESSERT Michelle | | | | messert@chu-besancon.fr | |
|  | Dr FAGNONI-LEGAT Christine | | | | clegat@chu-besancon.fr | |
|  | Tel : 03 81 66 89 03 Fax : 03 81 66 84 89 | | | | | |

| **BORDEAUX** | | Centre N^o^  **04** | (CHU Bordeaux) Hôpital Pellegrin, 1 place Amélie Raba Léon  33076 Bordeaux Cedex. | | | |
| --- | --- | --- | --- | --- | --- | --- |
| **Investigateur**  **principal** | **Pr BROCHET Bruno** | | | [bruno.brochet@chu-bordeaux.fr](mailto:bruno.brochet@chu-bordeaux.fr).  Tel : 05 56 79 55 21 Fax : 05 56 79 49 51 | | 33/7618 |
| **Investigateurs** | (NT) DEBRUXELLES Sabrina | | | sabrina.debruxelles@club-internet.fr | | En cours |
|  | (NE) Dr JEANNIN Séverine | | | samfaitrien@hotmail.com | | En cours |
|  | (NE) Dr OUALLET Jean Christophe | | | jean-christophe.ouallet@chu-bordeaux.fr | | 33/11914 |
| **Pharmacien(s)** | Dr GRELLET Jean | | | | [jean.grellet@chu-bordeaux.fr](mailto:jean.grellet@chu-bordeaux.fr) | |
|  | Dr JOURAND Audrey | | | | audrey.jourand@chu-bordeaux.fr | |
|  | Tel : 05 56 79 55 03 Fax : 05 57 82 08 25 | | | | | |

| **CAEN** | | Centre N^o^  **05** | (CHU Caen) Hôpital de la Cote de Nacre, Avenue de la Cote de Nacre, 14033 Caen Cedex. | | | |
| --- | --- | --- | --- | --- | --- | --- |
| **Investigateur**  **Principal** | **Pr DEFER Gilles** | | | [defer-gi@chu-caen.fr](mailto:defer-gi@chu-caen.fr).  Tel : 02 31 06 46 21 Fax : 02 31 06 46 27 | | 14/3587 |
| **Investigateurs** | (NT) Dr DERACHE Nathalie | | | sderache@free.fr | | En cours |
|  | (NE) Dr JAILLON Valérie | | | [rbn-sep@wanadoo.fr](../../../../../Mes%20documents/Mes%20fichiers%20reçus/rbn-sep@wanadoo.fr) | | 14/4608 |
| **Pharmacien(s)** | Dr OLLIVIER | | | | [ollivier-c@chu-caen.fr](mailto:ollivier-c@chu-caen.fr) | |
|  | Tel : 02 31 27 23 52 Fax : 02 31 27 26 32 | | | | | |

| **CLERMONT** | | Centre N^o^  **06** | Hôpital Gabriel Montpied, 58 rue Montalembert, BP 69  63003 Clermont Ferrand Cedex 1. | | | | |
| --- | --- | --- | --- | --- | --- | --- | --- |
| **Investigateur**  **Principal** | **Pr CLAVELOU Pierre** | | | [pclavelou@chu-clermontferrand.fr](mailto:pclavelou@chu-clermontferrand.fr).  Tel : 04 73 75 22 01 Fax : 04 73 75 22 02 | | | 63/3055 |
| **Investigateurs** | (NT) Dr THAITE Frédéric | | | [ftaithe@chu-clermontferrand.fr](mailto:pclavelou@chu-clermontferrand.fr). | | | 63/5272 |
|  | (NT) Dr AUFAUVRE Dominique | | | | [daufauvre@chu-clermontferrand.fr](mailto:daufauvre@chu-clermontferrand.fr) | | 63/4175 |
|  | (NE) Dr FERRIER Anna | | | [aferrier@chu-clermontferrand.fr](mailto:aferrier@chu-clermontferrand.fr) | | | 63/5140 |
|  | (NE) Dr GUY Nathalie | | | [nguy@chu-clermontferrand.fr](mailto:nguy@chu-clermontferrand.fr) | | | 63/4890 |
|  | (NE) Dr DIONET Elsa | | | edionet@chu-clermontferrand.fr | | | 63/5672 |
| **Pharmacien(s)** | Dr CHOPINEAU | | | | |  | |
|  | Dr BAER Martine | | | | | mbaer@chu-clermontferrand.fr | |
|  | Tel : 04 73 75 17 60 Fax : 04 73 75 17 59 | | | | | | |

| **CRETEIL** | | Centre N^o^  **07** | (AP HP) Henri Mondor, 51, avenue du Maréchal de Lattre de Tassigny, 94010 Créteil cedex. | | | |
| --- | --- | --- | --- | --- | --- | --- |
| **Investigateur**  **Principal** | **Pr CREANGE Alain (NE)** | | | [creange@univ-paris12.fr](mailto:creange@univ-paris12.fr).  Tel : 01 49 81 23 04 Fax : 01 49 81 23 26 | | 94/10016 |
| **Investigateurs** | (NE) Dr GUEGUEN Antoine | | | antoinegueguen@yahoo.fr | |  |
|  | Dr NINEB Amine | | |  | |  |
| **Pharmacien(s)** | CARVALHO Muriel | | | | muriel.verlinde-carvalho@hmn.aphp.fr | |
|  | DO-VAN-LAHN Nathalie | | | |  | |
|  | Tel : 01 49 81 27 59 Fax : 01 49 81 27 70 | | | | | |

| **DIJON** | | Centre N^o^  **08** | (CHU Dijon) Hôpital Général, 3 rue du faubourg Raines  21033 Dijon Cedex. | | |
| --- | --- | --- | --- | --- | --- |
| **Investigateur**  **Principal** | **Pr MOREAU Thibault** | | | [thibault.moreau@chu-dijon.fr](mailto:thibault.moreau@chu-dijon.fr).  Tel : 03 80 29 37 53 Fax : 03 80 29 36 72 | 21/3932 |
| **Investigateurs** | (NT) Dr COUVREUR Grégory | | | [gregory.couvreur@chu-dijon.fr](mailto:gregory.couvreur@chu-dijon.fr). | 21/1040662 |
|  | (NE) Dr BENATRU Isabelle | | | [isabelle.benatru@chu-dijon.fr](mailto:isabelle.benatru@chu-dijon.fr). | 21/1043856 |
| **Pharmacien(s)** | Mme GUIGNARD Marie-Hélène | | | | |
|  | Tel : 03 80 29 37 67/ 03 80 29 53 64 Fax : 03 80 29 32 75 / 03 80 29 38 76 | | | | |

| **LILLE (CHU)** | | Centre N^o^  **09** | (CHU Lille) Hôpital Salengro, Rue du Pr. Emile Laine  59037 Lille Cedex. | | | |
| --- | --- | --- | --- | --- | --- | --- |
| **Investigateur**  **Principal** | **Pr VERMERSCH Patrick** | | | [pvermersch@chru-lille.fr](mailto:pvermersch@chru-lille.fr).  Tél :03 20 44 57 65 Fax : 03 20 44 44 84 | | 59/13487 |
| **Investigateurs** | (NT) Dr STOJKOVIC Tanya | | | [t-s4012tojkovic@chru-lille.fr](mailto:t-stojkovic@chru-lille.fr). | | 59/15356 |
|  | (NE) Dr ZEPHIR Hélène | | | [h-zephir@chru-lille.fr](mailto:h-zephir@chru-lille.fr). | | 59/18442 |
|  | (NE) Dr LACOUR Arnaud | | | [a-lacour@chru-lille.fr](../../../../../Mes%20documents/Mes%20fichiers%20reçus/a-lacour@chru-lille.fr) | | En cours |
|  | Dr WAUCQUIER Nawal | | | n-waucquieràchru-lille.fr | |  |
| **Pharmacien(s)** | Dr YILMAZ Monique | | | | myilmaz@chru-lille.fr | |
|  | Dr THIELMANS Béatrice | | | | bthielmans@chru-lille.fr | |
|  | Tel : 03 20 44 42 31 / 03 20 44 62 57 Fax : 03 20 44 59 60 | | | | | |

| **LILLE (GHICL)** | | Centre N^o^  **10** | (GHICL) Hôpital St. Philibert, 115 Rue du Grand But, BP 249  59462 Lomme Cedex. | | | | |
| --- | --- | --- | --- | --- | --- | --- | --- |
| **Investigateur**  **Principal** | **Pr HAUTECOEUR Patrick** | | | [Hautecoeur.Patrick@ghicl.fupl.asso.fr](mailto:Hautecoeur.Patrick@ghicl.fupl.asso.fr).  Tel : 02 20 22 50 79 Fax : 03 20 22 38 55 | | | 59/11815 |
| **Investigateurs** | (NT) Dr MACKOWIAK Alexandre | | | | [mackowiak.alexandre@ghicl.net](mailto:mackowiak.alexandre@ghicl.net) | | 59/17072 |
|  | (NE) Dr MEYNIEU Philippe | | | [meynieu.philippe@ghicl.net](mailto:meynieu.philippe@ghicl.net) | | | 59/17973 |
| **Pharmacien(s)** | Dr BOURY | | | | | [boury.eric@ghicl.net](mailto:boury.eric@ghicl.net) | |
|  | Mr FLORET | | | | |  | |
|  | Tel : 03 20 22 50 16 Fax : 03 20 22 81 71 | | | | | | |

| **LIMOGES** | | Centre N^o^  **11** | CHU Limoges, 2 Av Martin Luther King  87042 Limoges Cedex. | | | |
| --- | --- | --- | --- | --- | --- | --- |
| **Investigateur**  **Principal** | **Dr MAGY Laurent** | | | [laurent.magy@unilim.fr](mailto:laurent.magy@unilim.fr).  Tel : 05 55 05 65 61 Fax : 05 55 05 65 67 | | 87/2777 |
| **Investigateurs** | (NT) Pr VALLAT Jean Michel | | | [vallat@unilim.fr](mailto:vallat@unilim.fr). | | 87/1009239 |
|  | (NE) Dr LACOSTE Matthieu | | |  | | 87/3236 |
| **Pharmacien(s)** | Mlle JAVERLIAT | | | | [essclin-pharm@chu-limoges.fr](mailto:essclin-pharm@chu-limoges.fr) | |
|  | Tel : 05 55 05 61 55 Fax : 05 55 05 61 57 | | | | | |

| **LYON** | | Centre N^o^  **12** | (CHU Lyon) Hôpital neurologique Pierre Wertheimer, 56 Bd Pinel  69394 Lyon Cedex 03 | | | |
| --- | --- | --- | --- | --- | --- | --- |
| **Investigateur**  **Principal** | **Pr CONFAVREUX**  **Christian** | | | [christian.confavreux@chiu-lyon.fr](mailto:christian.confavreux@chiu-lyon.fr).  Tel : 04 72 35 75 22 Fax : 04 72 35 75 25 | | 69/05474 |
| **Investigateurs** | (NT) Dr BLANC Sandrine | | | [sandrine.blanc@chiu-lyon.fr](mailto:sandrine.blanc@chiu-lyon.fr). | | 69/11560 |
|  | (NT) Dr RICHE Georges | | | [georges.riche@chiu-lyon.fr](mailto:georges.riche@chiu-lyon.fr). | | 69/10955 |
|  | (NE) Dr AUBERTIN Pierre | | | [pierre.aubertin@chiu-lyon.fr](mailto:pierre.aubertin@chiu-lyon.fr). | | 69/1112361 |
| **Pharmacien(s)** | Mr MARTIN | | | | [Patrice.martin@chu-lyon.fr](mailto:Patrice.martin@chu-lyon.fr) | |
|  | Tel : 04 72 35 71 03 Fax : | | | | | |

| **MARSEILLE** | | Centre N^o^  **13** | Hôpital La Timone, Bd Jean Moulin  13385 Marseille cedex. | | | |
| --- | --- | --- | --- | --- | --- | --- |
| **Investigateur**  **Principal** | **Pr PELLETIER Jean (NE)** | | | [jean.pelletier@ap-hm.fr](mailto:jean.pelletier@ap-hm.fr).  Tel : 04 91 38 59 39 Fax : 04 91 38 62 56 | | 13/15573 |
| **Investigateurs** | Dr AUDOIN Bertrand (NT) | | | [bertrand.audoin@ap-hm.fr](../../../../../Mes%20documents/Mes%20fichiers%20reçus/bertrand.audoin@ap-hm.fr) | | 13/20318 |
| **Pharmacien(s)** | Pr BRAGUER Diane | | | | diane.braguer@pharmacie.univ-mrs.fr | |
|  | HONORE Stephane | | | | stephane.honore@mail.ap-hm.fr | |
|  | Tel : 04 91 83 56 35 Fax : | | | | | |

| **NANCY** | | Centre N^o^  **14** | (CHU Nancy) Hôpital central, 26 Av Maréchal de Lattre de Tassigny, CO n^o^34, 54035 Nancy Cedex. | | | | |
| --- | --- | --- | --- | --- | --- | --- | --- |
| **Investigateur**  **Principal** | **Dr DEBOUVERIE Marc** | | | m.[debouverie@chu-nancy.fr](mailto:debouverie@chu-nancy.fr).  Tel : 03 83 85 12 75 Fax : 03 83 85 27 34 | | | 54/4948 |
| **Investigateurs** | (NT) Dr PITTION Sophie | | | s.[pittion@chu-nancy.fr](mailto:pittion@chu-nancy.fr). | | | 54/6973 |
|  | (NE) Dr LACOUR Jean Christophe | | | | jc.[lacour@chu-nancy.fr](mailto:lacour@chu-nancy.fr). | | 54/5470 |
|  | (NE) Dr LOUIS Sarah | | | s.[louis@chu-nancy.fr](mailto:louis@chu-nancy.fr) | | | 54/6830 |
|  | (NE) Dr GOSPODARU Razvan Nicolaie | | | rgospodaru@gmail.com | | | 54/7359 |
|  | (NE) Dr LANOTTE Livia | | | lorsep.lanotte@orange.fr | | | 54/7560 |
| **Pharmacien(s)** | Mme BIGEARD | | | | | c.bigeard@chu-nancy.fr | |
|  | Mme COMMUN | | | | | n.commun@ chu-nancy.fr | |
|  | Tel : 03 83 85 14 88 Fax : 03 83 85 22 06 | | | | | | |

| **NICE** | | Centre N^o^  **15** | (CHU Nice) Hôpital Pasteur, 30 Av de la Voie Romaine, BP69  06002 Nice Cedex. | | | |
| --- | --- | --- | --- | --- | --- | --- |
| **Investigateur**  **Principal** | **Dr LEBRUN-FRENAY**  **Christine (NE)** | | | [christine.lebrun-frenay@wanadoo.fr](mailto:christine.lebrun-frenay@wanadoo.fr).  Tel : 04 92 03 77 44 Fax : 04 92 03 79 07 | | 06/08554 |
| **Investigateurs** | Dr BAYREUTHER caroline | | |  | | 06/10776 |
|  | Dr LACHAUD Sylvain | | |  | | 06/10665 |
|  | Dr BEDOUCHA Pierre | | |  | | 06/3113 |
| **Pharmacien(s)** | Dr DUFRENE WOTAWA Anne | | | | wotawa.a@chu.nice.fr | |
|  | Tel : 04 92 03 77 44 Fax : 04 92 03 76 94 | | | | | |

| **NIMES** | | Centre N^o^  **16** | (CHU Nîmes) Hôpital Caremeau, Place du Professeur Robert Debré,  30029 Nîmes Cedex 4. | | | |
| --- | --- | --- | --- | --- | --- | --- |
| **Investigateur**  **Principal** | **Dr CASTELNOVO**  **Giovanni (NE)** | | | [giovanni.castelnovo@chu-nimes.fr](mailto:giovanni.castelnovo@chu-nimes.fr).  Tel : 04 66 68 32 63 Fax : 04 66 68 37 33 | | 30/3221 |
| **Investigateurs** | (NT) Dr LABAUGE Pierre | | | [pierre.labauge@chu-nimes.fr](mailto:pierre.labauge@chu-nimes.fr). | | 31/130 |
|  | (NT) LE BAYON Alice | | | alice.lebayon@chu-nimes.fr | | En cours |
|  | (NT) Dr LE FLOCH Anne | | | annelefloch@yahoo.fr | | 30/4604 |
| **Pharmacien(s)** | Mr MAILLARD Christian | | | | [christian.maillard@chu-nimes.fr](../../../../../Mes%20documents/Mes%20fichiers%20reçus/christian.maillard@chu-nimes.fr) | |
|  | Mme FAVIER | | | | essais.cliniques@chu-nimes.fr | |
|  | Mme EYSSETTE Bernadette | | | |  |  |
|  | Tel : 04 66 68 32 92 / 04 66 68 34 22 Fax : 04 66 68 33 28 | | | | | |

| **PAU** | | Centre N^o^  **17** | Centre Hospitalier de Pau, 4 Bd Hauterive, BP 1156  64046 Pau Cedex. | | | |
| --- | --- | --- | --- | --- | --- | --- |
| **Investigateur**  **Principal** | **(NE) Dr LARRIEU Jean Marc** | | | [jean-marc.larrieu@chu-pau.fr](mailto:jean-marc.larrieu@chu-pau.fr).  Tel : 05 59 92 49 65 Fax : 05 59 92 67 49 | | 64/4711 |
| **Investigateurs** | (NT) Dr BARROSO Bruno | | | [bruno.barroso@ch-pau.fr](mailto:bruno.barroso@ch-pau.fr). | | 64/4863 |
| **Pharmacien(s)** | Mme FERRARI Sylvie | | | | [sylvie.ferrari@ch-pau.fr](mailto:sylvie.ferrari@ch-pau.fr) | |
|  | Mr JOMIER Jean Yves | | | | jean-yves.jomier@ch-pau.fr | |
|  | Tel : 05 59 92 48 63 Fax : 05 59 72 67 26 | | | | | |

| **REIMS** | | Centre N^o^  **18** | (CHU Reims) Hôpital Robert Debré, Av du Général Koenig  51092 Reims cedex. | | | | |
| --- | --- | --- | --- | --- | --- | --- | --- |
| **Investigateur**  **Principal** | **Pr BAKCHINE Serge (NE)** | | | [sbakchine@chu-reims.fr](mailto:sbakchine@chu-reims.fr).  Tel : 03 26 78 71 35 Fax : 03 26 78 43 16 | | | 51/3796 |
| **Investigateurs** | (NT) Dr CHAUNU Marie Pierre | | | | [mpchaunu@chu-reims.fr](mailto:mpchaunu@chu-reims.fr). | | 51/2960 |
| **Pharmacien(s)** | Mme DUFRECHOU Nathalie | | | | | ndufrechou@chu-reims.fr | |
|  | Mr GOURDIER | | | | | [bgourdier@chu-reims.fr](mailto:bgourdier@chu-reims.fr) | |
|  | Tel : 03 26 78 39 87 Fax : 03 26 78 85 29 | | | | | | |

| **RENNES** | | Centre N^o^  **19** | CHU Ponchaillou, 2 rue Henri Le Guilloux  35033 Rennes cedex 9. | | | | | |
| --- | --- | --- | --- | --- | --- | --- | --- | --- |
| **Investigateur**  **Principal** | **Pr EDAN Gilles** | | | [gilles.edan@chu-rennes.fr](mailto:gilles.edan@chu-rennes.fr).  Tel : 02 99 28 94 55 Fax : 02 99 28 41 32 | | | | 35/2194 |
| **Investigateurs** | (NT) Dr LEPAGE Emmanuelle | | | | [emmanuelle.lepage@chu-rennes.fr](mailto:emmanuelle.lepage@chu-rennes.fr). | | | 35/5781 |
|  | (NE) Dr YAOUANQ Jacqueline | | | | | [jacqueline.yaouanq@chu-rennes.fr](mailto:jacqueline.yaouanq@chu-rennes.fr). | | 35/02991 |
| **Pharmacien(s)** | Dr JAVAUDIN | | | | | | [Loic.javaudin@chu-rennes.fr](mailto:Loic.javaudin@chu-rennes.fr) | |
|  | Tel : 02 99 28 42 51 Fax : 02 99 28 41 51 | | | | | | | |

| **ROTHSCHILD** | | Centre N^o^  **20** | Fondation Rothschild, 25 rue Manin  75019 Paris Cedex 20. | | | |
| --- | --- | --- | --- | --- | --- | --- |
| **Investigateur**  **Principal** | **Dr GOUT Olivier** | | | [ogout@fo-rothschild.fr](mailto:ogout@fo-rothschild.fr).  Tel : 01 48 03 68 52 Fax : 01 48 03 27 01 | | 75/57183 |
| **Investigateurs** | (NT) Dr DESCHAMPS Romain | | | | [rdeschamps@fo-rothschild.fr](mailto:rdeschamps@fo-rothschild.fr). | 75/69792 |
|  | (NE) Dr LE CANUET Pierre | | | [plecanuet@fo-rothschild.fr](mailto:plecanuet@fo-rothschild.fr). | | 75/38205 |
|  | Dr MOULIGNIER Antoine | | |  | | 75/56393 |
| **Pharmacien(s)** | Mr BARKATZ | | | | | |
|  | Tel : 01 48 03 69 46 Fax : 01 48 03 69 44 | | | | | |

| **TENON** | | Centre N^o^  **21** | (AP HP) Hôpital Tenon, 4 rue la Chine  75970 Paris Cedex. | | | |
| --- | --- | --- | --- | --- | --- | --- |
| **Investigateur**  **Principal** | **Pr GIANNESINI Claire** | | | claire.giannesini@tnn.ap-hop-paris.fr  Tel : 01 56 01 66 52 Fax : 01 56 01 72 02 | | 75/71887 |
| **Investigateurs** | Pr ROULLET Etienne | | | [etienne.roullet@tnn.ap-hop-paris.fr](mailto:etienne.roullet@tnn.ap-hop-paris.fr). | | 75/40218 |
|  | Dr BENSA Caroline | | | [carobensa@netcourrier.com](mailto:carobensa@netcourrier.com) | | 75/70890 |
|  | Dr PEZ Dominique | | | [dominique.pez@wanadoo.fr](mailto:dominique.pez@wanadoo.fr) | | 75/37575 |
|  | Dr BENOIST Ludovic | | | [lbenoist@club-internet.fr](mailto:lbenoist@club-internet.fr) | | 60/4092 |
|  | Dr TEHINDRAZANARIVELO | | | [djacobaalain@aol.com](mailto:djacobaalain@aol.com) | | En cours |
| **Pharmacien(s)** | Mme BECKER Annie | | | | [annie.becker@tnn.ap-hop-paris.fr](mailto:annie.becker@tnn.ap-hop-paris.fr) | |
|  | Mme DEBRIX Isabelle | | | | isabelle.debrix@tnn.aphp.fr | |
|  | Tel : 01 56 01 68 89 Fax : 01 56 01 78 38 | | | | | |

| **TOULOUSE** | | Centre N^o^  **22** | (CHU Toulouse) Hôpital Purpan, Place du Docteur Baylac, TSA 40031, 31059 toulouse Cedex 9. | | | |
| --- | --- | --- | --- | --- | --- | --- |
| **Investigateur**  **Principal** | **Pr CLANET Michel** | | | [clanet@cict.fr](mailto:clanet@cict.fr).  Tel : 05 61 77 20 67 Fax : 05 61 77 94 43 | | 31/1038707 |
| **Investigateurs** | (NT) Dr VIALA Frédérique | | | [viala.f@chu-toulouse.fr](mailto:viala.f@chu-toulouse.fr). | | 31/9957 |
|  | (NE) Dr DECHAUMONT-  PALACIN Sophie | | | [S_dechaumont@yahoo.fr](../../../../../Mes%20documents/Mes%20fichiers%20reçus/S_dechaumont@yahoo.fr) | | 31/10768 |
| **Pharmacien(s)** | Mr CANONGE | | | | [canonge.jm@chu-toulouse.fr](mailto:canonge.jm@chu-toulouse.fr) | |
|  | Mr MICHENOT | | | | [michenot.f@chu-toulouse.fr](mailto:michenot.f@chu-toulouse.fr) | |
|  | Tel : 05 67 77 10 74 Fax : | | | | | |

| **STRASBOURG** | | Centre N^o^  **23** | (CHRU Starsbourg) Hôpital civil, 1 place de l’hôpital, BP 426  67091 Strasbourg Cedex. | | | |
| --- | --- | --- | --- | --- | --- | --- |
| **Investigateur**  **Principal** | **Dr DE SEZE Jérôme** | | | [jerome.de.seze@chru-strasbourg.fr](mailto:jerome.de.seze@chru-strasbourg.fr).  Tel : 03 88 11 62 29 Fax : 03 88 11 63 43 | | 59/16133 |
| **Investigateurs** | (NT) Dr FLEURY Marie | | | [marie-celine.fleury@chru-strasbourg.fr](mailto:marie-celine.fleury@chru-strasbourg.fr). | | 67/8372 |
|  | (NE) Dr STEINMETZ Gisèle | | | [gisele.steinmetz@chru-strasbourg.fr](mailto:gisele.steinmetz@chru-strasbourg.fr). | | 67/03930 |
|  | (NE) Dr BLANC Frédéric | | |  | | En cours |
|  | (NE) Dr COLLONGUES Nicolas | | | collonguesnicolas@yahoo.fr | | En cours |
| **Pharmacien(s)** | Mme HUTT | | | |  | |
|  | Mme BERETZ Laurence | | | | laurence.beretz@chru-strasbourg.fr | |
|  | Tel : 03 88 11 68 64 Fax : | | | | | |

| **METZ** | | Centre N^o^  **24** | (CHR Metz-Thionville) Hôpital Notre Dame de Bon Secours, 1 place Philippe de Vigneuilles, 57038 Metz cedex 01. | | | |
| --- | --- | --- | --- | --- | --- | --- |
| **Investigateur**  **Principal** | **(NT) Pr GODET Etienne** | | | [e.godet@chr-metz-thionville.rss.fr](../../../../../Mes%20documents/Mes%20fichiers%20reçus/e.godet@chr-metz-thionville.rss.fr)  Tel : 03 87 55 34 08 Fax : 03 87 55 34 05 | | 57/2061 |
| **Investigateurs** | (NE) Dr WAGNER Marc | | |  | | 57/4779 |
| **Pharmacien(s)** | Mr GUSTIN | | | | [b.gustin@chr-metz-thionville.rss.fr](../../../../../Mes%20documents/Mes%20fichiers%20reçus/b.gustin@chr-metz-thionville.rss.fr) | |
|  | Tel : 03 87 55 34 76 Fax : 03 87 55 39 68 | | | | | |

| **MONTPELLIER** | | Centre N^o^  **25** | (CHU Montpellier), Hôpital de Gui de Chauliac, 80 av Augustin Fliche, 34295 Montpellier Cedex 5. | | | | |
| --- | --- | --- | --- | --- | --- | --- | --- |
| **Investigateur**  **Principal** | **(NT) Dr CAMU William** | | | [w-camu@chu-montpellier.fr](mailto:w-camu@chu-montpellier.fr)  Tel : 04 67 33 78 22 Fax : 04 67 33 81 36 | | | 34/7819 |
| **Investigateurs** | (NE) Dr GARRIGUES Guillaume | | | |  | | 34/11257 |
|  | (NT) Dr Gaillard Nicolas | | | | nicola.gaillard@wanadoo.fr | | 34/11948 |
| **Pharmacien(s)** | Dr RAMBOURG | | | | | [i-roch_torreilles@chu-montpellier.fr](mailto:i-roch_torreilles@chu-montpellier.fr) | |
|  | Tel : 04 67 33 71 21 Fax : 04 67 33 70 49 | | | | | | |

| **POISSY** | | Centre N^o^  **26** | CHU de POISSY.,10, rue Champ Gaillard. 78300 POISSY | | | |
| --- | --- | --- | --- | --- | --- | --- |
| **Investigateur**  **Principal** | **(NT) Dr HEINZLEF**  **Olivier Guy** | | | Tel : 01 39 27 41 82  Fax : 01 39 27 47 89 | | 75 / 59413 |
| **Investigateurs** | (NE) Dr CAMBON Henri | | |  | | 78 / 10668 |
| **Pharmacien(s)** | STOLL Eric | | | | estoll@chi-poissy-st-germain.fr | |
|  | Tel : 01 39 27 47 13 Fax : 01 30 27 44 34 | | | | | |

| **NANTES** | | Centre N^o^  **27** | Hôpital Guillaume et René Laënnec, Boulevard Jacques Monod,  44093 Nantes cedex 1 | |
| --- | --- | --- | --- | --- |
| **Investigateur**  **Principal** | **Pr Damier Philippe** | | [philippe.damier@chu-nantes.fr](mailto:philippe.damier@chu-nantes.fr)  Tél. : 02.40.16.52.05 Fax. : 02.40.16.56.63 | N° 44 / 6797 |
| **Investigateurs** | Dr Laplaud David  Dr Wiertlewski Sandrine | | [david.laplaud@chu-nantes.fr](mailto:david.laplaud@chu-nantes.fr)  Tél. : 02.40.16.52.85 Fax. : 02.40.16.56.63  [sandrine.wiertlewski@chu-nantes.fr](mailto:sandrine.wiertlewski@chu-nantes.fr)  Tél. : 02.40.16.52.85 Fax. : 02.40.16.56.63 | N° 44 / 1078995  N° 44 / 108632 |
| **Pharmacien(s)** | Dr Rouiller-Furic Isabelle | | [isabelle.furic@chu-nantes.fr](mailto:isabelle.furic@chu-nantes.fr) | |
|  | Tél. : 02.40.16.55.36 Fax. : 02.40.16.55.37 | | | |

**III- SOMMAIRE**
I. PAGE DE COUVERTURE ET PAGE DE GARDE
II. LISTE DES PRINCIPAUX RESPONSABLES POUR LE PROTOCOLE
III. SOMMAIRE
IV. RESUME DU PROTOCOLE
V. TEXTE DU PROTOCOLE
1. JUSTIFICATION DE L’ETUDE 
1.1 SITUATION DU PROBLEME
1.1.1 INTRODUCTION
1.1.2 ETAT DES CONNAISSANCES SUR LES TRAITEMENTS ACTUELS DES FORMES SECONDAIREMENT PROGRESSIVES DE SCLEROSE EN PLAQUES
1.1.2.1. Traitements par interféron
1.1.2.2 Mitoxantrone 
1.1.2.3 Corticoïdes 
1.1.2.4 Traitement par cyclophosphamide 
1.1.2.4.1. Données d’efficacité 
1.1.2.4.2 Données de tolérance 
1.1.2.4.3 Retombées attendues de l’étude 
1.2 Description des traitements à l’essai 
1.2.1 Traitements à l’essai 
1.2.2 Intérêt par rapport aux traitements disponibles 
1.2.2.1 L’interféron bêta 
1.2.2.2 Mitoxantrone 
1.2.2.3 Corticoïdes 
1.3. Hypothèse testée 
1.4 Choix méthodologiques 
1.4.1 Traitement évalué 
1.4.1.1 Choix 
1.4.1.2 Choix de la dose 
1.4.2. Choix du Traitement de comparaison 
1.4.2.1.Existe-il un traitement de référence des formes SP 
1.4.2.2.Choix de la méthylprednisolone comme traitement de comparaison 
1.4.2.3. Mode d’administration et choix des doses 
1.4.3 Méthodes de mise en œuvre du double-insu 
1.4.3.1 Problème des anti-émétiques 
1.4.3.2 Perfusion de rinçage 
1.4.3.3 Suivi biologique 
1.4.4 Choix de la population étudiée

1.4.5 Choix du Critère de jugement principal 
1.4.6 Choix des critères secondaires 
2. OBJECTIFS
2.1 L’objectif principal
2.2 Les objectifs secondaires
3. METHODES GENERALES
3.1 Schéma d’étude 
3.2 Randomisation 
3.3 Sélection des centres investigateurs 
4. CRITERES D’ELIGIBILITE 
4.1 Critères d’inclusion
4.2 Critères de non inclusion
4.3 Critères d’exclusion 
5. TRAITEMENTS DE L’ESSAI 
5.1 Traitements 
5.1.1 Groupe Cyclophosphamide (CPM) 
5.1.2 Groupe Méthylprednisolone (MP)
5.2 Déroulement des traitements
6. TRAITEMENTS ASSOCIES 
6.1 Traitements antiémétiques

6.2 Traitements interdits 
6.3 Traitements déconseillés
6.4 Prise en charge des poussées 
7. CRITERES DE JUGEMENT 
7.1 Critère de jugement principal 
7.2 Critères de jugement secondaire 
7.3 Critères d’évaluation exploratoires 
8. DEROULEMENT DE L’ESSAI 
8.1 Investigateurs 
8.2 Calendrier de l’essai 
8.3 Consentement 
8.4 Visite de sélection (visite 1)
8.5 Période de sélection 
8.6 Visite d’inclusion  et d’initiation de traitement (visite 2)
8.7 Visites de suivi et hospitalisations pour traitement
8.8 Visites de fin de traitement et de fin d’étude
8.9 Survenue du critère principal de jugement

8.10 Levée d’insu
9. EVENEMENTS INDESIRABLES
9.1 Définition et déclaration d’un évènement indésirable grave
9.1.1 Définition
9.1.2 Déclaration et suivi des EIG
9.1.3 Conduite à tenir en cas d’évènement indésirable grave (EIG)
9.2 Conduite à tenir en cas de survenue de grossesse
10. MONITORAGE
10.1 Organisation générale
10.2 Cahiers d’observation

10.3 Audit
10.4 Archivage des documents à la fin de l’étude
11. SURVEILLANCE DE L’ESSAI 
11.1 Conseil scientifique
11.2 Comité de Méthodologie et de Gestion de l’essai
11.3 Comité de suivi et de validation des évènements
11.4 Comité indépendant de surveillance
12. ASPECTS STATISTIQUES
12.1 Nombre de sujets nécessaires
12.2 Méthodes statistiques prévues pour l’analyse
12.2.1 Généralités
12.2.2 Description de l’inclusion et du suivi
12.2.3 Caractéristiques des patients avant la mise sous traitement
12.2.4 Analyse du critère de jugement principal
12.2.5 Analyse des critères de jugement secondaires

12.2.6 Analyse des critères de jugement exploratoires
13 CONSIDERATIONS ETHIQUES ET REGLEMENTAIRES
13.1 Considérations éthiques générales et avis du CCPPRB
13.2 Amendements du protocole
13.3 Confidentialité des données
13.4 Assurance
14.DEVIATIONS DU PROTOCOLE
14.1 Arrêt de traitement, abandon de l’essai
14.2 Fermeture des centres, Violations de protocole

14.3 Modification du schéma thérapeutique
15 PUBLICATIONS DES RESULTATS
16 ASPECTS BUDGETAIRES, SURCOUTS

17. REFERENCES
18. ANNEXES

1. Calendrier de l’essai

2. Caractéristiques des traitements à l’essai (Zophren®, Cyclophosphamide CPM, méthyprednisolone MP).

3. Note d’information au patient

4. Formulaire de consentement

5. Coûts pharmacie

6. Attestation d’assurance responsabilité civile

7. Avis Favorable du CPP

8. Déclaration d’intention AFSSAPS

9. Déclaration d’Helsinki

**IV- RESUME DU PROTOCOLE :**

Justificatif : Le traitement actuel des formes secondairement progressives (SP) de sclérose en plaques (SEP) ne fait pas l’objet d’un consensus. Les médicaments utilisés actuellement peuvent présenter des indications restreintes ou des effets indésirables importants, ou leur efficacité n’a pas été démontrée de manière indiscutable. Le cyclophosphamide (CPM) a montré des résultats encourageants dans des études ouvertes mais il n’a pas encore été évalué de manière rigoureuse. Il s’agit d’un essai clinique randomisé multicentrique national de phase IIIb en double insu sur deux groupes parallèles.

L’objectif principal est de comparer l’efficacité du traitement par CPM par rapport à un traitement par méthylprednisolone (MP) administrés par voie IV pour empêcher l’aggravation confirmée sur 4 mois de 1 point d’EDSS (ou de 0,5 point si l’EDSS de départ est égale à 5, 5,5, 6 ou 6.5) à deux ans. L’étude concernera deux groupes randomisés parallèles. Le premier groupe recevra chaque mois pendant un an puis tous les deux mois pendant un an une dose de 750mg/m² de surface corporelle (SC) de CPM par voie intraveineuse (IV). Le second groupe recevra chaque mois pendant un an puis tous les deux mois pendant un an une dose de 1g de MP par voie IV.

Les objectifs secondaires sont de comparer dans les deux groupes de randomisation  la tolérance et la sécurité de ces traitements, l’effet des traitements sur des critères d’évaluation secondaire : score composite (MSFC) et les 3 z scores le composant, et le pourcentage de patients ayant progressé de 0,5 point ou 1 point de l’échelle EDSS (1 si l’EDSS initial est 4,0 ou 4,5 et de 0,5 point si l’EDSS initial est 5, 5,5, 6,0 ou 6.5) et nombre de poussées. La période d’inclusion est de 3 ans et demi et la durée de l’essai est de 2 ans par patient. (5 ans et demi).

Critères d’inclusion principaux : Homme ou Femme de 18 à 65 ans inclus présentant une forme secondairement progressive récente de SEP  avec une phase d’aggravation progressive du handicap d’au moins 6 mois et de moins de 4 ans avec une réduction du périmètre de marche et une aggravation du score EDSS d’au moins 0,5 point dans les 12 derniers mois non attribuable à des séquelles de poussées. Le score EDSS doit être situé entre 4,0 et 6,5 inclus avec un périmètre de marche d’au moins 50 mètres quelque soit l’aide (uni ou bilatérale).
Organisation :

Dans chaque centre, chaque patient sera suivi par deux médecins investigateurs, un neurologue traitant (NT) chargé de la prise en charge médicale et neurologique du patient et un neurologue évaluateur (NE) chargé de l’évaluation neurologique des patients. Le calcul de la taille de l’échantillon a été réalisé sur la base d’une proportion attendue de patients sans aggravation à 2 ans de 75% dans le groupe CPM et de 60% dans le groupe MP. Le calcul pour pouvoir comparer les délais d’aggravation observés dans chacun des groupes par un test du logrank de formulation bilatérale, avec un risque α = 5% et une puissance (1-β) = 80%, le nombre de sujets à inclure par groupe doit être au minimum de 155 patients. 180 patients par groupe seront inclus. L’analyse statistique sera réalisée en intention de traiter. Pour les tests statistiques, le risque de première espèce α est fixé à 5%. Le critère de jugement principal sera analysé dans un premier temps à l’aide de la méthode de Kaplan-Meier et les délais de survenue seront comparés entre groupes de traitement par un test du logrank. Dans un second temps, un modèle de Cox sera construit pour étudier l’effet des facteurs pronostiques.

**V- PROTOCOLE**

**1. JUSTIFICATION DE L’ETUDE :**

- 1. **Situation du problème**
     1. **Introduction**

La sclérose en plaques (SEP) est une affection de l'adulte jeune, invalidante et fréquente (prévalence en France estimée à 60 pour 100 000 habitants). Les plaques sont formées d'un infiltrat inflammatoire se développant autour de veinules et de zones de destruction de la myéline, avec une atteinte axonale associée (Brochet, 2001a). Sur le plan clinique, la maladie débute le plus souvent (85% des cas) par des poussées partiellement ou totalement régressives. Ces poussées correspondent à la survenue de nouvelles lésions inflammatoires du SNC. Dans la majorité des cas cette phase de poussées dite phase rémittente est suivie par une phase d’évolution progressive émaillée ou non de poussées surajoutées. La caractéristique majeure de cette phase secondairement progressive (SP) est l’existence d’une aggravation continue du handicap et, si il y a des poussées, cette aggravation doit persister en dehors des poussées (Lublin et Reingold, 1996). Le handicap présenté par les patients est vraisemblablement lié à des lésions myéliniques et axonales irréversibles. Ce handicap s'accumule avec le nombre de poussées et surtout du fait de cette progression secondaire et fait toute la sévérité de cette affection dans ses dimensions médicales, humaines, et socio-économiques. Compte tenu de la chronicité de la maladie et de l’âge moyen de début vers 30 ans et de la durée moyenne d’évolution (30 à 40 ans), l’impact de la maladie en terme socio-économique est considérable (Amato et al., 2002). La SEP génère des handicaps physiques et sociaux dont la lourdeur dépend de la forme clinique, du mode évolutif et du degré de sévérité de l’atteinte. Le handicap physique est générateur d’un handicap social et d’une incapacité de travail plus ou moins longue, source de pertes financières pour le malade et sa famille mais aussi pour la collectivité par le biais des différentes prestations, indemnités et allocations versées. Peu d’études ont été consacrées, à ce jour, au versant économique. Les études disponibles mettent tout d’abord l’accent sur le poids significatif des coûts indirects dans le coût total de la maladie (Amato et al., 2002) avec des répercussions sur le monde professionnel considérables. Touchant avec prédilection le sujet jeune, en pleine période d’activité professionnelle, la SEP est responsable d’incapacités et de mises en inactivité importantes (arrêts de travail, invalidités précoces). La SEP provoque également un retrait de la vie sociale et associative en raison du handicap qui confine progressivement les patients à leur domicile. L’incidence sur l’entourage n’en est que plus importante puisque l’état du patient peut conduire à la nécessité d’une aide pluriquotidienne dont l’ampleur s’accroît avec le handicap. Dans le même temps, les études montrent la part prépondérante des coûts hospitaliers dans les coûts directs, ce qui constitue un schéma commun à l’Allemagne et la France. Enfin pour les dépenses médicales comme pour les coûts indirects, deux variables cliniques apparaissent comme hautement prédictives du coût de cette maladie : la forme clinique et l’importance du handicap. Il est certain que toute prise en charge permettant de diminuer le handicap peut permettre de diminuer le poids de cette maladie sur la société. Il est donc essentiel de prendre le handicap comme critère d’efficacité des traitements.

- - 1. **Etat des connaissances sur les traitements actuels des formes secondairement progressives de Sclérose en Plaques.**
       1. **Traitements par interféron:**

Plusieurs traitements ont fait leur preuve dans le traitement de fond des formes rémittentes de SEP comme l’interféron bêta (Bétaféron ®, Avonex ® et Rebif ®) et l’acétate de glatiramère (copaxone ®) (Freedman et al., 2002, Noseworthy, 1999). Les résultats obtenus à la phase progressive pour ces produits sont moins évidents. Quatre études multicentriques ont été menées avec l’interféron bêta dans les formes SP. Deux études ont concerné le Bétaféron®. L'étude européenne (European study group, 1998) a étudié 718 patients avec un EDSS < 6,5 dont 358 patients ayant reçu du placebo et 362 de l’interféron. Le critère principal d'efficacité était le temps pour atteindre une progression confirmée du handicap c'est-à-dire d'un point de l’échelle Expanded Disability Status Scale (EDSS) dans la population en intention de traiter. Cette échelle ordinale dépend principalement de la marche à ce niveau de handicap. A 33 mois la différence entre les deux groupes était significative. A l’issue du suivi de 30 mois 49,8% des patients sous placebo avaient une aggravation d’un point d’EDSS contre 38,9% sous interféron. A noter que les différences ont été acquises rapidement : 13-15 mois après le début de l'étude 35% avaient progressé d'un point EDSS dans le groupe placebo contre 23% dans le groupe IFN. Après cette date les deux courbes sont très parallèles. Tout se passe comme si l'effet principal (action sur le processus inflammatoire?) survenait très tôt et qu'ensuite le traitement entretienne cet effet. Il faut en effet noter qu'une proportion élevée de patients avait eu des poussées dans les deux ans précédant l'inclusion (71,8 et 68,1% respectivement pour les groupes IFN et placebo). L'effet était cependant aussi important dans le groupe des patients n'ayant pas de poussées surajoutées que celui en ayant et quelque soit l'EDSS de départ. Les résultats en imagerie par résonance magnétique (IRM), positifs sur les lésions en T2 et T1 mais négatifs en ce qui concerne l'atrophie confirment que l'IFN agit surtout sur la constitution des lésions focales. Cet essai a conduit à l’obtention d’une AMM européenne pour le bétaféron® dans les formes SP. L'étude nord-américaine, non encore publiée (abstract Neurology, 2000 (54)p 2352), réalisée avec le même interféron (bétaféron ®) avec des critères d'inclusion et une méthodologie identique n'a pas confirmé ces résultats. Cette étude a inclus 939 patients ayant une SEP SP. Alors que les résultats IRM sur les lésions en T2 et T1 sont très positifs, il n'y a aucune différence en terme d'évolution d'EDSS entre les 2 groupes. Il a été proposé comme explication de la différence de résultats entre ces deux études l'existence de caractéristiques différentes entre les deux populations. Les patients de l'étude américaine avaient eu beaucoup moins de poussées dans la période précédant l'étude (56% sans poussée dans les 2 ans précédents) et avaient une phase progressive plus ancienne. Ce point est essentiel : la plupart des études thérapeutiques effectuées dans les formes SP montre une efficacité plus importante quand le début de la phase progressive est récent. L’étude IMPACT (Cohen et al., 2002) a étudié l’efficacité de l’interféron béta 1 a intramusculaire une fois par semaine à la dose de 60µg/semaine dans les formes de SEP secondairement progressive chez 436 sujets durant 2 ans versus placebo. Une efficacité significative a été retrouvée concernant l’évolutivité du handicap évalué par un score composite (Multiple Sclerosis Functional Composite, MSFC) qui était l’élément principal de jugement. Ce score qui combine les z scores d’un test de marche chronométrée de 25 pieds (TW25), d’un test chronométré d’habileté manuelle (nine hole peg test, 9HPT) et d’un test d’attention (PASAT version 3s) est plus sensible au changement que l’EDSS mais sa pertinence clinique est discutée (Cutter, 1999 ; Cutter et al., 1999). Une efficacité sur les poussées surajoutées a été retrouvée ainsi que sur l’activité en IRM. Il n’a pas été retrouvé par contre d’efficacité sur l’ambulation ou l’EDSS (critère secondaire). Les résultats de l'étude SPECTRIMS (SPECTRIMS study group, 2001), qui a étudié le Rebif ® 22µg et 44µg chacun 3 fois par semaines en sous-cutané contre placebo chez 618 patients ayant une SEP SP durant 3 ans vont dans le même sens. Dans cette étude les résultats IRM sont très significatifs, et meilleurs avec le 44 que le 22, mais les résultats cliniques concernant la progression du handicap sont négatifs. Une efficacité sur la fréquence des poussées surajoutées a été en revanche retrouvée. Le pourcentage de patients ayant eu des poussées dans les 2 ans précédents était de 47%, donc très proche de l'étude précédente. Une analyse post-hoc a montré que ces patients ayant eu des poussées dans les deux ans précédents avaient par contre une diminution significative de la progression du handicap. Une nouvelle indication d’AMM a donc été délivrée pour l’interféron béta 1 a sous cutané 3 fois par semaine (Rebif 44 ®), restreinte aux formes de SEP SP avec poussées surajoutées. Il semble donc que si l'interféron peut avoir un effet positif dans les formes SP, cet effet a beaucoup plus de chance d'être observé si on traite ces patients le plus précocement possible et s’il persiste des arguments en faveurs du caractère inflammatoire de la maladie (persistance de poussées, évolutivité inflammatoire en IRM). Ces essais ont cependant permis de montrer plusieurs points importants :

- Il existe une certaine sensibilité des formes SP aux traitements à visée immunologique (effet concordant en IRM dans les 4 essais) mais l’effet des immunomodulateurs reste modeste dans ces formes

- Les outils d’analyse utilisés (EDSS) sont peu sensibles au changement à ce stade de la maladie mais la signification clinique des autres critères (score composite) est incertaine. Ces essais ont concerné principalement des patients ayant une forme peu évolutive (patients s’étant aggravés d’un point EDSS sur 2 ans avant l’étude dans la majorité des cas) chez lesquels la mise en évidence d’un effet thérapeutique est plus difficile.

- L’effet est plus net si la forme SP est plus récente ou associée à des poussées.

- - - 1. **Mitoxantrone :**

D’autres traitements ont été essayés. La mitoxantrone, un immunosuppresseur puissant, a montré des résultats positifs au cours d’un essai randomisé contre placebo (Hartung et al., 2002) confirmant l’impact possible des traitements immunosuppresseurs dans ces formes mais ce traitement pose des problèmes de toxicité à long terme qui empêche son utilisation prolongée. L’AMM récente accordée à ce produit (ELSEP) concerne les formes SP mais à condition que le patient ait une forme très évolutive (deux points minimum d’aggravation d’EDSS dans l’année précédente et IRM active).

- - - 1. **Corticoïdes :**

Une étude a comparé un traitement par de fortes doses de méthylprednisolone (MP) IV tous les deux mois pendant 2 ans à de faibles doses de corticoïdes chez 109 patients ayant une SEP SP (Kinkel 1999). L'objectif mesuré était la proportion de sujets avec un échec thérapeutique (aggravation d'une des composantes d’un score composite pendant au moins 5 mois ou 3 poussées en 12 mois). Parmi les patients traités à forte dose, 38,9% ont atteint cet objectif contre 53,7% dans le groupe contrôle. Ce résultat n'était pas significatif mais l'analyse par courbe de survie a montré un effet modeste mais significatif du traitement.

- - - 1. **Traitement par cyclophosphamide :**

Le cyclophosphamide (CPM) est un agent alkylant qui est couramment utilisé dans le traitement de maladies auto-immunes comme le Lupus Erythémateux Disséminé (LED). Il a fait l’objet de différentes études dans la SEP sans qu’un consensus ne soit atteint quant à son efficacité.

- - - - 1. **Données d’efficacité :**

Les premières études concernaient un protocole d’induction à fortes doses sur quelques jours qui ne s’est pas révélé adapté à une maladie inflammatoire chronique nécessitant un traitement prolongé. C’est pourquoi les résultats initiaux encourageants (Hauser et al., 1983) n’ont pas été confirmés par deux essais contrôlés (Likovsky, 1991 ; Canadian Cooperative Multiple Sclerosis Study Group, 1991). L’essai principal réalisé au Canada (Canadian Cooperative Multiple Sclerosis Study Group, 1991) a été pénalisé par l’existence d’un groupe contrôle très stable puisque seuls 29% des patients sous placebo se sont aggravés en 30 mois de suivi moyen. Depuis ces essais, des études ouvertes ont concerné l’utilisation de bolus mensuels à plus faibles doses selon les protocoles utilisés dans le LED dans les formes progressives de SEP (750mg/m² de surface corporelle par mois) (Weiner et al., 1993 ; Hohol et al., 1999 ; Zephir et al., 2004). L’étude de Weiner et al.,(1993) a ouvert la voie en traitant par bolus mensuels des patients ayant reçu le traitement à forte dose. L’effet rapporté était plus important chez les sujets plus jeunes et ayant une forme progressive récente. L’étude de Hohol (1999) a confirmé ces résultats avec des résultats encourageants, en particulier chez les patient ayant une durée de progression antérieure courte : 2,1 ans en moyennes et inférieurs à 5 ans en général. L’étude rétrospective française (Zéphir et al., 2004) portant sur 490 patients a récemment montré que ce traitement permettait une stabilisation ou amélioration de 78% des patients ayant une sep SP et 73,5% des formes progressives primaires à un an (Zéphir et al., 2004). Les patients ayant une amélioration de l’EDSS avaient une ancienneté de la phase SP de 5,1 ans en moyenne. Plusieurs études pilotes de phase II, ont concerné des formes rémittentes comme celle de Killian et al. (1988) qui portait sur seulement 14 patients. Des études non encore publiées mais présentée à l’American Academy of Neurology en 2003, ont montré l’intérêt des bolus de CPM pendant 6 mois dans les formes rémittentes ou SP s’aggravant très rapidement (Smith et al., Patti et al. ; Gauthier et al.). Smith et al. ont étudié 58 patients en phase rémittente RRMS en échec d’interféron (soit 2 poussées en un an, soit deux cures IV de corticoïdes en 1 an soit aggravation de 1,5 EDSS en 1 an) traités par 3 jours de méthylprednisolone (MP) IV puis randomisés ensuite entre 6 cures mensuelles de cyclophosphamide (CY) (800 mg/m2) plus MP ou MP seule. Les patients ont poursuivi l’ IFN b-1a (30 mg IM par semaine) pendant la phase de traitement IV de 6 mois puis les 18 mois de suivi. Le critère primaire d’efficacité était le changement du nombre de lésions rehaussées par le gadolinium en IRM. Un mois après les 3 cures initiales de stéroïdes le nombre moyen de lésions rehaussées était de 0,87 et était équilibré entre les 2 groupes (p=0,71). A 3 mois une augmentation moyenne de + 0,57 lésions rehaussées a été mesurée sous stéroïdes contre une diminution de 0,7 dans le groupe CPM (p=0,01). A 6 mois l’augmentation était de + 0.19 en moyenne sous corticoïdes contre une diminution de 0.77 sous CPM (p=0,04). Pendant la phase IV seules 8 IRM sur 60 étaient « actives » sous CPM contre 21 sur 54 sous MP. Le nombre moyen de lésions rehaussées était 1,18 sous MP contre 0,2 sous CPM (p=0.001). 6 mois après l'arrêt du traitement IV une augmentation de 0,58 lésions Gd + était observée dans le bras MP contre une diminution de 0,53 sous CPM (p=0.02). Patti et al ont étudié cliniquement et en IRM pendant 36 mois après un traitement de 18 mois combinant l’interféron beta et le CPM dans un groupe de 10 patients ayant une SEP rémittente très active et en échec d’IFNB . Après les 18mois de combinaison ils ont poursuivis l’IFN. Le taux de poussées très abaissé s’est maintenu pendant le suivi (0.13) et l’EDSS est resté stable (2.35 en moyenne), de même que le nombre de lésions en T2 alors qu’aucune nouvelle lésion rehaussée par le gadolinium n’était détectée sur des IRM annuelles.

Gauthier ont analysé 47 patients ayant une SEP rémittente traités par CPM IV et MP en secours après échec des autres traitements (interféron ou acétate de glatiramère) à cause d’une progression clinique (EDSS) [n=21] ou de changements sur l’IRM [n=24] ou une combinaison entre les deux. Les patients avaient un EDSS de 3.2 et une durée de maladie de 6.53 ans en moyenne. 80 % avaient été traités avec l'interféron-beta et 20 % avec copaxone ®. Le traitement (mensuel / 6 mois) a interrompu la progression de la maladie dans 78 % des cas (EDSS) et a stabilisé l'activité IRM dans 75 %.

- - - - 1. **Données de tolérance :**

Ces différentes études ont montré une tolérance acceptable de ce traitement au cours de la SEP aux doses utilisées. Dans l’étude rétrospective française seuls 4,5% des patients ont présenté un événement indésirable sévère (nausées et vomissements, leucopénie, infections, alopécie réversible et hépatites). Seul un patient a du interrompre ce traitement du fait de ces événements indésirables (hépatite). Les doses utilisées (750 mg/m² de surface corporelle (SC) permettent d’éviter d’atteindre les doses cumulées à risque onco-hématologique.

Un risque de cancer de la vessie a été rapporté chez les patients ayant une SEP et ayant une sonde urinaire à demeure (ce qui constituera un critère de non inclusion) et ayant reçu des doses cumulées de plus de 50 grammes de cyclophosphamide ce qui est très supérieur aux doses proposées (De Ridder et al., 1998). Le mode d’action supposé du CPM utilisé à ces doses dans la SEP ne serait pas lié à une immunosuppression mais à un effet immunomodulateur : augmentation des lymphocytes de type Th2, sécréteurs d’interleukine 4 et porteurs du récepteur aux chémokines CCR4 (Comabella et al., 1998, Karni et al., 2004). Un essai contrôlé du CPM en bolus mensuels intraveineux (IV) est nécessaire pour établir son efficacité dans les formes SP récentes.

- - - - 1. **Retombées attendues de l’étude :**

Le traitement actuel des formes SP de la SEP ne fait pas l’objet d’un consensus. Les médicaments utilisés actuellement peuvent présenter des indications restreintes ou des effets indésirables importants, ou leur efficacité n’a pas été démontrée de manière indiscutable. Le cyclophosphamide semble donner des résultats encourageants dans le traitement des formes SP de SEP, mais il n’a pas encore été évalué de manière rigoureuse. L’essai clinique randomisé en double insu qui fait l’objet de ce protocole permettra d’évaluer l’efficacité et la tolérance du cyclophosphamide dans les formes SP de la SEP. Si les résultats en sont positifs, une nouvelle option thérapeutique sera disponible pour ralentir l’aggravation du handicap neurologique chez les patients concernés.

- 1. **Description des traitements à l’essai :**
     1. **Traitements à l’essai :**

L’étude concernera deux groupes randomisés parallèles. Le premier groupe recevra chaque mois pendant un an puis tous les deux mois pendant un an une dose de 750mg/m² de surface corporelle (SC) de cyclophosphalmide (endoxan ®) par voie intraveineuse (IV). Le second groupe recevra chaque mois pendant un an puis tous les deux mois pendant un an une dose de 1g de méthilprednisolone (solumédrol ®) par voie IV. (Caractéristiques de produits : annexe 2)

- - 1. **Intérêt par rapport aux traitements disponibles :**

Il n’existe pas de traitement de référence des formes SP de SEP. Comme nous l’avons vu trois médicaments disposent d’une AMM dans cette indication mais ne peuvent pas être considérés comme traitement de référence :

- - - 1. **L’interféron bêta :**

Le bétaféron ® (interféron bêta 1b) dispose d’une AMM pour les formes SP et le rebif 44 ® (interféron bêta 1a) dispose d’une AMM pour les formes SP avec poussées surajoutées. A l’heure actuelle la grande majorité des patients débutant une forme SP de SEP a été traitée par interféron bêta pour la phase rémittente. Le début de la phase SP signifie chez ces patients un échec du traitement de fond par interféron et ils ne relèvent donc pas, dans ce cas, de la poursuite d’un tel traitement. L’intérêt du traitement à l’essai est de stabiliser la maladie de ces patients ne répondant pas aux interférons. Les études pilotes décrites plus haut laissent augurer d’une efficacité chez les patients en échec d’interféron. Les patients débutant une phase SP, réunissant les critères d’inclusion du protocole et n’ayant pas eu d’interféron et ne présentant pas de contre-indications à ce traitement auront le choix de participer à l’étude ou de choisir de recourir au traitement par interféron. Les patients seront informés dans la notice d’information de la disponibilité du traitement par interféron et des résultats des études le concernant dans cette forme, en particulier l’absence d’effet sur le handicap mesuré par EDSS dans 3 des 4 études réalisées. S’ils font le choix de l’interféron et que ce traitement échoue ils pourront être inclus dans le protocole, s’ils le souhaitent, qu’ils réunissent toujours les critères d’inclusion et que la période d’inclusion ne soit pas terminée.

- - - 1. **Mitoxantrone :**

L’AMM concernant ce traitement ne concerne que les patients ayant eu une aggravation d’au moins deux points d’EDSS dans l’année précédente et la présence de lésions prenant le contraste sur l’IRM. Il s’agit donc de patients ayant une forme agressive de SEP qui ne relèvent pas de ce protocole.

- - - 1. **Corticoïdes :** Voir plus loin choix du comparateur.
  1. **Hypothèse testée :**

L’hypothèse testée dans l’étude est que le traitement par cyclophosphamide administré par voie IV en bolus mensuels pendant un an puis bimestriels pendant un an est supérieur à la méthylprednisolone (MP) administrée par voie IV au même rythme pour empêcher l’aggravation confirmée sur 4 mois de 1 point d’EDSS (ou de 0,5 point si l’EDSS de départ est égale à 5, 5,5, 6 ou 6.5) à deux ans. Il s’agit d’un essai de supériorité.

- 1. **Choix méthodologiques :**

L’objectif général de l’étude est d’évaluer l’efficacité (en termes de stabilisation) et la tolérance du cyclophosphamide (CPM) dans le traitement des formes progressives secondaires récentes de sclérose en plaques. Le schéma le plus adapté est celui d’un essai clinique randomisé en double insu.

- - 1. **Traitement évalué :**
       1. **Choix :**

Les études préliminaires, non comparatives, du CPM dans les formes progressives de SEP ont montré des résultats encourageants, notamment lors de l’administration du produit en bolus mensuels (Cf. Chapitre 1.1.2.3.). Cependant, seul un essai clinique randomisé permettra d’évaluer de manière valide son efficacité.

- - - 1. **Choix de la dose :**

Le mode d’administration (bolus par voie IV) et la dose (750mg/m² de surface corporelle par bolus) sont ceux qui ont fait l’objet des études ouvertes (Hohol et al., 1999; Zephir et al., 2004).

- - 1. **Choix du Traitement de comparaison :**
       1. **Existe-il un traitement de référence des formes SP :**

Bien que trois médicaments possèdent l’AMM en France dans cette indication (Interférons Bêta et mitoxantrone), il n’existe pas à l’heure actuelle de traitement de référence indiscutable pour les patients présentant des formes SP de SEP. En effet, le Betaferon® et le Rebif ® sont indiqués dans les formes rémittentes et la plupart des patients en bénéficient actuellement. Ainsi, le passage à une forme SP pour ces patients correspond à un échec thérapeutique qui ne justifie pas la poursuite de ce traitement. Le Rebif® possède l’AMM seulement pour les formes SP avec poussées surajoutées. Les résultats montrant une efficacité de ce médicament ont été obtenus, au cours d’un essai randomisé contre placebo, seulement dans le sous-groupe de patients ayant présenté des poussées et en regroupant les deux doses de Rebif étudiées, lors d’une analyse post hoc (SPECTRIMS study group, 2001). Les résultats étaient statistiquement significatifs sur le pourcentage de patients ayant une progression du handicap à 3 ans, mais seule une tendance était retrouvée pour le délai de progression du handicap. Ainsi, l’efficacité de ce produit n’est pas validée de manière indiscutable, même dans le sous-groupe de patients pour lesquels le traitement possède l’AMM. Enfin, l’Elsep® (mitoxantrone) possède une AMM seulement pour les formes SP très évolutives et présente une toxicité hématologique importante et une cardiotoxicité qui limitent son utilisation.

- - - 1. **Choix de la méthylprednisolone comme traitement de comparaison :**

L’absence de traitement de référence indiscutable des formes SP de SEP pourrait faire discuter la comparaison du traitement évalué à un placebo. Cependant, l’aggravation observée au cours de la phase SP de la SEP étant irréversible, il est difficilement envisageable de laisser des patients ayant une forme SP de SEP récente et évolutive sous placebo. Plusieurs études du CPM et de la mitoxantrone (Edan et al., 1997, Smith et al., 2003, AAN) ont utilisé la méthylprednisolone en bolus mensuels IV comme comparateur. L’étude décrite plus haut (Kinkel, 1999) n’a pas montré d’effet significatif par rapport au placebo mais l'analyse par courbe de survie a montré un effet modeste mais significatif du traitement. Le recours à ce comparateur peut risquer de diminuer la puissance de l’étude en diminuant la différence entre les groupes comparés mais ce risque apparaît modéré et il devrait faciliter l’acceptation du protocole et donc les inclusions

- - - 1. **Mode d’administration et choix des doses :**

La méthylprednisolone (Solumédrol) sera administrée à raison de 1 g par voie IV, une fois par mois pendant 1 an (selon le protocole de l’étude de Kinkel puis une fois tous les deux mois pendant un an pour suivre le même rythme que du cyclophosphamide (Endoxan®).

- - 1. **Méthodes de mise en œuvre du double-insu :**

Les essais thérapeutiques dans la SEP font systématiquement appel à deux investigateurs, l’un chargé de l’évaluation des critères de jugement (neurologue évaluateur NE) et l’un chargé de l’évaluation des événements indésirables et de la prise en charge du patient (neurologue traitant NT).

- - - 1. **Problème des anti-émétiques :**

Un problème spécifique posé par cette étude est la nécessité de prévenir les troubles digestifs (nausées, vomissements) dus au cyclophosphamide. Les protocoles habituels utilisent les médicaments anti-émétiques type anti-sérotonine (Zophren ®) (caractéristique du produit : annexe 2). Il est indispensable que les patients sous cyclophosphamide reçoivent un traitement de ce type pour limiter les événements indésirables, pour le confort des patients et pour maintenir au maximum le double-insu pour le neurologue traitant. En revanche il ne serait pas justifié d’administrer ce produit aux patients sous MP. De même à la fin du traitement intraveineux, du zophren® per os est habituellement prescrit pendant quelques jours. Pour régler ce problème la stratégie adoptée sera la fourniture par la pharmacie du zophren ® injectable ou de serum glucosé isotonique dans des seringues ne permettant pas d’identifier le produit par l’investigateur et les infirmières et la prescription d’une ordonnance pour tous les patients à la sortie de zophren ® per os « si besoin » qui y recourront en cas de nausées ou de vomissements.

- - - 1. - **Perfusion de rinçage :**

Tous les patients, quelque soit le groupe recevront la perfusion de rinçage de sérum glucosé dont le but est d’éviter les effets irritants vésicaux du cyclophosphamide. La durée de cette perfusion sera identique pour tous les patients, soit 8 heures afin de permettre la réalisation du traitement en hospitalisation pour les deux groupes.

- - - 1. **- Suivi biologique :**

Le CPM induit fréquemment une lymphopénie alors que le MP induit plus fréquemment une polynucléose neutrophile. Cependant cette dernière est très transitoire et ne devrait pas persister sur le bilan réalisé avant l’hospitalisation suivante. Pour éviter que ces constatations biologiques ne perturbent l’insu les bilans biologiques seront transmis uniquement au centre coordonnateur qui ne les transmettra au neurologue traitant qu’en cas de nécessité médicale (suspicion d’infection par exemple). C’est le centre coordonnateur qui transmettra aux pharmaciens les consignes pour adaptation de dose.

- - 1. **Choix de la population étudiée :**

La population étudiée est représentée par les patients atteints d’une forme SP de SEP pour lesquels il n’existe pas de traitement de référence efficace sur le handicap. Les données des études ouvertes du cyclophosphamide indiquant que ce traitement est principalement efficace si le début de la phase progressive est récent il a été choisi de sélectionner des patients dont la phase progressive a moins de 4 ans. Afin de mettre en évidence un effet clinique la population sélectionnée doit présenter des signes objectifs d’aggravation qui sont définis par la réduction du périmètre de marche critère majeur de mesure de la progression. Cette aggravation minimale est de 0,5 point d’EDSS sur les 12 derniers mois à cause d’une réduction du périmètre de marche. Afin de mesurer l’effet du traitement sur le périmètre de marche les patients doivent présenter une limitation de ce périmètre mais être capables de marcher, ce qui correspond aux bornes des critères d’éligibilité de 4 à 6.5 points d’EDSS avec un périmètre de marche d’au moins 50 mètres quelque soit l’aide (uni ou bilatérale).

- - 1. **Choix du Critère de jugement principal :**

Le handicap conditionne la qualité de vie des patients atteints de SEP, le pronostic de leur maladie et les coûts médico-économiques attribuables à cette maladie. La mesure la plus utilisée du handicap dans la SEP est l’échelle « expanded disability status scale » (EDSS) (Kurtzke et al., 1983). C’est pourquoi le délai d’aggravation du score EDSS a été choisi. Une des critiques concernant cette échelle concerne sa variabilité inter-observateur (Brochet, 2001b). La variabilité est moindre dans le segment 4 - 6.5 de l’échelle dont la cotation dépend essentiellement des mesures de marche. Elle peut à ce stade de la maladie être due à une évaluation du périmètre de marche ne reposant que sur l’interrogatoire et sur des divergences d’interprétation de l’échelle en fonction du périmètre de marche. Afin d’éviter ces problèmes il sera demandé une mesure objective du périmètre de marche et la transposition en cotation EDSS sera effectuée par les experts du centre coordonnateur à partir de ces données brutes de mesure du périmètre de marche.

- - 1. **Choix des critères secondaires :**

Le score composite de la sclérose en plaques (Multiple Sclerosis Functional Score, MSFC) a été développé récemment pour proposer un score plus sensible. Il représente la moyenne des z scores d’une mesure de marche chronométrée, d’un test d’habileté manuelle et d’un test d’attention auditive. Ce score a été utilisé dans les essais thérapeutiques récents sur la SEP et constitue le critère principal de l’étude IMPACT de l’avonex ® dans les formes SP de SEP. Afin de permettre la comparaison avec ces études ce score sera utilisé comme critère secondaire ainsi que les sous-scores le composant.

Le nombre de poussées sera pris en compte pour comparaison avec les essais existant. Il sera également fait appel à titre de critères exploratoires à des autoquestionnaires de qualité de vie et de handicap. L’échelle SEP-59 (Vernay et al., 2000) est une échelle de qualité de vie validée dans la SEP en français. L’échelle MSIS est une échelle brève de handicap récemment traduite (Roullet, communication personnelle).

1. **OBJECTIFS**
   1. **L’objectif principal** de cette étude est de comparer l’efficacité d’un traitement par cyclophosphamide (CPM) en bolus IV à 750 mg/m² SC répétés mensuellement pendant un an puis tous les deux mois pendant un an par rapport à la méthylprednisolone (MP) en bolus IV à la dose de1 g délivrés au même rythme, sur le délai d’aggravation du handicap neurologique mesuré par l’échelle EDSS (plus d’1 point si l’EDSS de base est 4 ou 4,5 et plus de 0,5 point si l’EDSS de base est 5, 5,5, 6 ou 6.5) à deux ans et confirmé après 4 mois (ou 2 mois si elle survient à la dernière administration de traitement).
   2. **Les objectifs secondaires** sont de comparer dans les deux groupes de randomisation :

- la proportion de patients ayant progressé de 0,5 point ou 1 point de l’échelle EDSS (1 si

l’EDSS initial est 4 ou 4,5 ou 0,5 point si l’EDSS initial est 5, 5,5, 6 ou 6.5) à 2 ans (visite de fin de la période programmée de traitement),

- l’effet des traitements sur des critères d’évaluation secondaire : score composite (MSFC) et

les 3 z scores le composant,

**-** le nombre de poussées,

- la tolérance aux traitements.

1. **METHODES GENERALES**
   1. **Schéma d’étude :**

  Il s’agit d’un essai clinique randomisé multicentrique national de phase IIIb en double insu sur deux groupes parallèles. La période d’inclusion est de 3 ans et demi et la durée de l’essai est de 2 ans par patient.

- 1. **Randomisation :**

La liste de randomisation est établie par le statisticien de l’Unité de soutien méthodologique du CHU de Bordeaux (USMR) avant le début de l’essai. La randomisation est stratifiée sur le centre. Il n’est pas prévu de stratification sur les facteurs pronostiques éventuels, comme l’existence de poussées dans l’année précédant la randomisation ou la présence de lésions prenant le gadolinium. Les effectifs des deux groupes de traitement sont équilibrés. Un document complet décrivant la procédure de randomisation est conservé de manière confidentielle à l’USMR.

La randomisation des patients est effectuée de façon centralisée par le médecin prescripteur du centre coordonnateur via le site Internet de l’USMR, http:/usmr.isped.u-bordeaux2.fr. Lorsqu’un neurologue traitant souhaite effectuer la randomisation d’un patient, il envoie par fax les pages du cahier d’observation nécessaires à l’inclusion du patient au médecin prescripteur du centre coordonnateur qui vérifie la conformité des données aux critères d’inclusion. Le médecin prescripteur du centre coordonnateur remplit l’écran « demande de randomisation » et valide le contenu de l’écran. Si les données se révèlent conformes aux critères d’éligibilité, la randomisation est effectuée. Le médecin prescripteur du centre coordonnateur envoie le fax d’accord d’inclusion au neurologue traitant et ce dernier prévient la pharmacie de l’inclusion d’un nouveau patient. Lors de cette visite de première administration du traitement et pour chacune des visite suivantes, le pharmacien sera informé par le médecin prescripteur du centre coordonnateur, via l’ordonnance de prescription du traitement alloué au patient et de l’adaptation de la posologie en fonction du bilan biologique. Le médecin prescripteur et le pharmacien conserve de manière confidentielle le groupe de traitement attribué à chaque patient. Le traitement débutera au mieux dans les 7 jours après la demande de randomisation et au maximum 15 jours après. Ce délai peut être prolongé en cas de survenue d’anomalie clinique ou biologique n’autorisant pas le début du traitement avec accord du centre de coordination.

- 1. **Sélection des centres investigateurs :**

Ce protocole d’essai clinique a été proposé et discuté aux centres spécialisés dans le prise en charge de la SEP qui participaient à la réunion annuelle du club francophone de la SEP (CFSEP). Un certain nombre de centres ont alors manifesté leur désir de participation. Lors de la rédaction du protocole un comité scientifique a été constitué comportant des représentants de la grande majorité des centres de référence dans la prise en charge de la SEP (et dépassant les centres précédents) et le protocole a été discuté par voie électronique entre ces experts. Les centres sélectionnés pour l’étude représentent d’une part un grand nombre de services de neurologie de CHU ayant développés une expertise dans le domaine de la SEP et ayant dans leur très grande majorité participé à des essais cliniques dans le domaine et pour quelques uns de service de neurologie d’hôpitaux généraux ayant développés une expertise dans le domaine de la SEP et participant au réseau de prise en charge de la SEP en Aquitaine (AQUISEP).

1. **CRITERES D’ELIGIBILITE :**
   1. **Critères d’inclusion**:

Tous les critères suivants doivent être remplis par les patients pour être éligibles pour participer à l’étude :

- Homme ou Femme de 18 à 65 ans inclus ;

- Sujet affilié ou bénéficiaire d’un régime de sécurité sociale ;
- Présentant une forme secondairement progressive récente de SEP :
 - SEP certaine selon les critères de Mc Donald et al. (2001) ;
 - Forme secondairement progressive de SEP selon la définition de Lublin et Reingold (1996).

- Les patients doivent avoir présenté une phase d’aggravation progressive du handicap d’au moins 6 mois et de moins de 4 ans;
- L’aggravation du handicap récente doit être responsable d’une aggravation du score EDSS d’au moins 0,5 point dans les 12 derniers mois non attribuable à des séquelles de poussées ;
- Les patients doivent avoir présenté une réduction du périmètre de marche dans l’année écoulée;
- EDSS entre 4,0 et 6,5 inclus ;
- Les patients doivent :

- avoir signé un consentement éclairé après information et avant toute procédure nécessitée par le protocole;
- accepter de recourir à une contraception efficace durant toute la durée de l’étude s’ils sont en âge de procréer (préservatif ou contraception efficace du patient ou du partenaire).

- 1. **Critères de non inclusion**

Les patients ne peuvent être recrutés pour l’étude s’ils remplissent l’un des critères suivants :

- Personne placée sous sauvegarde de justice (article L1122-2 du code de la santé Publique).

- Autres formes cliniques de SEP :
 - Phase SP ayant débuté depuis plus de 4 ans ;
 - Forme rémittente pure de SEP sans progression entre les poussées ;
 - Forme progressive primaire de SEP ;
- Pathologies associées :
 -Présence de toute autre maladie autre que la SEP pouvant contribuer aux

symptômes et signes neurologiques ou en affecter l’évaluation ;

-Maladie organique ou psychiatrique compromettant la capacité du patient à comprendre les informations données et à donner ou retirer son consentement en toute connaissance des informations nécessaires et sa capacité à suivre le protocole ;

-Abus d’alcool ou de stupéfiants actuel ou dans les 2 ans précédents;

- Contre-indication aux traitements à l’essai :
 - Cystite hémorragique préexistante.
 - Allergie connue au cyclophosphamide, aux corticoïdes et en particulier au

Solumédrol ®;
 - Maladie infectieuse en cours ;
 - Patients porteurs d’une cathétérisation permanente de la vessie (sondage

urinaire à demeure) ;
 - Grossesse et allaitement. ;
 - Insuffisance médullaire sévère (Hb <9g/dl  ou Leucocytes <3000/mm3  ou

Lymphocytes < 900 /mm3  ou Plaquettes < 125 000/mm3). Ce critère devra

être réévalué après le consentement, pendant la période de screening au vu du

bilan biologique;
 - Etat psychotique ;
 - Ulcère gastrique ou duodénal en évolution ;
 - Diverticulose colique ;
 - Antécédents de troubles du rythme cardiaque après injection de MP IV ;
 - Atteinte hépatique sévère (ASAT > 3X limite supérieure de la normale) ou

rénale sévère (Créatinine > 180 µmol/L) ;
 - Diabète ;
- Participation en cours ou dans les 3 mois précédents à un autre essai thérapeutique ;
- Traitement antérieur par CPM, irradiation totale lymphoïde, anticorps monoclonal anti-CD4 ou anti-CD52 ou anti-VLA 4, cladribine ou cyclosporine A.

-  Traitement antérieur par mitoxantrone sauf si celui-ci a été effectué durant la phase rémittente et plus de 3 ans avant le début de l’étude. Dans ce cas la, un bilan des Numérations Formule Sanguine et de l’échographie cardiaque transthoracique devra être fourni.

- En cas de traitement antérieur par interféron bêta, imurel ou méthotrexate un intervalle d’au moins un mois doit être respecté entre l’arrêt de ce traitement et le début du traitement à l’étude. Pour les statines voir chapitre 6.
- En cas de traitement antérieur par corticoïdes intraveineux mensuels, ce traitement doit avoir été arrêté depuis au moins 1 an.

- Arrêt du traitement aux corticoïdes de 3 à 5 jours inférieur à 2 mois.

- 1. **Critères d’exclusion :** Voir chapitre 14.

1. **TRAITEMENTS DE L’ESSAI**:
   1. **Traitements**:

Les solutés de perfusions des produits testés seront préparés à la pharmacie hospitalière. Le pharmacien sera informé par le médecin prescripteur du centre coordonnateur du groupe dans lequel le patient a été randomisé. Les traitements préparés par la pharmacie seront étiquetés conformément à la réglementation selon la procédure opératoire standard de l’USMR et de manière à maintenir l'insu.

- - 1. **Groupe Cyclophosphamide (CPM)**:

Ce groupe recevra du Cyclophosphamide (DCI) anhydre (flacon de 1g de poudre pour préparation injectable). La posologie sera adaptée par le médecin prescripteur du centre coordonnateur en fonction des résultats de la NFS. La posologie est de 750 mg/m² de surface corporelle si les lymphocytes sont > 1400 ; 500 mg/m² si les lymphocytes sont entre 1000 et 1400 et 400 mg/m² entre 900 et 1000.

En cas de report répété de traitement (au moins deux fois) pour raison biologique, le médecin prescripteur en réfèrera à l’Investigateur Coordonnateur afin de discuter d’une éventuelle adaptation de posologie de traitement.

Afin de préparer une solution isotonique de CPM prête à l'emploi, la poudre doit être dissoute dans une solution de chlorure de sodium 0,9 %. La solution reconstituée ne doit pas être conservée au-delà de 48 heures et ne doit pas dépasser une concentration de 2 %. La voie d'administration est la voie veineuse en perfusion courte (3 heures). Le médicament préalablement reconstitué dans une solution de sérum glucosé à 5 % est introduit dans le liquide de perfusion (soluté injectable isotonique de glucose) par le pharmacien. Une fois le traitement reçu de la pharmacie, l’infirmière doit mettre en place une voie veineuse (2,5 litres de G5 sur 8 heures), faire l’injection de traitement anti-émétique (voir plus loin) puis, après 30 minutes, mettre en Y la perfusion de traitement à l’étude pendant 3 heures . Le schéma thérapeutique est le suivant : administration de CPM toutes les 4 semaines pendant 48 semaines (12 cycles) puis toutes les 8 semaines pendant 48 semaines.

- - 1. **Groupe Méthylprednisolone (MP)**:

Les patients du groupe MP recevront  un lyophylisat de méthylprednisolone (DCI) hemisuccinate (flacon de 500mg). La dose de MP sera de 1g reconstitué dans de l’eau pour préparation injectable (eau PPI). Les solutés seront préparés à la pharmacie. Les procédures d’administration seront identiques à celles du groupe CPM.

En cas de report répété de traitement (au moins deux fois) pour raison biologique, le médecin prescripteur en réfèrera à l’Investigateur Coordonnateur afin de discuter d’une éventuelle adaptation de posologie de traitement.

Une fois le traitement reçu de la pharmacie, l’infirmière doit mettre en place une voie veineuse (2,5 l G5 sur 8 heures), faire l’injection de traitement anti-émétique (voir plus loin) puis mettre en Y la perfusion de traitement à l’étude pendant 3heures. Le schéma thérapeutique est le suivant : administration de MP toutes les 4 semaines pendant 48 semaines (12 cycles) puis toutes les 8 semaines pendant 48 semaines.

- 1. **Déroulement des traitements**:

Les administrations se dérouleront au cours d’hospitalisations. Trois jours ouvrables avant son admission le patient doit effectuer un bilan sanguin qui lui aura été prescrit par le neurologue traitant à la visite précédente  et comprenant une numération formule sanguine (NFS), plaquettes, vitesse de sédimentation, un ionogramme sanguin, le dosage de l’urée, de la créatinine et des transaminases.

Le laboratoire devra faxer le résultat au médecin prescripteur du centre coordonnateur. Si le taux d'Hb <9g/dl  ou le nombre de leucocytes est <3000/mm3  ou de lymphocytes < 900 /mm3  ou de plaquettes < 125 000/mm3 le médecin prescripteur du centre coordonnateur informe le neurologue traitant qu’il doit envoyer au patient une ordonnance pour une nouveau bilan (NFS) une semaine plus tard et reporter l’hospitalisation d’une semaine.

En cas de report répété de traitement (au moins deux fois) pour raison biologique, le médecin prescripteur en réfèrera à l’Investigateur Coordonnateur afin de discuter d’une éventuelle adaptation de posologie de traitement.

Si il existe une augmentation des polynucléaires neutrophiles (> 2 fois la limite supérieure de la normale) et/ou une VS élevée (> 2X normale à la première heure) le médecin prescripteur du centre coordonnateur communique les éléments pertinents du bilan au neurologue traitant qui doit contacter le patient afin de déterminer s’il présente des signes infectieux (fièvre, brûlures mictionnelles, douleurs lombaires ou autre). Une visite (consultation) non programmée peut être décidée et un ECBU réalisé afin de décider de reporter l’hospitalisation.

A l’arrivée, il faut peser le patient, prendre la TA et la température, réaliser un ECG, et faire un examen des bandelettes urinaires : Si les bandelettes sont positives  il faut prélever un ECBU.

En cas de bandelettes urinaires positives ou d’ECBU positif et en l’absence de signe clinique d’infection urinaire (fièvre, signes locaux) et biologiques d’infection le traitement pourra être effectué sous les conditions suivantes : débuter traitement par un antiseptique ou antibiotique, par exemple, noroxine ® (norfloxacine) 400 mg matin et soir pour 5 jours (en respectant contre-indications et règles d’associations médicamenteuses). La procédure pour le traitement anti-émétique est décrite au chapitre 6.

1. **TRAITEMENTS ASSOCIES**:
   1. **Traitements antiémétiques**:

Les patients du groupe CPM doivent recevoir un traitement antiémétique :

8mg de Zophren ® en IV lente dans la tubulure 30 minutes avant la mise en place du soluté de perfusion du traitement à l’étude suivi d’une administration de 8mg en IV lente dans la perfusion 4 heures et 8 heures après la première injection. Afin de préserver l’insu il sera organisé la fourniture par la pharmacie du zophren ® injectable ou de sérum glucosé isotonique dans des seringues ne permettant pas d’identifier le produit par le neurologue traitant et les infirmières et étiquetés avec le numéro d’identification du patient.

Les patients du groupe CPM devant également recevoir des antiémétiques per os pendant quelques jours après la perfusion une prescription sera remise à tous les patients de zophren ® per os « si besoin » afin que ceux-ci puissent y recourir en cas de nausées ou de vomissements à la dose de 8mg toutes les 12 heures jusqu’à cinq jours.

- 1. .**Traitements interdits**:

Sont contre-indiqués pendant toute la durée de l’essai le recours à une irradiation totale lymphoïde, aux anticorps monoclonaux anti-CD4, anti-CD52 ou anti-VLA 4, à la mitoxantrone, à la cladribine ou la cyclosporine A, et au vaccin contre la fièvre jaune, les autres vaccins vivants atténués (sauf fièvre jaune), et d’une façon générale tout agent immunosuppresseur ou immunomodulateur (dont azathioprine, methotrexate, interférons, acétate de glatiramère, pentoxyphylinne). Le recours à tout autre traitement en cours d’évaluation clinique est également interdit.

- 1. **Traitements déconseillés**:

Plusieurs études expérimentales et une étude clinique suggère un effet possible des statines dans la SEP. Les patients sous statine à dose fixe depuis au moins 12 mois et réunissant cependant les critères d’éligibilité dans l’étude pourront être inclus dans l’étude. Si au cours de l’étude l’état de santé d’un patient nécessite la mise sous statine le neurologue traitant devra préférer si possible la prescription d’une autre classe d’hypolipémiant. Si l’indication est formelle et que l’état morbide justifiant cette prescription ne constitue pas un critère d’exclusion de l’étude (par exemple accident vasculaire cérébral invalidant) le patient pourra être maintenu dans l’étude.

- 1. **Prise en charge des poussées**:

Les patients recevront des consignes afin de contacter le centre en cas de suspicion de poussée dès que possible. Le neurologue traitant devra alors demander au patient de venir au centre pour une visite non programmée comprenant une consultation avec lui et un examen neurologique avec le neurologue évaluateur au plus tard 9 jours après l’appel du patient.

La définition des poussées retenue dans l’étude est l’apparition de nouveaux signes neurologiques ou l’aggravation de signes neurologiques présents de façon aiguë pendant au moins 48 heures et responsable d’une augmentation de 0,5 point d’EDSS au moins OU d’un point d’un score fonctionnel (FSS) de Kurtzke. Afin de distinguer les poussées d’une aggravation progressive plus rapide il sera requis pour retenir une poussée que l’aggravation doit s’être constituée en moins d’un mois.

En cas de survenue d’une poussée le neurologue traitant peut décider la mise en route d’un traitement. Le protocole conseillé (Brochet, 2001c) est : Méthylprednisolone 1 gramme dans un soluté de glucosé à 5% sur 3 heures pendant 5 jours sans relais oral. Les hospitalisations pour poussée doivent être consignées comme événement indésirable mais ne constituent pas un événement indésirable grave.

1. **CRITERES DE JUGEMENT** :
   1. **Critère de jugement principal**:

Le critère de jugement principal est le délai d’aggravation du score EDSS (Kurtzke, 1983). Cette aggravation est définie par une augmentation de 0,5 point de l’échelle EDSS si l’EDSS initial est 5, 5,5, 6 ou 6,5 ou 1 point si l’EDSS initial est 4,0 ou 4,5 et à condition que cette aggravation soit confirmée lors des évaluations effectuées quatre mois après la première visite montrant cette aggravation. Si l’aggravation est constatée lors de la dernière visite de la période de traitement la confirmation à deux mois, lors de la visite de fin d’étude, sera suffisante. Le délai d’aggravation sera calculé par la différence entre la date de la première visite avec cette aggravation et la date d’initiation du traitement.

Afin de diminuer le risque de variations inter observateurs dans les cotations de l’EDSS il sera demandé aux neurologues évaluateurs de réaliser un examen neurologique type qui leur permettra de coter les échelles fonctionnelles FSS de Kurtzke qui servent à établir l’EDSS dans les premiers niveaux de l’échelle et de mesurer le périmètre de marche sur 500 mètres. Le score EDSS sera établi au niveau du centre coordonnateur à partir de ces données sources.

- 1. **Critères de jugement secondaire**:

- Proportion de patients ayant progressé de 0,5 point ou 1 point de l’échelle EDSS (1 si l’EDSS initial est 4 ou 4,5 ou 0,5 point si l’EDSS initial est 5, 5,5, 6 ou 6,5) à 2 ans (visite de fin de la période programmée de traitement). 
- Score composite de la SEP (Multiple Sclerosis composite score : MSFC) à deux ans (Cutter, 1999). Le MSFC comprend la combinaison de 3 mesures, une mesure chronométrée d’habileté motrice du membre supérieur, le nine hole peg test (9HPT), une mesure de marche : temps de marche (T.M.) chronométré sur 8 m, et un test cognitif mesurant l’attention, le PASAT (version 3 secondes). Le score MSFC est établi à partir des 3 z scores de ces 3 mesures selon la formule : (Z 9HPT – Z TM + Z PASAT)/3.
- Z scores du 9 HPT, z score du TM et z score du PASAT 3s à deux ans.
- Nombre de poussées pendant l’étude.
– Tolérance au traitement : proportion et délai de survenue des événements indésirables graves et non graves, évalués à la fin de l'étude.

- 1. **Critères d’évaluation exploratoires :**

Ces critères ne seront pas considérés comme des critères d’efficacité :

- Auto questionnaire de qualité de vie (échelle SEP 59, Vernay et al., 2000) à la 1^ère^ visite d’administration du traitement (semaine 0, visite 2), à un an (semaine 44, visite 13) et à la dernière visite administration du traitement (semaine 92 ,visite 19) permettant d’établir 15 scores correspondants aux axes de l’échelle.

- Auto questionnaire de handicap de l’échelles MSIS et auto questionnaire d’évaluation de la marche (MSWS12) à la 1^ère^ visite d’administration du traitement (semaine 0, Visite 2), à un an (semaine 44, visite 13) et à la dernière visite d’administration du traitement (semaine 92, visite 19).

- Tous les critères de jugement secondaires recueillis aux visites 13 (semaine 44) et 19 (semaine 92).

- Recueil de la numération des éosinophiles des bilans biologiques comme critère d’action du cyclophosphamide ( Endoxan®) (Smith DR et al, 2005).

1. **DEROULEMENT DE L’ESSAI :**
   1. **Investigateurs :**

Dans chaque centre, chaque patient sera suivi par deux neurologues :

- Le neurologue traitant (NT) sera chargé de la prise en charge médicale et neurologique du patient et en particulier de l’évaluation des événements indésirables cliniques et biologiques. Il sera en insu du groupe assigné. Il sera responsable de tous les aspects de la prise en charge neurologique (examen clinique, prise en charge des poussées).

-Le neurologue évaluateur (NE) sera chargé de l’évaluation neurologique des patients : examen neurologique, mesure du temps de marche et du périmètre de marche, autres échelles cliniques. Il pourra être aidé pour la mesure du MSFC et du périmètre de marche par un technicien de recherche clinique ou une infirmière. Il est en insu du groupe de traitement (ainsi que les personnels cités ci-dessus) et ne doit recevoir aucune information du patient ou d’autre membre de l’équipe sur les événements indésirables et les problèmes médicaux du patient autres que le recueil des symptômes neurologiques nécessaire à l’évaluation. Il n’a pas accès aux classeurs de recueil (CRF) du NT. Il transmet les feuilles d’évaluation au NT qui les classe.

- 1. **Calendrier de l’essai :** Tableau (annexe 1)
  2. **Consentement :**

Lors de la visite de sélection, avant toute autre procédure prévue dans l’essai, le neurologue traitant (NT) informera le patient de l’objectif, de la nature des contraintes et des risques prévisibles de l’essai. Il remettra au patient la lettre d’information détaillée et le formulaire de consentement (figurant en annexe 3, 4). Le patient pourra obtenir toutes les réponses à ses questions de la part du médecin avant toute procédure d’évaluation. Si le patient donne son accord de participation, il inscrira son nom ainsi que le neurologue traitant sur le formulaire de consentement puis ils dateront et signeront ce formulaire en 3 exemplaires. Un exemplaire du consentement sera conservé par l’investigateur en lieu sûr pour une durée de 30 ans après la fin de l’essai. Une autre copie sera remise au patient et le troisième exemplaire sera remis au promoteur à la fin de l’essai.

- 1. **Visite de sélection (visite 1) :**

Elle est assurée par le NT. Au cours de cette visite le NT :

- vérifie les critères d’éligibilité,

- Informe le patient des modalités, des contraintes et des risques prévisibles de l’essai et recueille le consentement,

- procède à un examen clinique général, à une mesure du périmètre de marche permettant de vérifier que le patient remplit les critères d’inclusion en terme d’EDSS (évalué par le NE),

- recueille les antécédents, les pathologies associées et les traitements en cours et passés,

- remet une prescription pour le bilan biologique (NFS, plaquettes, VS, ionogramme sanguin, glycémie, créatininémie, transaminases et pour les femmes en âge de procréer test de grossesse),

- une mesure du résidu post-mictionnel sera réalisée par bladder scan ou échographie,

- en absence de bilan radiologique (thorax, sinus, dents) datant de moins d’un mois, prescrit cet examen,

 - en absence de bilan radiologique (thorax, sinus, dents) datant de moins d’un mois, prescrit ces examens. Il est laissé à l’appréciation de l’investigateur de faire ou non une IDR à la tuberculine,

- donne les consignes au patient concernant les poussées.

- 1. **Période de sélection :**

Pendant la période de sélection, située entre la visite de sélection et la visite d’inclusion, qui ne doit pas excéder 5 semaines, est réalisé un examen par imagerie par résonance magnétique avec injection de gadolinium. Cet examen ne fait pas partie des évaluations du protocole puisqu’il est considéré comme rentrant dans le suivi habituel des patients et permettra d’analyser d’éventuels facteurs de prédiction de l’efficacité des traitements. Un protocole recommandé sera proposé aux centres.

Pendant cette période le NE (ou le technicien de recherche si le centre en dispose) doit faire passer à deux reprises le MSFC (temps de marche sur 10m, 9HPT, PASAT 3s).

Le NT communique les données nécessaires à l’inclusion du patient (critères d’inclusion et de non inclusion) au centre coordonnateur. Le médecin prescripteur du centre coordonnateur vérifie sur les résultats du bilan biologique (prescrit lors de la visite de sélection) que le patient ne présente pas de critères de non éligibilité. Le NT est informé par le centre coordonnateur de l’inclusion définitive du patient et qu’il peut convoquer le patient pour la visite de premier administration du traitement. Cette date de convocation sera communiquée par le NT au médecin prescripteur du centre coordonnateur afin d’envoyer la prescription du traitement à l’essai à la pharmacie afin que le traitement soit dispensé à cette date prévue.

- 1. **Visite d’inclusion  et d’initiation de traitement (visite 2):**

Cette visite a lieu en hospitalisation. Le NT vérifie que le patient remplit toujours les critères d’éligibilité. Il effectue un examen clinique général, note les traitements associés et les événements indésirables.

Le NE (avec l’aide éventuelle d’un technicien de recherche) procède à :

- un examen neurologique,

- une mesure du périmètre de marche sur 500 mètres chronométré avec l’aide minimum,

- une mesure du temps de marche sur 8 m,

- une mesure du NHPT, deux essais par main,

- un test de la PASAT 3s,

- Il doit remettre au patient les auto questionnaires que le patient doit remplir avant le début du traitement et récupérer les questionnaires remplis. Le NE remet au NT les résultats des évaluations et les questionnaires qui sont classés dans le CRF et dont une copie est transmise au centre coordonnateur.
- Avant le traitement il faut peser le patient, prendre la TA et la température, réaliser un ECG, et faire un examen des bandelettes urinaires : si les bandelettes sont positives  il faut prélever un ECBU. Les procédures du traitement sont décrites au chapitre 5.2. La procédure pour le traitement anti-émétique est décrite au chapitre 6.
- Après le traitement le NT recueille les événements indésirables éventuels et procède à un examen clinique.
- Les ordonnances (traitement anti-émétique éventuel et bilan biologique) sont remises au patient ainsi que son rendez-vous pour l’hospitalisation suivante.

Il est précisé que « Lors des visites de traitements, et dans le but de préserver l’insu, les patients des 2 bras recevront des repas non salés non sucrés.

- 1. **Visites de suivi et hospitalisations pour traitement :**

Les visites et traitements ont lieu toutes les 4 semaines durant 44 semaines puis toutes les 8 semaines, les 44 semaines suivantes en hospitalisation.

Si une des conditions indiquées au paragraphe 5.2 est présente, le centre coordonnateur en informe le NT qui propose au patient une visite non programmée et décale la visite et le traitement programmés. Si le centre coordonnateur a donné son feu vert pour le traitement le patient est hospitalisé.

Le NT effectue un examen clinique général, note les traitements associés et les évènements indésirables à l’occasion de chaque administration de traitement.

Les évaluations cliniques neurologiques (NE) ont lieu au mieux toutes les 4 semaines et au maximum toutes les 8 semaines durant 44 semaines, puis toutes les 8 semaines les 44 semaines suivantes en hospitalisation. Les visites n°2, 3 et 19 sont obligatoires (auto questionnaires et MSFC).

Le NE procède à :

- un examen neurologique,

- une mesure du périmètre de marche sur 500 mètres chronométré avec l’aide minimum,
 - une mesure du temps de marche sur 8 m (aux visites 13, 19, 20 et en cas de visite de fin de traitement prématuré),

- une mesure du NHPT, deux essais par main (aux visites 13, 19,20 et à la visite de fin d’étude et en cas de visite de fin de traitement prématuré),

- un test de la PASAT 3s (aux visites 13, 19, 20 et en cas de visite de fin de traitement prématuré).

Il doit remettre au patient les auto questionnaires que le patient doit remplir avant le début du traitement à la visite 2,13 et 19.

Le NE remet au NT les résultats des évaluations pour classement dans le CRF et une copie est transmise au centre coordonnateur.

Les procédures de traitement sont identiques à celle de la visite d’initiation de traitement.
- Après le traitement le NT recueille les événements indésirables éventuels et procède à un examen clinique.

- Les ordonnances (traitement anti-émétique éventuel et bilans biologiques) sont remises au patient ainsi que son rendez-vous pour l’hospitalisation suivante.

- 1. **Visites de fin de traitement et de fin d’étude :**

Huit semaines après la dernière administration programmée de traitement a lieu la visite de fin d’étude. Dans la période de 8 semaines qui précède cette visite une IRM encéphalique, rentrant dans le suivi normal de ces patients sera réalisée.

Au cours de cette visite : Le NT procède au recueil des événements indésirables, traitements associés et procède à un examen clinique.

Le NE procède à :

- un examen neurologique,

- une mesure du périmètre de marche sur 500 mètres chronométré avec l’aide minimum,
 - une mesure du temps de marche sur 8 m,

- une mesure du NHPT, deux essais par main,

- un test de la PASAT 3s,

Il doit remettre au patient les auto questionnaires que le patient doit remplir sur place.

Le NE remet au NT les résultats des évaluations pour classement dans le CRF et une copie est transmise au centre coordonnateur.

Un questionnaire d’évaluation de l’insu est rempli par le patient, le NE et le NT et le technicien de recherche éventuel.

En cas d’arrêt prématuré de traitement (Cf. paragraphe Déviations au protocole) une visite de fin de traitement est organisée huit semaines après la dernière administration au cours de laquelle le NT évalue les événements indésirables, les traitements associés et procède à un examen clinique. Si le patient ne souhaite pas poursuivre le suivi il est procédé à la visite de fin d’étude comme indiqué au début de ce paragraphe. Sinon les évaluations sont poursuivies comme prévues par le protocole jusqu’à la date programmée de visite de fin d’étude, date à laquelle a lieu la visite de fin d’étude. Les traitements reçus par le patient durant cette période seront recueillis.

A l’issu de l’étude, le neurologue traitant du centre entamera une discussion sur les options thérapeutiques pouvant être proposées au patient dans le cadre de la prise en charge classique de sa maladie. Le traitement par Endoxan ayant des aspects limitatifs quant à la dose cumulée pouvant être reçu par le patient, une communication du traitement à l’essai sera donc nécessaire. Les doses utilisées (750 mg/m² de surface corporelle (SC) permettent d’éviter d’atteindre les doses cumulées à risque onco-hématologique (Moore., 1991 ; Radis et al., 1995 et Talar-Williams et al., 1996) pendant l’essai. Afin d’éviter d’atteindre ces doses dans le cadre du traitement ultérieur de la maladie , une levée d’insu et une communication du traitement sera donc réalisé par le médecin prescripteur auprès du neurologue traitant. Le neurologue évaluateur restera en insu jusqu’à la fin de l’étude.

Le médecin prescripteur communiquera au neurologue traitant du centre, le traitement et les doses reçues par le patient.

- 1. **Survenue du critère principal de jugement :**

Le centre coordonnateur établit l’EDSS pour chaque patient dès réception des données de base des évaluations (périmètre de marche mesuré et examen neurologique). Dès qu’un patient présente le critère de jugement principal (augmentation de 1 point d’EDSS si l’EDSS initial est 4 ou 4,5 ou de 0,5 point si l’EDSS initial est 5, 5,5, 6 ou 6,5, confirmée à la visite réalisée quatre mois après) il en informe le NT. Lors de la visite suivante le NT doit en informer le patient et réévalue avec celui-ci les options thérapeutiques. Si le patient décide d’arrêter le traitement à l’essai et s’il est d’accord, il sera suivi jusqu’à la fin de l’essai selon le même calendrier que prévu initialement. Le maintien du patient dans l’essai permettra une meilleure analyse des critères de jugement secondaires.

En cas de nécessité pratique ce suivi peut être bimestriel. Si le patient refuse ce suivi, prévoir une visite de fin de traitement 4 mois après.

- 1. **Levée d’insu :**

En cas d’événement indésirable grave pour lequel une levée d’insu pourrait être nécessaire (légitime uniquement si la connaissance du traitement administré peut modifier la prise à charge à effectuer), le neurologue traitant contacte l’investigateur coordonnateur pour prendre cette décision après discussion et demande au médecin prescripteur de faire cette levée d’insu.

Pour chaque levée d’insu, le neurologue traitant doit noter précisément les raisons, la date et le nom de la personne qui a effectué la levée d’insu. Toute levée d’insu doit être notifiée au centre coordonnateur.

En cas d’urgence mettant en jeu la vie ou la santé du patient, le neurologue traitant pourra demander au pharmacien responsable du centre de lever l’insu s’il juge que cette information est indispensable à la prise en charge du patient sans attendre l’accord de l’investigateur coordonnateur.

1. **EVENEMENTS INDESIRABLES**
   1. **Définition et déclaration d’un évènement indésirable**
      1. **Définition:**

Un événement indésirable est une manifestation nocive et non recherchée, subie par une personne participant à une recherche biomédicale, quelle que soit la cause de cette manifestation, et qui n'est pas nécessairement liée au traitement évalué ou à la recherche.

Tous les événements indésirables survenus pendant l'étude seront recherchés et reportés dans le cahier d'observation.

Les événements suivants sont définis comme événements indésirables graves (EIG) :

- Décès ou événement menaçant le pronostic vital (en particulier événement indésirable de grade 4).

- Evénement entraînant une incapacité organique ou fonctionnelle ;

- Evénement nécessitant ou prolongeant une hospitalisation (à l’exception des hospitalisations prévues pour l’administration du traitement à l’essai et des hospitalisations pour poussée de SEP en l’absence de complications) ;

- Anomalie congénitale touchant la descendance d’un patient ayant reçu le traitement de l’essai ;

- **Evénements potentiellement graves**

Il s'agit d'événements indésirables ou de résultats anormaux d'analyse biologique, en tenant compte du contexte.

**- Evénements inattendus**

Evénements dont la nature, la gravité, l'évolution ou la fréquence ne concordent pas avec les informations relatives au produit (Brochure investigateur).

**- Evénements attendus**

Evénements dont la nature, la gravité, l'évolution ou la fréquence sont indiqués dans les informations relatives au produit (Cf. Annexe 2 : Caractéristiques des traitements à l’essai (Zophren®, Cyclophosphamide CPM, méthyprednisolone MP).

- - 1. **Déclaration et suivi des EIG :**

. **Déclaration**: Le promoteur/l’unité de vigilance déclare sans délai les EIG inattendus et les faits nouveaux survenus au cours de la recherche :

**-** à l’autorité compétente,

- au Comité de Protection des Personnes compétent. Le comité s’assure, si nécessaire, que les sujets participant à la recherche ont été informés des effets indésirables et qu’ils confirment leur consentement.

Pour les recherches portant sur un médicament, le promoteur/l’unité de vigilance enregistre dans la base de données EudraVigilance tous les EIG inattendus.

**Suivi de l'événement après sa notification** : Après sa notification initiale, l'EIG doit obligatoirement faire l'objet d'un suivi jusqu'à sa résolution. Le NT doit recueillir toutes informations complémentaires relatives à l'EIG sur le formulaire de déclaration complémentaire d'un événement indésirable grave et le faxer dès qu'il en a connaissance au CRPV de Bordeaux et au centre coordonnateur.

- - 1. **Conduite à tenir en cas d’évènement indésirable grave (EIG)**

La survenue d’un événement indésirable grave peut nécessiter un arrêt immédiat du traitement administré dans le cadre de l’essai, sur décision du NT. Le traitement d’urgence sera adapté à la situation rencontrée et, si nécessaire, au bras de randomisation du patient (l’investigateur s’adressera alors au pharmacien du site d’hospitalisation pour une levée d’insu). (Cf. Chapitre Levée d’insu).

- 1. **Conduite à tenir en cas de survenue de grossesse
     Notification d'une grossesse**

La survenue d'une grossesse dans la période ou au décours immédiat d'une étude chez une patiente participant à l'étude ou, chez la partenaire d'un patient participant à l'étude doit être déclare par l'investigateur selon les mêmes modalités qu'un EIG.

De même, toute interruption volontaire de grossesse (IVG), interruption thérapeutique de grossesse (ITG) ou fausse couche (FC) nécessitant une hospitalisation est un EIG à déclarer.

**Suivi de la grossesse après notification :** La grossesse fera l'objet d'un suivi particulier jusqu'à l'accouchement ou l'IVG

**Conséquences pour la prise en charge :** La survenue d’une grossesse chez une patiente participant à l'étude nécessite, après confirmation, une levée d’insu sur le bras de traitement ainsi qu’un arrêt immédiat de celui-ci. Cette décision devra également être confirmée par le Comité de gestion. Toutes les mesures devront être prises pour adapter le suivi de cette grossesse au traitement attribué à la patiente pendant l’essai.

1. **MONITORAGE**
   1. **Organisation générale.**

Afin d’assurer la mise en place, le suivi et le monitorage des centres un attaché de recherche clinique senior (chef de projet, CP) et deux attaché(e)s de recherche clinique (ARC) basé(e)s sur le centre coordonnateur seront recruté(e)s.

Le CP aidera l'investigateur coordonnateur (Pr Bruno Brochet) à élaborer les différents documents de l'étude en collaboration avec le centre coordonnateur.

Le CP et les ARC assureront la mise en place de l’étude dans les différents centres. Au niveau du centre coordonnateur  le CP, aidé des ARC, effectuera le suivi de l’essai, des inclusions, des EIG et des paramètres d’évaluation cliniques et biologiques. Il transmettra toutes les données nécessaires au suivi de l’essai et en particulier toutes les données biologiques à l’équipe médicale coordinatrice du centre coordonnateur  afin que celle-ci statue sur les adaptations de dose. Il transmettra aux pharmaciens les instructions relatives aux doses. Il transmettra les informations utiles également, au conseil scientifique et au comité de surveillance.

Visite de Monitorage : Les ARC assurent le monitorage de tous les centres par une visite sur site une fois tous les 4 mois environ à partir de la première inclusion, par des transmissions de courrier et de fax, et des appels téléphoniques réguliers.

La validation des données portera sur 100% des données clé (consentement éclairé, critères d'éligibilité, dates de suivi, événements indésirables graves, doses prescrites et reçues du traitement, évaluation neurologique).

Le CP et les ARC transmettent les données au centre de saisie selon un rythme et des modalités qui seront déterminées au début de l’essai.

- 1. **Cahiers d’observation :**

Dans chaque centre, les données seront recueillies sur des cahiers d’observation (CRF) imprimés en dupliqué qui seront fournis au début de l’étude. Ils sont gérés par le neurologue traitant (NT). Le NE n’a pas accès au CRF. Des formulaires d’évaluation seront remis au NE qui les transmettra au fur et à mesure au NT, avec les auto questionnaires remplis, qui les classera après transmission d’une copie au centre coordonnateur.

Les CRF seront conservés dans le centre pendant toute la durée de l’étude jusqu’à la fin de l’analyse afin de ne pas compromettre l’insu le NE disposera d’un dossier source séparé.

- 1. **Audit**

Le promoteur ou les autorités de santé peuvent pratiquer un audit des données pour vérifier le déroulement de l'étude et sa gestion selon les Bonnes Pratiques Cliniques. Il pourra également s'agir d'un audit indépendant mandaté par le promoteur.

- 1. **Archivage des documents à la fin de l'étude**

Les médecins investigateurs archiveront et conserveront

- pendant au moins 15 ans après la fin de l’étude les documents suivants relatifs aux études : version actualisée du protocole et des annexes et amendements éventuels ; cahiers d’observation; tous les autre documents et correspondance relatifs à l’essai.

- pendant une durée de 30 ans suivant la fin de l'étude : un exemplaire des consentements éclairés signés des participants stockés dans une enveloppe scellée.

Les dossiers sources des patients devront également rester disponibles pendant tout ce temps.
Le promoteur archivera

- pendant une durée de 15 ans : Le protocole et les amendements éventuels au protocole, les cahiers d'observation, tous les autres documents et courriers relatifs à l'étude

- pendant une durée de 30 ans suivant la fin de l'étude : un exemplaire des consentements éclairés signés des participants stockées dans une enveloppe scellée.

1. **SURVEILLANCE DE L’ESSAI :**
   1. **Conseil scientifique**

Le protocole a été élaboré après discussions au sein du conseil scientifique qui est composé des investigateurs principaux de chaque centre, des membres du centre coordonnateur et de l’équipe de l’Unité de Soutien Méthodologique à la Recherche Clinique et Epidémiologique du CHU de Bordeaux (USMR).

Pendant la durée de l’essai il se réunira au moins tous les 3 mois (réunion téléphonique ou physique). Il s’assure de la bonne marche de l’essai et du respect du protocole, et vérifie ses aspects éthiques. Il s’informe auprès du centre coordonnateur de l’état d’avancement de l’essai, des problèmes éventuels et des résultats. Il décide toute modification pertinente du protocole nécessaire à la poursuite de l’essai. En présence d’un rythme d’inclusion trop lent, d’un trop grand nombre de perdus de vue, de violations du protocole, ou pour des raisons médicales et/ou administratives, le Conseil Scientifique se réserve le droit de prendre la décision d’interrompre ou de poursuivre l’essai. Il précisera, avant la fin de l’essai, les modalités éventuelles du suivi prolongé des patients inclus dans l’essai.

- 1. **Comité de Méthodologie et de Gestion de l’essai**

1. Centre coordonnateur

Le centre investigateur coordonnateur est situé dans le département de neurologie du CHU de Bordeaux et travaille en collaboration avec le Centre de Méthodologie et de Gestion.

**Composition :**

L’équipe du centre investigateur coordonnateur est composée de :

- Pr Bruno Brochet,

- Mme Mathilde Deloire (Chef de Projet),

- Mme Madeleine Dumoulin (Médecin prescripteur),

- Mr Timothé Loock (ARC)

**Rôle :**

- Rédige le protocole
- Est chargé du monitorage de l’étude (Ouverture, suivi et clôture des centres investigateurs)
- S’occupe de l’organisation et logistique de l’étude
- Coordonne le fonctionnement des centres investigateurs, des pharmacies hospitalières.
- Informe le Conseil Scientifique du déroulement de l’étude,
- Prépare les réunions des différents comités de suivi de l’étude et des assemblées générales des investigateurs.
- Code les CRF
- Elabore les CRF ainsi que les différents documents de l’étude (Lettre d’information, consentement, classeurs investigateur, pharmacie et infirmier) en collaboration avec l’USMR
- Elabore et soumet les différents amendements au protocole
- Présente l’avancement de l’étude à l’USMR
- Le médecin prescripteur s’occupe de la gestion des bilans biologiques, des inclusions et des ordonnances de prescription.

2. Centre de méthodologie et de gestion

Le Centre de Méthodologie et de Gestion est situé à l’Unité de Soutien Méthodologique à la Recherche Clinique et Epidémiologique du CHU de Bordeaux (USMR) (coordonnateur méthodologiste : Paul Perez)

**Composition** :

L’équipe projet est composée de :

- Paul Perez, méthodologiste,

- Christine Germain et Nelly Fournet, statisticiennes,

- Monique Gachet, attachée de recherche clinique (ARC) référent,

- Guillaume Dupouy, analyste programmeur

Par ailleurs, deux opérateurs de saisie sont chargés de la double saisie des fiches du classeur d’observation.

**Rôle :**

- Supervise la conception méthodologique de l’étude.
- Collabore à la conception du protocole avec l’investigateur coordonnateur. Elle finalise la rédaction du protocole, des notes d’informations et formulaires de consentement et du cahier d’observation avant soumission au CPP et l’AFSSAPS.
- Soumet l’étude au CCTIRS et la déclare à la CNIL.
- Réalise le site Internet permettant l’accès sécurisé aux programmes de randomisation
- Réalise et gère la base de données informatique dédiée à l’étude.
- prépare en collaboration avec le chef de projet et les ARC du centre coordonnateur la mise en place et le suivi de l’essai
- Effectue l’analyse statistique des données.
- Participe aux publications et autres valorisations des résultats de l’étude.

Le Centre de Méthodologie et de Gestion, en collaboration avec l’ARC du centre coordonnateur participe à la préparation des dossiers facilitant la prise de décisions pour le Comité Indépendant de Surveillance, les résumés présentant l’état d’avancement de l’essai et les fichiers permettant l’analyse des données pour le Conseil Scientifique. »

- 1. **Comité de suivi et de validation des évènements**

Il est localisé à Bordeaux et est composé de membres de l’équipe médicale coordinatrice dirigée par le Pr B Brochet et est complété lors de réunions élargies d’experts extérieurs. Il se réunit chaque semaine tout au long de l’essai et organise des réunions élargies au moins une fois par trimestre. Le Comité de Validation des Evénements est chargé d’analyser et de classer les événements indésirables et leur imputabilité aux traitements de l’essai. Il est également chargé d’analyser et de valider les diagnostics de progression clinique selon le critère de jugement principal et d’en informer les investigateurs.

- 1. **Comité indépendant de surveillance**

Il est composé d’experts en neurologie, en pharmacovigilance et en méthodologie ne participant pas à l’essai. Le Comité Indépendant de Surveillance se réunit au moins une fois au début de l’essai, et une fois pour examiner les données de tolérance des 30 premiers sujets inclus puis tous les 3 mois (par conférence téléphonique). Il examine le rythme des inclusions afin de vérifier si l’essai permettra d’atteindre ses objectifs et il s’assure que l’essai se déroule conformément au protocole approuvé par le CCPPRB et conformément à la législation. Il revoit toutes les données disponibles sur les événements indésirables survenus au cours de l’essai.

1. **ASPECTS STATISTIQUES :**
   1. **Nombre de sujets nécessaires :**

Le critère de jugement principal est le délai d’aggravation du score EDSS (Cf. Chapitre 7).
L’aggravation est définie :

- par une augmentation du score de plus d’1 point (EDSS initial égal à 4 ou 4,5 points) ou de plus de 0,5 points (EDSS initial entre 5 et 6,5 points inclus),

- confirmée lors d’une visite de contrôle réalisée 4 mois après la 1^ère^ visite montrant cette aggravation ou 2 mois après si l’aggravation est constatée lors de la dernière visite de la période de traitement.

Le délai d’aggravation sera calculé par la différence entre la date de la 1^ère^ visite avec aggravation et la date d’initiation du traitement.

Le calcul de la taille de l’échantillon a été réalisé grâce au logiciel N-Query® (v 4.0). La proportion attendue de patients sans aggravation à 2 ans est de 75% dans le groupe CPM (76% à deux ans dans l’étude de Hohol et al, 1999) et de 60% dans le groupe MP ( Kinkel, 1999). Si l’on fait le calcul pour pouvoir comparer les courbes de survenue d’une aggravation dans chacun des groupes par un test du log-rank de formulation bilatérale, avec un risque α = 5% et une puissance (1-β) = 80%, le nombre de sujets à inclure par groupe doit être au minimum de 155 patients (2 groupes de même effectif). Pour une proportion attendue de perdus de vue égale à 5%, il est nécessaire d’inclure au moins 155 patients par groupe. Par précaution, 180 patients seront inclus dans chacun des 2 groupes.

- 1. **Méthodes statistiques prévues pour l’analyse:**
     1. **Généralités :**

L’analyse sera réalisée en intention de traiter, c’est à dire que tous les patients qui ont fait l’objet de la randomisation seront analysés dans leur groupe de randomisation, même s‘ils n’ont jamais pris le traitement, s’ils l’ont arrêté en cours d’étude, ou s’ils ont changé de traitement. Il n’est pas prévu d’analyse intermédiaire. Pour les tests statistiques, le risque de première espèce α est fixé à 5%. Les analyses statistiques seront réalisées avec le logiciel SAS.

- - 1. **Description de l’inclusion et du suivi :**

Le nombre de patients inclus, la courbe des inclusions (évolution du nombre de patients inclus entre la première et la dernière inclusion), le nombre de visites théoriques correspondant au nombre de patients inclus et le nombre de visites réellement effectuées seront présentés et comparés entre groupes.

- - 1. **Caractéristiques des patients avant la mise sous traitement :**

Les patients seront décrits selon le groupe de traitement et en fonction des violations au protocole :

- Patients exclus de l’analyse / Patients inclus dans l’analyse.

- Patients inclus dans l’analyse avec violations mineures du protocole / Patients inclus dans l’analyse sans violation mineure du protocole.

Les caractéristiques des patients avant la mise sous traitement seront décrites par groupes : fréquences pour les variables qualitatives ; moyennes, écarts-types, médianes, minimum et maximum pour les variables quantitatives.

- - 1. **Analyse du critère de jugement principal :**

Le critère de jugement principal est le délai d’aggravation du score EDSS (Cf. Chapitre 7), estimé 2 ans après le début du traitement. L’analyse sera réalisée lorsque tous les patients auront terminé l’ensemble du suivi prévu dans l’étude.

Dans un premier temps, les courbes de survenue d’une aggravation seront tracées à l’aide de la méthode de Kaplan-Meier et les délais de survenue seront comparés entre groupes de traitement par un test du log-rank.

Dans un second temps, un modèle de Cox sera construit pour étudier l’effet des facteurs pronostiques connus sur l’efficacité du traitement.

Les facteurs pronostiques analysés sont :

- l’existence de poussées dans les 12 derniers mois précédant le début du traitement,

- la présence de lésions rehaussées par le produit de contraste à l’IRM réalisée avant l’initiation du traitement,

- la durée de la maladie à la date d’initiation du traitement.

Le modèle de Cox prendra en compte chacun de ces 3 facteurs pronostiques ainsi que l’interaction entre le traitement et l’existence de poussée ou la présence de lésions rehaussées par le produit de contraste afin d’étudier une éventuelle différence d’effet des traitements en fonction de l’existence de ces facteurs pronostiques. En cas d’interaction statistiquement significative, les résultats des deux traitements seront décrits et comparés dans chaque sous-groupe de facteur pronostique.

- - 1. **Analyse des critères de jugement secondaires :**

- Proportion de patients aggravés à 2 ans : La proportion de patients aggravés à 2 ans sera décrite dans les 2 groupes de traitement (proportion et intervalle de confiance à 95 % de cette estimation). La comparaison des proportions de patients aggravés à 2 ans sera réalisée par un test du Chi² si les effectifs théoriques sont suffisants (n ≥ 5) ou par un test de Fisher sinon.

- Z-scores et MSFC : Le calcul des z scores utilisera comme population de référence les données de la population à l’inclusion selon les recommandations internationales (Cutter et al ., 1999). Les moyennes des z-scores et du MSFC seront comparées par un test de Student si les conditions d’application sont respectées (variances homogènes, distributions normales). Si les variances ne sont pas homogènes, on utilisera un test de Student pour variances inégales. Si la distribution n’est pas normale, on utilisera un test de Mann-Withney

- Nombre de poussées : Le nombre de poussées sera décrit dans les deux groupes de patients (moyenne et intervalle de confiance à 95 %, médiane, minimum-maximum). Les moyennes du nombre de poussées observées dans les deux groupes seront comparées par un test de Student si les conditions de validité du test sont respectées (distribution normale, variances homogènes). Si les variances sont inégales entre les deux groupes, on utilisera un test de Student pour variances inégales, si la distribution du nombre de jours n’a pas une distribution normale, on utilisera un test non paramétrique de Mann-Whitney

- Comparaison des groupes pour la tolérance : La proportion d’événements indésirables graves et non graves survenus au cours du suivi dans chaque groupe de traitement sera décrite, et les groupes seront comparés pour la fréquence de survenue de ces événements, leur gravité et leur imputabilité aux traitements de l’essai. Les comparaisons de proportions seront réalisées à l’aide du test du Chi-2 si les effectifs théoriques sont suffisants (n ≥ 5), ou par un test exact de Fisher sinon.

L’analyse du délai de survenue d’un événement sera effectuée en prenant en compte uniquement la première survenue de l’événement. Les courbes de survenue d’événement seront tracées à l’aide de la méthode de Kaplan-Meier. Les tests de comparaison du délai de survenue de l’événement entre groupes de traitement seront réalisés par un test du log-rank.

- - 1. **Analyse des critères de jugement exploratoires :**
- Auto questionnaire de qualité de vie, auto questionnaire de handicap de l’échelle MSIS et auto questionnaire de la marche à 1 an et à 2 ans
- Numération des éosinophiles

Pour décrire l’évolution de ces critères, ils seront comparés entre les deux groupes de traitement par une analyse de variance avec mesures répétées dans le temps.

- Pour chacun des critères secondaires (proportion de patients aggravés à 2 ans, Z-scores et MSFC, nombre de poussées), une analyse de variance avec mesures répétées dans le temps sera réalisée pour décrire l’évolution au cours de la période de suivi de chacun de ces critères.

Afin de mieux analyser l'effet des traitements comparés sur les différentes composantes motrices et cognitives de la maladie, il sera également analysé des critères exploratoires permettant de comparer l'effet des traitements sur la marche, la cognition (test PASAT) et sur le handicap global (EDSS).

Les critères exploratoires additionnels sont les suivants :

 - du nombre de réponses correctes par ligne du test PASAT

 -  du nombre de réponses correctes consécutives maximum par test PASAT.

- des aires sous la courbe des courbes des médianes de l'EDSS

- du score de périmètre de marche entre la ligne de base et la dernière évaluation entre les deux groupes.

| **Score** | **Périmètre de Marche** |
| --- | --- |
| **1** | Aucune limitation ou ≥ 700 m |
| **2** | ≥ 500 m à < 700m |
| **3** | ≥ 300 m à < 500m |
| **4** | ≥ 200 m à < 300m |
| **5** | ≥ 100 m à < 200m |
| **6** | > 100 m quelque soit l'aide |
| **7** | ≥ 25 m à = 100m quelque soit l'aide |
| **8** | ≥ 5 m à < 25m quelque soit l'aide |
| **9** | < 5m quelque soit l'aide |

1. **CONSIDERATIONS ETHIQUES ET REGLEMENTAIRES**
   1. **Considérations éthiques générales et avis du CPP**

Le promoteur et les investigateurs s'engagent à ce que cette étude soit réalisée conformément :

- au texte de la déclaration d’Helsinki adopté par l’Assemblée Mondiale en juin 1964, modifié à Tokyo (Octobre 75), à Venise (Octobre 83), et à Hongkong (Septembre 89), Washington 2002, Tokyo 2004. (Annexe 9)

- aux recommandations des Bonnes Pratiques Cliniques (ICH 4 du 1^er^ Mai 1996)

- au texte de la loi française n°88-11.38 sur la protection des personnes se prêtant à des recherches biomédicales (loi Huriet du 20/12/88, modifiée le 23/01/90)

- L'étude est conduite conformément au protocole, hormis dans les situations d'urgence nécessitant la mise en place d'actes thérapeutiques précis. Les investigateurs s'engagent à respecter le protocole en tous points en particulier en ce qui concerne le recueil du consentement, la notification et le suivi des événements indésirables graves.

Le protocole de cette étude, la note d'information aux patients et le formulaire de consentement (cf Annexe 3, 4) sera soumis au CPP Sud-ouest et Outre Mer III de Bordeaux et ne pourra débuter qu’après avoir reçu un avis favorable. La version définitive sera approuvée par le promoteur et tous les investigateurs participant à l’essai.

Le promoteur adresse avant le début du protocole une lettre d'intention à l'Agence Française de Sécurité des Produits de Santé (AFSSAPS) et informe les directeurs de hôpitaux concernés (annexe 8).

Les données enregistrées à l'occasion de cette étude feront l'objet d'un traitement informatisée à l'USMR du CHU de Bordeaux dans le respect de la loi "Informatique et Libertés" du 6 août 2004 complétée par la loi du 1^er^ juillet 1994 et son décret d'application du 9 mai 1995 et par la loi du 4 mars 2002.

La soumission au CCTIRS et la déclaration à la Commission Nationale de l'Informatique et des Libertés (CNIL) de ce traitement informatisé des données conformément à l'article 40 de la loi "Informatique et Libertés" seront effectuées par l'USMR.

- 1. **Amendements du protocole :**

Après l’approbation du protocole par le CPP, toute modification substantielle fera l'objet d'un amendement écrit qui sera soumis au Conseil Scientifique, au promoteur de l’essai, au CPP et signé par tous les médecins investigateurs. Les amendements mineurs, ne modifiant pas le sens du protocole, sont communiqués au CPP à titre d'information.

- 1. **Confidentialité des données :**

Chaque patient est identifié par un numéro d’identification, composé d’un numéro de centre investigateur sur 2 chiffres (01 à 99), choisi par le centre coordonnateur lors de l’accord de participation du centre, de la première initiale de son nom et de son prénom (code lettre), et d’un numéro de patient dans le centre, sur 2 chiffres (01 à 99), établi de façon séquentielle dans l’ordre d’inclusion des patients. Chaque centre investigateur tiendra à jour une liste de correspondance entre les nom et prénom de chaque patient inclus et son numéro d’identification dans l’essai. Cette liste sera conservée dans un lieu sûr fermant à clé.

Les données concernant un patient inclus et nécessaires à l’essai sont reportées dans le cahier d’observation du patient par le neurologue traitant, après chaque visite prévue dans le protocole. Il y insérera les fiches d’évaluation que le neurologue évaluateur lui transmet. Seuls sont notés sur le cahier d’observation le numéro d’identification du patient et le code lettre. Les cahiers d’observation sont conservés à l’écart du dossier clinique du patient, dans un lieu sûr. Seuls les doubles des fiches du cahier d’observation sont adressés au centre coordonnateur. Les doubles des fiches sont saisis en double saisie, puis classés dans un lieu sûr fermant à clé. Les données sont régulièrement chargées dans la base de données, gérées à travers un système de gestion de base de données et une application développée spécifiquement pour l’essai. Dans la base de données, chaque patient n’est identifié que par son numéro d’identification. L’accès à cette base de données est protégé par un mot de passe. Les actions possibles sur les données sont fonction des autorisations attribuées à chaque utilisateur de la base. Les patients seront informés que toutes les données de l’essai seront informatisées et conservées de façon confidentielle.

- 1. **Assurance**

Le CHU de Bordeaux, promoteur de cet étude a souscrit une assurance responsabilité civile auprès de la société Gerling France conformément aux dispositions de l’article L209.7 du code de santé publique du 20/12/1988 et art. 5 du 25/07/1991. Une copie de l'attestation d'assurance est jointe en Annexe 6.

1. **DEVIATIONS AU PROTOCOLE :**
   1. **Arrêt de traitement, abandon de l’essai :**

Le traitement peut être arrêté :

- en cas de retrait de son consentement par le patient. Tout patient peut décider de se retirer de l’étude à n’importe quel moment sans en fournir de raisons. Cependant l’investigateur peut demander au patient quelle est cette raison en lui précisant qu’il peut ne pas répondre à cette question.

- survenue d’une grossesse ou d’une autre condition de non inclusion,

- survenue d’un EIG nécessitant selon l’avis du NT un arrêt du traitement ou survenue d’un nouvel état morbide justifiant, selon le NT l’arrêt du traitement.

En cas d’arrêt prématuré du traitement tout effort doit être fait pour poursuivre le suivi jusqu’à la fin de l’essai selon le même calendrier que prévu initialement. Le suivi peut être bimestriel ou, si le patient refuse ce suivi, prévoir une visite de fin de traitement 4 mois après.

Quand le neurologue traitant envisage un arrêt prématuré de traitement, le centre coordonnateur doit être avisé immédiatement (dans un délai de 3 jours ouvrables) par télécopie. Une visite de fin de traitement et éventuellement de fin d’étude doit être organisée huit semaines après la dernière administration comme indiqué dans le paragraphe 8.8.

Lorsqu’un patient souhaite abandonner l’essai, comme il a le droit de le faire à tout moment, le NT proposera une visite de fin d’étude comme indiquée paragraphe 8.8 et devra en aviser le centre coordonnateur par fax et courrier.

Après l’abandon de l’essai par le patient celui-ci bénéficiera d’une prise en charge de son état de santé la meilleure possible compte tenu des connaissances du moment. Les patients ayant quitté l’étude ne seront pas remplacés.

- 1. **Fermeture des centres, Violations de protocole :**

En cas d’absence d’inclusion après 6 mois le centre de gestion se réserve le droit de fermer un centre et de le remplacer.

En cas de violation majeure au protocole manifeste (absence de consentement, erreur d’attribution de traitement) le centre coordonnateur se réserve le droit de retirer un patient du protocole et éventuellement de fermer un centre si les mesures prises pour corriger ces violations ne sont pas suivies d’effet. Les patients retirés du protocole ne seront pas remplacés.

Les violations mineures au protocole seront notées afin d’être prise en compte dans l’analyse.

En cas de perdus de vus les investigateurs devront tenter d’obtenir du patient des informations sur son devenir, et en particulier sur la survenue éventuelle du critère du jugement principal ou d’EIG.

- 1. **Modifications du schéma thérapeutique**

Les modifications éventuelles de dose ou de rythme d’administration du produit évalué (report d’une administration du traitement en cas de signes de toxicité) devront être notées dans le cahier d’observation.

1. **PUBLICATIONS DES RESULTATS :**

A l’issue de cette étude un comité de rédaction rédigera les publications scientifiques exposant les résultats et les soumettra aux journaux scientifiques internationaux les plus appropriés selon un choix pris par ce conseil scientifique à la majorité.

Sur ces publications il sera mentionné que le CHU de Bordeaux est promoteur de cet essai. Il sera fait également mention des sources de financement.

La liste des auteurs sera proposée par le centre coordonnateur au comité scientifique en fonction de la participation à l’élaboration du protocole, à son déroulement, à l’analyse des données et à la rédaction du manuscrit. Les autres personnes ayant contribué à l’étude seront réunies sous l’appellation «groupe d’étude français du cyclophosphamide dans la sclérose en plaques ».Les noms de tous les investigateurs de tous les centres seront cités et remerciés en fin d’article.

1. **ASPECTS BUDGETAIRES, SURCOUTS :**

Afin d’assurer la réalisation de l’étude un financement a été obtenu auprès du programme hospitalier de recherche clinique national 2004 Ce budget doit couvrir les frais médicamenteux inhérents à l’essai (cyclophosphamide), les surcoûts générés au niveau des pharmacies hospitalières par le travail de préparation et d’étiquetage des traitements à l’essai, le coût d’analyse statistique, les fournitures (CRF) et le recrutement d’un chef de projet et des ARC.

Les traitements préventifs des effets secondaires émétisants seront considérés comme des surcoûts et leur étiquetage par la pharmacie sera pris en compte.

1. **REFERENCES**

- Amato MP, Battaglia MA, Caputo D, Fattore G, Gerzeli S, Pitaro M, Reggio A, Trojano M; Mu. S. I. C. Study Group. The costs of multiple sclerosis: a cross-sectional, multicenter cost-of-illness study in Italy. J Neurol. 2002 Feb; 249 (2):152-63.

- Brochet B. Aspects physiopathologiques, cliniques, thérapeutiques de la sclérose en plaques. Encyclop.Méd.Chir. (Paris, France), Neurologie, 17-074-B-10, 2001a, 26p.

- Brochet B. Principales échelles neurologiques utilisées en pratique courante: sclérose en plaques. . Encyclop.Méd.Chir. (Paris, France), Neurologie, 17-035-A-81-2001b, 9p.

- Brochet B. Indications thérapeutiques lors des poussées de sclérose en plaques. Rev Neurol (Paris) 2001c, 157: 988-995.

- Canadian Cooperative Multiple Sclerosis Study Group. The Canadian cooperative trial od cyclophosphamide and plasma exchange in progressive multiple sclerosis. Lancet, 1991, 337: 442-446.

- Cohen JA, Cutter GR, Fischer JS et al., Benefit of interferon beta 1 a on MSFC progression in secondary progressive MS. Neurology, 2002, 59: 679-687.

- Comabella M, Balashov K, Issazadeh S et al. Elevated interleukin 12 in progressive multiple sclerosis correlates with disease activity and is normalized by pulse cyclophosphamide therapy. J Clin Invest, 1998, 102: 671-678.

- Cutter GR. Measures of impairment and disability. In : Rudick RA, Goodkin DE eds. Multiple sclerosis therapeutics. London : Martin Dunitz Ltd, 1999 : 19-30.

- Cutter GR, Baier ML, Rudick RA, Cookfair DL, Fisher JS, Petkau J et al. Development of a multiple sclerosis functional composite as a clinical trial outcome measure. Brain 1999 ; 122 : 871-882.

- de Ridder D, van Poppel H, Demonty L, DHooghe B, Gonsette R, Carton H et al. Bladder cancer in patients with multiple sclerosis treated with cyclophosphamide. J Urol 1998 ; 159 : 1881-1884.

- Edan G, Miller D, Clanet M, Confavreux C, Lyon-Caen O, Lubetzki C et al. Therapeutic effect of mitoxantrone combined with methylprednisolone in multiple sclerosis: a randomised multicentre study of active disease using MRI and clinical criteria. J Neurol Neurosurg Psychiatry 1997; 62 : 112-118.

- European Study Group on Interferon β−1b in secondary progressive MS. Placebo-Controlled multicentre randomised trial of interferon β−1b in treatment of secondary progressive multiple sclerosis. Lancet, 1998,352:1491-97.

- Freedman MS, Blumhardt LD, Brochet B, Comi G, Noseworthy JH, Sandberg-Wollheim M, Soelberg-Sørensen and the Paris Workshop group. International consensus on the use of disease-modifying agents in multiple sclerosis. Multiple sclerosis, 2002, 8: 19-23.

- Gauthier SA, Bharanidharan P et al., Treatment of Relapsing Remitting Interferon / Glatiramer Acetate Unresponsive Patients with Pulse Cyclophosphamide. AAN, 2003 (abstract P02-130).

- Gayou A, Brochet B, Dousset V. Transitional progressive multiple sclerosis: a clinical and imaging study. J Neurol Neurosurg Psychiatry 1997 ; 63 : 396-398.

- Hauser SL, Dawson DL, Lehrich JR et al. Intensive immunosuppression in progressive multiple sclerosis. A randomized three arm study of high dose intravenous cyclophosphamide, plasma exchange and ACTH. NEJM, 1983, 308: 173-180.

- Hartung HP, Gonsette R, Konig N, Kwiecinski H, Guseo A, Morrissey SP, Krapf H, Zwingers T; Mitoxantrone in Multiple Sclerosis Study Group (MIMS). Mitoxantrone in progressive multiple sclerosis: a placebo-controlled, double-blind, randomised, multicentre trial. Lancet. 2002 Dec 21-28;360(9350):2018-25.

- Hohol MJ, Olek MJ, Orav EJ, Stazzone L, Hafler DA, Khoury SJ et al. Treatment of progressive multiple sclerosis with pulse cyclophosphamide /methylprednisolone: response to therapy is linked to the duration of progressive disease. Mult Scler 1999 ; 5 : 403-409.

- Karni A, Balashov K, Hancock WW et al. Cyclophosphamide modulates CD4+ T cells into a T helper type 2 phenotype and reverses increased interferon gamma production of CD8+ T cells in secondary progressive multiple multiple sclerosis. J Neuroimmunolgy, 2004, 146: 189-198.

- Killian J.M., Bressler R.B., Armstrong R.M., Huston D.P. (1988). Controlled pilot trial of monthly intravenous cyclophosphamide in multiple sclerosis. Arch Neurol, 1988, 145 : 27-30.

- Kinkel RP. Methylprednisolone. In : Rudick RA, Goodkin DE eds. Multiple sclerosis therapeutics. London : Martin Dunitz Ltd, 1999 : 349-370.

- Kurtzke JF. Rating neurological impairment in multiple sclerosis : an expanded disability status scale (EDSS). Neurology 1983; 33 : 1444-1452.

- Likosky W.H., Fireman B., Elmore R.et al.. Intense immunosuppression in chronic progressive multiple sclerosis: the Kaiser study. J Neurol Neurosurg Psychiatry, 1991, 54: 1055-1060.

- Lublin FD, Reingold SC. Defining the clinical course of multiple sclerosis: results of an international survey. National Multiple Sclerosis Society (USA) Advisory Committee on Clinical Trials of New Agents in Multiple Sclerosis. Neurology 1996 ; 46 : 907-911.

- McDonald WI, Compston A, Edan G, Goodkin D, Hartung HP, Lublin FD, McFarland HF,Paty DW, Polman CH, Reingold SC, Sandberg-Wollheim M, Sibley W, Thompson A, van den Noort S, Weinshenker BY, Wolinsky JS. Recommended diagnostic criteria for multiple sclerosis: guidelines from the International Panel on the diagnosis of multiple sclerosis. Ann Neurol. 2001 Jul;50(1):121-7.

- Moore MJ. Clinical pharmacokinetics of Cyclophosphamide. Clin Pharmacokinet 1991 ; 20 : 194-208.

- Noseworthy JH, Gold R, Hartung HP. Treatment of multiple sclerosis: recent trials and future perspectives. Curr Opin Neurol 1999 ; 12 : 279-293.

- Patti F, Reggio E et al., Rapidly Transitional Multiple Sclerosis Patients Treated with Combination of Cyclophosphamide and Interferon Beta: Follow-Up 36 Months after Discontinuation of Therapy. AAN, 2003 (abstract P02-132).

- Radis CD, Kahl LE, Baker GL, Wasko MC, Cash JM, Gallatin A, Stolzer BL, Agarwal AK, Medsger TA Jr, Kwoh CK. Effects of cyclophosphamide on the development of malignancy and on long-term survival of patients with rheumatoid arthritis. A 20-year followup study. Arthritis Rheum 1995 ; 38 :1120-1127.

- Smith DR; Weinstock-Guttman, B, Cohen JA et al. Blinded, Randomized Trial of Pulse Cyclophosphamide in IFN B Resistant Active MS, AAN, 2003 (abstract S11-005).

- Smith DR; Weinstock-Guttman, B, Cohen JA et al. A randomized blinded trial of combination therapy with cyclophosphamide in patients-with active multiple sclerosis on interferon beta.

Mult Scler. 2005 ; 11(5):573-82.

- SPECTRIMS study group. Randomized controlled-trial of interferon beta 1a in secondary progressive MS. Neurology, 2001, 56: 1496-504..

- Talar-Williams C, Hijazi YM, Walther MM, Linehan WM, Hallahan CW, Lubensky I, Kerr GS, Hoffman GS, Fauci AS, Sneller MC. Cyclophosphamide-induced cystitis and bladder cancer in patients with Wegener granulomatosis. Ann Intern Med. 1996 ; 124 :477-484.

- Vernay D; Gerbaud L; Biolay S, Coste J, Debourse J, Aufauvre D et al. Qualité de vie et sclérose en plaques: validation de la version française d'un auto questionnaire (SEP-59). Rev Neurol 2000; 156 : 247-63.

- Weiner HL, Mackin GA, Orav EJ, Intermittent cyclophosphamide pulse therapy in progressive multiple sclerosis. Neurology, 1993, 43: 910-918.

-Zephir H, De Sèze J, Duhamel A et al., Treatment of progressive forms of multiple sclerosis with cyclophosphamide: a cohort study of 490 patients. J of Neurol Sci, 2004, 218: 73-77.

**18. ANNEXES :**

**ANNEXE 1 : Calendrier de l’essai**

Les mesures en italiques sont à effectuer par le NE.

**ANNEXE 2 : Caractéristiques des traitements à l’essai**

**1-ENDOXAN® injectable 500 mg** : **cyclophosphamide : BAXTER - Division Oncology**.

**FORMES ET PRESENTATIONS**

*Poudre pour solution injectable à 500 mg :*
Flacon + ampoule de solvant de 25 ml, boîte unitaire.
Modèle hospitalier : Flacon sans solvant, boîte unitaire.

**COMPOSITION**

|  | *p flacon* |
| --- | --- |
| Cyclophosphamide (DCI) anhydre | 500 mg |
| (soit en cyclophosphamide monohydraté : 534,5 mg/fl) | |

| *Solvant :* | *p ampoule* |
| --- | --- |
| Eau pour préparations injectables | 25 ml |

##### DC/INDICATIONS

- Traitement adjuvant et en situation métastatique des adénocarcinomes mammaires.
- Traitement des cancers ovariens, des cancers bronchiques notamment à petites cellules, des séminomes et carcinomes embryonnaires testiculaires, des cancers de la vessie, des sarcomes, des neuroblastomes, des lymphomes malins hodgkiniens et non hodgkiniens, des myélomes multiples, des leucémies aiguës, notamment lymphoïdes.
- A forte dose, conditionnement des allo et autogreffes médullaires.

A dose plus faible, traitement des polyarthrites rhumatoïdes, de certaines formes sévères de lupus érythémateux aigus disséminés, de néphropathies auto-immunes corticorésistantes.

**DC/POSOLOGIE ET MODE D’ADMINISTRATION**

**Posologie :** La posologie du cyclophosphamide est fonction de l'indication thérapeutique (traitement antitumoral ou immunodépresseur, type et localisation de la tumeur, traitement initial ou d'entretien) et de la place du médicament dans le traitement entrepris (utilisé seul ou en association avec d'autres médicaments cytostatiques). Elle est individuelle et doit tenir compte de l'état clinique et hématologique du patient (cf Mises en garde/Précautions d'emploi).

Le cyclophosphamide injectable est habituellement utilisé à des doses moyennes de 150 à 1200 mg/m ^2^ chez l'enfant et de 500 à 4000 mg/m ^2^ chez l'adulte, toutes les 3 à 4 semaines, administrées :

- sur 1 à 3 jours à chaque cycle,
- en deux injections à 7 jours d'intervalle.

**Mode d'administration :** Afin de préparer une solution isotonique prête à l'emploi, la poudre doit être dissoute dans une solution de chlorure de sodium à 0,9 % à raison de 50 ml pour 1 g. En cas de besoin, elle peut également être dissoute dans une solution de Ringer ou du sérum glucosé. La solution reconstituée ne doit pas être conservée au-delà de 48 heures et ne doit pas dépasser une concentration de 2 %.
La voie d'administration habituelle est la voie veineuse en perfusion courte (30 minutes à 2 heures). La perfusion sur 24 heures est également possible. Le médicament préalablement reconstitué dans une solution de chlorure de sodium à 0,9 % est introduit dans le liquide de perfusion (soluté injectable isotonique de glucose ou de chlorure de sodium).
Il est recommandé d'associer l'administration d'Uromitexan à partir de 600 mg/m 2/j et/ou d'assurer une hydratation suffisante.
Dans certains cas (capital veineux altéré), la voie IM peut être utilisée sans dépasser la dose de 500 mg par injection pour des raisons de volume. La dissolution dans du chlorure de sodium à 0,9 % permet d'obtenir une solution isotonique.
D'autres voies peuvent être utilisées comme la voie intra-artérielle.

**DC/CONTRE-INDICATIONS**

**Absolues :**

- Insuffisance médullaire sévère.
- Infection urinaire aiguë, cystite hémorragique préexistante.
- Allergie connue au cyclophosphamide.
- Grossesse et allaitement.
- Vaccin contre la fièvre jaune, phénytoïne à visée prophylactique (cf Interactions).

**Relatives :**

- Vaccins vivants atténués (cf Interactions).

**DC/MISE EN GARDE ET PRECAUTIONS D’EMPLOI**

**Mises en garde :**

- Les patients des deux sexes en période d'activité génitale doivent suivre une contraception efficace.
- La prudence est recommandée en cas d'insuffisance hépatique ou rénale préexistante qui devra, si besoin, être corrigée avant le début du traitement, ou pourra nécessiter une réduction de dose.
- Avant de débuter le traitement, il est nécessaire de contrôler les infections éventuelles et de corriger les troubles électrolytiques importants.
- Pendant le transport et le stockage d'Endoxan, il peut éventuellement se produire une liquéfaction du produit en raison d'une température trop élevée.
  Il est facile de distinguer visuellement les flacons qui ont subi une telle altération : le cyclophosphamide liquéfié apparaît comme un liquide visqueux, incolore ou jaunâtre (habituellement sous forme de gouttelettes ou d'une phase continue). Ne pas utiliser de flacons présentant une telle altération.

**Précautions d'emploi :**

- Une surveillance régulière de l'hémogramme est nécessaire pendant toute la durée du traitement (avant chaque cycle).
- L'utilisation du cyclophosphamide peut nécessiter une adaptation de la posologie ou une variation de l'espacement des cycles chez les patients présentant un diabète insipide, une leucopénie, une thrombopénie ou une infiltration cellulaire tumorale de la moelle osseuse.
- Lors de l'utilisation prolongée ou de l'utilisation de fortes doses du médicament ou chez les patients à risque (radiothérapie antérieure du petit bassin, toxicité thérapeutique vésicale antérieure...), il est recommandé d'assurer une hydratation abondante et d'associer la prise d'Uromitexan pour prévenir les risques de cystite hémorragique (cf Posologie/Mode d'administration). Il convient également de s'assurer que la diurèse du patient est bonne et de pratiquer si nécessaire des recherches d'hématuries microscopiques.
- L'alopécie peut parfois être prévenue par la mise en place d'un garrot pneumatique à la racine des cheveux lors du traitement ou par celle d'un casque réfrigérant.

**DC/INTERACTIONS**

**Interactions médicamenteuses :** En raison de l'augmentation du risque thrombotique lors des affections tumorales, le recours à un traitement anticoagulant est fréquent. La grande variabilité intra-individuelle de la coagulabilité au cours de ces affections, à laquelle s'ajoute l'éventualité d'une interaction entre les anticoagulants oraux et la chimiothérapie anticancéreuse, imposent, s'il est décidé de traiter le patient par anticoagulants oraux, d'augmenter la fréquence des contrôles de l'INR.

*Associations contre-indiquées :*

- Phénytoïne (introduite en prophylaxie de l'effet convulsivant de certains anticancéreux) ; décrit pour busulfan, ifosfamide, étoposide, téniposide : risque de majoration de la neurotoxicité (busulfan, ifosfamide) ou de perte d'efficacité du cytotoxique (étoposide, téniposide) par augmentation du métabolisme hépatique du cytotoxique par la phénytoïne.
- Vaccin contre la fièvre jaune : risque de maladie vaccinale généralisée mortelle.

*Associations déconseillées :*

- Vaccins vivants atténués (sauf fièvre jaune) : risque de maladie vaccinale généralisée éventuellement mortelle. Ce risque est majoré chez les sujets déjà immunodéprimés par la maladie sous-jacente.
  Utiliser un vaccin inactivé lorsqu'il existe (poliomyélite).

*Associations nécessitant des précautions d'emploi :*

- Phénytoïne (en cas de traitement antérieur à la chimiothérapie) ; décrit pour busulfan, ifosfamide, étoposide, téniposide : risque de majoration de la neurotoxicité (busulfan, ifosfamide) ou de perte d'efficacité du cytotoxique (étoposide, téniposide) par augmentation du métabolisme hépatique du cytotoxique par la phénytoïne. Surveillance clinique et adaptation de la posologie de l'anticancéreux.

*Associations à prendre en compte :*

- Ciclosporine (décrit pour doxorubicine, étoposide) : immunodépression excessive avec risque de lymphoprolifération.
- Tacrolimus (par extrapolation à partir de la ciclosporine) : immunodépression excessive avec risque de lymphoprolifération.

**DC/EFFETS INDESIRABLES**

La tolérance générale et locale du cyclophosphamide est bonne.
Une neutropénie et rarement une thrombopénie modérée peuvent être observées : elles sont toujours spontanément réversibles après diminution de la posologie ou à l'arrêt du traitement.
Certains patients peuvent présenter des nausées associées ou non à des vomissements qui sont facilement prévenus ou supprimés par les antiémétiques.
A forte dose, on peut également observer une toxicité gastro-intestinale à type de mucites et/ou de diarrhées.
L'alopécie est inconstante, transitoire et réversible.
Lors de l'emploi de doses élevées ou lors de traitements prolongés, il existe un risque de cystite hémorragique (cf Mises en garde/Précautions d'emploi) ainsi qu'une possibilité d'altération rénale, particulièrement en cas de lésions préexistantes.
Dans de rares cas, une hépatotoxicité avec modifications du bilan biologique hépatique a été observée.
Une aménorrhée ou une azoospermie sont possibles, voire définitives.
A très fortes doses, il existe un risque de cardiotoxicité (cardiomyopathie aiguë), potentialisé par irradiation antérieure de l'aire cardiaque ou l'utilisation d'anthracyclines et/ou de pentostatine.
En cas de leucopénies sévères, les mesures suivantes sont préconisées : administration d'antibiotiques et/ou d'antifongiques. Elles peuvent être prévenues par l'utilisation des facteurs de croissance granulocytaire.
Comme pour tout traitement cytostatique à doses cumulées élevées, le traitement par le cyclophosphamide peut être responsable de tumeurs secondaires. Le risque de développer une tumeur du tractus urinaire, comme un syndrome myélodysplasique pouvant évoluer en leucémie aiguë, est augmenté.

Autres effets indésirables :

- des cas de pneumopathies interstitielles, voire de fibroses pulmonaires, de pseudosécrétions inappropriées d'hormone antidiurétique (SIADH) ont pu être observées avec de fortes doses ;
- des réactions d'hypersensibilité au cyclophosphamide sont possibles, pouvant évoluer dans des cas isolés en état de choc ;
- des sensations vertigineuses associées à des troubles de la vision transitoires ont été rapportées.

**DC/GROSSESSE ET ALLAITEMENT** Contre-indiqué.

**DC/SURDOSAGE**

Il n'existe pas d'antidote spécifique du cyclophosphamide.
En cas de surdosage, il sera nécessaire d'adapter les soins en fonction de la toxicité constatée.
Au niveau rénal, l'uromitexan bloque le pouvoir irritant de l'acroléine, métabolite toxique pour la muqueuse vésicale formé au cours de la biotransformation du cyclophosphamide.
Le cyclophosphamide est dialysable.

**PP/PHARMACODYNAMIQUE**

Agent alkylant, moutarde à l'azote (L : antinéoplasique et immunomodulateur).
Agent alkylant bifonctionnel de type oxazaphosphorine appartenant à la famille des moutardes azotées agissant après transformation dans l'organisme.
Le cyclophosphamide agit par interaction directe sur l'ADN en formant des liaisons covalentes avec les substrats nucléophiles par l'intermédiaire de ses radicaux alcoyles. Ceci entraîne des modifications profondes chimiques ou enzymatiques de l'ADN ainsi que la formation de ponts alcoyles intrabrins ou interbrins, avec pour conséquence une inhibition de la transcription et de la réplication de l'ADN aboutissant à la destruction cellulaire. Cette action est cycle dépendant, elle respecte les cellules en Go.
Immunodépresseur.

**PP/PHARMACOCINETIQUE**

La molécule initiale est une prodrogue inactive. Elle est hydroxylée dans le foie par les microsomes hépatiques aboutissant essentiellement à la formation du 4-hydroxycyclophosphamide et à son tautomère l'aldo-cyclophosphamide puis à la moutarde phosphoramide (métabolite actif) et à l'acroléine (métabolite urotoxique).
Une voie métabolique accessoire conduit à une quantité négligeable des métabolites déchloréthylés.
Le temps moyen de demi-vie plasmatique du cyclophosphamide varie de 4 à 7 heures : il est plus court chez l'enfant (4 heures) que chez l'adulte (7 heures en moyenne).
Sous forme inchangée, il n'est pas lié de façon significative aux protéines plasmatiques (12 à 14 %) alors que ses métabolites le sont davantage (52 à 60 %).
La barrière hématoencéphalique est facilement traversée par le cyclophosphamide et un peu moins par ses métabolites (20 %) ce qui explique son intérêt dans le traitement de certaines tumeurs cérébrales. Son élimination à l'état inchangé ainsi que celle de ses métabolites est essentiellement urinaire.

**DP/CONDITIONS PARTICULIERES DE CONSERVATION**

A conserver à une température inférieure à 25 °C.
**- Après reconstitution :** 24 heures à une température comprise entre 2 °C et 8 °C et à l'abri de la lumière.
**- Après dilution dans le milieu de perfusion :** à utiliser immédiatement.

**DP/MODALITES DE MANIPULATION**

Utiliser de préférence en perfusion intraveineuse après dilution extemporanée dans 25 ml d'une solution de chlorure de sodium à 0,9 %.

**LISTE 1**

| AMM | 315 820.4 (1972/97 rév 13.06.2003) 1 fl 500 mg + solv. |
| --- | --- |
|  | 321 195.0 (1977/97 rév 13.06.2003) 1 fl 500 mg. |

| **PRIX :** | 5,12 € (1 fl 500 mg + solv). |
| --- | --- |

Remb Séc soc à 100 %. Collect.

Modèle hospitalier : Collect.

2- SOLU-MÉDROL® 20 mg, 40 mg, 120 mg, méthylprednisolone,  **PHARMACIA SAS**

## **FORMES et PRÉSENTATIONS**

*Poudre et solvant pour solution injectable à 20 mg, 40 mg et 120 mg :*
Flacon de lyophilisat + ampoule autocassable de solvant (2 ml), boîte unitaire.
Modèle hospitalier : Boîtes de 10 flacons.

COMPOSITION

| Lyophilisat : | p flacon |
| --- | --- |
| Méthylprednisolone (DCI) hémisuccinate exprimé en méthylprednisolone | 20 mg |
| ou | 40 mg |
| ou | 120 mg |

| Solvant : | p ampoule |
| --- | --- |
| Eau pour préparations injectables | 2 ml |

*Excipients :* Solu-Médrol 20 mg et 40 mg : phosphate monosodique anhydre, phosphate disodique anhydre, lactose. Solu-Médrol 120 mg : phosphate monosodique anhydre, phosphate disodique anhydre, solution d'hydroxyde de sodium à 10 % qsp pH 7,5 à 7,7.

**DC/INDICATIONS**

Celles de la corticothérapie générale per os, lorsque la voie parentérale est nécessaire en cas d'impossibilité de la voie orale (vomissements, aspiration gastrique, troubles de la conscience).

Les affections nécessitant un effet thérapeutique rapide :

- Allergiques : œdème de Quincke sévère en complément des antihistaminiques ; choc anaphylactique en complément de l'adrénaline.
- Infectieuses : fièvre thyphoïde sévère, en particulier avec confusion mentale, choc, coma ; laryngite striduleuse (laryngite sous-glottique) chez l'enfant.
- Neurologiques : œdème cérébral des tumeurs, de l'hématome sous-dural et œdème cérébral lié à un abcès à toxoplasme.
- ORL : dyspnée laryngée.

**DC/POSOLOGIE et MODE D'ADMINISTRATION**

Cette spécialité n'est pas adaptée à l'administration par voie inhalée par nébulisateur.
Équivalence anti-inflammatoire (équipotence) : 5 mg de prednisone = 4 mg de méthylprednisolone.
Injections intraveineuse ou intramusculaire.
La posologie est variable en fonction du diagnostic, de la sévérité de l'affection, du pronostic, de la réponse du patient et de la tolérance au traitement.
**- Solu-Médrol 20 mg et 40 mg :**
Après mélange, la solution obtenue peut être administrée directement par voie IM profonde, par voie IV lente ou par perfusion IV après dilution dans une solution de chlorure de sodium ou de glucose.

- Adulte : 20 à 60 mg par jour. Cette dose peut être renouvelée 2 à 3 fois par 24 heures, si nécessaire. Coût du traitement journalier : 1,84 à 5,52 € (Solu-Médrol 20 mg).
- Enfant : 1 à 3 mg/kg/jour. Coût du traitement journalier : 0,092 à 0,28 €/kg (Solu-Médrol 20 mg).

**- Solu-Médrol 120 mg :**
Réservé à l'adulte.

Après mélange, la solution obtenue est administrée par voie IV :

- soit directement en injection lente,
- soit par perfusion IV après dilution dans une solution isotonique de chlorure de sodium ou de glucose.

Lorsque la voie IV ne peut être utilisée, l'administration pourra être effectuée par voie IM profonde sous asepsie rigoureuse.
La posologie est de 120 mg par jour. Dans des situations très exceptionnelles, cette posologie pourra être répétée.
Coût du traitement journalier : 5,17 €.

**DC/CONTRE-INDICATIONS**

**Absolues :**

- Tout état infectieux à l'exclusion des indications spécifiées (cf Indications).
- Certaines viroses en évolution (notamment hépatites, herpès, varicelle, zona).
- États psychotiques encore non contrôlés par un traitement.
- Vaccins vivants.
- Hypersensibilité à l'un des constituants.
- Troubles de l'hémostase ou traitement anticoagulant en cours, en cas d'injection intramusculaire.

Il n'existe toutefois aucune contre-indication absolue pour une corticothérapie d'indication vitale.

**Relatives :**

- Médicaments non antiarythmiques donnant des torsades de pointes (cf Interactions).

**DC/MISES EN GARDE et PRÉCAUTIONS D'EMPLOI**

**Mises en garde :**

De rares cas de réactions pseudo-anaphylactiques étant survenus chez des patients traités par une corticothérapie parentérale, une attention particulière sera portée avant toute administration chez des sujets présentant un terrain atopique.
En cas d'ulcère gastroduodénal, la corticothérapie n'est pas contre-indiquée si un traitement anti-ulcéreux est associé.
En cas d'antécédents ulcéreux, la corticothérapie peut être prescrite, avec une surveillance clinique et au besoin après fibroscopie.
La corticothérapie peut favoriser la survenue de diverses complications infectieuses dues notamment à des bactéries, des levures et des parasites. La survenue d'une anguillulose maligne est un risque important. Tous les sujets venant d'une zone d'endémie (régions tropicale, subtropicale, sud de l'Europe) doivent avoir un examen parasitologique des selles et un traitement éradicateur systématique avant la corticothérapie.
Les signes évolutifs d'une infection peuvent être masqués par la corticothérapie.
Il importe, avant la mise en route du traitement, d'écarter toute possibilité de foyer viscéral, notamment tuberculeux, et de surveiller, en cours de traitement, l'apparition de pathologies infectieuses.
En cas de tuberculose ancienne, un traitement prophylactique antituberculeux est nécessaire s'il existe des séquelles radiologiques importantes et si l'on ne peut s'assurer qu'un traitement bien conduit de 6 mois par la rifampicine a été donné.
L'emploi des corticoïdes nécessite une surveillance particulièrement adaptée, notamment chez les sujets âgés et en cas de colites ulcéreuses (risque de perforation), diverticulites, anastomoses intestinales récentes, insuffisance rénale, insuffisance hépatique, ostéoporose, myasthénie grave.
L'attention est attirée chez les sportifs, cette spécialité contenant un principe actif pouvant induire une réaction positive des tests pratiqués lors des contrôles antidopage.

**Précautions d'emploi :**

Un traitement par voie orale sera institué en relais dès que possible.
Une rétention hydrosodée est habituelle, responsable en partie d'une élévation éventuelle de la pression artérielle. L'apport sodé sera réduit.
La supplémentation potassique n'est justifiée que pour des traitements à fortes doses, prescrits pendant une longue durée ou en cas de risque de troubles du rythme ou d'associations à un traitement hypokaliémiant.
Lorsque la corticothérapie est indispensable, le diabète et l'hypertension artérielle ne sont pas des contre-indications mais le traitement peut entraîner leur déséquilibre. Il convient de réévaluer leur prise en charge.
Les patients doivent éviter le contact avec des sujets atteints de varicelle ou de rougeole.

**DC/INTERACTIONS**

**Interactions médicamenteuses :**

*Associations déconseillées :*

- Médicaments donnant des torsades de pointes : astémizole, bépridil, érythromycine IV, halofantrine, pentamidine, sparfloxacine, sultopride, terfénadine, vincamine. Torsades de pointes (l'hypokaliémie est un facteur favorisant, de même que la bradycardie et un espace QT long préexistant). Utiliser des substances ne présentant pas l'inconvénient d'entraîner des torsades de pointes en cas d'hypokaliémie.

*Associations nécessitant des précautions d'emploi :*

- Acide acétylsalicylique par voie générale (et par extrapolation, autres salicylés) : diminution de la salicylémie pendant le traitement par les corticoïdes et risque de surdosage salicylé après son arrêt (augmentation de l'élimination des salicylés par les corticoïdes).
  Adapter les doses de salicylés pendant l'association et après l'arrêt du traitement par les corticoïdes.
- Antiarythmiques donnant des torsades de pointes (amiodarone, disopyramide, quinidiniques, sotalol) : torsades de pointes (l'hypokaliémie est un facteur favorisant, de même que la bradycardie et un espace QT long préexistant). Prévention de l'hypokaliémie et, si besoin, correction ; surveillance de l'espace QT. En cas de torsades, ne pas administrer d'antiarythmique (entraînement électrosystolique).
- Anticoagulants oraux : impact éventuel de la corticothérapie sur le métabolisme de l'anticoagulant oral et sur celui des facteurs de la coagulation. Risque hémorragique propre à la corticothérapie (muqueuse digestive, fragilité vasculaire) à fortes doses ou en traitement prolongé supérieur à 10 jours. Lorsque l'association est justifiée, renforcer la surveillance : contrôle biologique au 8 ^e^ jour, puis tous les 15 jours pendant la corticothérapie et après son arrêt.
- Autres hypokaliémiants : diurétiques hypokaliémiants (seuls ou associés), laxatifs stimulants, amphotéricine B (voie IV) : risque accru d'hypokaliémie (effet additif). Surveillance de la kaliémie, avec, si besoin, correction à prendre particulièrement en compte en cas de thérapeutique digitalique.
- Digitaliques : hypokaliémie favorisant les effets toxiques des digitaliques. Surveillance de la kaliémie et, s'il y a lieu, ECG.
- Héparines (voie parentérale) : aggravation par l'héparine du risque hémorragique propre à la corticothérapie (muqueuse digestive, fragilité vasculaire) à fortes doses ou en traitement prolongé supérieur à 10 jours. L'association doit être justifiée, renforcer la surveillance.
- Inducteurs enzymatiques : anticonvulsivants (carbamazépine, phénobarbital, phénytoïne, primidone), rifampicine. Diminution des concentrations plasmatiques et de l'efficacité des corticoïdes par augmentation de leur métabolisme hépatique. Les conséquences sont particulièrement importantes chez les addisoniens et en cas de transplantation. Surveillance clinique et biologique, adaptation de la posologie des corticoïdes pendant l'association et après arrêt de l'inducteur enzymatique.
- Insuline, metformine, sulfamides hypoglycémiants : élévation de la glycémie avec parfois cétose (diminution de la tolérance aux glucides par les corticoïdes). Prévenir le patient et renforcer l'autosurveillance sanguine et urinaire, surtout en début de traitement. Adapter éventuellement la posologie de l'antidiabétique pendant le traitement par les corticoïdes et après son arrêt.
- Isoniazide (décrit pour la prednisolone) : diminution des concentrations plasmatiques de l'isoniazide. Mécanisme invoqué : augmentation du métabolisme hépatique de l'isoniazide et diminution de celui des glucocorticoïdes. Surveillance clinique et biologique.

*Associations à prendre en compte :*

- Antihypertenseurs : diminution de l'effet antihypertenseur (rétention hydrosodée des corticoïdes).
- Ciclosporine : augmentation possible des concentrations plasmatiques de ciclosporine et de la créatinémie. Mécanisme invoqué : diminution de l'élimination hépatique de la ciclosporine.
- Interféron alpha : risque d'inhibition de l'action de l'interféron.
- Vaccins vivants atténués : risque de maladie généralisée éventuellement mortelle. Ce risque est majoré chez les sujets déjà immunodéprimés par la maladie sous-jacente.
  Utiliser un vaccin inactivé lorsqu'il existe (poliomyélite).

**DC/GROSSESSE et ALLAITEMENT**

**Grossesse :**

Chez l'animal, l'expérimentation met en évidence un effet tératogène variable selon les espèces.
Dans l'espèce humaine, il existe un passage transplacentaire. Cependant, les études épidémiologiques n'ont décelé aucun risque malformatif lié à la prise de corticoïdes lors du premier trimestre.
Lors de maladies chroniques, nécessitant un traitement tout au long de la grossesse, un léger retard de croissance intra-utérin est possible. Une insuffisance surrénale néonatale a été exceptionnellement observée après corticothérapie à doses élevées. Il est justifié d'observer une période de surveillance clinique (poids, diurèse) et biologique du nouveau-né.
En conséquence, les corticoïdes peuvent être prescrits pendant la grossesse si besoin.

**Allaitement :**

En cas de traitement à doses importantes et de façon chronique, l'allaitement est déconseillé.

**DC/EFFETS INDÉSIRABLES**

- De rares cas de réactions anaphylactiques ont pu être rapportés chez des patients traités par des corticostéroïdes par voie parentérale (cf Mises en garde/Précautions d'emploi). Des troubles du rythme cardiaque ont également été décrits, liés à l'administration intraveineuse.
- Désordres hydroélectrolytiques : hypokaliémie, alcalose métabolique, rétention hydrosodée, hypertension artérielle, insuffisance cardiaque congestive.
- Troubles endocriniens et métaboliques : syndrome de Cushing iatrogène, inertie de la sécrétion d'ACTH, atrophie corticosurrénalienne parfois définitive, diminution de la tolérance au glucose, révélation d'un diabète latent, arrêt de la croissance chez l'enfant, irrégularités menstruelles.
- Troubles musculosquelettiques : atrophie musculaire précédée par une faiblesse musculaire (augmentation du catabolisme protidique), ostéoporose, fractures pathologiques, en particulier tassements vertébraux, ostéonécrose aseptique des têtes fémorales.
- Troubles digestifs : ulcères gastroduodénaux, ulcérations du grêle, perforations et hémorragies digestives ; des pancréatites aiguës ont été signalées, surtout chez l'enfant.
- Troubles cutanés : acné, purpura, ecchymoses, hypertrichose, retard de cicatrisation.
- Troubles neuropsychiques :
  - fréquemment : euphorie, insomnie, excitation ;
  - rarement : accès d'allure maniaque, états confusionnels ou confuso-oniriques, convulsions ;
  - état dépressif à l'arrêt du traitement.
- Troubles oculaires : certaines formes de glaucome et de cataracte.

**PP/PHARMACODYNAMIE**

Glucocorticoïde, usage systémique (H : hormones non sexuelles ; D : dermatologie ; M : système locomoteur ; S : organes sensoriels).
Les glucocorticoïdes physiologiques (cortisone et hydrocortisone) sont des hormones métaboliques essentielles. Les corticoïdes synthétiques incluant la méthylprednisolone sont utilisés principalement pour leur effet anti-inflammatoire. A forte dose, ils diminuent la réponse immunitaire. Leur effet métabolique et de rétention sodée est moindre que celui de l'hydrocortisone.

**PP/PHARMACOCINÉTIQUE**

Diffusion rapide ; demi-vie : 3 h 30.
L'élimination est à la fois urinaire et biliaire.
Passage dans le lait et à travers le placenta.

**DP/CONDITIONS PARTICULIÈRES DE CONSERVATION**

*Après reconstitution :* la solution doit être utilisée extemporanément.

LISTE I

| AMM | 309 717.0 (1991 rév 1998) 20 mg, fl + solv. |
| --- | --- |
|  | 309 718.7 (1991 rév 1998) 40 mg, fl + solv. |
|  | 315 528.1 (1991 rév 1998) 120 mg, fl + solv. |
|  | 558 648.2 (1995 rév 1998) 20 mg, 10 fl. |
|  | 558 650.7 (1995 rév 1998) 40 mg, 10 fl. |
|  | 558 653.6 (1995 rév 1998) 120 mg, 10 fl. Mis sur le marché en 1962 et 1969 (120 mg). |

| **PRIX :** | 1,84 € (flacon de 20 mg + solvant). |
| --- | --- |
|  | 2,50 € (flacon de 40 mg + solvant). |
|  | 5,17 € (flacon de 120 mg + solvant). |

Remb Séc soc à 65 %. Collect.
Collect (boîtes de 10 flacons).

3-ZOPHREN® solution injectable ondansétron**: Laboratoire GlaxoSmithKline**

**FORMES et PRÉSENTATIONS**

*Solution injectable IV à 2 mg/ml :*
Ampoule de 2 ml, boîte unitaire.
Ampoule de 4 ml, boîte unitaire.
Modèle hospitalier : Boîtes de 5.

COMPOSITION

|  | *p ampoule* | |
| --- | --- | --- |
|  | *de 2 ml* | *de 4 ml* |
| Ondansétron (DCI) chlorhydrate dihydraté exprimé en ondansétron | 4 mg | 8 mg |

*Excipients :* acide citrique monohydraté, citrate de sodium, chlorure de sodium (9 mg/ml), eau ppi.

**DC/INDICATIONS**

- Prévention et traitement des nausées et vomissements aigus induits par la chimiothérapie cytotoxique moyennement à hautement émétisante et la radiothérapie hautement émétisante chez l'adulte.
- Prévention des nausées et vomissements aigus induits par la chimiothérapie cytotoxique moyennement à hautement émétisante chez l'enfant.
- Traitement des nausées et vomissements postopératoires chez l'adulte et l'enfant.

**DC/POSOLOGIE et MODE D'ADMINISTRATION**

**Posologie :**

**- Adulte à partir de 15 ans :**
**- Nausées et vomissements induits par les traitements cytotoxiques :**
La dose initiale habituelle est de 8 mg administrée soit en IV lente 30 minutes avant la chimiothérapie ou la radiothérapie, soit en comprimé ou sirop 2 heures avant la chimiothérapie moyennement émétisante ou la radiothérapie.
Dans certaines circonstances (utilisation de drogues cytotoxiques très émétisantes et/ou prescrites à très fortes doses, facteurs liés au patient tels que sujet jeune, de sexe féminin, ayant l'expérience de phénomènes émétiques lors de précédents traitements cytotoxiques...), une dose plus élevée (32 mg en IV lente sur plus de 15 minutes avant le début du traitement cytotoxique, ou 8 mg en IV lente suivis d'une perfusion de 1 mg/heure sur 24 heures ou 8 mg en IV lente suivis de 2 injections de 8 mg en IV lente à 4 heures d'intervalle) et/ou une association à une corticothérapie pourront être utilisées d'emblée.
**- Nausées et vomissements postopératoires :**
4 mg en IV lente.
**- Enfant de plus de 2 ans :**
**- Nausées et vomissements induits par les traitements cytotoxiques :**
La dose initiale est de 5 mg/m ^2^ administrée en IV lente juste avant la chimiothérapie.
**- Nausées et vomissements postopératoires :**
0,1 mg/kg en IV lente unique jusqu'à un maximum de 4 mg.
Coût d'une injection :

11,86 € (4 mg) ; 22,24 € (8 mg).
**- Sujet âgé :**
Chez le sujet âgé de plus de 65 ans, l'efficacité et la tolérance ont été semblables à ce qui est observé chez l'adulte plus jeune.
**- Insuffisant hépatique :**
Il est recommandé de ne pas dépasser une dose totale journalière de 8 mg chez ces patients.
**- Patient métaboliseur lent :**
Le métabolisme de la spartéine et de la débrisoquine au niveau du cytochrome P450 n'est pas modifié. Aucune adaptation posologique n'est donc nécessaire chez ce type de patients.

**Mode d'administration :**
**- Compatibilité avec les liquides de perfusion :**
En accord avec les bonnes pratiques pharmaceutiques, les solutions de perfusion doivent être préparées extemporanément. Toutefois, il a été prouvé que l'ondansétron injectable est stable 7 jours à température ambiante (au-dessous de 25 °C) sous éclairage fluorescent ou dans un réfrigérateur lorsqu'il est dilué dans les liquides de perfusion suivants :

- chlorure de sodium à 0,9 %,
- solution glucosée à 5 %,
- solution de mannitol à 10 %,
- solution de Ringer,
- solution de chlorure de potassium à 0,3 % + chlorure de sodium à 0,9 %,
- solution de chlorure de potassium à 0,3 % + solution glucosée à 5 %.

Des études de compatibilité ont été effectuées, les solutions d'ondansétron sont stables :

- dans les poches à perfusion en chlorure de polyvinyle,
- dans les nécessaires de perfusion en chlorure de polyvinyle,
- dans les poches de perfusion en polyéthylène,
- dans les flacons en verre de type 1.

Les solutions d'ondansétron dans une solution de chlorure de sodium à 0,9 % ou de glucose à 5 % administrées dans des seringues en polypropylène sont stables. On peut donc considérer que l'ondansétron injectable, dilué avec les autres liquides de perfusion compatibles, est stable dans des seringues en polypropylène.
Remarque : la préparation doit être faite dans des conditions aseptiques appropriées.

**- Compatibilité avec d'autres produits :**
L'ondansétron peut être administré en perfusion intraveineuse à partir d'une poche de perfusion ou d'une seringue électrique.

Les produits suivants peuvent être administrés simultanément au niveau d'une perfusion en Y pour peu que les concentrations d'ondansétron soient comprises entre 16 µg/ml et 160 µg/ml (c'est-à-dire 8 mg pour 500 ml et 8 mg pour 50 ml respectivement) :

- Cisplatine : à des concentrations n'excédant pas 0,48 mg/ml (c'est-à-dire 240 mg dans 500 ml) administrées sur 1 à 8 heures.
- 5-fluoro-uracile : à des concentrations n'excédant pas 0,8 mg/ml (c'est-à-dire 2,4 g dans 3 litres ou 400 mg dans 500 ml) administrées à un débit d'au moins 20 ml par heure (500 ml par 24 heures). Des concentrations plus élevées de 5-fluoro-uracile peuvent provoquer une précipitation d'ondansétron. Les perfusions de 5-fluoro-uracile peuvent contenir jusqu'à 0,045 % de chlorure de magnésium en addition des autres excipients compatibles.
- Carboplatine : à des concentrations allant de 0,18 mg/ml à 9,9 mg/ml (c'est-à-dire 90 mg dans 500 ml à 990 mg dans 100 ml), administrées sur une période de 10 minutes à une heure.
- Étoposide : à des concentrations allant de 0,14 mg/ml à 0,25 mg/ml (c'est-à-dire 72 mg dans 500 ml à 250 mg dans 1 litre), administrées sur une période de 30 minutes à une heure.
- Cyclophosphamide : à des doses de 100 mg à 1 g diluées avec de l'eau pour préparations injectables, 5 ml pour 100 mg de cyclophosphamide selon les recommandations du fabricant et administrées en bolus IV sur environ 5 minutes.
- Doxorubicine : à des doses de 10 à 100 mg reconstituées avec de l'eau pour préparations injectables, 5 ml pour 10 mg de doxorubicine selon les recommandations du fabricant et administrées en bolus IV sur environ 5 minutes.
- Ceftazidime : à des doses de 250 mg à 2000 mg diluées dans de l'eau pour préparations injectables selon les recommandations du fabricant (c'est-à-dire 2,5 ml pour 250 mg et 10 ml pour 2 g de ceftazidime) et administrées en bolus IV sur environ 5 minutes.

**DC/CONTRE-INDICATIONS**

Allergie à l'un des composants.

**DC/MISES EN GARDE et PRÉCAUTIONS D'EMPLOI**

**Mises en garde :**

Un bilan cardiovasculaire doit être effectué en cas de survenue de douleurs thoraciques et de syncope, ou de troubles du rythme cardiaque.
Prendre en compte le risque éventuel d'hypersensibilité croisée avec les autres antagonistes des récepteurs 5 HT_3_.

**Précautions d'emploi :**

En cas d'insuffisance hépatique sévère, les paramètres pharmacocinétiques de l'ondansétron sont significativement modifiés : réduction de la clairance plasmatique totale, augmentation de la demi-vie plasmatique.
L'ondansétron pouvant favoriser un syndrome occlusif, il convient de surveiller attentivement le transit des patients en cours de traitement (cf Effets indésirables).
En cas de régime désodé ou hyposodé, tenir compte de la teneur en sodium (cf Composition).

**DC/GROSSESSE et ALLAITEMENT**

**Grossesse :**

Les études effectuées chez l'animal n'ont pas mis en évidence d'effet tératogène de l'ondansétron.
En l'absence d'effet tératogène chez l'animal, un effet malformatif dans l'espèce humaine n'est pas attendu. En effet, à ce jour, les substances responsables de malformations dans l'espèce humaine se sont révélées tératogènes chez l'animal au cours d'études bien conduites sur deux espèces.
En clinique, il n'existe pas actuellement de données suffisamment pertinentes pour évaluer un éventuel effet malformatif ou fœtotoxique de l'ondansétron lorsqu'il est administré pendant la grossesse.
En conséquence, par mesure de précaution, il est préférable de ne pas utiliser l'ondansétron pendant la grossesse.

**Allaitement :**

En cas d'allaitement ou de désir d'allaitement et compte tenu du passage de l'ondansétron dans le lait maternel, l'utilisation de ce produit est déconseillée.

**DC/EFFETS INDÉSIRABLES**

Les effets indésirables suivants peuvent survenir :

- céphalées, bouffées de chaleur ou flush, hoquets,
- rarement, anomalies biologiques hépatiques,
- constipation pouvant, dans de rares cas, se compliquer d'iléus ou d'occlusion intestinale, en particulier chez des patients présentant des facteurs de risque associés : ralentisseurs du transit, antécédent de chirurgie digestive...,
- hypotension, douleurs thoraciques avec ou sans décalage du segment ST, troubles du rythme et bradycardie,
- réactions extrapyramidales telles que crises oculogyres, dystonies sans séquelles cliniques ou convulsions,
- réactions allergiques immédiates, quelquefois sévères, incluant des réactions anaphylactiques,
- troubles visuels transitoires et vertiges au cours des injections IV rapides,
- possibilité de réactions au point d'injection ou le long de la veine perfusée (érythème, urticaire, prurit, douleur) et rares cas de veinites.

**DC/SURDOSAGE**

Un surdosage en ondansétron peut entraîner les effets indésirables déjà mentionnés dans la rubrique Effets indésirables. Il n'existe pas d'antidote spécifique de l'ondansétron. Par conséquent, en cas de surdosage, seule une thérapeutique symptomatique appropriée sera instaurée.

**PP/PHARMACODYNAMIQUE**

Antagoniste de la sérotonine (A04AA01 : appareil digestif et métabolisme).
L'ondansétron est un antagoniste des récepteurs 5HT_3_ à la sérotonine, impliqués dans les phénomènes de réflexe émétique.
L'administration d'ondansétron ne modifie pas les taux sériques de prolactine.

**PP/PHARMACOCINETIQUE**

L'ondansétron est métabolisé et les métabolites sont excrétés dans les fèces et l'urine.
Après administration IV, le pic sérique est atteint en environ 15 minutes. La concentration au pic est de 95,6 ng/ml.
La demi-vie d'élimination est d'environ 3 heures ; cependant, elle peut être prolongée jusqu'à 5 heures chez le sujet âgé.
Liaison aux protéines : 70 à 76 %.

**DP/COMPATIBILITES**

L'ondansétron injectable est incompatible avec les solutions bicarbonatées.
Il ne doit pas être administré dans la même seringue ou la même perfusion que d'autres médicaments.
Il ne doit être administré qu'avec les liquides de perfusion recommandés.
Il ne doit pas être autoclavé.

**DP/CONDITIONS PARTICULIERES DE CONSERVATION**

Ce médicament doit être conservé à une température inférieure à 25 °C et à l'abri de la lumière dans son étui d'origine.

**LISTE1**

| AMM | 335 392.8 (1992 rév 09.05.2001) 1 amp 4 mg. |
| --- | --- |
|  | 556 831.4 (1990 rév 09.05.2001) 5 amp 4 mg. |
|  | 335 393.4 (1992 rév 09.05.2001) 1 amp 8 mg. |
|  | 556 832.0 (1990 rév 09.05.2001) 5 amp 8 mg. Mis sur le marché en 1990. |

| **PRIX :** | 11,86 € (1 amp à 4 mg). |
| --- | --- |
|  | 22,24 € (1 amp à 8 mg). |

Remb Séc Soc à 65 % selon la procédure des médicaments d'exception (prescription en conformité avec la fiche d'information thérapeutique). Collect.

**Note d’information au patient**

Le Dr …………………exerçant dans le service …………………….. ……de l’Hôpital ………………………. vous propose de participer au protocole :

ESSAI THERAPEUTIQUE RANDOMISE MULTICENTRIQUE EN DOUBLE INSU EN GROUPES PARALLELES

DU CYCLOPHOSPHAMIDE INTRAVEINEUX VERSUS METHYLPREDNISOLONE DANS LE TRAITEMENT DE FOND DES FORMES PROGRESSIVES SECONDAIRES RECENTES DE SCLEROSE EN PLAQUES : ETUDE P.R.OM.E.S.S

Version 1.0 du 09/01/2008

**Promoteur : Centre Hospitalier Universitaire de Bordeaux**

### Investigateur Coordonnateur : Pr. Bruno Brochet

***Merci de lire attentivement ce document d’information, il nous servira de base pour les discussions ultérieures. Vous trouverez un glossaire des termes médicaux spécifiques à la fin du formulaire de consentement pour une meilleure compréhension de cette étude****.*

**Partie 1 - Pourquoi cette étude ?**

Vous présentez une sclérose en plaques qui est une maladie inflammatoire du système nerveux. Cette maladie se présente sous différentes formes évolutives et les traitements de ces différentes formes ne sont pas identiques. La forme actuelle de votre maladie est ce qu’on appelle la forme secondairement progressive ce qui veut dire que l’aggravation que vous avez présenté l’année dernière n’était pas uniquement (ou pas du tout) due à des séquelles de nouvelles poussées mais à une aggravation progressive en particulier des troubles de la marche.

Actuellement il existe plusieurs traitements validés pour les formes rémittentes (c'est-à-dire uniquement par poussées) de sclérose en plaques. Les traitements disponibles à la phase progressive sont moins nombreux et d’efficacité inconstante. Deux interférons disposent d’une autorisation de mise sur le marché, le bétaféron ® pour les patients ayant une forme secondairement progressive et le rebif ® pour les patients ayant une forme secondairement progressive avec poussées surajoutées. Ces produits peuvent stabiliser la maladie d’un certain pourcentage de patients dans cette situation mais ne sont pas utiles chez ceux qui ont débuté leur aggravation progressive alors qu’ils étaient déjà sous interféron auparavant. Ils ne sont pas efficaces chez tous les patients ayant une forme secondairement progressive. Un autre médicament l’Elsep ® (mitoxantrone) a reçu une autorisation de mise sur le marché pour les patients ayant une forme agressive de SEP définie par certains critères cliniques et d’IRM. La majorité des patients ayant une forme secondairement progressive ne réunit pas ces critères et ne relève donc pas de ce traitement mais dans le cas où vous réuniriez les critères d’indication d’Elsep ® votre neurologue vous en informera avant votre décision de participer ou non au présent protocole.

Depuis plusieurs années plusieurs centres spécialisés dans la prise en charge de la SEP utilisent le cyclophosphamide (endoxan®) par cures de perfusions mensuelles dans cette indication. Ce médicament est un médicament immunosuppresseur qui agit sur les globules blancs responsables de l’inflammation présente dans cette maladie. Aux doses utilisées son action est en fait essentiellement immunomodulatrice c'est-à-dire qu’il diminue l’inflammation sans diminuer de façon importante le nombre de globules blancs. Deux études dites « ouvertes », c'est-à-dire sans groupe contrôle, ont été publiées ces dernières années sur un total de 585 patients ayant une sclérose en plaques montrant des résultats très encourageants avec ce traitement puisque dans ces études 73 à 78% des patients ne se sont pas aggravés. Aucune étude contrôlée n’a à ce jour été réalisée à ces doses dans cette indication. Des études contrôlées avaient été réalisées en utilisant des doses et des schémas d’administration différents il y a quelques années avec des résultats contradictoires mais ces études utilisaient un traitement sur quelques jours sans traitement d’entretien. Des études pilotes ont été réalisées ces dernières années dans d’autres formes de la SEP avec ce médicament avec des résultats positifs. Il est donc d’une grande importance de réaliser une étude contrôlée pour déterminer l’efficacité de ce traitement.

**Quel est l'objectif de cette étude ?**

**L'objectif principal de cette étude est donc de comparer l’efficacité du traitement par du cyclophosphamide (endoxan®) par rapport à un traitement par méthylprednisolone (Solumédrol®) administrés par voie intraveineuse.** Ce protocole a été élaboré, relu et approuvé par les experts du comité scientifique de l’étude. Il a été soumis et approuvé par les experts du programme hospitalier de recherche clinique national.

**Partie 2 - Comment va se dérouler cette étude ?**

La durée prévue de l’étude est de 5 ans soit 3 ans de recrutement des patients y participant et deux ans de traitement au sein de l’étude. 6 mois supplémentaires seront ensuite nécessaires pour l’analyse des résultats. La participation de chaque patient est de 100 semaines (environ 2 ans). Il est prévu de faire participer 360 patients à cette étude dans plusieurs services hospitaliers de neurologie en France. Tous les patients acceptant de participer à cette étude devront avoir entre 18 et 65 ans inclus et  une forme secondairement progressive de SEP dont la phase d’aggravation progressive ne doit pas avoir dépassé 4 ans avec certains critères, en particulier l’aggravation récente doit correspondre à une réduction du périmètre de marche (distance de marche sans arrêt) dans les 12 derniers mois non attribuable à des séquelles de poussées. Ne peuvent participer à l’étude les patients ayant des antécédents de cystite

hémorragique, d’allergie connue au cyclophosphamide (endoxan®), aux corticoïdes et en particulier au méthylprednisolone (Solumédrol ®)  ou ayant une maladie infectieuse en cours, ainsi que les patients porteurs d’une cathétérisation permanente de la vessie (sondage urinaire à demeure). Les femmes enceintes ou allaitantes ne peuvent participer à l’étude et une contraception efficace doit être envisagée pour les femmes en âge de procréer (voir plus loin). Les patients ayant déjà été traités antérieurement par du cyclophosphamide (endoxan®), une irradiation totale lymphoïde, un anticorps monoclonal anti-CD4 ou anti-CD52 ou anti-VLA4, de la cladribine ou la cyclosporine A ne peuvent pas participer à l’étude.	Le traitement antérieur par mitoxantrone est un critère de non-inclusion sauf si celui-ci a été effectué durant la phase rémittente et plus de 3 ans avant le début de l’étude. Dans ce cas là, un bilan des Numérations Formule Sanguine et de l’échographie cardiaque transthoracique devra être fourni.

En cas de traitement antérieur par interféron bêta, imurel ou méthotrexate, un intervalle d’au moins un mois doit être respecté entre l’arrêt de ce traitement et le début du traitement à l’étude.

Si vous recevez un traitement contre l’hypercholestérolémie, avertissez en votre neurologue, certaines restrictions pouvant intervenir. Les patients participant à l’étude seront répartis en deux groupes par tirage au sort sans qu’eux-mêmes ou les médecins les suivant ne sache dans quel groupe ils auront été affecté (double insu). Les patients du premier groupe recevront le traitement par cyclophosphamide (endoxan ®) et les patients du second groupe recevront de la méthylprednisolone (solumédrol ®) au même rythme. Aucun patient ne reçoit de placebo.
Le traitement de comparaison est un traitement corticoïde qui a été choisi pour ne pas exposer le groupe de comparaison à un placebo. Le solumédrol ® est le traitement habituel des poussées de SEP sous formes de perfusions pendant plusieurs jours consécutifs. Sous la forme de perfusions tous les deux mois il a fait l’objet d’une étude dans le traitement des formes secondairement progressive de SEP en le comparant pendant 2 ans à de faibles doses de corticoïdes chez 109 patients. Une tendance favorable mais non significative a été observée mais l’analyse de l’évolution du handicap a montré un effet significatif du traitement. Le traitement administré dans ce groupe est donc probablement supérieur à l’abstention thérapeutique.

Le traitement par cyclophosphosphamide (endoxan®) ou par méthylprednisolone (solumédrol ®) est injecté dans une perfusion intraveineuse sur 3 heures toutes les 4 semaines pendant 44 semaines puis toutes les 8 semaines jusqu’à la semaine 92.
Le bénéfice attendu est une supériorité du traitement par cyclophosphosphamide (endoxan®) par rapport au traitement par méthylprednisolone (solumédrol ®) pour empêcher l’aggravation du handicap après deux ans de traitement. Cet objectif sera analysé en utilisant une échelle de mesure clinique reposant sur l’examen neurologique et la mesure du périmètre de marche appelée EDSS et fréquemment utilisé dans le suivi de la SEP et dans les essais thérapeutiques concernant cette maladie. La constatation d’une aggravation de ce score confirmée à deux examens successifs à 4 mois d’écart servira à définir cette aggravation du handicap.
En cas d’aggravation confirmée du handicap le patient en sera informé et il pourra rediscuter les choix thérapeutiques éventuels avec son neurologue. Il lui sera proposé également de poursuivre l’étude jusqu’à son terme s’il le souhaite.
En cas de suspicion de poussée vous devez contacter dès que possible le neurologue qui vous suit dans l’étude. Le neurologue vous demandera de venir au centre pour une visite non programmée au plus tard 7 jours après votre appel. Si nécessaire il vous proposera le traitement nécessaire pour la poussée (solumédrol ®).

**Partie 3 - Quels sont les inconvénients possibles ?**

La participation à cette étude impose certaines contraintes. Les traitements sont administrés dans des perfusions intraveineuses en milieu hospitalier. La durée totale de perfusion est de 8 heures. Une hospitalisation est donc nécessaire pour administrer ces perfusions toutes les 4 semaines pendant 44 semaines puis toutes les 8 semaines pendant 48 semaines. Une à deux visites interviendront avant la première perfusion et une visite 8 semaines après la dernière perfusion. Au cours des visites ou hospitalisations deux neurologues seront chargés de vous suivre. L’un (neurologue évaluateur) sera chargé de l’examen neurologique, de la mesure du périmètre de marche, et de tests neurologiques. Vous ne devrez lui communiquer aucune information sur les éventuels effets indésirables du traitement. Il pourra se faire aider d’une infirmière ou d’un assistant de recherche. L’autre (appelé neurologue traitant) sera chargé de votre suivi médical et recueillera tous les symptômes et signes éventuellement imputables au traitement reçu. C’est lui que vous devez contacter en cas de poussée.

Des prises de sang seront réalisées régulièrement au cours de l’étude afin de surveiller en particulier la numération des globules sanguins, le ionogramme sanguin, la glycémie, créatininémie, les transaminases et pour les femmes en âge de procréer des test de grossesse). Une prise de sang sera faite avant le début du traitement et une entre chaque hospitalisation. Un examen cytobactériologique des urines sera effectué en même temps. Avant chaque perfusion un électrocardiogramme sera réalisé et une analyse d’urines sur bandelettes.

Avant le début de l’étude, une intradermoréaction à la tuberculine (IDR), des radiographies simples des poumons, des sinus et des dents et une échographie de la vessie seront réalisés. Cette étude vous est proposée parce que les médecins qui vous soignent considèrent que les avantages liés au traitement à l’essai sont supérieurs aux inconvénients. Néanmoins, un certain nombre d’effets indésirables ont été décrits avec le cyclophosphamide (endoxan®) et nécessite une surveillance attentive. Cependant ces effets sont rares aux doses utilisées. Dans l’étude rétrospective française portant sur 490 patients ayant une SEP progressive et traités à la même dose que dans cette étude, seuls 4,5% des patients ont présenté un effet secondaire sévère, nausées et vomissements, leucopénie (baisse des globules blancs importante), infections, quelques pertes de cheveux réversibles et augmentation des enzymes hépatiques. Seul un patient a du interrompre ce traitement du fait de ces effets secondaires (augmentation prolongée des enzymes hépatiques).

Effets hématologiques : une baisse des globules blancs et plus rarement des plaquettes est fréquemment observée après la perfusion. La baisse des lymphocytes est souhaitée et reflète l’efficacité du traitement. Ces baisses sont réversibles rapidement et spontanément mais dans certains cas peuvent nécessiter la diminution de la dose voire l'arrêt du traitement. Cette baisse des globules blancs est le plus souvent modérée et sans conséquences. Elle n’impose pas de contraintes particulières en dehors de la réalisation des numérations sanguines. Dans de rares cas, cette baisse des globules blancs est plus importante et pourrait favoriser une infection. En cas de fièvre

ou de fatigue inexpliquée brutale il faut prévenir votre médecin pour qu’un contrôle de la numération sanguine soit effectué. Un traitement antibiotique pourrait être indiqué. De façon exceptionnelle, des leucémies et des tumeurs ont été décrites chez des patients traités par cyclophosphamide (endoxan®) après des traitements prolongés et à des doses très supérieures à celles utilisées dans la sclérose en plaques. Le risque, s’il existe, paraît très faible. Un risque de cancer de la vessie a été rapporté chez les patients ayant une SEP et ayant une sonde urinaire à demeure (ce qui constitue un critère de non inclusion) et ayant reçu des doses cumulées de plus de 50 grammes de cyclophosphamide (endoxan®) ce qui est très supérieur aux doses proposées. Les doses cumulées maximales seront dans cette étude inférieures à 25 grammes.

- Effets allergiques : comme pour tout médicament des épisodes d’allergie ont été rarement signalés notamment des érythèmes (rougeurs), exceptionnellement, des réactions anaphylactiques.

- Effets gastro-intestinaux : des nausées et des vomissements sont possibles lors des perfusions surtout à des doses plus élevées. Cependant pour les éviter un traitement par un *antiémétique* (zophren ®) sera administré en même temps que l’endoxan®. Des inflammations de la cavité buccale ont été observées à des doses supérieures.

- Grossesse et vie hormonale : le traitement est contre-indiqué pendant la grossesse en raison du risque de fausse couche spontanée et du risque de malformation possible pour le bébé. Il est également contre-indiqué durant l’allaitement. Des modifications du rythme menstruel et des aménorrhées ont également été décrites chez la femme. Les femmes en âge de procréer doivent accepter de recourir à une contraception efficace durant toute la durée de l’étude et pendant les 3 mois après l’arrêt de celle-ci et doivent avoir un test de grossesse négatif à l’inclusion. De rares azoospermies ont été observées chez l’homme. Une conservation de votre sperme peut être envisagée avant le début du traitement pour un éventuel projet parental par le CECOS (centre d’étude pour la conservation des œufs et de sperme humain). Les hommes susceptibles de procréer doivent accepter de recourir à une contraception efficace (préservatif ou contraception efficace du partenaire) durant toute la durée de l’étude.

- Vessie : afin d’éviter les rares inflammations de la vessie et cystites hémorragiques observées surtout à fortes doses, une perfusion de sérum physiologique est administrée pour assurer une diurèse suffisante.

- Peaux et cheveux : Une perte de quelques cheveux est possible au cours du traitement mais les rares alopécies sont observées à plus fortes doses.

Exceptionnellement, ont été rapportés : une cardiomyopathie aiguë, une fibrose pulmonaire.

Les perfusions de méthylprednisolone (solumédrol ®) seront administrées aux doses habituellement utilisées dans la SEP qui sont le plus souvent très bien tolérées mais peuvent occasionnellement exposer à des effets indésirables : De rares cas de réactions anaphylactiques (allergique) ont pu être rapportés chez des patients traités par des corticostéroïdes par voie injectable. Des troubles du rythme cardiaque ont également été décrits, liés à l'administration intraveineuse, en particulier si la vitesse de la perfusion est trop rapide.

Les effets secondaires habituels des corticoïdes au long cours sont rarement observés : Désordres hydroélectrolytiques, rétention hydrosodée, hypertension artérielle, insuffisance cardiaque, troubles endocriniens et métaboliques, troubles musculosquelettiques, ostéoporose, troubles digestifs, ulcères gastroduodénaux, ulcérations du grêle, perforations et hémorragies, troubles cutanés,  acné. Des troubles neuropsychiques à type d’euphorie, insomnie, excitation peuvent survenir et plus rarement des accès d'allure maniaque.

En cas d’arrêt ou de sortie d’étude prématurée votre neurologue vous proposera le traitement de votre maladie le plus adapté à votre cas en fonction des connaissances médicales du moment.

Le neurologue traitant prendra le temps nécessaire pour vous expliquer les effets secondaires du traitement et la façon de les prévenir. Il restera à votre disposition au cours de l’essai. N’hésitez pas à lui signaler tout effet attendu ou inattendu.

**Partie 4 - Que vous demandera-t-on ?**

Si, après avoir lu cette note d’information et avoir discuté de l’étude avec votre médecin, vous décidez d’y participer, vous devrez signer un formulaire confirmant votre accord de participation. Vous serez alors « pré inclus » et on effectuera une évaluation neurologique et des tests biologiques pour voir si vous pouvez être définitivement inclus dans l’étude. Si vos tests ne correspondent pas aux règles d’inclusion dans l’étude, votre participation à l’étude s’arrêtera et vous déciderez avec votre médecin du meilleur traitement à recevoir dans votre cas. Si vous réunissez les critères des tests complémentaires seront réalisés (radiographies, échographie, intradermo-réaction IDR) et chez les femmes, un test de grossesse sera effectué. Vous serez ensuite convoqués pour la première perfusion.

Le tableau vous décrit le calendrier des visites prévues.

**• : L’examen neurologique ainsi que la mesure du périmètre de marche pourront être réalisés une visite sur deux uniquement pour les visites n°3 à 12.**

A la fin de l’étude, votre neurologue vous proposera le traitement de votre maladie le plus adapté à votre cas en fonction des connaissances médicales du moment.

**Partie 5 - Que se passera-t-il en cas de survenue d’effets indésirables ?**

En cas d’effet secondaire peu important en particulier biologique la dose cyclophosphamide (endoxan®) sera ajustée par le pharmacien de l’hôpital. En cas d’effet secondaire grave (par exemple, baisse importante des globules blancs), le traitement sera provisoirement arrêté jusqu’à ce que la prise de sang revienne à des valeurs autorisant le traitement. Si la baisse est très importante votre médecin en sera informé et vous proposera, si nécessaire, un bilan sanguin toutes les deux semaines jusqu’à ce que l’effet disparaisse. Si l’effet secondaire persiste mais ne progresse pas après réduction des doses, le traitement sera poursuivi à dose réduite.

En cas de présence de germes dans les urines sans signes d’infection le traitement pourra être administré mais sous couverture d’un antiseptique urinaire. En cas d’infection urinaire symptomatique, un traitement antibiotique vous sera prescrit en fonction des germes présents et l’administration du traitement à l’essai sera reportée de 7 jours.

**Partie 6 - Quels sont vos droits ?**

Votre médecin doit vous fournir tous les détails concernant cette étude.

Votre participation à cette étude est entièrement volontaire. Si vous ne désirez pas prendre part à cette étude ou si vous souhaitez vous en retirer à quelque moment que ce soit, et quel que soit le motif, vous continuerez à bénéficier du suivi médical et cela n'affectera en rien votre surveillance future. Si vous décidez de participer à cette étude, votre médecin peut décider de vous faire arrêter les médicaments de l’essai à tout moment, en continuant une surveillance, si par exemple votre état de santé se dégrade et que d'autres traitements sont préférables ou si des effets secondaires le justifient.

Les données recueillies au cours de cette étude resteront strictement confidentielles, vos nom et adresse ne seront connus que de votre médecin. Compte tenu des nécessités de la recherche et de son analyse ultérieure, les données recueillies qui vous concernent feront l'objet d'un traitement informatisé et anonyme à l’unité de soutien méthodologique du CHU de Bordeaux. Les informations qui feront l’objet de ce traitement informatisé seront les suivantes : âge, sexe, antécédents médicaux, histoire de votre maladie, les traitements pris, les différentes évaluations réalisées au cours de l’étude, les événements cliniques et les résultats biologiques. Les articles 39, 40 et 56 de la loi 2004-801 du 6 août 2004 de la loi « Informatique et Libertés » prévoit votre droit d'accès, d'opposition et de rectification des données enregistrées sur informatique, à tout moment, par l’intermédiaire de votre médecin. De plus la loi du 4 mars 2002, garantit aux personnes ayant subi un préjudice du fait de la participation à une recherche avec bénéfice individuel direct, une indemnisation grâce à l'Office national d'indemnisation des accidents médicaux.

Cette étude a reçu un avis favorable du comité de protection des personnes Sud-ouest et Outre Mer III de Bordeaux après examen lors de sa séance du 30/03/2005. Le promoteur de cette étude est le CHU de Bordeaux, 12 rue Dubernat, 33400 Talence qui a souscrit une assurance de responsabilité civile conformément à la loi. Lorsque cette étude sera terminée, vous serez tenu informé personnellement des résultats par votre médecin dès que ceux-ci seront disponibles. Après avoir lu cette note d’information, n'hésitez pas à poser à votre médecin toutes les questions que vous désirez.

***Si vous acceptez de participer à cette étude, il vous suffit de signer le formulaire de consentement de participation*.**

**Un exemplaire du document complet vous sera remis. Nous vous remercions de votre collaboration à cette étude.**

**Nom du médecin ayant remis la note d'information : ……………………… Hôpital :………………………………**

**Date  :	Paraphe du patient :	Paraphe du médecin :**

**ANNEXE 4 : FORMULAIRE DE CONSENTEMENT**

ESSAI THERAPEUTIQUE RANDOMISE MULTICENTRIQUE EN DOUBLE INSU EN GROUPES PARALLELES

DU CYCLOPHOSPHAMIDE INTRAVEINEUX VERSUS METHYLPREDNISOLONE DANS LE TRAITEMENT DE FOND DES FORMES PROGRESSIVES SECONDAIRES RECENTES DE SCLEROSE EN PLAQUES : ETUDE P.R.OM.E.S.S

**Promoteur : Centre Hospitalier Universitaire de Bordeaux**

### Investigateur Coordonnateur : Pr. Bruno Brochet

Version du 1.0 du 04/01/2008

Je soussigné(e)................................................................(nom, prénom), certifie avoir lu et compris le document d’informations qui m’a été remis et avoir eu la possibilité de poser toutes les questions que je souhaitais au Dr ……………………………….

Je comprends les contraintes (en particulier les visites régulières à l’hôpital *et les différents examens)* liées à ma participation.

Je connais la possibilité qui m’est donnée d’interrompre le traitement à tout moment sans avoir à justifier ma décision mais je ferai mon possible pour informer le Dr . . . . . . . . . . . . . . . Cela ne remettra pas en cause la qualité des soins ultérieurs.

J’ai eu l’assurance que les décisions qui s’imposent pour ma santé seront prises à tout moment, conformément à l’état de connaissances sur la sclérose en plaques et à l’évolution de ma propre maladie.

J’accepte que les données enregistrées à l’occasion de ce protocole puissent faire l’objet d’un traitement informatisé. J’ai bien noté que le droit d’accès prévu par la loi “Informatique et Libertés” (article 39,40 et 56 du 6 Août 2004) s’exerce à tout moment auprès du Dr . . . . . . . . . . . . . . . . . . et que je pourrai exercer mon droit de rectification et d’opposition auprès de celui -ci.

J’accepte que seuls les médecins ou organisateurs impliqués dans le déroulement de cet essai, ainsi que les représentants des Autorités de Santé aient accès à l’information me concernant dans le respect le plus strict de la confidentialité.

Mon consentement ne décharge en rien les organisateurs de l’étude de leurs responsabilités. Je conserve tous les droits garantis par la loi.

A l'issue de cette étude, vous serez tenu personnellement informé des résultats globaux par votre médecin dès qu'ils seront disponibles.

*Le promoteur de l’étude (CHU de Bordeaux, 12 rue Dubernat, 33400 Talence) a souscrit une assurance de responsabilité civile en cas de préjudice auprès de la société Gerling France (n° 01/8088558) conformément aux dispositions de l’article L1127-07 du Code de la Santé Publique du 20 décembre 1988 et à l’article 5 de la loi du 25 juillet 1991*.

J'accepte de participer librement à cette recherche dans les conditions précisées dans le document d’information.

Fait à ..........................................le ...........................................

Nom  et prénom du Patient :..................................................................................................................................

Signature du Patient

Je soussigné(e), Docteur . . . . . . . . . . . . . . . . . . . . . . . . ., certifie avoir communiqué toutes informations utiles concernant cette étude. Je m’engage à faire respecter les termes de cette note de consentement, conciliant le respect des droits et des libertés individuelles et les exigences d’un travail scientifique.

Nom du Médecin : ................................................................

*Adresse du Médecin...........................................................................................................................................*

*Numéro de téléphone du Médecin :……………………………………* Signature du Médecin :……………………………….

Fait à ............................................... le .............................................................

**GLOSSAIRE**

- **Interféron** : Substance fabriquée par les globules blancs ayant des propriétés antivirales et immunomodulatrices.
- **Médicament immunosuppresseur** : Qui supprime ou réduit les réactions immunologiques spécifiques de l’organisme, en bloquant le système immunitaire de cet organisme. Médicaments qui diminuent la réponse immunitaire de l'organisme.
- **Médicament immunomodulateur** : Qui module les réactions immunitaires en les inhibant ou en les stimulant. . Médicament qui agit en modifiant les signaux émis par les cellules de défense de l'organisme
- **Réactions Immunitaires** : Ensemble de moyens de défense de l’organisme contre les agressions extérieures.
- **Etude contrôlée** : Etude correspondant aux critères scientifiques de bonne expérimentation clinique, avec un groupe témoin, et l’attribution des traitements par tirage au sort.
- **Ionogramme sanguin** : Formule représentant les concentrations des différents ions ou minéraux (sodium, potassium, chlore...) contenus dans le sang.
- **Créatininémie** : Mesure de la créatinine dans le sang.
- **Transaminase** : enzyme du foie.
- **Examen cytobactériologique des urines** : consiste à observer au microscope une goutte d'urine afin de rechercher la présence de globules blancs (ou leucocytes), de globules rouges (ou hématies) et d'éventuels microbes.
- **Réactions anaphylactiques** : Réaction aiguë allergique due à l'exposition de l’organisme à une substance étrangère précédemment présentée
- **Médicament antiémétique** : Médicament employé pour prévenir ou soulager les nausées et les vomissements.
- **Aménorrhées** : C'est l'absence de règles
- **Azoospermie** : c’est l'absence de spermatozoïdes dans le sperme.
- **Diurèse** : C’est la quantité d'urine par jour.
- **Alopécie** : correspond à une chute de cheveux sur tout ou une partie du cuir chevelu.
- **Cardiomyopathie** : Maladie du muscle cardiaque.
- **Fibrose pulmonaire** : transformation fibreuse des poumons.
- **Désordres hydro électrolytiques** : Déséquilibre de l’eau et des électrolytes dans l’organisme.
- **Rétention hydrosodée** : Présence en excès, dans l’organisme, d’eau et de sodium.
- **Troubles endocriniens** : Troubles caractéristiques de l’atteinte des glandes endocrines (se dit d’une glande dont la sécrétion est directement déversée dans le sang).
- **Ostéoporose** : Décalcification des os responsable d’une fragilité osseuse et, par suite, une augmentation du risque de fracture.
- **Intradermo-réaction (IDR) à la tuberculine** : Injection intradermique (entre l'épiderme et le derme) de tuberculine, contre laquelle l'organisme réagit en formant un bouton. La formation d'un bouton sur la peau dans les jours qui suivent l'intradermoréaction indique que l'organisme a été en contact avec (tuberculose ancienne ou en cours), ou que la vaccination par le BCG a été efficace et que l'organisme est donc immunisé contre la tuberculose.

**ANNEXE 5 : PHARMACIE**

##### PROTOCOLE CPM (Endoxan®) DANS LA S.E P : SCHEMA D’ADMINISTRATION PROPOSE

| **Horaire** | **BRAS CPM**  **(Endoxan®)** | **BRAS CORTICOIDE (Methylprednisolone)** |
| --- | --- | --- |
| T = 0  T = 0  **T = 30 min**  **T = 3heures**  T=3h30 min  **T = 8heures** | **Début hydratation :**  . **Acte infirmier** : mise en place poche G5 1 l  . **Produit fourni par la Pharmacie :**  poche gluc 5% 1 litre + 3 mg odansetron  (Zophren) +/- ( ½ dose)/  **IV lente sétron :**  **Acte infirmier :** IV lente dans tubulure  . **Produit fourni par la Pharmacie :**  seringue 10 ml : 8 mg odansetron (Zophren®) dans G5%.  Début perfusion CPM  **. Acte infirmier :** branchement poche en Y  **. Produit fourni par la Pharmacie :**  poche de glucosé 5 % 500 ml + CPM  (Endoxan®) dose totale  Changement perfusion hydratation  **Acte infirmier** : mise en place poche G5 1 l  . **Produit fourni par la Pharmacie :**  poche gluc 5% 1 litre + 3 mg odansetron  (Zophren®)    Arrêt perfusion CPM  **. Acte infirmier :** débranchement poche en Y  **Arrêt Protocole administration** | **Début hydratation :**  . **Acte infirmier** : mise en place poche G5 1 l  . **Produit fourni par la Pharmacie :**  poche gluc 5% 1 litre  **IV lente placébo:**  **Acte infirmier :** IV lente dans tubulure  . **Produit fourni par la Pharmacie :**  seringue 10 ml de G 5 %    Début perfusion Methylprednisolmone (MP)  **. Acte infirmier :** branchement poche en Y  **. Produit fourni par la Pharmacie :**  poche de glucosé 5 % 500 ml + 1 g  méthylprednisolone  Changement perfusion hydratation  **Acte infirmier** : mise en place poche G5 1 l  . **Produit fourni par la Pharmacie :**  poche gluc 5% 1 litre  Arrêt perfusion MP  **. Acte infirmier :** débranchement poche en Y  **Arrêt Protocole administration** |

**REMARQUE : Quelque soit le bras d’inclusion du patient :**

- **les produits fournis par la pharmacie seront présentés de manière rigoureusement identique**
- **ils seront identifiés par un étiquetage identique comportant : Code patient, service, N° de la cure, date de dispensation, date limite d’utilisation, conditions de conservation.**

#### EVALUATION DU COUT PHARMACEUTIQUE PAR CURE : PROTOCOLE CPM (Endoxan®) DANS LA S.E P :

**A/ BRAS CPM (ENDOXAN®)** Coût évalué sur la base des prix marchés CHU de Bordeaux en utilisant le schéma d’administration proposé précédent.

| **Préparation** | **Présentation** | **Coût unitaire** | **Nombre unités** | **Coût total** |
| --- | --- | --- | --- | --- |
| **1/ Perfusion glucosé 5 % hydrat + Odansetron (Zophren**®**) 8 mg**  **2/ CPM (Endoxan®) sur 3 heures (Pos moy 1400 mg)**  **3/ Changement perfusion hydratation**  **4/ Odansetron (Zophren**®**) 8 mg**  **5/ Odansetron (Zophren**®**) 8 mg**  **6/ Opération pharmaceutique** | - Poche souple Gluc 5 % 1 litre  - Zophren® 8 mg (4 ml)  **-** 1 seringue de 10 ml  - Poche souple Gluc 5 % 500 ml  - CPM (Endoxan®) 1000 mg  - CPM (Endoxan®) 500 mg  - Poche souple Gluc 5 % 1 litre  - Zophren® 8 mg (4 ml)  **-** 1 seringue de 10 ml  - Zophren® 8 mg (4 ml)  **-** 1 seringue de 10 ml  Temps évalué : 20 minutes  (facturé selon TAREX) | 0.53 €  2.335 €  0.090 €  0.41 €  10.89 €  5.31 €  0.53 €  2.335 €  0.090 €  2.335 €  0.090 €  6.45 € | 1  1  1  1  1  1  1  1  1  1  1  1 | **2.96 €**  **16.61 €**  **0.53 €**  **2.43** **€**  **2.43 €**  **6.45 €** |
| **Total** |  |  |  | **31.395 €** |

**REMARQUE** : Calcul basé sur une posologie moyenne de 1400 mg de CPM

**ANNEXE 9 : DECLARATION D'HELSINKI**

# DECLARATION D'HELSINKI DE L'ASSOCIATION MEDICALE MONDIALE

# Principes éthiques applicables aux recherches médicales sur des sujets humains

Adoptée par la 18e Assemblée générale, Helsinki, Juin 1964 et amendée par les

29e Assemblée générale, Tokyo, Octobre 1975

35e Assemblée générale, Venise, Octobre 1983

41e Assemblée générale, Hong Kong, Septembre 1989

48e Assemblée générale, Somerset West (Afrique du Sud), Octobre 1996

52e Assemblée générale, Edimbourg, Octobre 2000

l'Assemblée générale de l'AMM, Washington 2002 (addition d'une note explicative concernant le paragraphe 29) et

l'Assemblée générale de l'AMM, **Tokyo 2004 (**addition d'une note explicative concernant le paragraphe 30).

### INTRODUCTION

1. La Déclaration d'Helsinki, élaborée par l'Association médicale mondiale, constitue une déclaration de principes éthiques dont l'objectif est de fournir des recommandations aux médecins et autres participants à la recherche médicale sur des êtres humains. Celle-ci comprend également les études réalisées sur des données à caractère personnel ou des échantillons biologiques non anonymes.

2. La mission du médecin est de promouvoir et de préserver la santé de l'être humain. Il exerce ce devoir dans la plénitude de son savoir et de sa conscience.

3. Le Serment de Genève de l'Association médicale mondiale lie le médecin dans les termes suivants : "La santé de mon patient sera mon premier souci" et le Code international d'éthique médicale énonce que "le médecin devra agir uniquement dans l'intérêt de son patient lorsqu'il lui procure des soins qui peuvent avoir pour conséquence un affaiblissement de sa condition physique ou mentale".

4. Les progrès de la médecine sont fondés sur des recherches qui, in fine, peuvent imposer de recourir à l'expérimentation humaine.

5. Dans la recherche médicale sur les sujets humains, les intérêts de la science et de la société ne doivent jamais prévaloir sur le bien-être du sujet.

6. L'objectif essentiel de la recherche médicale sur des sujets humains doit être l'amélioration des méthodes diagnostiques, thérapeutiques et de prévention, ainsi que la compréhension des causes et des mécanismes des maladies. Les méthodes diagnostiques, thérapeutiques et de prévention, même les plus éprouvées, doivent constamment être remises en question par des recherches portant sur leur efficacité, leur efficience et leur accessibilité.

7. Dans la recherche médicale comme dans la pratique médicale courante, la mise en œuvre de la plupart des méthodes diagnostiques, thérapeutiques et de prévention expose à des risques et à des contraintes.

8. La recherche médicale est soumise à des normes éthiques qui visent à garantir le respect de tous les êtres humains et la protection de leur santé et de leurs droits. Certaines catégories de sujets sont plus vulnérables que d'autres et appellent une protection adaptée. Les besoins spécifiques des sujets défavorisés au plan économique comme au plan médical doivent être identifiés. Une attention particulière doit être portée aux personnes qui ne sont pas en mesure de donner ou de refuser elles-mêmes leur consentement, à celles qui sont susceptibles de donner leur consentement sous la contrainte, à celles qui ne bénéficieront pas personnellement de la recherche et à celles pour lesquelles la recherche est conduite au cours d'un traitement.

9. L'investigateur doit être attentif aux dispositions éthiques, légales et réglementaires applicables à la recherche sur les sujets humains dans son propre pays ainsi qu'aux règles internationales applicables. Aucune disposition nationale d'ordre éthique, légal et réglementaire ne doit conduire à affaiblir ou supprimer les mesures protectrices énoncées dans la présente déclaration.

**B. PRINCIPES FONDAMENTAUX APPLICABLES A TOUTE FORME DE**

**RECHERCHE MEDICALE**

10. Dans la recherche médicale, le devoir du médecin est de protéger la vie, la santé, la dignité et l'intimité de la personne.

11. La recherche médicale sur des êtres humains doit se conformer aux principes scientifiques généralement reconnus. Elle doit se fonder sur une connaissance approfondie de la littérature scientifique et des autres sources pertinentes d'information ainsi que sur une expérimentation appropriée réalisée en laboratoire et, le cas échéant, sur l'animal.

12. Des précautions particulières doivent entourer les recherches pouvant porter atteinte à l'environnement et le bien-être des animaux utilisés au cours des recherches doit être préservé.

13. La conception et l'exécution de chaque phase de l'expérimentation sur des sujets humains doivent être clairement définies dans un protocole expérimental. Ce protocole doit être soumis pour examen, commentaires, avis et, le cas échéant, pour approbation, à un comité d'éthique mis en place à cet effet. Ce comité doit être indépendant du promoteur, de l'investigateur ou de toute autre forme d'influence indue. Il doit respecter les lois et règlements en vigueur dans le pays où s'effectuent les recherches. Il a le droit de suivre le déroulement des études en cours. L'investigateur a l'obligation de fournir au comité des informations sur le déroulement de l'étude portant en particulier sur la survenue d'événements indésirables d'une certaine gravité. L'investigateur doit également communiquer au comité, pour examen, les informations relatives au financement, aux promoteurs, à toute appartenance à une ou des institutions, aux éventuels conflits d'intérêt ainsi qu'aux moyens d'inciter des personnes à participer à une recherche.

14. Le protocole de la recherche doit contenir une déclaration sur les implications éthiques de cette recherche. Il doit préciser que les principes énoncés dans la présente déclaration sont respectés.

15. Les études sur l'être humain doivent être conduites par des personnes scientifiquement qualifiées et sous le contrôle d'un médecin compétent. La responsabilité à l'égard d'un sujet inclus dans une recherche doit toujours incomber à une personne médicalement qualifiée et non au sujet, même consentant.

16. Toute étude doit être précédée d'une évaluation soigneuse du rapport entre d'une part, les risques et les contraintes et d'autre part, les avantages prévisibles pour le sujet ou d'autres personnes. Cela n'empêche pas la participation à des recherches médicales de volontaires sains. Le plan de toutes les études doit être accessible.

17. Un médecin ne doit entreprendre une étude que s'il estime que les risques sont correctement évalués et qu'ils peuvent être contrôlés de manière satisfaisante. Il doit être mis un terme à la recherche si les risques se révèlent l'emporter sur les bénéfices escomptés ou si des preuves consistantes de résultats positifs et bénéfiques sont apportées.

18. Une étude ne peut être réalisée que si l'importance de l'objectif recherché prévaut sur les contraintes et les risques encourus par le sujet. C'est particulièrement le cas lorsqu'il s'agit d'un volontaire sain.

19. Une recherche médicale sur des êtres humains n'est légitime que si les populations au sein desquelles elle est menée ont des chances réelles de bénéficier des résultats obtenus.

20. Les sujets se prêtant à des recherches médicales doivent être des volontaires informés des modalités de leur participation au projet de recherche.

21. Le droit du sujet à la protection de son intégrité doit toujours être respecté. Toutes précautions doivent être prises pour respecter la vie privée du sujet, la confidentialité des données le concernant et limiter les répercussions de l'étude sur son équilibre physique et psychologique.

22. Lors de toute étude, la personne se prêtant à la recherche doit être informée de manière appropriée des objectifs, méthodes, financement, conflits d'intérêts éventuels, appartenance de l'investigateur à une ou des institutions, bénéfices attendus ainsi que des risques potentiels de l'étude et des contraintes qui pourraient en résulter pour elle. Le sujet doit être informé qu'il a la faculté de ne pas participer à l'étude et qu'il est libre de revenir à tout moment sur son consentement sans crainte de préjudice. Après s'être assuré de la bonne compréhension par le sujet de l'information donnée, le médecin doit obtenir son consentement libre et éclairé, de préférence par écrit. Lorsque le consentement ne peut être obtenu sous forme écrite, la procédure de recueil doit être formellement explicitée et reposer sur l'intervention de témoins.

23. Lorsqu'il sollicite le consentement éclairé d'une personne à un projet de recherche, l'investigateur doit être particulièrement prudent si le sujet se trouve vis-à-vis de lui dans une situation de dépendance ou est exposé à donner son consentement sous une forme de contrainte. Il est alors souhaitable que le consentement soit sollicité par un médecin bien informé de l'étude mais n'y prenant pas part et non concerné par la relation sujet-investigateur.

24. Lorsque le sujet pressenti est juridiquement incapable, physiquement ou mentalement hors d'état de donner son consentement ou lorsqu'il s'agit d'un sujet mineur, l'investigateur doit obtenir le consentement éclairé du représentant légal en conformité avec le droit en vigueur. Ces personnes ne peuvent être inclues dans une étude que si celle-ci est indispensable à l'amélioration de la santé de la population à laquelle elles appartiennent et ne peut être réalisée sur des personnes aptes à donner un consentement.

25. Lorsque le sujet, bien que juridiquement incapable (un mineur par exemple), est cependant en mesure d'exprimer son accord à la participation à l'étude, l'investigateur doit obtenir que cet accord accompagne celui du représentant légal.

26. La recherche sur des personnes dont il est impossible d'obtenir le consentement éclairé, même sous forme de procuration ou d'expression préalable d'un accord, ne doit être conduite que si l'état physique ou mental qui fait obstacle à l'obtention de ce consentement est une des caractéristiques requises des sujets à inclure dans l'étude. Les raisons spécifiques d'inclure des sujets dans une étude en dépit de leur incapacité à donner un consentement éclairé doivent être exposées dans le protocole qui sera soumis au comité pour examen et approbation. Le protocole doit également préciser que le consentement du sujet ou de son représentant légal à maintenir sa participation à l'étude doit être obtenu le plus rapidement possible.

27. Les auteurs et les éditeurs de publications scientifiques ont des obligations d'ordre éthique. Lors de la publication des résultats d'une étude, les investigateurs doivent veiller à l'exactitude des résultats. Les résultats négatifs aussi bien que les résultats positifs doivent être publiés ou rendus accessibles. Le financement, l'appartenance à une ou des institutions et les éventuels conflits d'intérêt doivent être exposés dans les publications. Le compte-rendu d'une étude non conforme aux principes énoncés dans cette déclaration ne doit pas être accepté pour publication.

**C. PRINCIPES APPLICABLES A LA RECHERCHE MEDICALE CONDUITE AU**

**COURS D'UN TRAITEMENT**

28. Le médecin ne peut mener une recherche médicale au cours d'un traitement que dans la mesure où cette recherche est justifiée par un possible intérêt diagnostique, thérapeutique ou de prévention. Quand la recherche est associée à des soins médicaux, les patients se prêtant à la recherche doivent bénéficier de règles supplémentaires de protection.

29. Les avantages, les risques, les contraintes et l'efficacité d'une nouvelle méthode doivent être évalués par comparaison avec les meilleures méthodes diagnostiques, thérapeutiques ou de prévention en usage. Cela n'exclut ni le recours au placebo ni l'absence d'intervention dans les études pour lesquelles il n'existe pas de méthode diagnostique, thérapeutique ou de prévention éprouvée.1

30. Tous les patients ayant participé à une étude doivent être assurés de bénéficier à son terme des moyens diagnostiques, thérapeutiques et de prévention dont l'étude aura montré la supériorité.2

31. Le médecin doit donner au patient une information complète sur les aspects des soins qui sont liés à des dispositions particulières du protocole de recherche. Le refus d'un patient de participer à une étude ne devra en aucun cas porter atteinte aux relations que le médecin entretient avec ce patient.

32. Lorsqu'au cours d'un traitement, les méthodes établies de prévention, de diagnostic ou de thérapeutique s'avèrent inexistantes ou insuffisamment efficaces, le médecin, avec le consentement éclairé du patient, doit pouvoir recourir à des méthodes non éprouvées ou nouvelles s'il juge que celles-ci offrent un espoir de sauver la vie, de rétablir la santé ou de soulager les souffrances du malade. Ces mesures doivent, dans toute la mesure du possible, faire l'objet d'une recherche destinée à évaluer leur sécurité et leur efficacité. Toute nouvelle information sera consignée et, le cas échéant, publiée. Les autres recommandations appropriées énoncées dans la présente déclaration s'appliquent.

1 **Note explicative concernant le paragraphe 29**

L'AMM note avec préoccupation que le paragraphe 29 de la Déclaration d'Helsinki (Octobre 2000) est l'objet d'interprétations diverses et de possibles malentendus. Elle réaffirme par ailleurs que les essais avec témoins sous placebo ne doivent être utilisés qu'avec de grandes précautions et, d'une façon générale, lorsqu'il n'existe pas de traitement éprouvé. Toutefois, même s'il existe un traitement éprouvé, les essais avec témoins sous placebo peuvent être éthiquement acceptables dans les conditions suivantes:

• lorsque, pour des raisons méthodologiques impérieuses et scientifiquement solides, il n'existe pas d'autres moyens qui permettent de déterminer l'efficacité ou l'innocuité d'une méthode prophylactique, diagnostique ou thérapeutique ; ou

• lorsqu'une méthode prophylactique, diagnostique ou thérapeutique est mise à l'essai pour une affection bénigne et que la participation à l'essai n'expose pas à des risques supplémentaires de dommages significatifs ou durables.

Toutes les dispositions énoncées dans la DoH doivent être respectées, en particulier, la nécessité d'un examen éthique et scientifique approfondi.

2 **Note explicative concernant le paragraphe 30**

Par la présente, l'AMM réaffirme la nécessité d'identifier, lors de la planification d'une étude, l'accès post-étude pour les participants aux procédures prophylactiques, diagnostiques et thérapeutiques considérées comme bénéfiques par cette même étude ou un accès à d'autres soins appropriés. Les dispositions prises pour un tel accès ou pour d'autres soins doivent être décrites dans le protocole d'étude afin que le comité de révision éthique puisse étudier ces dispositions.

*La déclaration d'Helsinki (Document 17.C) est un document officiel de l'Association médicale mondiale, représentante des médecins dans le monde. Adoptée en 1964 à Helsinki (Finlande), elle fut révisée en 1975 à Tokyo (Japon), en 1983 à Venise (Italie), en 1989 à Hong Kong, en 1996 à Somerset West (Afrique du Sud), en 2000 à Edimbourg (Ecosse), par l'Assemblée générale de l'AMM, Washington 2002 (addition d'une note explicative concernant le paragraphe 29), et par l'Assemblée générale de l'AMM, Tokyo 2004 (addition d'une note explicative concernant le paragraphe 30).*

c/ SUMMARY OF CHANGES :

Twelve amendments have been submitted, and approved by the Ethic Committee (CPP), during the study between November 2005 (1^st^ amendment) and November 2009 (12^th^ amendment).

Changes in the name of clinical investigators and study personal have been submitted in amendments 1, 2, 3, 4, 6, 7, 8, 11 and 12.

Changes in procedures and organisation of the study without impact on the conduct of the study (for example change in committees, medication circuit etc...) have been submitted in amendments 1, 2, 3, 6 7, 9, 10 and11.

Significant changes in the protocol have been made in amendments 1, 2, 3, 4, 5, 10 and 12.

- Amendment 1 (November 2005) gave precision on the role of the prescribing physician.
- Amendment 2 (January 2006) indicated that a previous treatment with azathioprine in the month before randomisation is not allowed.
- Amendment 3 (April 2006) modified inclusion criteria: the maximum duration of the progressive phase before randomisation was change from 3 years to 4 years; Skin test for TB was recommended but no longer mandatory.
- Amendment 4 (April 2006) modified inclusion criteria: the maximum EDSS allowed at baseline was changed from 6.0 to 6.5. Inclusion of patients with only one exacerbation before the progressive stage (so-called “transitional progressive”) was allowed.
- Amendment 5 (October 2006) modified inclusion criteria: the maximum walking distance required at baseline was changed from 100 meters to 50 meters.
- Amendment 10 (February 2008) specified the conditions for unblinding after the end of the study.
- Amendment 12 (October 2009) added a few exploratory end-points (walking distance, PASAT correct answers, EDSS AUC).

2. Statistical analysis plan,
 a) Introduction and summary of changes
The original statistical analysis plan is the Statistical analysis section of the original protocol. (see above.)
In that version the Statistics Section is Section 12, p. 40 to 43.
The main changes in comparison with the final Analysis Plan were the introduction of two additional sensitivity analyses: one considering all patients who stopped their follow-up as having experienced deterioration of EDSS (worst case analysis for the comparison of proportions of deteriorated patients at 2 years), and the second one using multistate models to take into account the premature stopping of treatment and follow-up. This was decided after a blind data review (i.e. before unblinding) because of the high overall proportion of patients who stopped treatment and follow-up prematurely.
Minor changes included the possible use of a frailty model, instead of a standard Cox model, to better take into account the centre effect as a random effect, in case of significant variance of the center effect. As it was not the case the standard Cox model was used."

b) Final Analysis Plan

|  | USMR  Unité de Soutien Méthodologique en Recherche clinique et épidémiologique du CHU de Bordeaux | version | |
| --- | --- | --- | --- |
|  |  | *1.0* | *08/08/2013* |
| **Plan d’analyse statistique** | |  |  |

*PROMESS*

**Plan d’analyse statistique finale**

*Essai thérapeutique randomisé multicentrique en double insu en groupes parallèles du cyclophosphamide intra veineux versus methylprednisolone dans le traitement de fond des formes progressives et secondaires récentes de sclérose en plaques*

Investigateur coordonnateur : Pr. Bruno BROCHET

Méthodologiste : Dr. Paul Perez

Biostatisticien : Fabienne Marquant / Louise Baschet

Data manager : Catherine Maldonado

Case 75, 146 rue Léo-Saignat

33076 BORDEAUX Cedex

Tél : 33 (0)5 57 57 11 29, Fax : 33 (0)5 57 57 15 78

Email : [paul.perez@isped.u-bordeaux2.fr](mailto:antoine.benard@isped.u-bordeaux2.fr) **/** [louise.baschet@isped.u-bordeaux2.fr](mailto:louise.baschet@isped.u-bordeaux2.fr)

| **Tableau de signatures** | | | |
| --- | --- | --- | --- |
|  | Rédaction | Validation | Approbation |
| Nom |  |  |  |
| Fonction | Statisticien | Méthodologiste | Investigateur coordonnateur |
| Date  **Signature** |  |  |  |

| **Tableau des évolutions** | | |
| --- | --- | --- |
| **Version** | | **Motifications** |
| 0.1 | 29/04/2011 | Création par Fabienne Marquant |
| 0.2 | 17/05/2011 | Ajout de l’étape de revue des données, pas d’évaluation de l’insu car non recueillie |
| 0.3 | 15/06/2011 | Acceptation des précédentes modifications |
| 0.4 | 18/04/2012 | Précisions sur la revue des données, et précisions et questions sur les calculs des critères |
| 0.5 | 16/05/2012 | Quelques réponses sur les calculs des critères |
| 0.6 | 18/03/2013 | Modification Louise : mise au format qualité, simplification et compléments |
| 0.7 | 24/07/2013 | Intégration des conclusions de la revue des données du 14/05/2013 |
| 0.8 | 07/08/2013 | Réponses données en point le 30/07/2013 |
| 1.0 | 08/08/2013 | Version finale |

# Abréviations

| **9HPT** | nine hole peg test |
| --- | --- |
| **ARC** | Attaché de Recherche Clinique |
| **ASAT** | Aspartate amino transférase |
| **CPM** | cyclophosphamide |
| **d.m.** | données manquantes |
| **EDSS** | Expanded Disability Status Scale |
| **EI/EIG** | évenement indésirable / événement indésirable grave |
| **FSS** | score fonctionnel |
| **IC** | Intervalle de Confiance |
| **IMC** | Indice de Masse Corporelle |
| **ITT** | Intention to treat |
| **IV** | Intra-veineuse |
| **LOCF** | Last observation carried forward |
| **MP** | méthylprednisolone |
| **MSFC** | Multiple Sclerosis Functional Composite |
| **MSIS-29** | Multiple Sclerosis Impact Scale |
| **MSWS-12** | Multiple Sclerosis Walking Scale 12 items |
| **NT/NE** | neurologue traitant / neurologue évaluateur |
| **PASAT** | Paced Auditory Serial Addition Test |
| **Q1 / Q3** | 1^er^ quartile / 3^ème^ quartile |
| **S0…S92** | Semaine 0 … semaine 92 |
| **SC** | surface corporelle |
| **SEP** | Sclérose en plaque |
| **SP** | formes secondairement progressives |
| **TM** | temps de marche |
| **USMR** | Unité de soutien méthodologique en recherche clinique et épidémiologique du CHU |
| **V0…V20** | Visite 0 … visite 20 |

# Objet

Le présent document décrit les étapes et les méthodes d'analyse statistique à mettre en œuvre pour répondre à une question de recherche. Il est rédigé par le statisticien à partir du plan d'analyse statistique type, puis validé par le méthodologiste et approuvé par l'investigateur en charge de l'étude ou de la sous étude, avant le début de l'analyse, et donc avant le gel de la base.

Avant la validation définitive du plan d’analyse statistique, une revue des données est effectuée. Cette revue est faite en insu des groupes de traitement pour les données individuelles, et de façon groupée pour les données synthétiques. Les points suivants sont abordés avec l’équipe investigatrice :

- les violations au protocole « mineures » et « majeures », et les populations d’étude (ITT) seront définies
- le nombre de données manquantes pour les critères de jugement principal et secondaires sera décrit, et la stratégie de prise en compte des données manquantes validée,
- d’éventuelles incohérences non détectables par les contrôles informatiques pourront être relevées à partir des résultats de statistique descriptive présentés,
- des cas particuliers pour lesquels des questions se posent au moment de la création des variables d’intérêt seront discutés
- le critère de jugement principal sera validé patient par patient

Un plan de revue des données détaillé décrit le contenu du rapport de revue des données, et fait lui aussi l’objet d’une validation par le méthodologiste et d’une approbation par l’investigateur.

La levée d’insu ne sera effectuée qu’après cette revue des données et la validation définitive du plan d’analyse statistique, dans lequel seront détaillées les décisions prises pour les cas particuliers.

Toute modification majeure du plan d’analyse statistique ou ajout d’analyses post-gel de base sera tracée dans la partie 4. Analyses complémentaires (post gel de base), et fera l’objet d’une évolution du présent document avec validation et approbation.

**SOMMAIRE**

Abréviations	194

Objet	195

1.	Résumé de l’étude	198

1.1.	Rappel des objectifs	198

1.1.1.	Objectif principal	198

1.1.2.	Objectifs secondaires	198

1.2.	Schéma d’étude	198

1.3.	Randomisation	198

1.4.	Critères d’éligibilité	198

1.4.1.	Critères d’inclusion	198

1.4.2.	Critères de non inclusion	199

1.5.	Critères de jugement	200

1.5.1.	Critère de jugement principal	200

1.5.2.	Critères de jugement secondaires	201

1.5.3.	Critères de jugement exploratoires	201

1.5.4.	Critères exploratoires additionnels	201

2.	Aspects statistiques	202

2.1.	Stratégie d’analyse : population de l’étude	202

2.2.	Méthodes statistiques descriptives	202

2.3.	Méthodes statistiques comparatives	202

2.4.	Convention de calcul et gestion des données manquantes dans les analyses	203

2.4.1.	Délai entre 2 dates (incluant l’âge du patient)	203

2.4.2.	IMC	203

2.4.3.	Classe des traitements de fond	203

2.4.4.	Score EDSS	203

2.4.5.	Délai d’aggravation du score EDSS confirmé à 4 mois	205

2.4.6.	Existence de poussées dans les 12 mois précédant la pré-inclusion	206

2.4.7.	Durée de la maladie à la date d’initiation du traitement	206

2.4.8.	Proportion de patients ayant progressé de 0,5 point ou 1 point de l’échelle EDSS à la visite de fin de la période programmée de traitement (V19), et à la visite de 1 an (V13)	206

2.4.9.	Proportion de patients ayant une aggravation confirmée de l’échelle EDSS au cours du suivi	207

2.4.10.	Score composite de la SEP (Multiple Sclerosis composite score : MSFC) (Cutter, 1999), et z-score du 9 HPT, z-score du TM et z-score du PASAT 3s à V19	207

2.4.11.	Score du périmètre de marche	208

2.4.12.	Aggravation du périmètre de marche à la visite de fin de la période programmée de traitement (V19), et à la visite de 1 an (V13)	208

2.4.13.	Nombre de poussées pendant l’étude	209

2.4.14.	Tolérance au traitement	209

2.4.15.	SEP-59 : Score de qualité de vie	210

2.5.	Logiciel utilisés	210

2.6.	Risque de première espèce	210

3.	Plan d’analyse	211

3.1.	Description des inclusions et du suivi	211

3.1.1.	Bilan de la base	211

3.1.2.	Bilan des inclusions	211

3.1.3.	Respect du suivi prévu dans le protocole	211

3.1.4.	Description du traitement	211

3.1.5.	Diagramme de flux	211

3.2.	Description des patients à la visite de sélection et à l’inclusion	213

3.3.	Description des patients au cours du suivi	214

3.4.	Analyse des critères de jugement	214

3.4.1.	Critère de jugement principal	214

3.4.1.1.	Analyse principale	214

3.4.1.2.	Analyses de sensibilité	214

3.4.2.	Critères de jugement secondaires	215

3.4.3.	Critères d’évaluation exploratoires	215

3.4.4.	Critères exploratoires additionnels	216

# 1. Résumé de l’étude

## 1.1. Rappel des objectifs

1.1.1. Objectif principal

L’objectif principal est de comparer l’efficacité du traitement par CPM par rapport à un traitement par méthylprednisolone (MP) administrés par voie Intra-Veineuse (IV) pour empêcher l’aggravation confirmée sur 4 mois de 1 point d’EDSS (ou de 0,5 point si l’EDSS de départ est égale à 5, 5,5, 6 ou 6,5) à deux ans. L’étude concerne deux groupes randomisés parallèles. Le premier groupe devait recevoir chaque mois pendant un an puis tous les deux mois pendant un an une dose de 750mg/m² de surface corporelle (SC) de CPM par voie intraveineuse (IV). Le second groupe devait recevoir chaque mois pendant un an puis tous les deux mois pendant un an une dose de 1g de MP par voie IV.

1.1.2. Objectifs secondaires

Les objectifs secondaires sont de comparer dans les deux groupes de randomisation :

- la proportion de patients ayant progressé de 0,5 point ou 1 point de l’échelle EDSS (1 si l’EDSS initial est 4 ou 4,5 ou 0,5 point si l’EDSS initial est 5, 5,5, 6 ou 6.5) à 2 ans,
- le score composite MSFC et les 3 z scores le composant,
- le nombre de poussées,
- l’aggravation du périmètre de marche entre S0 et S92,
- la tolérance aux traitements,

## 1.2. Schéma d’étude

Il s’agit d’un essai clinique randomisé multicentrique national de phase IIIB en double insu sur deux groupes parallèles. La durée de l’essai est de deux ans par patient.

## 1.3. Randomisation

La liste de randomisation est établie par le statisticien de l’Unité de soutien méthodologique du CHU de Bordeaux (USMR) avant le début de l’essai. **La randomisation est stratifiée sur le centre**. Il n’est pas prévu de stratification sur les facteurs pronostiques éventuels, comme l’existence de poussées dans l’année précédant la randomisation ou la présence de lésions prenant le gadolinium. Les effectifs des deux groupes de traitement sont équilibrés. Un document complet décrivant la procédure de randomisation est conservé de manière confidentielle à l’USMR.

## 1.4. Critères d’éligibilité

1.4.1. Critères d’inclusion

Tous les critères suivants doivent être remplis par les patients pour être éligibles pour participer à l’étude :

- Homme ou Femme de 18 à 65 ans inclus
- Sujet affilié ou bénéficiaire d’un régime de sécurité sociale
- Présentant une forme secondairement progressive récente de SEP
- SEP certaine selon les critères de Mc Donald et al. (2001)
  - Forme secondairement progressive de SEP selon la définition de Lublin et Reingold (1996).
  - Les patients doivent avoir présenté une phase d’aggravation progressive du handicap d’au moins 6 mois et de moins de 4 ans
- L’aggravation du handicap récente doit être responsable d’une aggravation du score EDSS d’au moins 0,5 point dans les 12 derniers mois non attribuable à des séquelles de poussées
- Les patients doivent avoir présenté une réduction du périmètre de marche dans l’année écoulée
- EDSS entre 4,0 et 6,5 inclus
- Les patients doivent :
  - signé un consentement éclairé après information et avant toute procédure nécessitée par le protocole
  - accepter de recourir à une contraception efficace durant toute la durée de l’étude s’ils sont en âge de procréer (préservatif ou contraception efficace du patient ou du partenaire).
    1.4.2. Critères de non inclusion

Les patients ne peuvent être recrutés pour l’étude s’ils remplissent l’un des critères suivants :

- Personne placée sous sauvegarde de justice (article L11222 du code de la santé Publique).
- Autres formes cliniques de SEP :
  - Phase SP ayant débuté depuis plus de 4 ans
  - Forme rémittente pure de SEP sans progression entre les poussées
  - Forme progressive primaire de SEP
- Pathologies associées :
  - Présence de toute autre maladie autre que la SEP pouvant contribuer aux symptômes et signes neurologiques ou en affecter l’évaluation
  - Maladie organique ou psychiatrique compromettant la capacité du patient à comprendre les informations données et à donner ou retirer son consentement en toute connaissance des informations nécessaires et sa capacité à suivre le protocole
- Abus d’alcool ou de stupéfiants actuel ou dans les 2 ans précédents
- Contre-indication aux traitements à l’essai :
  - Cystite hémorragique préexistante.
  - Allergie connue au cyclophosphamide, aux corticoïdes et en particulier au Solumédrol ®
  - Maladie infectieuse en cours
  - Patients porteurs d’une cathétérisation permanente de la vessie (sondage urinaire à demeure)
  - Grossesse et allaitement.
  - Insuffisance médullaire sévère (Hb <9g/dl  ou Leucocytes <3000/mm3  ou Lymphocytes < 900 /mm3  ou Plaquettes 125 000/mm3). Ce critère devra être réévalué après le consentement, pendant la période de screening au vu du bilan biologique
  - Etat psychotique
  - Ulcère gastrique ou duodénal en évolution
  - Diverticulose colique
  - Antécédents de troubles du rythme cardiaque après injection de MP IV
  - Atteinte hépatique sévère (ASAT > 3X limite supérieure de la normale) ou rénale sévère (Créatinine > 180 µmol/L)
  - Diabète
- Participation en cours ou dans les 3 mois précédents à un autre essai thérapeutique
- Traitement antérieur par CPM, irradiation totale lymphoïde, anticorps monoclonal antiCD4 ou antiCD52 ou antiVLA 4, cladribine ou cyclosporine A.
- Traitement antérieur par mitoxantrone sauf si celui-ci a été effectué durant la phase rémittente et plus de 3 ans avant le début de l’étude. Dans ce cas-là, un bilan des Numérations Formule Sanguine et de l’échographie cardiaque transthoracique devra être fourni.
- En cas de traitement antérieur par interféron bêta, imurel ou méthotrexate un intervalle d’au moins un mois doit être respecté entre l’arrêt de ce traitement et le début du traitement à l’étude. Pour les statines voir chapitre 6.
- En cas de traitement antérieur par corticoïdes intraveineux mensuels, ce traitement doit avoir été arrêté depuis au moins 1 an.
- Arrêt du traitement aux corticoïdes de 3 à 5 jours inférieur à 2 mois.

## 1.5. Critères de jugement

1.5.1. Critère de jugement principal

Le critère de jugement principal est le délai d’aggravation du score EDSS (Kurtzke, 1983). Cette aggravation est définie par une augmentation de 0,5 point de l’échelle EDSS si l’EDSS initial est 5, 5,5, 6 ou 6,5 ou 1 point si l’EDSS initial est 4,0 ou 4,5 et à condition que cette aggravation soit confirmée lors des évaluations effectuées quatre mois après la première visite montrant cette aggravation. Si l’aggravation est constatée lors de la dernière visite de la période de traitement, la confirmation à deux mois, lors de la visite de fin d’étude, sera suffisante. Le délai d’aggravation sera calculé comme la différence entre la date de la première visite avec cette aggravation et la date d’initiation du traitement.

1.5.2. Critères de jugement secondaires
- Proportion de patients ayant progressé de 0,5 point ou 1 point de l’échelle EDSS (1 si l’EDSS initial est 4 ou 4,5 ou 0,5 point si l’EDSS initial est 5, 5,5, 6 ou 6,5) à 2 ans (visite de fin de la période programmée de traitement).
- Score composite de la SEP (Multiple Sclerosis composite score : MSFC) à deux ans (Cutter, 1999).
- Z scores du 9 HPT, z score du TM et z score du PASAT 3s à deux ans.
- Nombre de poussées pendant l’étude.
- Tolérance au traitement : proportion et délai de survenue des événements indésirables graves et non graves, évalués à la fin de l'étude.
  1.5.3. Critères de jugement exploratoires

NB : Ces critères ne seront pas considérés comme des critères d’efficacité.

- Auto questionnaire de qualité de vie (échelle SEP 59, Vernay et al., 2000) à S0 (Visite 2), S44 (Visite 13), et S92 (Visite 19) permettant d’établir 15 scores correspondants aux axes de l’échelle.
- Auto questionnaire de mesure du retentissement de la SEP (MSIS-29) et auto questionnaire d’évaluation de la marche (MSWS-12) à S0 (Visite 2), S44 (Visite 13), et S92 (Visite 19)
- Tous les critères de jugement secondaires recueillis aux visites 13 (semaine 44) et 19 (semaine 92).
- Taux de lymphocytes et numération des éosinophiles des bilans biologiques comme critère d’action du cyclophosphamide (Endoxan®) (Smith DR et al, 2005).
  - taux de lymphocytes
  - nombre d’adaptations de traitement en fonction du taux de lymphocytes
  - nombre de lymphopénies graves
  - éosinophilie
    1.5.4. Critères exploratoires additionnels
- le nombre de réponses correctes consécutives maximum par test PASAT à S44,
- l’évolution du score de périmètre de marche entre S0 (Visite 2) et S44 (Visite 19)

# 2. Aspects statistiques

## 2.1. Stratégie d’analyse : population de l’étude

L’analyse sera réalisée **en intention de traiter**, c’est à dire que tous les patients randomisés seront inclus dans l'analyse dans le groupe dans lequel ils ont été initialement randomisés, et toutes leurs données seront analysées quels que soient leurs changements ou arrêt de traitement au cours de l’étude.

Ne pourront être exclus de l’analyse que les patients qui présentent au moins une des conditions suivantes :

- patients n’ayant jamais pris le traitement de la recherche (à condition de ne pas avoir eu connaissance du groupe dans lequel ils ont été randomisés) ;
- patients inclus à tort pour consentement non signé ;
- patients ayant retiré leur consentement.

Cette décision d’exclusion sera prise par le Conseil Scientifique après documentation de l’observation par le Centre de Méthodologie et de Gestion des données, en insu du groupe de traitement et de l’évolution du patient après l’inclusion.

En dehors de ces exclusions, les patients décédés, perdus de vue ou ayant abandonné la recherche seront tous inclus dans l’analyse.

La gestion des données manquantes est définie pour chaque critère dans le §2.4.

## 2.2. Méthodes statistiques descriptives

La description se fait toujours globalement et par groupe de traitement.

Les variables qualitatives seront décrites en termes d’effectif, de pourcentage et éventuellement d’intervalle de confiance.

Les intervalles de confiance des proportions seront calculés en utilisant la loi binomiale.

Les variables quantitatives seront décrites en termes d’effectifs, de moyennes, écart-type, médiane, Q1, Q3, minimum, et maximum.

Les intervalles de confiance des variables quantitatives seront calculés en faisant l’hypothèse que la variable suit une distribution théorique Normale. En cas d’écart trop important par rapport à cette loi, des transformations appropriées de la variable quantitative seront effectuées.

## 2.3. Méthodes statistiques comparatives

Les comparaisons entre groupes de traitement se font systématiquement :

- en ajustant sur la variable de stratification (centre en effet aléatoire),
- en ajustant sur la variable initiale de stratification et d’autres caractéristiques initiales en tant que facteurs pronostiques. Ces ajustements nécessitent éventuellement d’utiliser des modèles appropriés dont la pertinence du choix est discutée en fonction de la distribution et du type de variables.

Les variables qualitatives seront comparées entre les groupes à l’aide des régressions logistiques à effets mixtes afin d’ajuster ces associations sur des facteurs pronostiques.

Les variables quantitatives seront comparées entre les groupes à l’aide de régressions linéaires à effets mixtes afin d’ajuster ces associations sur des facteurs pronostiques. Les conditions d’application de ces modèles seront vérifiées. Des transformations, entre autres une transformation en rang, seront envisagées en cas de distribution non gaussienne, et des régressions de poisson seront envisagées pour les variables de comptage.

## 2.4. Convention de calcul et gestion des données manquantes dans les analyses

2.4.1. Délai entre 2 dates (incluant l’âge du patient)

(date événement – date 0) en jours calculée avec la fonction DATDIF de SAS.

(date événement – date 0) en semaine calculée avec la fonction DATDIF de SAS, divisé par 7.

(date événement – date 0) en mois calculée avec la fonction DATDIF de SAS, divisé par 30.4375.

(date événement – date 0) en année calculée avec la fonction YRDIF de SAS.

Si le jour est manquant la convention est d’utiliser le 15 (milieu du mois). Si le jour et le mois sont manquants, la convention est d’utiliser la valeur 1 pour le jour et la valeur 7 pour le mois (milieu de l’année). Si seul le mois est manquant, la convention est d’utiliser le 6.

Si l’année au moins d’une date est manquante, le délai n’est pas calculé, la valeur manquante n’est pas remplacé (sauf indication contraire dans le plan d’analyse).

2.4.2. IMC

IMC = Poids (kg) / (Taille (m) x Taille (m))

2.4.3. Classe des traitements de fond

Les antécédents de traitement liés à la SEP sont définis en 3 classes :

- Immunomodulateurs : L04AX11 (Avonex), L03AB08 (Betaferon), L03AB07 (Rebif), L03AX13 (Copaxone)

- Immunosuppresseurs : L01BA01 (Methotrexate), L01DB07 (Mitoxantrone), L04AX01 (Imurel), L04AA06 (Cellcept)

- autres.

2.4.4. Score EDSS

Définition :

Le score est calculé selon l’algorithme qui suit à partir des sous-scores. Il a dû être calculé de cette manière par le centre coordonnateur, au cours de l’étude.

Dans les cas où le score n’est pas calculable, il arrive que le score ait tout de même été recueilli, au niveau du centre investigateur. Dans ce cas, il est impossible de vérifier le score et une variabilité inter-centres est possible.

Selon le protocole c’est le score calculé par le centre coordonnateur à partir de ses items qui doit être utilisé pour l’analyse.

Cependant, afin de minimiser le nombre de données manquantes, il est proposé que Le score recueilli soit exploité, le score calculé ne sert qu’à faciliter la vérification des cas complets.

0) *Distance de marche non renseignée*	EDSS non calculable
1) *Marche sans aide ni repos ≥ 700m :*

Si tous les scores sont manquants ou si un seul est renseigné, le score EDSS n’est pas calculé.

Sinon, les quelques scores manquants sont considérés comme nuls.

| **Score des FSS** | **EDSS** |
| --- | --- |
| Tous les scores = 0 | 0 |
| 1 à 6 scores = 1 ; les autres = 0 | 1 |
| Les 7 scores = 1 | 1.5 |
| 1 score = 2 ; les autres = 0 ou 1 | 2 |
| 2 scores = 2 ; les autres = 0 ou 1 | 2.5 |
| 3 à 4 scores = 2 ; les autres = 0 ou 1 | 3 |
| 5 à 6 scores = 2 ; les autres = 0 ou 1 | 3.5 |
| Les 7 scores = 2 | 3.5 |
| 1 score = 3 (ou FSV 4 ou 5) ; les autres = 0 ou 1 | 3 |
| 1 score = 3 (ou FSV 4 ou 5) ; 1 ou 2 scores = 2 ; les autres = 0 ou 1 | 3.5 |
| 1 score = 3 (ou FSV 4 ou 5) ; + de 2 scores = 2 | 4 |
| 2 scores = 3 (ou FSV 4 ou 5) ; les autres = 0 ou 1 | 3.5 |
| 2 scores = 3 (ou FSV 4 ou 5) ; 1 scores ou + = 2 | 4 |
| + de 2 scores = 3 (ou FSV 4 ou 5) ; mais tous < 4 (ou FSV 6) | 4 |
| 1 score = 4 (ou FSV 6) ; les autres = 0 ou 1 | 4 |
| 1 score = 4 (ou FSV 6) ; 1 score ou plus = 2 | 4.5 |
| 1 score = 4 (ou FSV 6) ; 1 score ou plus = 3 | 4.5 |
| 2 scores ou + = 4 (ou FSV 6) | 5 |
| Au moins un score > 4 | 5 |

2) *Marche limitée entre 500 inclus et 700m exclus :*	EDSS = 4
3) *Marche limitée entre 300 inclus et 500m exclus :*

Même algorithme que 1), si le score obtenu est 4, coter 4.5, si le score obtenu < 4, coter 4, si le score obtenu est 5, laisser 5

4) *Marche entre 200 inclus et 300m exclus :*	EDSS = 5
5) *Marche entre 100 inclus et 200m exclus :*	EDSS = 5.5
6) *Marche <100 :*

| **Distance avec aide unilatérale** | **Distance avec aide bilatérale** | **EDSS** |
| --- | --- | --- |
| ≥ 100m | N’importe, même manquant | 6 |
| N’importe, même manquant | >120 | 6 |
| < 20 | [20 ;120 ] | 6.5 |
| [20 ; 100[ | manquant | 6.5 |
| [20 ; 100[ | ≤ 100 | 6.5 |
| manquant | [20 ; 100] | 6.5 |
| <20 | <20 | 7 |
| <20 | manquant | 7 |
| manquant | < 20 | 7 |
| manquant | manquant | Non calculable |

Gestion des données manquantes :

Pour les sous-scores, la gestion des données manquantes est prévue dans l’algorithme.

2.4.5. Délai d’aggravation du score EDSS confirmé à 4 mois

Définition :

- Aggravation =
  - augmentation de 0,5 point de l’échelle EDSS si l’EDSS initial est 5, 5,5, 6 ou 6,5
  - ou 1 point si l’EDSS initial est 4,0 ou 4,5 ou < 4

EDSS initial= EDSS à l'inclusion si disponible, sinon EDSS initial = EDSS à la visite de sélection

EDSS = EDSS recueilli (cf. §2.4.2)

Gestion de la censure par intervalle :

Il peut s'écouler plus de 2 mois entre 2 visites, l'aggravation observée lors d'une visite a pu débuter n'importe quand entre cette visite et la précédente, plus de 2 mois plus tôt. Le délai d'aggravation est donc potentiellement surestimé si on prend comme début la date de la première visite avec score aggravé ("censure par intervalle").

Pour l’analyse principale : la date de début d’aggravation est le milieu de l'intervalle entre la dernière visite non aggravée et la première visite aggravée.

- Confirmation = aggravation confirmée lors des évaluations effectuées au cours des quatre mois après la première visite montrant cette aggravation. Si l’aggravation est constatée lors de la dernière visite de la période de traitement, la confirmation à deux mois (un délai de 8 semaines est nécessaire), lors de la visite de fin d’étude, sera suffisante.
  - On vérifie que le score reste aggravé lors de toutes les visites pendant les 4 mois qui suivent le début de l'aggravation.
  - Si la visite V(+2) a lieu 3,5 mois après le début de l’aggravation et non pas 4 mois après, on considère cette aggravation comme confirmée.
  - S’il y a un délai de plus de 6 mois entre 2 visites, toutes ces visites « tardives » ne sont pas utilisées, on considère que les visites n’ont pas eu lieu.
  - Si l’aggravation est constatée avant l’arrêt du traitement, lors de la dernière visite (autre que la V20), et qu’il n’y a aucune visite postérieure à cette évaluation pour sortie d’étude, on considère l’aggravation à cette dernière visite comme confirmée.
  - Si l'aggravation a lieu lors d'une visite post-arrêt, et qu’il n’y a aucune visite postérieure à cette évaluation pour sortie d’étude, l’aggravation n’est pas confirmée (les informations de ces visites ont été récupérées a posteriori).
  - Il peut arriver que l’aggravation soit confirmée à 4 mois, mais que le score EDSS redescende ensuite. Ceci ne modifie pas le critère.

Le délai d’aggravation sera calculé comme la différence entre la date de début d’aggravation (rappel : milieu entre la dernière visite non aggravée et la première visite aggravée) et la date d’initiation du traitement, en semaines

Gestion des suivis incomplets :

Pour l’analyse principale : les données des patients ayant arrêté prématurément le suivi avant observation d’une aggravation confirmée sont censurés, c’est-à-dire que l’aggravation n’a pas pu être observée et qu’on ne fait pas d’hypothèse sur ce qui a pu se produire suite à la sortie d’étude.

Des analyses de sensibilité permettront de confirmer/ infirmer les conclusions d’abord en considérant chaque sortie prématurée d’étude comme un échec de traitement et les patients seront considérés comme aggravés, puis par le modèle global multi-état (non aggravé, aggravation confirmée, sortie d’étude) : l’arrêt du suivi est un évènement concurrent absorbant (voir §3.4.1.2).

Gestion des données manquantes :

Si la date de la visite est manquante, elle n’est pas considérée.

Tous les cas sont prévus et aucune donnée ne sera manquante, par contre des délais pourront être censurés et l’analyse en tiendra compte.

2.4.6. Existence de poussées dans les 12 mois précédant la pré-inclusion

Définition :

si la variable POUSSEE_NB_ANNEE > 0 alors existence_poussees = oui,

si POUSSEE_NB_ANNEE = 0 alors existence_poussees = non

Gestion des données manquantes :

Aucune, les patients pour lesquels le nombre de poussées dans les 12 mois précédant la pré-inclusion ne pourront pas être analysés.

2.4.7. Durée de la maladie à la date d’initiation du traitement

Définition :

date d’initiation du traitement - date de la première poussée.

Gestion des données manquantes :

Si le jour du début de la maladie n’est pas recueilli, on remplace par 15.

Si le mois est manquant, on remplace par juin (6), à condition que ceci n’inverse pas l’ordre chronologique entre début maladie puis initiation traitement (auquel cas la donnée reste manquante et le patient n’est pas analysé).

Si l’année est manquante, la date est manquante, et donc le patient ne pourra pas être analysé.

2.4.8. Proportion de patients ayant progressé de 0,5 point ou 1 point de l’échelle EDSS à la visite de fin de la période programmée de traitement (V19), et à la visite de 1 an (V13)

Définition :

L’aggravation est définie par une progression de 1 si l’EDSS initial est inférieur ou égal à 4.5 ou 0,5 point si l’EDSS initial est supérieur ou égal à 5. Score initial = score à l’inclusion si possible, sinon score à la visite de sélection.

Ce critère est mesuré de façon transversale à la visite de fin de la période programmée de traitement (V19), et à la visite de 1 an (V13)

Gestion des données manquantes :

Pour les patients ayant réalisé la visite concernée, mais pour lesquels le score EDSS est manquant :

- on impute l’état à la visite suivante (V20 / V14), si disponible.

- Sinon, si le patient était aggravé à la visite précédente (V18 / V12), le patient est considéré comme aggravé.

- Sinon, le patient est considéré comme aggravé (missing = failure). (La méthode du biais maximum sera utilisée pour juger de la robustesse des conclusions à cette imputation sur ces patients.)

Pour les patients n’ayant pas effectué la visite concernée pour cause d’arrêt prématuré du traitement :

- le score à la visite à 2 ans+/- 3 mois (1 an +/- 2 mois) post-arrêt est considéré, si disponible.

- sinon, si l’arrêt du traitement est dû à des effets indésirables, on considère le patient en échec de stratégie, c’est-à-dire aggravation

- sinon, si le patient était aggravé aux dernières nouvelles avant la visite concernée, on considère le patient en aggravation

- sinon, on considère le patient en aggravation (missing = failure). (La méthode du biais maximum sera utilisée pour juger de la robustesse des conclusions à cette imputation sur ces patients).

Une seconde analyse sur données disponibles sera réalisée, afin de comparer les deux bras de traitement chez les patients continuant le traitement à la visite concernée.

2.4.9. Proportion de patients ayant une aggravation confirmée de l’échelle EDSS au cours du suivi

Définition :

L’aggravation confirmée est définie comme pour le délai d’aggravation confirmée.

- On vérifie que le score reste aggravé lors de toutes les visites pendant les 4 mois qui suivent le début de l'aggravation.
- Si la visite V(+2) a lieu 3,5 mois après le début de l’aggravation et non pas 4 mois après, on considère cette aggravation comme confirmée.
- S’il y a un délai de plus de 6 mois entre 2 visites, toutes ces visites « tardives » ne sont pas utilisées, on considère que les visites n’ont pas eu lieu.
- Si l’aggravation est constatée avant l’arrêt du traitement, lors de la dernière visite (autre que la V20), et qu’il n’y a aucune visite postérieure à cette évaluation pour sortie d’étude, on considère l’aggravation à cette dernière visite comme confirmée.
- Si l'aggravation a lieu lors d'une visite post-arrêt, et qu’il n’y a aucune visite postérieure à cette évaluation pour sortie d’étude, l’aggravation n’est pas confirmée (les informations de ces visites ont été récupérées a posteriori).
- Il peut arriver que l’aggravation soit confirmée à 4 mois, mais que le score EDSS redescende ensuite. Ceci ne modifie pas le critère.

Gestion des données manquantes :

Les données manquantes sont gérées dans l’algorithme. Pour les patients n’ayant pas complété le suivi, la durée du suivi est décrite conjointement, afin de juger de la comparabilité des groupes.

2.4.10. Score composite de la SEP (Multiple Sclerosis composite score : MSFC) (Cutter, 1999), et z-score du 9 HPT, z-score du TM et z-score du PASAT 3s à V19

Définition :

Le MSFC comprend la combinaison de 3 mesures, une mesure chronométrée d’habileté motrice du membre supérieur, le nine hole peg test (9HPT), une mesure de marche : temps de marche (T.M.) chronométré sur 8 m, et un test cognitif mesurant l’attention, le PASAT (version 3 secondes).

Les valeurs obtenues pour chacun de ces tests seront transformées en z-scores de la façon suivante :

Z_9HPT_	=	[moyenne(1/(gauche),1/(droit)) – 0.0439] / 0.0101

où	1/gauche = 1/(moyenne(9HPT test 1 bras gauche, 9HPT test 2 bras gauche)

1/droit	= 1/(moyenne(9HPT test 1 bras droit, 9HPT test 2 bras droit)

Z_TM_	=	(moyenne(TM test 1, TM test 2) – 9.5353) / 11.4058

Z_PASAT_	=	(nombre de réponses correctes au PASAT – 45.0311) / 12.0771

Le score MSFC est établi à partir des 3 z scores de ces 3 mesures selon la formule :

(Z_9HPT_ – Z TM + Z PASAT) / 3.

Pour la visite de sélection, pour laquelle deux tests devaient être effectués, le score MSFC sera calculé en utilisant les résultats du deuxième test.

Les résultats du premier test pourraient cependant être analysés, dans l’optique d’une comparaison des résultats des deux tests de la visite de sélection.

Gestion des données manquantes :

Si les résultats pour un bras sont manquants, mais les résultats du second bras sont disponibles, le Z_9HPT_ est calculé à partir des résultats du seul bras disponible.

De même, si un seul test du temps de marche est disponible, le Z_TM_ est calculé à partir des résultats de ce seul test.

Si un des éléments nécessaires au calcul est manquant, alors le score MSFC est manquant.

De même, pour tous les patients n’ayant pas réalisé leur visite 19, le score MSFC est manquant.

L’analyse principale sera réalisée sur données disponibles. Une analyse de sensibilité en LOCF sera réalisée.

2.4.11. Score du périmètre de marche

| **Score** | **Périmètre de Marche** |
| --- | --- |
| **1** | Aucune limitation ou ≥ 700 m, aucune aide |
| **2** | ≥ 500 m à < 700m, aucune aide |
| **3** | ≥ 300 m à < 500m, aucune aide |
| **4** | ≥ 200 m à < 300m, aucune aide |
| **5** | ≥ 100 m à < 200m, aucune aide |
| **6** | > 100 m quelle que soit l'aide |
| **7** | ≥ 25 m à = 100m quelle que soit l'aide |
| **8** | ≥ 5 m à < 25m quelle que soit l'aide |
| **9** | < 5m quelle que soit l'aide |

Gestion des données manquantes :

Aucune. Les analyses seront réalisées sur données disponibles.

2.4.12. Aggravation du périmètre de marche à la visite de fin de la période programmée de traitement (V19), et à la visite de 1 an (V13)

Définition :

Une aggravation est définie par une augmentation d’au moins un point du score du périmètre de marche (défini au § précédent) par rapport au score du périmètre de marche initial.

Score du périmètre initial = score à l’inclusion si possible, sinon score à la visite de sélection

Ce critère est mesuré de façon transversale lors de la visite de fin de la période programmée de traitement (V19), et à la visite de 1 an (V13)

Gestion des données manquantes :

Pour les patients ayant réalisé la visite concernée, mais pour lesquels le périmètre de marche est manquant :

- on impute l’état à la visite suivante (V20 / V14), si disponible.

- Sinon, si le patient était aggravé à la visite précédente (V18 / V12), le patient est considéré comme aggravé.

- Sinon, le patient est considéré comme aggravé (missing = failure). La méthode du biais maximum sera utilisée pour juger de la robustesse des conclusions à cette imputation à ces patients.

Pour les patients n’ayant pas effectué la visite concernée pour cause d’arrêt prématuré du traitement :

- le score à la visite à 2 ans+/- 3 mois (1 an +/- 2 mois) post-arrêt est considéré, si disponible.

- sinon, si l’arrêt du traitement est dû à des effets indésirables, on considère le patient en échec de stratégie, c’est-à-dire aggravation

- sinon, si le patient était aggravé aux dernières nouvelles, on considère le patient en aggravation

- sinon, on considère le patient en aggravation (missing=failure). La méthode du biais maximum sera utilisée pour juger de la robustesse des conclusions à cette imputation.

Une seconde analyse sur données disponibles sera réalisée, afin de comparer les deux bras de traitement chez les patients ayant continué le traitement.

2.4.13. Nombre de poussées pendant l’étude

Définition :

Le nombre de poussées sera déterminé par le nombre de fiches EI remplies pour des poussées entre S0 et S92. (quand EI_NOM_1 = '10048393' ou EI_NOM_1 ='10048393' ou DIAGN_NOM_1 ='10048393' ou DIAGN_2 = '10048393'))

Gestion des données manquantes : Aucune donnée manquante

2.4.14. Tolérance au traitement

Définition :

La tolérance au traitement sera déterminée par la fréquence et le délai de survenue des événements indésirables graves et non graves, évalués à la fin de l'étude.

Un patient est considéré intolérant au traitement s’il a au moins une fiche EI dont l’imputabilité au traitement n’est pas exclue.

Le délai d’intolérance est défini comme le délai entre le début du traitement et la date du premier EI dont l’imputabilité au traitement n’est pas exclue.

Un patient est considéré gravement intolérant au traitement s’il a au moins une fiche EIG dont l’imputabilité au traitement n’est pas exclue.

Le délai d’intolérance grave est défini comme le délai entre le début du traitement et la date du premier EIG dont l’imputabilité au traitement n’est pas exclue.

Gestion des données manquantes : Pas de donnée manquante.

2.4.15. SEP-59 : Score de qualité de vie

Définition :

Les scores moyens sont calculés pour chaque dimension, les réponses étant normées de 0 à 100, où 100 représente la meilleure qualité de vie possible. Le score moyen d'un axe se définit comme la somme des notes attribuées à chaque item divisée par le nombre d'items.

| Dimensions | Nombre d'items | Numéro des items | Nombre minimal d’items pour calculer le score |
| --- | --- | --- | --- |
| Activité physique | 10 | 3a - 3j | 6 |
| Limitations liées à la santé physique | 4 | 4a - 4d | 3 |
| Limitations liées à la santé mentale | 3 | 5a - 5c | 2 |
| Bien-être social | 3 | 6 ; 10 ; 18 | 2 |
| Douleur | 3 | 7 ; 8 ; 19 | 2 |
| Energie | 4 | 9a ; 9e ; 9g ; 9i | 3 |
| Bien-être émotionnel | 5 | 9b ; 9c ; 9d ; 9f ; 9h | 3 |
| Santé générale (Health perception) | 6 | 1 ; 11a ; 11b ; 11c ; 11d ; 22 | 4 |
| Détresse | 4 | 12a - 12d | 3 |
| Fonction cognitive | 4 | 14a - 14d | 3 |
| Fonction sexuelle | 4 | 15a - 15d | 3 |
| Satisfaction sexuelle | 1 | 16 | 1 |
| Bien-être général | 2 | 20, 21 | 2 |
| Sommeil | 2 | 13a, 13b | 2 |
| Support social | 4 | 17a - 17d | 3 |

Questions pour lesquelles la meilleure qualité de vie est représentée par la plus petite réponse : 1, 2, 6, 7, 8, 9a, 9d, 9e, 9h, 11b, 11d, 13b, 16, 17, 18, 19.

Questions pour lesquelles la meilleure qualité de vie est représentée par la plus grande réponse : 3, 4, 5, 9b, 9c, 9f, 9g, 9i, 10, 11a, 11c, 12, 13a, 14, 15, 20, 21.

Gestion des données manquantes :

Le nombre d’items minimal pour calculer le score moyen pour chaque dimension est indiqué dans le tableau. Si plus d’items ne sont pas renseigné, le score de cette dimension est manquant. L’analyse est réalisée sur données disponibles.

## 2.5. Logiciel utilisés

Les analyses statistiques seront réalisés avec le logiciel SAS (V9.2) et R (V2.14.1).

## 2.6. Risque de première espèce

Tous les tests statistiques seront effectués au risque d’erreur de première espèce α=5%.

# 3. Plan d’analyse

## 3.1. Description des inclusions et du suivi

3.1.1. Bilan de la base
- nombre de fiches saisies dans chaque table
- nombre de patients par table
  3.1.2. Bilan des inclusions
- Nombre de patients pré-inclus dans l’étude
  - dont non inclus et raison de non inclusion
- Nombre de patients inclus
  - dont inclus dans l’analyse avec violations mineures du protocole
  - dont exclus de l’analyse et raison d’exclusion
- Période d’inclusion + courbe des inclusions (inclusions attendues / réalisées)
- Respect des critères d’éligibilité
  3.1.3. Respect du suivi prévu dans le protocole

Nombre de patients ayant complété toutes leurs visites

Nombre de patients suivis sur les 2 ans (y compris les patients avec des visites manquantes)

Durée de suivi pour les patients suivis moins de 2 ans

Délai entre l’inclusion et chaque visite du neurologue évaluateur

Nombre de patients à chaque visite

Nombre de visites non programmées

3.1.4. Description du traitement

Effectifs de patients qui :

- ont reçu le traitement pendant 2 ans
- ont arrêté le traitement et raison d’arrêt prématuré

nombre de visites NT effectuées

nombre de visites non programmées effectuées

3.1.5. Diagramme de flux

Diagramme de flux selon les recommandations CONSORT :

**Alloués au traitement CPM (n= )**

**Pré-inclus (n= )**

Non inclus (n=	)

♦  non éligibles (n= )

♦  refus de participer (n= )

♦  autres raisons (n= )

**Inclus et randomisés (n= )**

**Recrutement**

**Alloués au traitement MP (n= )**

**Randomisation**

Ont un suivi de 2 ans (n= )

Dont ceux qui ont effectué les 20 visites (n= )

Ont arrêté le suivi (n= )

Dont ceux qui ont effectué la visite d’arrêt (n= )

Dont ceux qui ont effectué leur visite 8 semaines après l’arrêt du traitement (n= )

nombre de visites NE effectuées : *médiane, mi-max*

Analysés (n= )

Exclus des analyses (n= )

*Raisons d’exclusions* (n= )

Ont un suivi de 2 ans (n= )

Dont ceux qui ont effectué les 20 visites (n= )

Ont arrêté le suivi (n= )

Dont ceux qui ont effectué la visite d’arrêt (n= )

Dont ceux qui ont effectué leur visite 8 semaines après l’arrêt du traitement (n= )

nombre de visites NE effectuées : *médiane, mi-max*

ont reçu le traitement pendant 2 ans (n= )

ont arrêté le traitement (n= )

à l'initiative du clinicien pour EI (n=	)

à l'initiative du clinicien pour autre raison (n=	)

à l'initiative du patient pour EI (n=	)

à l'initiative du patient pour autre raison (n=	)

à l'initiative du patient pour raison non communiquée (n= )

nombre de visites NT effectuées : *médiane, min-max*

Exclus pour retrait de consentement (n=	)

**Suivi**

ont reçu le traitement pendant 2 ans (n= )

ont arrêté le traitement (n= )

à l'initiative du clinicien pour EI (n=	)

à l'initiative du clinicien pour autre raison (n=	)

à l'initiative du patient pour EI (n=	)

à l'initiative du patient pour autre raison (n=	)

à l'initiative du patient pour raison non communiquée (n= )

nombre de visites NT effectuées : *médiane, min-max*

Exclus pour retrait de consentement (n=	)

Analysés (n= )

Exclus des analyses (n= )

*Raisons d’exclusions* (n= )

**Analyse**

## 3.2. Description des patients à la visite de sélection et à l’inclusion

Les patients seront décrits globalement puis en fonction des violations au protocole :

- Patients inclus dans l’analyse (globalement puis selon le groupe de traitement) / patients non inclus / patients exclus après randomisation (pour autre raison que retrait de consentement), si applicable

- parmi les patients inclus dans l’analyse : patients avec un suivi complet / arrêt prématuré du suivi (pas de visite à 2 ans +/- 3 mois).

Les caractéristiques des patients seront décrites sans test de comparaison.

Les variables à décrire sont les suivantes :

- **Caractéristiques socio-démographiques :**
  - Âge à la visite de sélection
  - Sexe
  - Contraception
  - Poids, taille et IMC
- **Caractéristiques liées à la SEP**
  - Durée depuis la première poussée en années
  - Durée depuis le diagnostic SEP en années
  - Durée depuis le début de la phase progressive en années
  - Plus de deux poussées depuis le début de la maladie
  - Nombre de poussées
    - au cours des 6 mois précédant la visite de pré-inclusion
    - au cours de l’année précédant cette visite de sélection
    - au cours de la phase de progression
    - dans les deux années ayant précédé la phase de progression
  - Critères de Paty
  - Critères de Barkhof
- **Evaluation neurologique**
  - Score de périmètre de marche
  - Réduction du périmètre de marche au cours des 12 derniers mois
  - Score EDSS (à la pré-inclusion et à l’inclusion)
  - score du PASAT (à la pré-inclusion et à l’inclusion)
- **Historiques de maladies et de traitements**
  - Antécédents de maladie, chirurgie
  - Antécédents des traitements de fond (liés à la SEP) : Immunomodulateurs / Immunosuppresseurs /autres

## 3.3. Description des patients au cours du suivi

Les caractéristiques des patients seront décrites sans test de comparaison.

- Evaluation neurologique
  - Score de périmètre de marche
  - Score EDSS
  - Proportion de patients ayant progressé de 0.5 point ou 1 point de l’échelle EDSS
- Apparition de poussée depuis la visite précédente
- Score composite de la SEP (Multiple Sclerosis composite score : MSFC)
- z-score du 9 HPT,
- z-score du TM et z-score du PASAT
- Auto questionnaire de qualité de vie SEP59 (15 dimensions),
- auto questionnaire du retentissement de la SEP (MSIS)
- auto questionnaire de la marche (MSWS-12)

## 3.4. Analyse des critères de jugement

3.4.1. Critère de jugement principal
       3.4.1.1. Analyse principale

Le critère de jugement principal est le **délai d’aggravation du score EDSS**, estimé 2 ans après le début du traitement.

Les courbes de survenue d’une aggravation seront tracées à l’aide de la méthode de Kaplan-Meier

Un modèle de survie à fragilités sera construit pour prendre en compte l’effet des facteurs pronostiques connus sur l’efficacité du traitement.

Le facteur pronostique analysé en première intention est le centre, en effet aléatoire.

L’analyse en seconde intention inclura en plus les facteurs pronostiques suivants :

- l’existence de poussées dans les 12 mois précédant la pré-inclusion,

- la durée de la maladie à la date d’initiation du traitement,

Le modèle à fragilités prendra en compte chacun de ces facteurs pronostiques, ainsi que l’interaction entre le traitement et l’existence de poussées dans les 12 mois précédant la pré-inclusion, afin d’étudier une éventuelle différence d’effet du traitement en fonction de l’existence de poussées dans les 12 mois précédant la pré-inclusion.

Les patients non aggravés avant leur arrêt prématuré du suivi seront considérés comme censurés aux dernières nouvelles sur l'aggravation (c'est à dire le plus souvent à la dernière visite effectuée par le patient). La censure par intervalle est gérée par imputation du milieu de l'intervalle entre la dernière visite non aggravée et la première visite aggravée. (cf. §0))

3.4.1.2. Analyses de sensibilité

Une première analyse de sensibilité sera réalisée en considérant tous les patients ayant arrêté le suivi sans aggravation confirmée, comme aggravés à la date de dernières nouvelles.

Une deuxième analyse de sensibilité prendra en compte le fait que les patients aient pu sortir de l'étude en ayant fait une aggravation non constatée entre leur dernière visite et leur sortie d'étude avec un modèle multi-état (initial, aggravé, abandon - état absorbant) avec ajustement et sans sur les covariables.

Une troisième analyse de sensibilité reprendra l’analyse principale en considérant le centre en effet fixe.

3.4.2. Critères de jugement secondaires
- **Proportion de patients ayant progressé de 0.5 point ou 1 point de l’échelle EDSS** **à 2 ans et à 1 an** (1 si l’EDSS initial est ≤4, ou 0.5 point si l’EDSS initial est ≥5), **et proportion de patients ayant une aggravation confirmée de l’échelle EDSS au cours du suivi,**
- **et aggravation du périmètre de marche à la visite de fin de la période programmée de traitement (V19), et à la visite de 1 an (V13)**

Ces proportions seront décrites et comparées entre les 2 groupes de traitement selon les stratégies décrites en 2.2 et 2.3 pour les variables qualitatives.

Une description des délais d’observation du critère (délai entre la visite d’inclusion et la visite à 2 ans, ou la dernière visite avec score EDSS/périmètre de marche disponible) est réalisée afin de juger de la validité de l’imputabilité des données manquantes.

- **Score composite de la SEP (Multiple Sclerosis composite score : MSFC) et z-score du 9 HPT, z-score du TM et z-score du PASAT 3s à 2 ans et à 1 an**

Les z-scores et le MSFC seront décrits et comparés entre les 2 groupes de traitement selon les stratégies décrites en 2.2 et 2.3 pour les variables quantitatives.

- **Nombre de poussées pendant l’étude**

Le nombre de poussées observées durant l’étude sera décrit dans les deux groupes de patients selon la stratégie décrite en 2.2 et 2.3 pour les variables quantitatives.

Si un ajustement est nécessaire, une régression de poisson ou binomiale négative pourra être utilisée selon la distribution de la variable et la vérification des hypothèses sous-jacentes à ces modèles.

- **Tolérance au traitement**

La proportion d’événements indésirables graves et non graves survenus au cours du suivi dans chaque groupe de traitement sera décrite et comparée entre les groupes, selon les stratégies décrites 2.2 et 2.3 pour les variables qualitatives.

La fréquence de survenue de chacun de ces évènements, leur gravité et leur imputabilité aux traitements de l’essai seront décrites et comparées entre les groupes, selon les stratégies décrites en 2.2 et 2.3

L’analyse du délai de survenue d’un événement sera effectuée en prenant en compte uniquement la première survenue de l’événement. Les courbes de survenue d’événement seront tracées à l’aide de la méthode de Kaplan-Meier.

La première analyse portera sur l’ensemble des événements indésirables graves, quelle que soit leur imputabilité aux traitements de l’essai. Secondairement, une analyse portera uniquement sur les événements imputables aux traitements de l’essai, quelle que soit leur gravité. Enfin une analyse portera sur les événements graves imputables aux traitements de l’essai.

3.4.3. Critères d’évaluation exploratoires

Ces critères ne seront pas considérés comme des critères d’efficacité :

**Auto questionnaire de qualité de vie, auto questionnaire du retentissement de la SEP (MSIS) et auto questionnaire de la marche (MSWS-12) à S44 (visite 13) et S92 (visite 19)**

Les scores à un ou deux ans seront décrits et comparés entre les groupes de traitement selon les stratégies décrites en 2.2 et 2.3 pour les variables quantitatives.

Les mêmes stratégies que celles utilisées pour l’analyse des critères de jugement secondaires seront appliquées.

Les éosinophilies seront comparées entre les patients ayant atteint le CJP et ceux ne l’ayant pas atteint, dans chacun des groupes.

3.4.4. Critères exploratoires additionnels

Le nombre de réponses correctes consécutives maximum par test PASAT sera décrit et comparé entre les deux groupes selon les stratégies décrites en 2.2 et 2.3. pour les variables quantitatives. (sur données disponibles)
