# Supplementary material for: Double-Blind Controlled Randomized Trial of Cyclophosphamide versus Methylprednisolone in Secondary Progressive Multiple Sclerosis
Source: PLoS One. 2017 Jan 3;12(1):e0168834. doi: 10.1371/journal.pone.0168834 (PMC5207788; doi:10.1371/journal.pone.0168834)
Supplement: S2 File — (DOCX) [file pone.0168834.s002.docx]

**PROTOCOL SYNOPSYS**

Brief Title: Efficacy of Cyclophosphamide Versus Methylprednisolone in Patients With Secondary Progressive Multiple Sclerosis ( PROMESS )

Official Title: A Double-blind, Two-arm, Multicenter, Randomized Trial to Evaluate Efficacy of Cyclophosphamide Versus Methylprednisolone in Patients With Recent Secondary Progressive Multiple Sclerosis: P.R.OM.E.S.S Study

**Sponsor/Collaborators**

Sponsor: University Hospital, Bordeaux

Responsible Party: Sponsor

Collaborators: Ministry of Health, France

Oversight

FDA Regulated?: No

IND/IDE Protocol?: No

Review Board: Approval Status: Approved

Approval Number: 2005/09

Board Name: CPP Bordeaux A

Board Affiliation: French Ministry of Health (DGS)

Email: ccpprb.bxA@wanadoo.fr

Data Monitoring?: Yes

Plan to Share Data?:

Oversight Authorities: France: Afssaps - Agence française de sécurité sanitaire des produits de santé (Saint-Denis)

-

**Study Description**

**Brief Summary**: Preliminary not-controlled clinical studies of the efficacy of monthly intravenous cyclophosphamide administration in secondary progressive multiple sclerosis reported encouraging results, but no randomized controlled trial has been conducted so far.

The primary objective of this trial is to evaluate the efficacy of IV cyclophosphamide as compared to IV methylprednisolone administered every 4 weeks during 1 year and every 8 weeks during 1 year, on the delay to confirmed disability deterioration as assessed by the Expanded Disability Status Scale (EDSS) in patients with secondary progressive multiple sclerosis. The secondary objectives are to evaluate safety, tolerability and efficacy at 2 years on the Multiple Sclerosis Functional Composite (MSFC), the percentage of patients with disability deterioration (EDSS) and the number of relapses. An intention-to-treat statistical analysis will be carried out.

**Detailed Description**:

Background

Preliminary not-controlled clinical studies of the efficacy of monthly intravenous cyclophosphamide administration in secondary progressive multiple sclerosis reported encouraging results, but no randomized controlled trial has been conducted so far. A slight efficacy of Methylprednisolone has been reported in this indication.

Objectives

The primary objective is to evaluate the efficacy of IV cyclophosphamide on the prevention of disability deterioration in patients with secondary progressive multiple sclerosis.

The secondary objectives are to evaluate safety, tolerability and efficacy of IV cyclophosphamide on the Multiple Sclerosis Functional Composite (MSFC) and the number of relapses.

Study design:

Randomized double-blind two-arm controlled trial.

Intervention

Experimental group : IV cyclophosphamide infusion administered every 4 weeks during 1 year and every 8 weeks during 1 year.

Control group : IV methylprednisolone infusion administered every 4 weeks during 1 year and every 8 weeks during 1 year.

Outcomes

Primary outcome : delay to disability deterioration as assessed by the Expanded Disability Status Scale (EDSS: 0.5 or 1 point increase, depending on baseline score) evaluated every 4 weeks for one year, then every 8 weeks for one year.

Secondary outcomes :

- proportion of patients with disability deterioration (EDSS: 0.5 or 1 point increase, depending on baseline score),
- Multiple Sclerosis Functional Composite (MSFC) and the Z scores of MSFC three components,
- number of MS relapses,
- proportion of patients with adverse events and delay of occurrence of adverse events,
- Quality of life questionnaires
- Disability self-assessment questionnaires Main time of assessment : 2 years.

Sample size

360 patients

Statistical analysis

Intention-to-treat analysis.

**Statistical methods** :

Introduction
Data will be analyzed by intention to treat, that is to say that all patients who have been randomized will be analyzed in their group of randomization, even if they have never taken the treatment, if they have arrested him, or if they changed treatment. Interim analysis is not planned. For statistical tests, the risk of the first order is fixed at 5%. Statistical analyses will be performed with SAS software.

Description of enrollment and follow-up. :
The number of included patients, the curve of inclusions (evolution of the number of patients included between the first and the last inclusion), the number of theoretical visits and the number of actual visits will be presented and compared between groups

Characteristics of patients before treatment:
Patients will be described according to the group of treatment and according to protocol violations:

- Patients excluded from the analysis / Patients included in the analysis.
- Patients included in the analysis with minor violations of the protocol / Patients included in the analysis without minor violation of the protocol.
The characteristics of patients before treatment will be described by groups: frequencies for the qualitative variables; means, standard deviation, median, minimum and maximum for quantitative variables.

Analysis of the primary endpoint:
The primary endpoint is the time of worsening of the EDSS score, estimated 2 years after the start of treatment. It will be analyzed when all patients have completed the scheduled follow-up.

As a first step, the delay of worsening curves will be plotted using the Kaplan-Meier method and time of occurrence will be compared between treatment groups by the logrank test.

In a second step, a Cox model will be built to study the effect of prognostic factors on the efficiency of treatment.

The prognostic factors analysed are:
 - the existence of relapses in the 12 months before the beginning of the treatment, - the presence of gadolinium-enhanced lesions on MRI performed prior to initiation of treatment,
 - the disease duration at the time of initiation of treatment.
The Cox model will take into account each of these 3 prognostic factors as well as the interaction between treatment and the existence of relapses or the presence of gadolinium-enhanced lesions on MRI to investigate a possible difference of effect of treatments based on the existence of these prognostic factors. In the event of statistically significant interaction, the results of both treatments will be described and compared in each subgroup of prognostic factor.

Analysis of the secondary endpoints:
- Proportion of patients deteriorated at 2 years:

The proportion of patients worsened at 2 years will be described in the 2 treatment groups (proportion and 95% of this estimate confidence interval). Comparison of the proportions of patients worsened at 2 years will be conducted by a Chi-square test if theoretical numbers are sufficient (n≥5) or by Fisher's test otherwise.

- Z-scores and MSFC : z scores will be calculated using as reference population the population at baseline according to international recommendations (Cutter et al ., 1999). The mean z-scores and the mean MSFC will be compared by a Student's test if the implementing conditions are met (homogeneous variances, normal distributions). If variances are not homogeneous, a Student's test for unequal variances will be used. If the distribution is not normal, a Mann-Whitney test will be used.

- Number of relapses (exacerbations) :

The number of exacerbations will be described in the two groups of patients (mean and 95% confidence interval, median, minimum-maximum). The means of the number exacerbations observed in the two groups will be compared by a Student's test if the validity of the test conditions are met (normal distribution, homogeneous variances). If variances are not homogeneous, a Student's test for unequal variances will be used. If the distribution is not normal, a Mann-Whitney test will be used.

- Comparison groups for safety :
The proportion of serious adverse events (SAE) and non-serious AE occurring during follow-up in each treatment group will be described, and groups will be compared for the frequency of occurrence of these events, their severity and their accountability to the study treatments. Comparisons of proportions will be carried out by a Chi-square tes if theoretical numbers are sufficient (n≥5) or by Fisher's test otherwise.

The time of occurrence of an event will be analyzed taking into account only the first occurrence of the event. The event curves will be plotted using the Kaplan-Meier method. The comparison tests of the time of occurrence of the events between treatment groups will be carried out by the log-rank test.

- Analysis of exploratory judgement criteria:
  - Quality of life at 2 years patient related outcome (PEO), handicap PRO (MSIS) and self evaluation of ambulmation at one and two years
- Number of eosiniophils

To describe the evolution of each score during the follow-up, the means scores will be compared between the two treatment groups by analysis of variance with repeated in time measurements.

For each of the secondary criteria (proportion of patients worsened at 2 years, Z-scores, MSFC, number of relapses), the same analyses will be made at 6 months, as well as 44 weeks. Analysis of variance with repeated in time measurements will be carried out to describe the evolution during the follow-up period for each of these criteria.

**Conditions**

Multiple Sclerosis, Chronic Progressive

Keywords: Multiple Sclerosis, Chronic Progressive

Cyclophosphamide

Methylprednisolone

Randomized Controlled Trials

Double-Blind Study

Study Design

Study Type: Interventional

Primary Purpose: Treatment

Study Phase: Phase 3

Intervention Model: Parallel Assignment

Number of Arms: 2

Masking: Double Blind (Subject, Investigator)

Allocation: Randomized

Endpoint Classification: Efficacy Study

Arms and Interventions

Arms Assigned Interventions

Experimental: IV cyclophosphamide infusion administered every 4 weeks during 1 year and every 8 weeks during 1 year.

Active Comparator: Methylprednisolone IV infusion administered every 4 weeks during 1 year and every 8 weeks during 1 year.

**Outcome Measures**

Primary Outcome Measure:

Delay to disability deterioration as assessed by the Expanded Disability Status Scale (EDSS: 0.5 or 1 point increase, depending on baseline score) [Time Frame: every 4 weeks for one year, then every 8 weeks for one year]

Secondary Outcome Measure:

- Proportion of patients with disability deterioration (EDSS: 0.5 or 1 point increase, depending on baseline score) [Time Frame: every month during one year then every two months during the 2nd year]
- Multiple Sclerosis Functional Composite (MSFC) and the Z scores of MSFC three components [Time Frame: Visit number 1, 2, 13(at one year),19 (at two years) and 20 (last visit)]
- Number of MS relapses [Time Frame: all along the follow up period]
- Proportion of patients with adverse events and delay of occurrence of adverse events [Time Frame: all along the follow up period]
- Quality of life questionnaires [Time Frame: visit 2, 13(at one year) and 19 (at two years)]
- Disability self-assessment questionnaires [Time Frame: visite 2, 13 et 19]

**Eligibility**

Minimum Age: 18 Years

Maximum Age: 65 Years

Gender: Both

**Inclusion Criteria**:

• Multiple sclerosis (MS) subjects (McDonald et al criteria),

• Aged 18 to 65

• Diagnosis of secondary progressive MS ( Lublin and Reingold criteria)

• Progressive deterioration phase of at least 6 months and less than 4 years.

• Reduction of walking capacity and increase EDSS not ascribed to consequence of relapses (at least 0.5 point) in the last 12 months

• EDSS between 4.0 and 6.5 included

• Female participating must use contraceptives while on study drug

• Written informed consent

• Patient protected by French social security system

**Exclusion Criteria:**

• Others diseases interfering with MS or treatment

• Recent history (within the previous 2 years) of drug or alcohol abuse.

• Patients with psychiatric illnesses who are unable to provide written, informed consent prior to any testing under this protocol

• Hemorrhagic cystitis

• Pregnant or lactating women

• Known allergy at cyclophosphamide, corticoids and in particular methylprednisolone

• Persistent infectious diseases

• Patients with bladder permanent catheterization

• Known history of cardiac arrhythmia after methylprednisolone intravenous treatment

• Abnormal screening/baseline blood tests exceeding any of the limits defined below : Hb < 9g/dl or Total white blood cell count less than 3 000/mm3 or lymphocytes count less than 900/ mm3 or Platelet count less than 125 000/mm3

• Gastric or duodenal ulcer in evolution

• Gut diverticulosis

• Diabetes mellitus

• Known history of active hepatitis (ASAT >3 X ULN)

• Known history of renal failure (creatinine level > 180 μmol/L)

• Psychosis

• Current or past (< 3 months) participation in another drug trial

• Prior use of cyclophosphamide, lymphoid irradiation, monoclonal antibodies anti

CD4 or anti CD52 or anti-VLA-4 therapies, cladribine ou cyclosporine A

• Other clinical types of MS : Secondary progressive phase evolving for more than 4 years ; Remittent type of MS without progression between relapses ; Primary progressive type of MS

• Use of interferon beta, methotrexate or imurel in the month prior to study.

• Treatment with intravenous monthly corticoids in the year prior to study.

• Treatment with corticoids (3 to 5 days) in the 2 month prior to study.

**Contacts/Locations**

Study Officials:

Bruno Brochet, Professor, Study Principal Investigator

University Hospital, Bordeaux, France

Paul Perez, Dr, Study Chair

University Hospital, Bordeaux, France

Locations: France

Hôpital Pellegrin, Département de neurologie

Bordeaux, France, 33076

CH d'Angoulême Girac

Saint Michel, France, 16470

CH de la Cote Basque

Bayonne, France, 64109

CHU Besançon

Besançon, France, 25030

CHU Caen

Caen, France, 14033

Hôpital Gabriel Montpied

Clermont Ferrand, France, 63003

AP HP Henri Mondor

Créteil, France, 94010

CHU Dijon

Dijon, France, 21033

CHU Lille Hôpital Salengro

Lille, France, 59037

GHICL Hôpital St. Philibert

Lomme, France, 59462

CHU Limoges

Limoges, France, 87042

(CHU Lyon) Hôpital neurologique

Lyon, France, 69394

Hôpital La Timone

Marseille, France, 13385

CHU Nancy Hôpital central

Nancy, France, 54035

CHU Nice Hôpital Pasteur

Nice, France, 06002

(CHU Nîmes) Hôpital Caremeau

Nîmes, France, 30029

Centre Hospitalier de Pau

- Page 6 of 6 -

Pau, France, 64046

(CHU Reims) Hôpital Robert Debré

Reims, France, 51092

CHU Ponchaillou

Rennes, France, 35033

Fondation Rothschild

Paris, France, 75019

(AP HP) Hôpital Tenon

Paris, France, 75970

(CHRU Starsbourg) Hôpital civil

Strasbourg, France, 67091

(CHR Metz-Thionville) Hôpital Notre Dame de Bon Secours

Metz, France, 57038

(CHU Montpellier), Hôpital de Gui de Chauliac

Montpellier, France, 34295

CHU de POISSY

Poissy, France, 78300

Hôpital Guillaume et René Laënnec

Nantes, France, 44093

References

Citations:

Links:

Study Data/Documents:

**SUMMARY OF CHANGES :**

Twelve amendments have been submitted and approved by the Ethic Committee (CPP), during the study between November 2005 (1st amendment) and November 2009 (12th amendment)..

Changes in the name of clinical investigators and study personal have been submitted in amendments 1, 2, 3, 4, 6, 7, 8, 11 and 12.

Changes in procedures and organisation of the study without impact on the conduct of the study (for example change in committees, medication circuit etc...) have been submitted in amendments 1, 2, 3, 6 7, 9, 10 and11.

Significant changes in the protocol have been made in amendments 1, 2, 3, 4, 5, 10 and 12.

- Amendment 1 (November 2005) gave precision on the role of the prescribing physician.
- Amendment 2 (January 2006) indicated that a previous treatment with azathioprine in the month before randomisation is not allowed.
- Amendment 3 (April 2006) modified inclusion criteria: the maximum duration of the progressive phase before randomisation was change from 3 years to 4 years; Skin test for TB was recommended but no longer mandatory.
- Amendment 4 (April 2006) modified inclusion criteria: the maximum EDSS allowed at baseline was changed from 6.0 to 6.5. Inclusion of patients with only one exacerbation before the progressive stage (so-called “transitional progressive”) was allowed.
- Amendment 5 (October 2006) modified inclusion criteria: the maximum walking distance required at baseline was changed from 100 meters to 50 meters.
- Amendment 10 (February 2008) specified the conditions for unblinding after the end of the study.
- Amendment 12 (October 2009) added a few exploratory end-points (walking distance, PASAT correct answers, EDSS AUC).

**Statistical analysis plan,**The original statistical analysis plan is the Statistical analysis section of the original protocol (translated)
Needed sample size :
The primary outcome measure is the delay to disability deterioration as assessed by the Expanded Disability Status Scale (EDSS): 0.5 or 1 point increase, depending on baseline score: >1 point when baseline EDSS was 4 or 4.5 and >0.5 point whne baseline EDSS was 5, 5.5 or 6.0.
 This deterioration has to be confirmed at the next visit at least 4 months after the first visit showing the deterioration or after 2 months if deterioration is observed at the final visit of the treatment period. The deterioration delay will be calculated by the difference between the first visit showing deterioration and the baseline treatment visit (first administration).
The sample size calculation has been made using the N-Query® software (v 4.0). The proportion of patients without deterioration is expected to be 75% in the CPM group after two years according to Hohol et al. (1999) and 60% in the MP group ( Kinkel, 1999).
By comparing the delay of deterioration curves in each group by a bilateral logrank test (α = 5% and (1-β) = 80%) the number to be included in each group is at least 155 patients (2 groups with the same size). Assuming that 5% of patients will be lost for follow-up, 180 patients will be included in each group.

CHANGES

The main changes in comparison with the final Analysis Plan were the introduction of two additional sensitivity analyses: one considering all patients who stopped their follow-up as having experienced deterioration of EDSS (worst case analysis for the comparison of proportions of deteriorated patients at 2 years), and the second one using multistate models to take into account the premature stopping of treatment and follow-up. This was decided after a blind data review (i.e. before unblinding) because of the high overall proportion of patients who stopped treatment and follow-up prematurely.
Minor changes included the possible use of a frailty model, instead of a standard Cox model, to better take into account the centre effect as a random effect, in case of significant variance of the center effect. As it was not the case the standard Cox model was used."
