# Supplementary material for: Double-Blind Controlled Randomized Trial of Cyclophosphamide versus Methylprednisolone in Secondary Progressive Multiple Sclerosis
Source: PLoS One. 2017 Jan 3;12(1):e0168834. doi: 10.1371/journal.pone.0168834 (PMC5207788; doi:10.1371/journal.pone.0168834)
Supplement: S3 File — (PDF) [file pone.0168834.s003.pdf]

ClinicalTrials.gov Protocol Registration and Results System (PRS) Receipt
Release Date: 03/14/2012

ClinicalTrials.gov ID: NCT00241254

Study Identification

Unique Protocol ID: 9408-04

Brief Title: Efficacy of Cyclophosphamide Versus Methylprednisolone in Patients With Secondary Progressive Multiple Sclerosis (PROMESS)

Official Title: A Double-blind, Two-arm, Multicenter, Randomized Trial to Evaluate Efficacy of Cyclophosphamide Versus Methylprednisolone in Patients With Recent Secondary Progressive Multiple Sclerosis: P.R.OM.E.S.S Study

Secondary IDs: 2004-005

Study Status

Record Verification: March 2012

Overall Status: Completed

Study Start: December 2005

Primary Completion: March 2010 [Actual]

Study Completion: March 2012 [Actual]

Sponsor/Collaborators

Sponsor: University Hospital, Bordeaux

Responsible Party: Sponsor

Collaborators: Ministry of Health, France

Oversight

FDA Regulated?: No

IND/IDE Protocol?: No

Review Board: Approval Status: Approved
Approval Number: 2005/09
Board Name: CPP Bordeaux A
Board Affiliation: French Ministry of Health (DGS)
Phone:
Email: ccpprb.bxA@wanadoo.fr

Data Monitoring?: Yes

Plan to Share Data?:

Oversight Authorities: France: Afssaps - Agence française de sécurité sanitaire des produits de santé (Saint-Denis)

Study Description

Brief Summary: Preliminary not-controlled clinical studies of the efficacy of monthly intravenous cyclophosphamide administration in secondary progressive multiple sclerosis reported encouraging results, but no randomized controlled trial has been conducted so far. The primary objective of this trial is to evaluate the efficacy of IV cyclophosphamide as compared to IV methylprednisolone administered every 4 weeks during 1 year and every 8 weeks during 1 year, on the delay to confirmed disability deterioration as assessed by the Expanded Disability Status Scale (EDSS) in patients with secondary progressive multiple sclerosis. The secondary objectives are to evaluate safety, tolerability and efficacy at 2 years on the Multiple Sclerosis Functional Composite (MSFC), the percentage of patients with disability deterioration (EDSS) and the number of relapses. An intention-to-treat statistical analysis will be carried out.

Detailed Description: Background

Preliminary not-controlled clinical studies of the efficacy of monthly intravenous cyclophosphamide administration in secondary progressive multiple sclerosis reported encouraging results, but no randomized controlled trial has been conducted so far. A slight efficacy of Methylprednisolone has been reported in this indication.

Objectives

The primary objective is to evaluate the efficacy of IV cyclophosphamide on the prevention of disability deterioration in patients with secondary progressive multiple sclerosis.

The secondary objectives are to evaluate safety, tolerability and efficacy of IV cyclophosphamide on the Multiple Sclerosis Functional Composite (MSFC) and the number of relapses.

Study design

Randomized double-blind two-arm controlled trial.

Intervention

Experimental group : IV cyclophosphamide infusion administered every 4 weeks during 1 year and every 8 weeks during 1 year.

Control group : IV methylprednisolone infusion administered every 4 weeks during 1 year and every 8 weeks during 1 year.

Outcomes

Primary outcome : delay to disability deterioration as assessed by the Expanded Disability Status Scale (EDSS: 0.5 or 1 point increase, depending on baseline score) evaluated every 4 weeks for one year, then every 8 weeks for one year.

Secondary outcomes : proportion of patients with disability deterioration (EDSS: 0.5 or 1 point increase, depending on baseline score), Multiple Sclerosis Functional Composite (MSFC) and the Z scores of MSFC three components, number of MS relapses, proportion of patients with adverse events and delay of occurrence of adverse events, quality of life questionnaires.

- Quality of life questionnaires
- Disability self-assessment questionnaires Main time of assessment : 2 years.

Sample size

360 patients

Statistical analysis

Intention-to-treat analysis.

Conditions

Conditions: Multiple Sclerosis, Chronic Progressive

Keywords: Multiple Sclerosis, Chronic Progressive
Cyclophosphamide
Methylprednisolone
Randomized Controlled Trials
Double-Blind Study

Study Design

Study Type: Interventional

Primary Purpose: Treatment

Study Phase: Phase 3

Intervention Model: Parallel Assignment

Number of Arms: 2

Masking: Double Blind (Subject, Investigator)

Allocation: Randomized

Endpoint Classification: Efficacy Study

Enrollment: 138 [Actual]

Arms and Interventions

Arms	Assigned Interventions
Experimental: 1<br>Cyclophosphamide	Drug: Cyclophosphamide (drug)<br>IV cyclophosphamide infusion administered every 4 weeks during 1 year and every 8 weeks during 1 year.
Active Comparator: 2<br>Methylprednisolone	Drug: Methylprednisolone (drug)<br>Control group : IV methylprednisolone infusion administered every 4 weeks during 1 year and every 8 weeks during 1 year.

Outcome Measures

Primary Outcome Measure:

1. Delay to disability deterioration as assessed by the Expanded Disability Status Scale (EDSS: 0.5 or 1 point increase, depending on baseline score)
   [Time Frame: every 4 weeks for one year, then every 8 weeks for one year] [Safety Issue: No]

Secondary Outcome Measure:

2. Proportion of patients with disability deterioration (EDSS: 0.5 or 1 point increase, depending on baseline score)
   [Time Frame: every month during one year then every two months during the 2nd year] [Safety Issue: No]
3. Multiple Sclerosis Functional Composite (MSFC) and the Z scores of MSFC three components
   [Time Frame: Visit number 1, 2, 13(at one year),19 (at two years) and 20 (last visit)] [Safety Issue: No]
4. Number of MS relapses
   [Time Frame: all along the follow up period] [Safety Issue: No]
5. Proportion of patients with adverse events and delay of occurrence of adverse events
   [Time Frame: all along the follow up period] [Safety Issue: Yes]
6. Quality of life questionnaires

[Time Frame: visit 2, 13(at one year) and 19 (at two years)] [Safety Issue: No]

7. Disability self-assessment questionnaires
   [Time Frame: visite 2, 13 et 19] [Safety Issue: No]

Eligibility

Minimum Age: 18 Years

Maximum Age: 65 Years

Gender: Both

Accepts Healthy Volunteers?: No

Criteria: Inclusion Criteria:

- Multiple sclerosis (MS) subjects (Mc Donald et al criteria),
- Aged 18 to 65
- Diagnosis of secondary progressive MS (Lublin and Reingold criteria)
- Progressive deterioration phase of at least 6 months and less than 4 years.
- Reduction of walking capacity and increase EDSS not ascribed to consequence of relapses (at least 0.5 point) in the last 12 months
- EDSS between 4.0 and 6.5 included
- Female participating must use contraceptives while on study drug
- Written informed consent
- Patient protected by French social security system

Exclusion Criteria:

- Others diseases interfering with MS or treatment
- Recent history (within the previous 2 years) of drug or alcohol abuse.
- Patients with psychiatric illnesses who are unable to provide written, informed consent prior to any testing under this protocol
- Hemorrhagic cystitis
- Pregnant or lactating women
- Known allergy at cyclophosphamide, corticoids and in particular methylprednisolone
- Persistent infectious diseases
- Patients with bladder permanent catheterization
- Known history of cardiac arrhythmia after methylprednisolone intravenous treatment
- Abnormal screening/baseline blood tests exceeding any of the limits defined below : Hb < 9g/dl or Total white blood cell count less than 3 000/mm3 or lymphocytes count less than 900/ mm3 or Platelet count less than 125 000/mm3
- Gastric or duodenal ulcer in evolution
- Gut diverticulosis
- Diabetes mellitus
- Known history of active hepatitis (ASAT >3 X ULN)
- Known history of renal failure (creatinine level > 180 µmol/L)
- Psychosis
- Current or past (< 3 months) participation in another drug trial
- Prior use of cyclophosphamide, lymphoid irradiation, monoclonal antibodies anti CD4 or anti CD52 or anti-VLA-4 therapies, cladribine ou cyclosporine A
- Other clinical types of MS : Secondary progressive phase evolving for more than 4 years ; Remittent type of MS without progression between relapses ; Primary progressive type of MS
- Use of interferon beta, methotrexate or imurel in the month prior to study.
- Treatment with intravenous monthly corticoids in the year prior to study.
- Treatment with corticoids (3 to 5 days) in the 2 month prior to study.

Contacts/Locations

Study Officials: Bruno Brochet, Professor
Study Principal Investigator
University Hospital, Bordeaux, France

Paul Perez, Dr
Study Chair
University Hospital, Bordeaux, France

Locations: France
Hôpital Pellegrin, Département de neurologie
Bordeaux, France, 33076

CH d'Angoulême Girac
Saint Michel, France, 16470

CH de la Cote Basque
Bayonne, France, 64109

CHU Besançon
Besançon, France, 25030

CHU Caen
Caen, France, 14033

Hôpital Gabriel Montpied
Clermont Ferrand, France, 63003

AP HP Henri Mondor
Créteil, France, 94010

CHU Dijon
Dijon, France, 21033

CHU Lille Hôpital Salengro
Lille, France, 59037

GHICL Hôpital St. Philibert
Lomme, France, 59462

CHU Limoges
Limoges, France, 87042

(CHU Lyon) Hôpital neurologique
Lyon, France, 69394

Hôpital La Timone
Marseille, France, 13385

CHU Nancy Hôpital central
Nancy, France, 54035

CHU Nice Hôpital Pasteur
Nice, France, 06002

(CHU Nîmes) Hôpital Caremeau
Nîmes, France, 30029

Centre Hospitalier de Pau

Pau, France, 64046

(CHU Reims) Hôpital Robert Debré
Reims, France, 51092

CHU Ponchaillou
Rennes, France, 35033

Fondation Rothschild
Paris, France, 75019

(AP HP) Hôpital Tenon
Paris, France, 75970

(CHRU Starsbourg) Hôpital civil
Strasbourg, France, 67091

(CHR Metz-Thionville) Hôpital Notre Dame de Bon Secours
Metz, France, 57038

(CHU Montpellier), Hôpital de Gui de Chauliac
Montpellier, France, 34295

CHU de POISSY
Poissy, France, 78300

Hôpital Guillaume et René Laënnec
Nantes, France, 44093

References

Citations:

Links:

Study Data/Documents:

U.S. National Library of Medicine | U.S. National Institutes of Health | U.S. Department of Health & Human Services
